# Switchable Synthesis of Tritylone Alcohols and 2-Benzoylbenzoate Esters from Spiroindane-1,3-diones

**DOI:** 10.1021/acs.joc.4c01296

**Published:** 2024-08-12

**Authors:** Jen-Yu Kuan, Jing-Huei Chen, Jeng-Liang Han

**Affiliations:** Department of Chemistry, National Chung Hsing University, Taichung City, Taiwan 40227, Republic of China

## Abstract

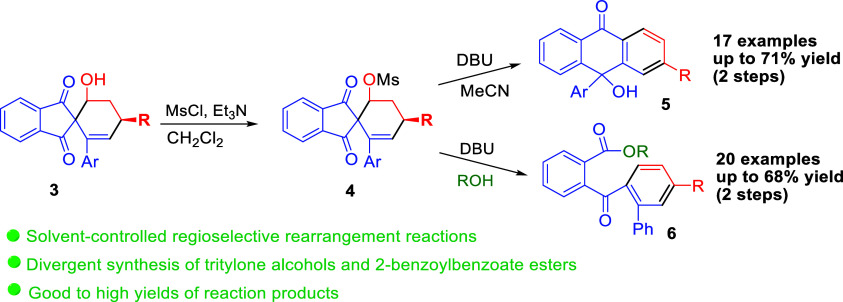

A solvent-controlled regioselective rearrangement reaction of spiroindane-1,3-diones with a leaving group has been developed. In acetonitrile solvent, the spiroindane-1,3-diones **3** were rearranged to provide tritylone alcohols, while 2-benzoylbenzoate ester derivatives were obtained if the reactions were performed in alcohols.

## Introduction

**Scheme 1:**
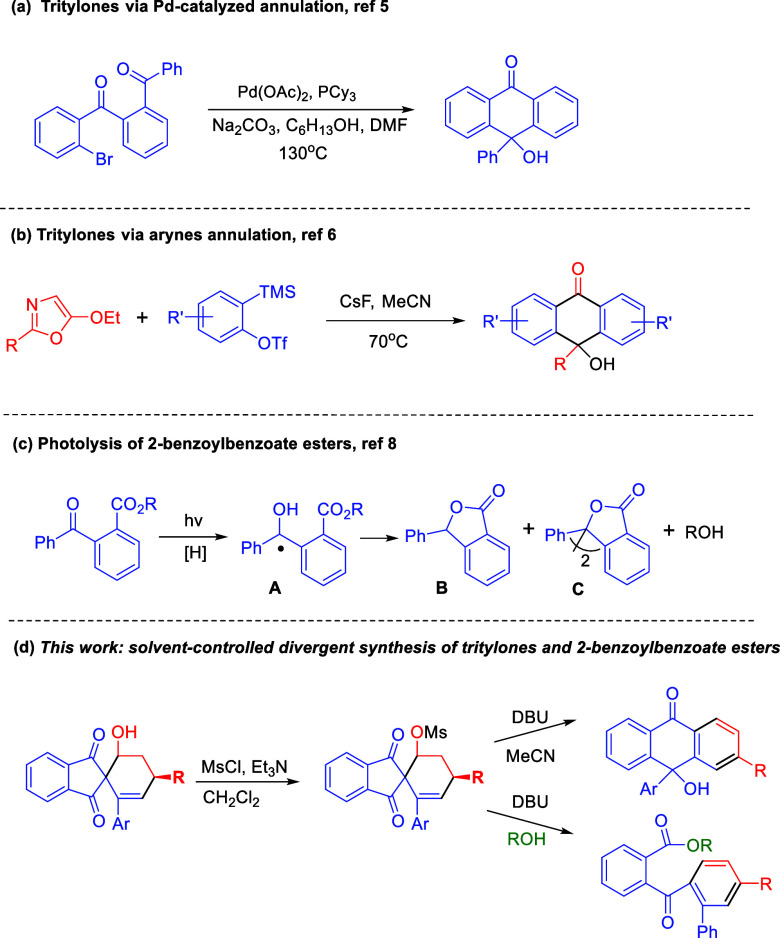
Research Background

Tritylone derivatives^1^ constitute an important class of organic polycyclic system that embedded in a plethora of synthetic compounds, which were used as precursors for the synthesis of fluorophores for bioimaging applications^2^ and organic light-emitting diodes.^3^ 9-Phenyl-9-tritylone ethers, which are prepared from the protection of 9-hydroxy-9-phenanthrones (tritylone alcohols), have been employed as photoremovable protecting groups for primary and secondary alcohols.^1g^ However, the methods for the synthesis of 9-hydroxy-9-phenanthrene are quite limited, and only symmetric products can be prepared. Classical synthetic routes toward tritylone alcohols employ the addition of Grignard reagents to anthraquinone.^1a,4^ In 2000, Yamamoto and co-workers reported a palladium-catalyzed intramolecular annulation of aryl bromides to ketones, affording 9-hydroxy-9-phenanthrene in 89% yield (Scheme 1a).^5^ Recently, Singh and co-workers developed an expeditious synthesis of tritylone alcohols by employing a cascade reaction of arynes with 5-ethoxyoxazoles (Scheme 1b).^6^ Hence, the development of efficient synthetic methodologies for synthesis of tritylone alcohols is still highly in demand.

Another class of photoremovable compounds for protection of primary and secondary alcohols are esters of 2-benzoylbenzoic acid,^7^ which were first described by Porter et al.^8^ and later developed by other research groups.^9^ These 2-benzoylbenzoate derivatives have been applied to photochemically trigger the activity of serine proteases,^10^ control release of fragrances,^9a,11^ or act as radical photoinitiators.^12^ The general mechanistic insight into the photolysis of 2-benzoylbenzoate esters involves two possible pathways (Scheme 1c).

First, the excited triplet state of the precursor reacts by (intermolecular) hydrogen abstraction from the solvent to form an intermediate radical **A**, followed by abstraction of a second hydrogen, and intramolecular cyclization yields the desired alcohol and a lactone byproduct **B**. The second possible pathway involves the dimerization of radical **A**, and then the intramolecular cyclization releases the alcohol and dimeric lactone **C**.

**Scheme 2:**
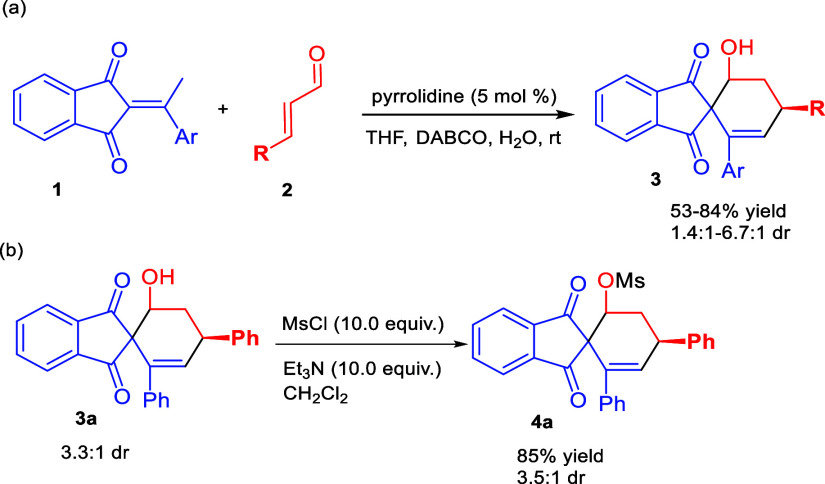
Synthesis of Spiroindane-1,3-diones and Mesylation

Divergent synthesis offers a rapid means to access a variety of structurally diverse chemical frameworks, which are essential for constructing a library of molecules for drug screening and discovery.^13^ Nonetheless, controlling the selectivity, reactivity, and compatibility from the same starting materials presents a formidable challenge, especially when dealing with competing reaction pathways. These reactions can be controlled by the choice of catalysts or modification of reaction conditions.^14,15^ We recently reported the organocatalyzed divergent vinylogous and Friedel–Crafts reaction.^16^ Continuing our efforts on exploring efficient methodologies based on the concept of divergent synthesis, we herein report a divergent synthesis of tritylone alcohols and 2-benzoylbenzoate esters through unprecedented solvent-controlled rearrangement reactions with spiroindane-1,3-diones, which were generated from 2-alkylidene 1,3-indandiones and enals according to our previous work with pyrrolidine as the catalyst (Scheme 2a).^17^

## Results and Discussion

Reaction of spiroindane-1,3-dione **3a** with MsCl under basic conditions provided the mesylation product **4a** for subsequent reaction condition optimization (Scheme 2b). As shown in Table 1, an initial experiment comprising 4**a** in MeCN at 70 °C was conducted using DBU as the base. The desired tritylone alcohol **5a** was isolated in a 37% yield (entry 1). A series of strong organic and inorganic bases were screened, and none of them gave better results than DBU (entries 2–5). Examination of the amounts of base loading showed that 15 equiv of DBU gave the highest reaction yield (80%) (entries 6–9). The screening of several polar and apolar solvents such as toluene, EA, and 1,2-DCE showed lower reaction yield outcomes as compared to reactions conducted in MeCN (entries 10–12). Intriguingly, 2-benzoylbenzoate derivative **6a** was obtained in 20% yield when EtOH was used as the solvent (entry 13). Further examination of the amounts of base loading showed that 2.5 equiv of DBU gave the highest reaction yield (68%) (entries 14–16). Decreasing or increasing the reaction concentration did not improve the reaction yield (entries 17 and 18). Hence, the final optimal conditions for tritylone alcohols **5** were chosen by conducting the reaction at 70 °C in MeCN (1.0 mL) with 15 equiv of DBU as the base (entry 8). For the formation of 2-benzoylbenzoate derivatives **6**, the final optimal reaction conditions were chosen by conducting the reaction with 2.5 equiv of DBU at 70 °C in 1.0 mL of EtOH (entry 16).

**Table 1:**
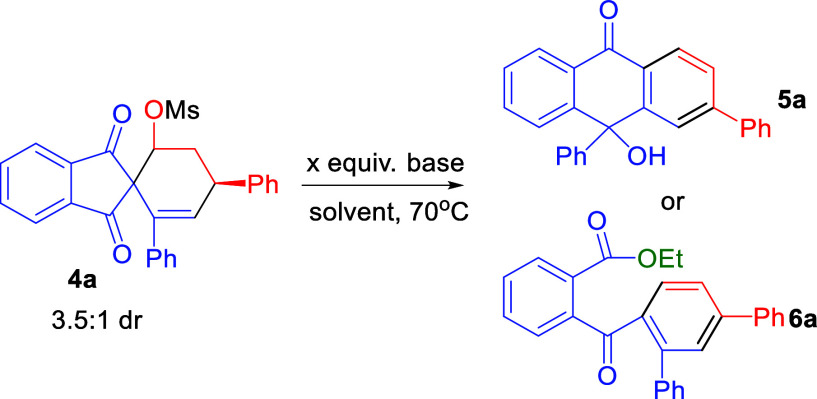
Screening for the Reaction Conditions^a^

entry	*x* (equiv)	base	solvent	time (h)	(**5a**) yield (%)^b^	(**6a**) yield (%)^b^
1	1.5	DBU	MeCN	72	37	
2	1.5	TMG	MeCN	72	37	
3	1.5	Cs_2_CO_3_	MeCN	48	32	
4	1.5	*t*-BuOK	MeCN	72	30	
5	1.5	NaH	MeCN	72	29	
6	5.0	DBU	MeCN	72	53	
7	10	DBU	MeCN	24	69	
8	15	DBU	MeCN	12	80	
9	20	DBU	MeCN	6	74	
10	15	DBU	toluene	24	19	
11	15	DBU	EA	24	N.R.	
12	15	DBU	1,2-DCE	24	N.R.	
13	15	DBU	EtOH	12		20
14	10	DBU	EtOH	12		45
15	5	DBU	EtOH	12		55
16	2.5	DBU	EtOH	18		68
17^c^	2.5	DBU	EtOH	24		59
18^d^	2.5	DBU	EtOH	12		58

a Unless otherwise noted, the solution of 0.1 mmol of **4a**, 1.5–20 equiv of base in 1.0 mL of solvent was stirred at 70 °C for indicated time.

b Isolated yields.

c 2.0 mL of EtOH.

d 0.5 mL of EtOH.

**Scheme 3:**
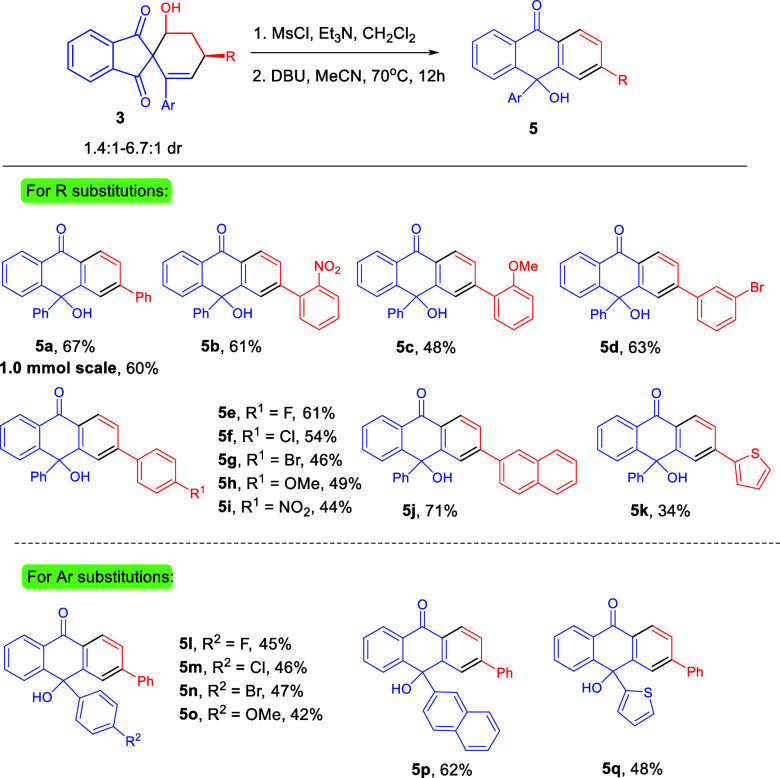
Scope of Tritylone Alcohols **5** The reaction was carried out by using 0.1 mmol of **3**, see the Supporting Information for detailed experimental procedure. Isolated yields for two steps.

With the optimal conditions established, we next explored the general substrate scope for the synthesis of tritylone alcohols **5**. Because some mesylated products were not stable during isolation, the mesylated compounds underwent to next step without further purification. As shown in Scheme 3, all reactions generally proceeded well to deliver the products in good isolated yields after 2 steps. A larger-scale reaction (1.0 mmol) of **3a** could smoothly take place to give product **5a** in slightly decreased yield (60%). The *o*-position of the phenyl group of the R substitutions has good substrate tolerance, and the corresponding products could be obtained in good yields (**5b** and **5c**). Substituent was also well tolerated on the *m*-position of the phenyl group of the R substitutions, and the yield of the product **5d** was not obviously changed.

Both electron-withdrawing and electron-donating substituents could be installed on the p-position of the phenyl group of the R substitutions, with the corresponding products afforded in slightly decreased yields compared to the *m*- and *o*-positions of the phenyl group of the R substitutions (**5e** to **5i**). The structure of **5** was confirmed using single-crystal X-ray diffraction analysis of **5g** (CCDC2348937).^18^

The naphthyl substituent of the R substitutions was found to be a compatible substrate in this reaction and afforded the product **5j** with the highest reaction yield (71%). In contrast, the thienyl substituent of the R substitutions delivered the lowest reaction yield of product **5k** (34%).

We then turned our attention to the synthesis of tritylone alcohols **5** bearing different Ar substituents with spiroindane-1,3-diones **3**. In all cases, the reactions proceeded well, obtaining **5l**–**5q** with good to high yields (42 to 62% for two steps). The electronic effect of *p*-position of the phenyl group of the Ar substitutions had no influence to the reaction outcome and provided the corresponding products **5l**–**5o** with similar yields. The naphthyl substituent of the Ar substitutions was found to work well in this reaction and afforded product **5p** with the highest reaction yield (62%). The thienyl substituent of the Ar substitutions had good substrate tolerance and delivered the corresponding product **5q** in 48% yield. We had tried the rearrangement reaction with the ethyl substituent (R = C_2_H_5_) of compound **3**. However, no desired product was observed.

**Scheme 4:**
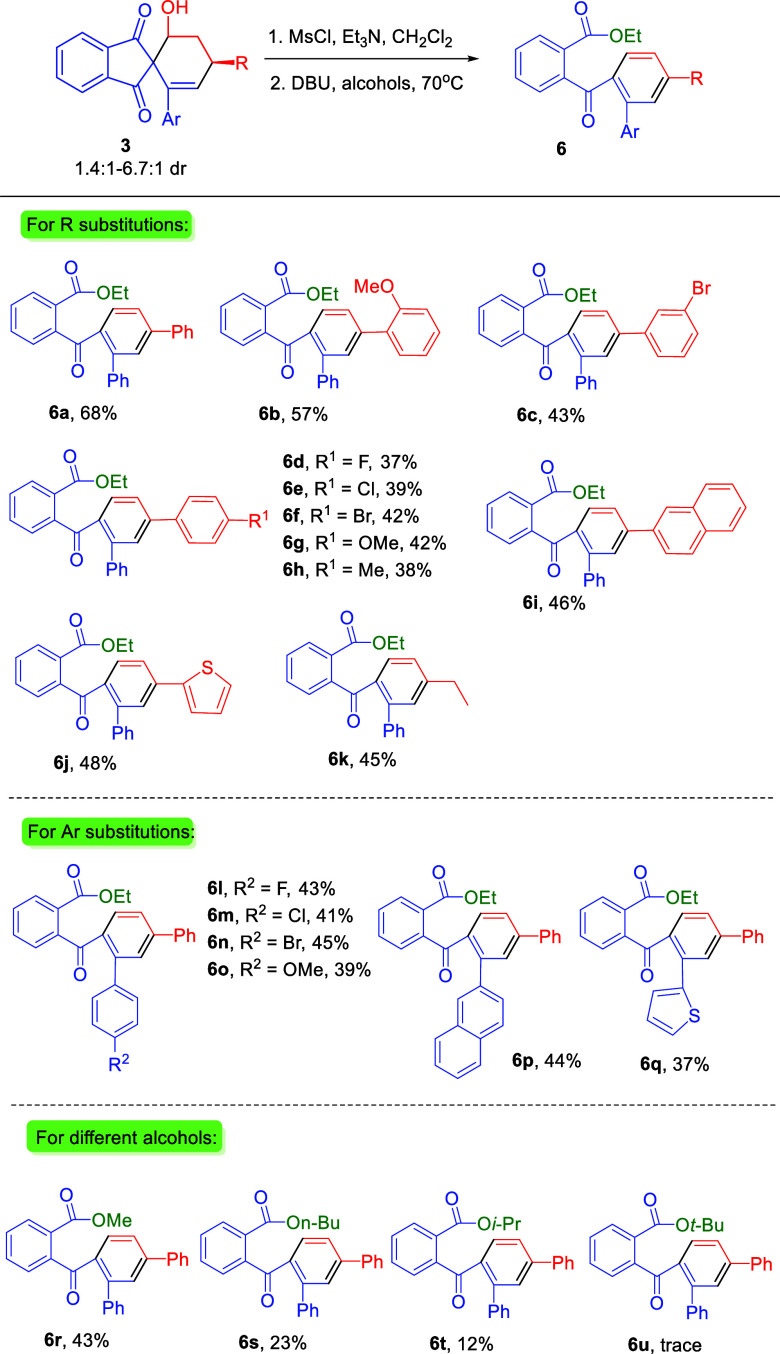
Scope of 2-Benzoylbenzoate Esters **6** The reaction was carried out by using 0.1 mmol of **3**, see the Supporting Information for detailed experimental procedure. Isolated yields for two steps.

We then focused our attention on establishing the scope with respect to the synthesis of 2-benzoylbenzoate derivatives **6**. As shown in Scheme 4, the *o*-position of the phenyl group of the R substitutions has good substrate tolerance, and the corresponding product **6b** could be obtained in a good yield after two steps (57%). The electronic effect of *m*- and *p*-positions of the phenyl group of the R or Ar substitutions had no influence to the reaction outcome and provided the corresponding products **6c**–**j** and **6l**–**q**, respectively, with slightly reduced yields (37–48%). The alkyl substituent of the R substitutions was found to be a compatible substrate in this reaction and afforded the product **6k** in 45% yield.

Next, we proceeded to investigate the synthesis of 2-benzoylbenzoate derivatives using different alcohols as the solvent. When the reaction was performed in MeOH, methyl ester **6r** was obtained in 43% isolated yield. The *n*-BuOH and isopropanol led to lower reaction yields of **6s** and **6t**, respectively. Only a trace amount of product was found when the reaction was performed in *t*-BuOH. These results indicated that the steric hindrance of alcohols will retard the reaction.

**Scheme 5:**
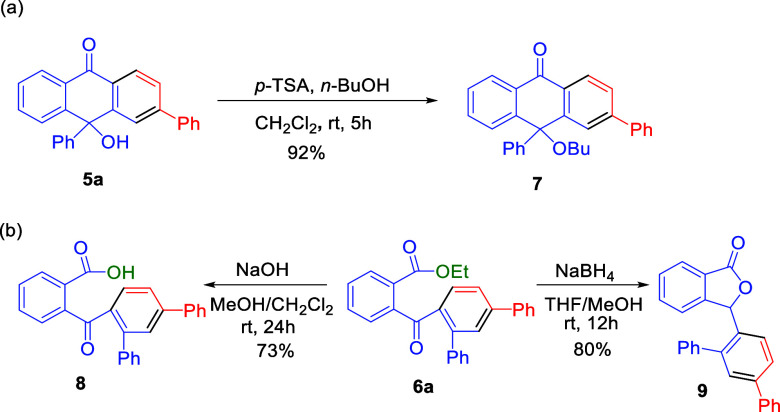
Chemical Transformations

To demonstrate the synthetic utility of these methodologies, the product **5a** could be transformed into tritylone butyl ether **7** under acidic conditions in 92% yield (Scheme 5a). The methyl ester of 2-benzoylbenzoate **6a** could be easily hydrolyzed in the basic conditions, affording the corresponding acid **8** in 73% yield (Scheme 5b). The structure of **6** was therefore confirmed using single-crystal X-ray diffraction analysis of acid **8** (CCDC 2348953).^18^ The γ-lactone product **9** was obtained in 80% yield when ester **6a** was reduced and underwent intramolecular cyclization.

**Scheme 6:**
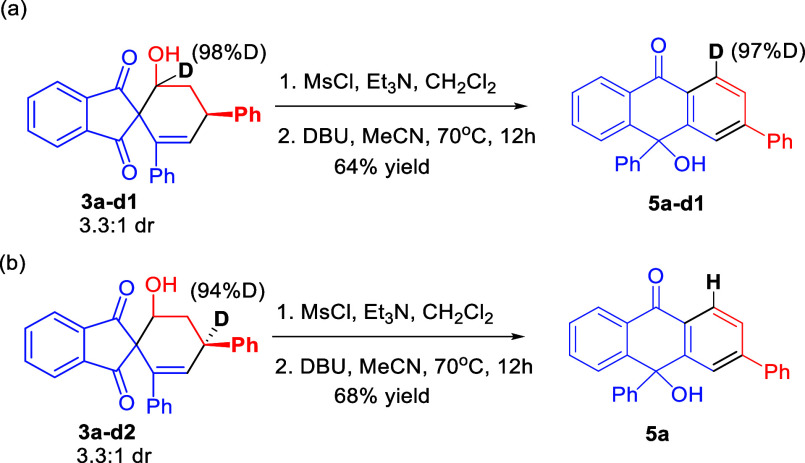
Deuterium-Labeling Experiments for Mechanistic Investigation

To further shed some light on the mechanism of the reaction, the deuterium-incorporated spiroindane-1,3-dione 3a**-**d1 (see the Supporting Information for the preparation) was applied in the deuterium-labeling study under otherwise identical conditions (Scheme 6a). The reaction afforded the deuterium-labeling product 5a-d1 in 64% yield with retention of the deuterium atom, which implies that no deprotonation occurred at this position during the aromatization.

In the other hand, the deuterium-incorporated spiroindane-1,3-dione 3a-d2 was also prepared (see the Supporting Information) and applied in the deuterium-labeling study under standard conditions (Scheme 6b). The reaction afforded product **5a** in 68% yield with no deuterium atom, which implies that the deprotonation occurred at this position during the reaction.

**Scheme 7:**
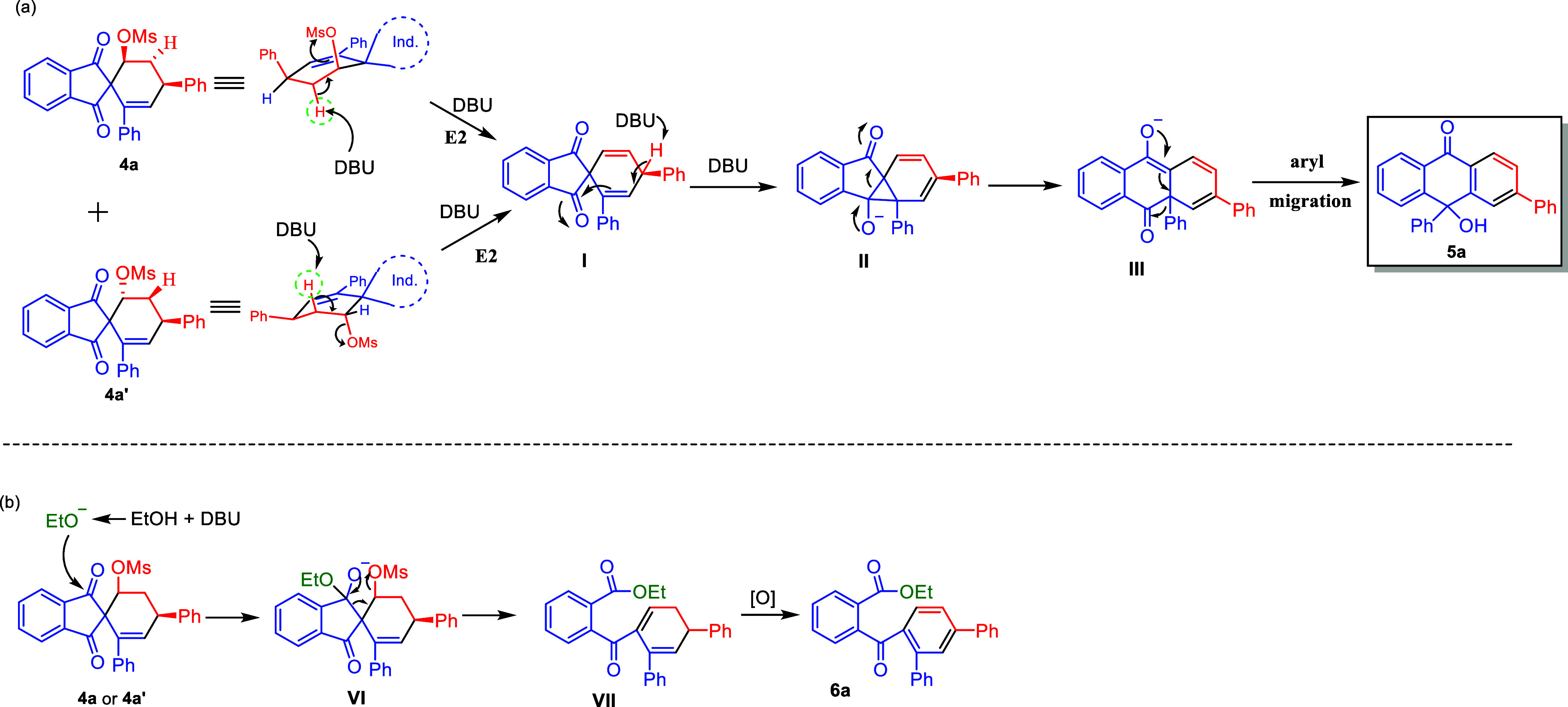
Proposed Mechanism for the Formation of Tritylone Alcohols **5** and 2-Benzoylbenzoate Esters **6**

Based on the deuterium-labeling experiments and relevant literature,^19,20^ the plausible reaction mechanisms for both reactions were proposed. As shown in Scheme 7a, the E2 elimination of diastereomer **4a** or **4a′** by DBU generates diene intermediate **I**. The following deprotonation of benzylic hydrogen by DBU and subsequent attack on the carbonyl group produced a cyclopropyl intermediate **II**. The ring-opening of the cyclopropyl ring results in intermediate **III**. Finally, compound **5a** is formed by 1,2-phenyl migration to the carbonyl group and protonation.

In alcohol solution, the generated alkoxide anion first nucleophilically attacks the carbonyl group of **4**a to form intermediate **VI** (Scheme 7b).^20^ Then, intermediate **VI** undergoes a ring expansion to obtain diene intermediate **VII**. Finally, compound **6a** is formed after the aromatization of intermediate **VII**.

In summary, we have developed the first solvent-controlled regioselective rearrangement reactions of spiroindane-1,3-diones with a leaving group. In acetonitrile solvent, the spiroindane-1,3-diones **3** were rearranged to provide tritylone alcohols, while 2-benzoylbenzoate ester derivatives were obtained if the reactions were performed in alcohols. This work not only highlights a novel protocol for synthesizing tritylone alcohols and 2-benzoylbenzoate ester derivatives but also demonstrates the synthetic potential of spiroindane-1,3-diones as synthetic precursors in the rearrangement reactions. The detailed mechanistic study and further applications of this chemistry toward other reaction designs are currently underway.

## Experimental Section

All commercially available reagents were used without further purification unless otherwise stated. All of the reaction solvents were purified before use. Proton nuclear magnetic resonance (^1^H NMR) spectra were recorded on a commercial instrument at 400 MHz. Carbon-13 nuclear magnetic resonance (^13^C{^1^H } NMR) spectra were recorded at 100 MHz. The proton signal for residual nondeuterated solvent (δ 7.26 for CHCl_3_) was used as an internal reference for ^1^H NMR spectra. For ^13^C{^1^H} NMR spectra, chemical shifts were reported relative to the δ 77.0 resonance of CHCl_3_. Coupling constants were reported in Hz. Melting points were determined on a BUCHI B-545 melting point apparatus and are uncorrected. High-resolution mass spectra were recorded on a Thermo Fisher Scientific LTQ Orbitrap XL mass spectrometer. The single crystal was measured by a Bruker D8 VENTURE X-ray single crystal diffractometer. Analytical thin-layer chromatography (TLC) was performed on silica gel 60 F254 precoated plates with visualization under UV light. Column chromatography was generally performed using 40–63 μm (230–400 mesh) silica gel, typically using a 50–100:1 weight ratio of silica gel to the crude product. Spiroindane-1,3-diones **3** were prepared according to known procedures.^17^

### General Procedure for the Synthesis of **5**

In a 7 mL glass vial, spiroindane-1,3-diones **3** (0.1 mmol) were dissolved in 1.0 mL of anhydrous DCM and cooled to 0 °C. MsCl (10.0 equiv) was added, followed by the slow addition of Et_3_N (10.0 equiv). The reaction was then stirred at room temperature for 12 h. After the reaction was completed (confirmed by TLC using DCM as eluent), the reaction was extracted by NH_4_Cl (aq.) and NaHCO_3_ (aq*.*) and dried by Na_2_SO_4_. Then the solvent was removed in vacuo, and 1.0 mL of MeCN and DBU (15 equiv) were added to the same vial and reacted at 70 °C in an oil bath for 12 h. After the reaction was completed (confirmed by TLC), the reaction was purified by column chromatography to obtain the pure products **5**.

### Synthesis of 1.0 mmol Scale of **5a**

In a 25 mL glass vial, spiroindane-1,3-diones **3a** (380 mg, 1 mmol) were dissolved in 10 mL of anhydrous DCM and cooled to 0 °C. MsCl (0.77 mL, 10 mmol) was added, followed by the slow addition of Et_3_N (1.39 mL, 10 mmol). The reaction was then stirred at room temperature for 12 h. After the reaction was completed (confirmed by TLC using DCM as eluent), the reaction was extracted by NH_4_Cl (aq*.*) and NaHCO_3_ (aq*.*) and dried by Na_2_SO_4_. Then the solvent was removed in vacuo, and 10 mL of MeCN and DBU (1.50 mL, 1.5 mmol) were added to the same vial and reacted at 70 °C in an oil bath for 12 h. After the reaction was completed (confirmed by TLC), the reaction was purified by column chromatography (hexane/toluene/ACN 20:20:1 to 10:10:1) to obtain the pure product **5a** in 60% yield over two steps (217 mg).

### General Procedure for the Synthesis of **6**

In a 7 mL glass vial, spiroindane-1,3-diones **3** (0.1 mmol) and triethylamine (1.0 mmol) were dissolved in 2.0 mL of DCM, and then methanesulfonyl chloride (1.0 mmol) was added dropwise and stirred for 12 h at room temperature. After the reaction was completed (confirmed by TLC), the solution was extracted by saturated aqueous NaHCO_3_ and saturated aqueous NH_4_Cl and water, dried over Na_2_SO_4_, and concentrated under reduced pressure. Then the solvent was removed in vacuo, and 1.0 mL of alcohols and DBU (2.5 equiv) were added to the same vial and reacted at 70 °C in an oil bath for 24 h. After the reaction was completed (confirmed by TLC), it was purified by column chromatography to obtain the pure products **6**.

### 10-Hydroxy-3,10-diphenylanthracen-9(10*H*)-one (**5a**)

Purified by silica gel column chromatography eluting with hexane/toluene/ACN 20:20:1 to 10:10:1; 67% yield over two steps (26.4 mg); white solid; mp 250–251 °C; ^1^H NMR (400 MHz, DMSO–*d*_6_): δ 8.24 (d, *J* = 8.0 Hz, 1H), 8.17 (d, *J* = 8.0 Hz, 1H), 7.87 (d, *J* = 1.2 Hz, 1H), 7.81 (dd, *J* = 8.2, 1.6 Hz, 1H), 7.66–7.62 (m, 4H), 7.52–7.46 (m, 3H), 7.41 (t, *J* = 7.3 Hz, 1H), 7.32–7.30 (m, 2H), 7.24–7.20 (m, 2H), 7.12–7.08 (m, 2H); ^13^C{^1^H} NMR (101 MHz, DMSO–*d*_6_): δ 182.9, 149.7, 149.0, 147.2, 145.2, 138.7, 134.0, 129.4, 129.2, 128.6, 128.5, 128.4, 127.9, 127.0, 126.8, 126.4, 126.2, 125.9, 125.1, 72.0; HRMS (EI) *m*/*z*: [M]^+^ calcd for C_26_H_18_O_2_: 362.1307; found, 362.1307.

### 10-Hydroxy-3-(2-nitrophenyl)-10-phenylanthracen-9(10*H*)-one (**5b**)

Purified by silica gel column chromatography eluting with hexane/toluene/ACN 20:20:1 to 10:10:1; 61% yield over two steps (25.9 mg); white solid; mp 250–251 °C; ^1^H NMR (400 MHz, CDCl_3_): δ 8.35 (d, *J* = 8.1 Hz, 1H), 8.30 (dd, *J* = 7.9, 1.3 Hz, 1H), 7.91 (dd, *J* = 8.0, 1.2 Hz, 1H), 7.67–7.65 (m, 1H), 7.63–7.56 (m, 3H), 7.53–7.45 (m, 2H), 7.41 (dd, *J* = 8.0, 1.8 Hz, 1H), 7.36–7.34 (m, 3H), 7.25–7.21 (m, 2H), 7.17–7.13 (m, 1H), 3.00 (s, 1H); ^13^C{^1^H} NMR (101 MHz, CDCl_3_): δ 183.3, 148.1, 147.6, 145.6, 143.2, 135.5, 134.2, 132.6, 131.7, 129.8, 128.9, 128.4, 128.3, 128.2, 127.9, 127.4, 126.98, 126.95, 125.4, 124.4, 73.2; HRMS (ESI) *m*/*z*: [M + Na]^+^ calcd for C_26_H_17_NO_4_Na: 430.1050; found, 430.1049.

### 10-Hydroxy-3-(2-methoxyphenyl)-10-phenylanthracen-9(10*H*)-one (**5c**)

Purified by silica gel column chromatography eluting with hexane/toluene/ACN 20:20:1 to 10:10:1; 48% yield over two steps (20.0 mg); white solid; mp 254–255 °C; ^1^H NMR (400 MHz, CDCl_3_): δ 8.32 (d, *J* = 8.2 Hz, 2H), 7.81 (d, *J* = 1.5 Hz, 1H), 7.64 (dd, *J* = 8.2, 1.4 Hz, 2H), 7.57 (td, *J* = 7.5, 1.3 Hz, 1H), 7.48–7.44 (m, 1H), 7.40–7.38 (m, 2H), 7.35–7.31 (m, 1H), 7.28–7.21 (m, 3H), 7.15 (t, *J* = 7.3 Hz, 1H), 7.01 (t, *J* = 7.4 Hz, 1H), 6.95 (d, *J* = 8.2 Hz, 1H), 3.72 (s, 3H), 2.88 (s, 1H); ^13^C{^1^H} NMR (101 MHz, CDCl_3_): δ 183.5, 156.4, 147.7, 147.3, 146.0, 144.4, 134.0, 130.6, 130.0, 129.6, 129.5, 129.4, 129.2, 128.4, 128.3, 128.2, 126.9, 126.8, 126.7, 125.5, 120.9, 111.3, 73.2, 55.4.; HRMS (ESI) *m*/*z*: [M + Na]^+^ calcd for C_27_H_20_O_3_Na: 415.1305; found, 415.1309.

### 3-(3-Bromophenyl)-10-hydroxy-10-phenylanthracen-9(10*H*)-one (**5d**)

Purified by silica gel column chromatography eluting with hexane/toluene/ACN 20:20:1 to 10:10:1; 63% yield over two steps (30.6 mg); white solid; mp 217–218 °C; ^1^H NMR (400 MHz, CDCl_3_): δ 8.30 (d, *J* = 8.2 Hz, 1H), 8.25 (dd, *J* = 7.8, 1.1 Hz, 1H), 7.81 (d, *J* = 1.8 Hz, 1H), 7.69 (t, *J* = 1.8 Hz, 1H), 7.66 (d, *J* = 7.3 Hz, 1H), 7.61–7.56 (m, 2H), 7.51–7.46 (m, 2H), 7.45–7.41 (m, 1H), 7.38–7.36 (m, 2H), 7.30 (t, *J* = 7.9 Hz, 1H), 7.25–7.21 (m, 2H), 7.15 (t, *J* = 7.3 Hz, 1H), 3.08 (s, 1H); ^13^C{^1^H} NMR (101 MHz, CDCl_3_): δ 183.4, 148.4, 147.7, 145.7, 145.0, 141.8, 134.2, 131.3, 130.4, 130.3, 129.7, 129.1, 128.5, 128.3, 128.2, 127.7, 127.0, 126.9, 126.8, 126.0, 125.3, 123.0, 73.2.; HRMS (EI) *m*/*z*: [M]^+^ calcd for C_26_H_17_O_2_ Br: 440.0412; found, 440.0405.

### 3-(4-Fluorophenyl)-10-hydroxy-10-phenylanthracen-9(10*H*)-one (**5e**)

Purified by silica gel column chromatography eluting with hexane/toluene/ACN 20:20:1 to 10:10:1; 61% yield over two steps (28.4 mg); white solid; mp 227–228 °C; ^1^H NMR (400 MHz, CDCl_3_): 8.30 (d, *J* = 8.2 Hz, 1H), 8.26 (dd, *J* = 7.8, 1.0 Hz, 1H), 7.79 (d, *J* = 1.8 Hz, 1H), 7.65 (dd, *J* = 8.0, 0.8 Hz, 1H), 7.61–7.51 (m, 4H), 7.46–7.42 (m, 1H), 7.39–7.37 (m, 2H), 7.25–7.21 (m, 2H), 7.17–7.09 (m, 3H), 3.04 (s, 1H); ^13^C{^1^H} NMR (101 MHz, CDCl_3_): δ 183.4, 163.0 (d, *J* = 247.2 Hz), 148.3, 147.7, 145.8, 145.6, 135.8, 134.1, 129.8, 129.03, 128.95, 128.6, 128.4, 128.3, 127.7, 126.9, 126.8, 126.6, 125.3, 115.9 (d, *J* = 21.6 Hz), 73.2. ^19^F NMR (376 MHz, CDCl_3_): δ −113.5.; HRMS (EI) *m*/*z*: [M]^+^ calcd for C_26_H_17_O_2_F: 380.1213; found, 380.1222.

### 3-(4-Chlorophenyl)-10-hydroxy-10-phenylanthracen-9(10*H*)-one (**5f**)

Purified by silica gel column chromatography eluting with hexane/toluene/ACN 20:20:1 to 10:10:1; 54% yield over two steps (30 mg); white solid; mp 248–249 °C; ^1^H NMR (400 MHz, CDCl_3_): δ 8.35 (d, *J* = 8.1 Hz, 1H), 8.30 (d, *J* = 7.1 Hz, 1H), 7.81 (d, *J* = 1.6 Hz, 1H), 7.67–7.62 (m, 2H), 7.61–7.55 (m, 3H), 7.48–7.42 (m, 3H), 7.38 (d, *J* = 7.4 Hz, 2H), 7.26–7.22 (m, 2H), 7.15 (t, *J* = 7.3 Hz, 1H), 2.94 (s, 1H); ^13^C{^1^H} NMR (101 MHz, CDCl_3_): δ 183.3, 148.4, 147.7, 145.7, 145.4, 138.6, 134.2, 132.0, 129.8, 128.9, 128.5, 128.3, 128.2, 127.8, 126.9, 126.8, 126.6, 125.3, 122.8, 73.2.; HRMS (EI) *m*/*z*: [M]^+^ calcd for C_26_H_17_O_2_Cl: 396.0917; found, 396.0921.

### 3-(4-Bromophenyl)-10-hydroxy-10-phenylanthracen-9(10*H*)-one (**5g**)

Purified by silica gel column chromatography eluting with hexane/toluene/ACN 20:20:1 to 10:10:1; 46% yield over two steps (27.4 mg); white solid; mp 248–249 °C; ^1^H NMR (400 MHz, CDCl_3_): δ 8.33 (d, *J* = 8.1 Hz, 1H), 8.28 (d, *J* = 7.1 Hz, 1H), 7.80 (d, *J* = 1.6 Hz, 1H), 7.65–7.61 (m, 2H), 7.59–7.55 (m, 1H), 7.49–7.43 (m, 3H), 7.38 (t, *J* = 8.0 Hz, 4H), 7.25–7.21 (m, 2H), 7.14 (t, *J* = 7.3 Hz, 1H), 2.94 (s, 1H); ^13^C{^1^H} NMR (101 MHz, CDCl_3_): δ 183.3, 148.4, 147.7, 145.7, 145.3, 138.1, 134.6, 134.2, 129.8, 129.1, 128.9, 128.6, 128.5, 128.3, 128.2, 127.8, 126.9, 126.8, 126.6, 125.3, 73.2.; HRMS (EI) *m*/*z*: [M]^+^ calcd for C_26_H_17_O_2_Br: 442.0391; found, 442.0402.

### 10-Hydroxy-3-(4-methoxyphenyl)-10-phenylanthracen-9(10*H*)-one (**5h**)

Purified by silica gel column chromatography eluting with hexane/toluene/ACN 20:20:1 to 10:10:1; 49% yield over two steps (22 mg); white solid; mp 248–249 °C; ^1^H NMR (400 MHz, CDCl_3_): δ 8.30–8.25 (m, 2H), 7.80 (d, *J* = 1.6 Hz, 1H), 7.65 (dd, *J* = 7.9, 0.8 Hz, 1H), 7.61 (dd, *J* = 8.2, 1.9 Hz, 1H), 7.56 (td, *J* = 7.5, 1.4 Hz, 1H), 7.53–7.49 (m, 2H), 7.45–7.41 (m, 1H), 7.39–7.37 (m, 2H), 7.25–7.21 (m, 2H), 7.16–7.12 (m, 1H), 6.95 (dt, *J* = 9.5, 2.5 Hz, 2H), 3.84 (s, 3H), 3.03 (s, 1H); ^13^C{^1^H} NMR (101 MHz, CDCl_3_): δ 183.4, 160.0, 148.3, 147.8, 146.2, 145.9, 134.0, 132.0, 129.9, 128.44, 128.40, 128.3, 128.2, 128.1, 127.6, 126.8, 126.5, 126.1, 125.3, 114.3, 73.2, 55.4.; HRMS (ESI) *m*/*z*: [M – H]^−^ calcd for C_27_H_19_O_3_: 391.1340; found, 391.1342.

### 10-Hydroxy-3-(4-nitrophenyl)-10-phenylanthracen-9(10*H*)-one (**5i**)

Purified by silica gel column chromatography eluting with hexane/toluene/ACN 20:20:1 to 10:10:1; 44% yield over two steps (10 mg); white solid; mp 247–248 °C; ^1^H NMR (400 MHz, CDCl_3_): δ 8.38 (d, *J* = 8.1 Hz, 1H), 8.29 (d, *J* = 8.8 Hz, 3H), 7.89 (d, *J* = 1.5 Hz, 1H), 7.72–7.66 (m, 4H), 7.60 (t, *J* = 7.4 Hz, 1H), 7.47 (t, *J* = 7.5 Hz, 1H), 7.38 (d, *J* = 7.6 Hz, 2H), 7.25 (t, *J* = 7.6 Hz, 2H), 7.16 (t, *J* = 7.2 Hz, 1H), 3.05 (s, 1H); ^13^C{^1^H} NMR (101 MHz, CDCl_3_): δ 183.2, 148.6, 147.7, 147.6, 146.0, 145.5, 144.0, 134.4, 129.8, 129.7, 128.6, 128.4, 128.24, 128.16, 127.9, 127.4, 127.2, 127.1, 127.0, 125.3, 124.1, 73.2.; HRMS (ESI) *m*/*z*: [M – H]^−^ calcd for C_26_H_16_NO_4_: 406.1085; found, 406.1086.

### 10-Hydroxy-3-(naphthalen-2-yl)-10-phenylanthracen-9(10*H*)-one (**5j**)

Purified by silica gel column chromatography eluting with hexane/toluene/ACN 20:20:1 to 10:10:1; 71% yield over two steps (32.3 mg); white solid; mp 257–258 °C; ^1^H NMR (400 MHz, CDCl_3_): δ 8.37 (d, *J* = 8.2 Hz, 1H), 8.29 (dd, *J* = 7.8, 1.2 Hz, 1H), 8.03 (d, *J* = 1.2 Hz, 1H), 7.98 (d, *J* = 1.6 Hz, 1H), 7.91–7.84 (m, 3H), 7.79 (dd, *J* = 8.2, 1.9 Hz, 1H), 7.68 (dd, *J* = 8.5, 1.8 Hz, 2H), 7.59 (td, *J* = 7.5, 1.4 Hz, 1H), 7.53–7.48 (m, 2H), 7.47–7.40 (m, 3H), 7.24 (t, *J* = 7.7 Hz, 2H), 7.16–7.13 (m, 1H), 3.06 (s, 1H); ^13^C{^1^H} NMR (101 MHz, CDCl_3_): δ 183.5, 148.3, 147.8, 146.6, 145.9, 137.0, 134.1, 133.4, 133.0, 129.9, 129.0, 128.70, 128.68, 128.5, 128.4, 128.3, 128.2, 127.7, 127.7, 127.6, 127.3, 127.0, 126.9, 126.6, 126.5, 125.4, 125.1, 73.3.; HRMS (ESI) *m*/*z*: [M – H]^−^ calcd for C_30_H_19_O_2_: 411.1391; found, 411.1397.

### 10-Hydroxy-10-phenyl-3-(thiophen-2-yl)anthracen-9(10*H*)-one (**5k**)

Purified by filtration and washed by hexane after the reaction was completed; 34% yield over two steps (17 mg); white solid; mp 238–239 °C; ^1^H NMR (400 MHz, DMSO–*d*_6_): δ 8.18 (d, *J* = 8.9 Hz, 1H), 8.14 (d, *J* = 7.7 Hz, 1H), 7.84–7.81 (m, 2H), 7.66–7.62 (m, 4H), 7.51–7.47 (m, 1H), 7.30–7.28 (m, 2H), 7.22 (t, *J* = 7.7 Hz, 2H), 7.16 (dd, *J* = 4.9, 3.7 Hz, 1H), 7.12–7.09 (m, 2H); ^13^C{^1^H} NMR (101 MHz, DMSO–*d*_6_): δ 182.7, 150.3, 149.2, 147.3, 142.0, 138.9, 134.2, 129.5, 129.2, 128.6, 128.4, 128.1, 127.5, 126.7, 126.1, 126.0, 125.2, 124.9, 124.7, 72.1.; HRMS (EI) *m*/*z*: [M]^+^ calcd for C_24_H_16_O_2_S: 368.0871; found, 368.0868.

### 10-(4-Fluorophenyl)-10-hydroxy-3-phenylanthracen-9(10*H*)-one (**5L**)

Purified by silica gel column chromatography eluting with hexane/toluene/ACN 20:20:1 to 10:10:1; 45% yield over two steps (20 mg); white solid; mp 248–249 °C; ^1^H NMR (400 MHz, CDCl_3_): δ 8.32 (d, *J* = 8.2 Hz, 1H), 8.27 (d, *J* = 7.8 Hz, 1H), 7.82 (d, *J* = 1.6 Hz, 1H), 7.68–7.56 (m, 5H), 7.47–7.43 (m, 3H), 7.40–7.34 (m, 3H), 6.94–6.89 (m, 2H), 3.02 (s, 1H); ^13^C{^1^H} NMR (101 MHz, CDCl_3_): δ 183.3, 148.0, 147.5, 146.8, 141.7, 139.6, 134.2, 129.8, 128.9, 128.6, 128.5, 128.4, 128.2, 127.7, 127.3, 127.2, 127.1 (d, *J* = 3.9 Hz), 127.0, 126.7, 115.3 (d, *J* = 21.1 Hz), 72.9. ^19^F NMR (376 MHz, CDCl_3_): δ −115.6.; HRMS (ESI) *m*/*z*: [M – H]^−^ calcd for C_26_H_16_O_2_F: 379.1140; found, 379.1149.

### 10-(4-Chlorophenyl)-10-hydroxy-3-phenylanthracen-9(10*H*)-one (**5m**)

Purified by silica gel column chromatography eluting with hexane/toluene/ACN 20:20:1 to 10:10:1; 46% yield over two steps (24.5 mg); white solid; mp 251–252 °C; ^1^H NMR (400 MHz, CDCl_3_): δ 8.30 (d, *J* = 8.2 Hz, 1H), 8.25 (d, *J* = 7.7 Hz, 1H), 7.79 (s, 1H), 7.65 (d, *J* = 7.4 Hz, 1H), 7.61–7.54 (m, 4H), 7.45–7.36 (m, 4H), 7.31 (d, *J* = 8.4 Hz, 2H), 7.19 (d, *J* = 8.2 Hz, 2H), 3.05 (s, 1H); ^13^C{^1^H} NMR (101 MHz, CDCl_3_): δ 183.3, 147.8, 147.3, 146.8, 144.5, 139.5, 134.2, 132.8, 129.7, 128.9, 128.6, 128.5, 128.4, 128.3, 127.7, 127.3, 127.2, 127.0, 126.9, 126.7, 72.9.; HRMS (ESI) *m*/*z*: [M – H]^−^ calcd for C_26_H_16_O_2_Cl: 395.0844; found, 395.0851.

### 10-(4-Bromophenyl)-10-hydroxy-3-phenylanthracen-9(10*H*)-one (**5n**)

Purified by silica gel column chromatography eluting with hexane/toluene/ACN 20:20:1 to 10:10:1; 47% yield over two steps (27 mg); white solid; mp 247–248 °C; ^1^H NMR (400 MHz, CDCl_3_): δ 8.29 (d, *J* = 8.1 Hz, 1H), 8.24 (d, *J* = 8.0 Hz, 1H), 7.80 (d, *J* = 1.6 Hz, 1H), 7.64 (dd, *J* = 8.2, 1.7 Hz, 1H), 7.62–7.55 (m, 4H), 7.47–7.42 (m, 3H), 7.41–7.39 (m, 1H), 7.37–7.33 (m, 2H), 7.28–7.25 (m, 3H), 3.10 (s, 1H); ^13^C{^1^H} NMR (101 MHz, CDCl_3_): δ 183.3, 147.7, 147.2, 146.8, 145.0, 139.5, 134.2, 131.5, 129.7, 129.0, 128.5, 128.4, 128.3, 127.7, 127.3, 127.2, 127.0, 126.7, 121.0, 72.9.; HRMS (ESI) *m*/*z*: [M – H]^−^ calcd for C_26_H_16_O_2_Br: 439.0339; found, 439.0341.

### 10-Hydroxy-10-(4-methoxyphenyl)-3-phenylanthracen-9(10*H*)-one (**5o**)

Purified by silica gel column chromatography eluting with hexane/toluene/ACN 20:20:1 to 10:10:1; 42% yield over two steps (18 mg); white solid; mp 234–235 °C; ^1^H NMR (400 MHz, CDCl_3_): δ 8.32 (d, *J* = 8.2 Hz, 1H), 8.27 (dd, *J* = 7.8, 1.2 Hz, 1H), 7.86 (d, *J* = 1.8 Hz, 1H), 7.68–7.65 (m, 2H), 7.60–7.56 (m, 3H), 7.46–7.42 (m, 3H), 7.40–7.36 (m, 1H), 7.30–7.27 (m, 2H), 6.75 (dt, *J* = 9.6, 2.6 Hz, 2H), 3.72 (s, 3H), 2.94 (s, 1H); ^13^C{^1^H} NMR (101 MHz, CDCl_3_): δ 183.5, 158.3, 148.5, 148.0, 146.6, 139.7, 138.2, 134.1, 129.8, 128.9, 128.6, 128.4, 128.2, 127.6, 127.3, 126.9, 126.9, 126.7, 126.6, 113.8, 73.1, 55.2.; HRMS (EI) *m*/*z*: [M]^+^ calcd for C_27_H_20_O_3_: 392.1412; found, 392.1405.

### 10-Hydroxy-10-(naphthalen-2-yl)-3-phenylanthracen-9(10*H*)-one (**5p**)

Purified by silica gel column chromatography eluting with hexane/toluene/ACN 20:20:1 to 10:10:1; 62% yield over two steps (27.1 mg); white solid; mp 230–231 °C; ^1^H NMR (400 MHz, CDCl_3_): δ 8.33 (d, *J* = 8.2 Hz, 1H), 8.27 (d, *J* = 7.7 Hz, 2H), 7.87 (t, *J* = 8.2 Hz, 2H), 7.71 (d, *J* = 7.8 Hz, 1H), 7.65 (t, *J* = 6.9 Hz, 2H), 7.59 (d, *J* = 8.7 Hz, 1H), 7.56–7.34 (m, 9H), 7.07 (d, *J* = 8.7 Hz, 1H), 3.25 (s, 1H); ^13^C{^1^H} NMR (101 MHz, CDCl_3_): δ 183.5, 147.9, 147.4, 146.7, 143.0, 139.6, 134.1, 133.1, 132.2, 129.9, 128.9, 128.7, 128.33, 128.26, 127.7, 127.5, 127.3, 127.1, 126.9, 126.3, 126.1, 124.3, 123.2, 73.3.; HRMS (EI) *m*/*z*: [M]^+^ calcd for C_30_H_20_O_2_: 412.1463; found, 412.1460.

### 10-Hydroxy-3-phenyl-10-(thiophen-2-yl)anthracen-9(10*H*)-one (**5q**)

Purified by silica gel column chromatography eluting with hexane/toluene/ACN 20:20:1 to 10:10:1; 48% yield over two steps (16.4 mg); white solid; mp 222–223 °C; ^1^H NMR (400 MHz, CDCl_3_): δ 8.31 (d, *J* = 8.1 Hz, 1H), 8.26 (d, *J* = 7.8 Hz, 1H), 8.16 (d, *J* = 1.6 Hz, 1H), 7.95 (d, *J* = 7.8 Hz, 1H), 7.71 (dd, *J* = 8.2, 1.7 Hz, 1H), 7.68–7.64 (m, 3H), 7.52–7.46 (m, 3H), 7.41 (t, *J* = 7.3 Hz, 1H), 7.15 (dd, *J* = 5.1, 1.1 Hz, 1H), 6.75 (dd, *J* = 5.1, 3.7 Hz, 1H), 6.47 (dd, *J* = 3.6, 1.2 Hz, 1H), 3.15 (s, 1H); ^13^C{^1^H} NMR (101 MHz, CDCl_3_): δ 183.2, 151.6, 147.4, 146.9, 146.5, 139.7, 133.9, 129.8, 129.0, 128.6, 128.5, 127.9, 127.4, 127.3, 127.1, 126.7, 125.8, 125.1, 124.7, 72.5.; HRMS (EI) *m*/*z*: [M]^+^ calcd for C_24_H_16_O_2_S: 368.0871; found, 368.0866.

### Ethyl 2-([1,1′:3′,1″-Rerphenyl]-4′-carbonyl)benzoate (**6a**)

Purified by silica gel column chromatography eluting with hexane/toluene 100:0 to 0:100; 68% yield over two steps (27.6 mg); colorless oil; ^1^H NMR (400 MHz, CDCl_3_): δ 7.75 (d, *J* = 8.1 Hz, 1H), 7.67–7.64 (m, 3H), 7.62 (d, *J* = 1.5 Hz, 1H), 7.60 (d, *J* = 7.3 Hz, 1H), 7.47 (t, *J* = 7.4 Hz, 2H), 7.41–7.34 (m, 4H), 7.32–7.30 (m, 2H), 7.25–7.15 (m, 3H), 4.21 (q, *J* = 7.1 Hz, 2H), 1.25 (t, *J* = 7.1 Hz, 3H); ^13^C{^1^H} NMR (101 MHz, CDCl_3_): δ 197.2, 167.7, 144.1, 143.1, 140.5, 140.0, 139.7, 136.0, 132.0, 131.7, 130.5, 129.7, 129.1, 129.0, 128.9, 128.2, 128.0, 127.2, 125.6, 61.5, 13.9; HRMS (ESI) *m*/*z*: [M + Na]^+^ calcd for C_28_H_22_O_3_Na: 429.1461; found, 429.1456.

### Ethyl 2-(2-Methoxy-[1,1′:3′,1″-terphenyl]-4′-carbonyl) Benzoate (**6b**)

Purified by silica gel column chromatography eluting with hexane/toluene 100:0 to 0:100; 57% yield over two steps (34.9 mg); colorless oil; ^1^H NMR (400 MHz, CDCl_3_): δ 7.71 (d, *J* = 7.9 Hz, 1H), 7.63 (dd, *J* = 8.0, 1.8 Hz, 1H), 7.61–7.58 (m, 2H), 7.40–7.34 (m, 6H), 7.32–7.28 (m, 1H), 7.24–7.20 (m, 2H), 7.18–7.14 (m, 1H), 7.05 (td, *J* = 7.5, 1.3 Hz, 1H), 7.02 (d, *J* = 8.3 Hz, 1H), 4.23 (q, *J* = 7.1 Hz, 2H), 3.84 (s, 3H), 1.28 (t, *J* = 7.2 Hz, 3H); ^13^C{^1^H} NMR (101 MHz, CDCl_3_): δ 197.2, 167.8, 156.5, 142.3, 141.9, 140.6, 140.0, 135.6, 132.2, 132.0, 130.8, 130.5, 130.4, 129.8, 129.4, 129.3, 128.9, 128.3, 127.9, 127.1, 120.9, 111.3, 61.5, 55.5, 13.9; HRMS (ESI) *m*/*z*: [M + Na]^+^ calcd for C_29_H_24_O_4_Na: 459.1567; found, 459.1562.

### Ethyl 2-(3-Bromo-[1,1′:3′,1″-terphenyl]-4′-carbonyl) Benzoate (**6c**)

Purified by silica gel column chromatography eluting with hexane/toluene 100:0 to 0:100; 43% yield over two steps (23.3 mg); colorless oil; ^1^H NMR (400 MHz, CDCl_3_): δ 7.80 (t, *J* = 1.8 Hz, 1H), 7.76 (d, *J* = 8.1 Hz, 1H), 7.63–7.57 (m, 4H), 7.53 (dq, *J* = 8.1, 1.0 Hz, 1H), 7.39–7.34 (m, 4H), 7.32–7.29 (m, 2H), 7.25–7.16 (m, 3H), 4.21 (q, *J* = 7.2 Hz, 2H), 1.27 (t, *J* = 7.2 Hz, 3H); ^13^C{^1^H} NMR (101 MHz, CDCl_3_): δ 197.0, 167.6, 143.3, 142.5, 141.9, 140.2, 139.9, 136.6, 132.0, 131.7, 131.1, 130.6, 130.5, 130.4, 130.3, 129.7, 129.1, 129.0, 128.0, 127.4, 125.9, 125.6, 123.1, 61.5, 13.9; HRMS (ESI) *m*/*z*: [M + Na]^+^ calcd for C_28_H_21_O_3_NaBr: 507.0566; found, 507.0576.

### Ethyl 2-(4-Fluoro-[1,1′:3′,1″-terphenyl]-4′-carbonyl) Benzoate (**6d**)

Purified by silica gel column chromatography eluting with hexane/toluene 100:0 to 0:100; 37% yield over two steps (28.5 mg); colorless oil; ^1^H NMR (400 MHz, CDCl_3_): δ 7.75 (d, *J* = 8.1 Hz, 1H), 7.64–7.59 (m, 4H), 7.57 (d, *J* = 1.8 Hz, 1H), 7.39–7.33 (m, 3H), 7.31 (dd, *J* = 4.0, 1.2 Hz, 2H), 7.25–7.14 (m, 5H), 4.21 (q, *J* = 7.1 Hz, 2H), 1.26 (t, *J* = 7.1 Hz, 3H); ^13^C{^1^H} NMR (101 MHz, CDCl_3_): δ 197.1, 167.6, 162.9 (d, *J* = 248.8 Hz), 143.3, 143.1, 140.4, 140.0, 136.0, 135.9, 132.0, 131.8, 130.5, 129.6, 129.5, 129.1, 129.0, 128.95, 128.87, 128.0, 127.3, 125.4, 115.9 (d, *J* = 21.8 Hz), 61.5, 13.9; ^19^F NMR (376 MHz, CDCl_3_): δ −114.0; HRMS (ESI) *m*/*z*: [M + Na]^+^ calcd for C_28_H_21_O_3_FNa: 447.1367; found, 447.1366.

### Ethyl 2-(4-Chloro-[1,1′:3′,1″-terphenyl]-4′-carbonyl) Benzoate (**6e**)

Purified by silica gel column chromatography eluting with hexane/toluene 100:0 to 0:100; 39% yield over two steps (28.5 mg); white solid; mp 133–134 °C; ^1^H NMR (400 MHz, CDCl_3_): δ 7.75 (d, *J* = 8.1 Hz, 1H), 7.63–7.60 (m, 3H), 7.58 (s, 2H), 7.44 (dt, *J* = 9.6, 2.3 Hz, 2H), 7.39–7.34 (m, 3H), 7.31–7.30 (m, 2H), 7.25–7.18 (m, 3H), 4.21 (q, *J* = 7.1 Hz, 2H), 1.26 (t, *J* = 7.2 Hz, 3H); ^13^C{^1^H} NMR (101 MHz, CDCl_3_): δ 197.0, 167.6, 143.3, 142.8, 140.3, 140.0, 138.2, 136.3, 134.4, 132.0, 131.8, 130.5, 129.6, 129.5, 129.1, 129.0, 128.5, 128.0, 127.3, 125.4, 61.5, 13.9; HRMS (ESI) *m*/*z*: [M + Na]^+^ calcd for C_28_H_21_O_3_NaCl: 463.1071; found, 463.1071.

### Ethyl 2-(4-Bromo-[1,1′:3′,1″-terphenyl]-4′-carbonyl) Benzoate (**6f**)

Purified by silica gel column chromatography eluting with hexane/toluene 100:0 to 0:100; 42% yield over two steps (21.2 mg); white solid; mp 138–139 °C; ^1^H NMR (400 MHz, CDCl_3_): δ 7.75 (d, *J* = 8.1 Hz, 1H), 7.62–7.57 (m, 5H), 7.52 (dt, *J* = 8.7, 2.0 Hz, 2H), 7.38–7.33 (m, 3H), 7.31–7.28 (m, 2H), 7.25–7.16 (m, 3H), 4.21 (q, *J* = 7.1 Hz, 2H), 1.26 (t, *J* = 7.1 Hz, 3H); ^13^C{^1^H} NMR (101 MHz, CDCl_3_): δ 197.0, 167.6, 143.3, 142.9, 140.3, 140.0, 138.7, 136.4, 132.1, 132.0, 131.8, 130.5, 129.6, 129.4, 129.1, 129.0, 128.8, 128.0, 127.3, 125.4, 122.6, 61.5, 13.9. HRMS (ESI) *m*/*z*: [M + Na]^+^ calcd for C_28_H_21_O_3_NaBr: 507.0566; found, 507.0571.

### Ethyl 2-(4-Methoxy-[1,1′:3′,1″-terphenyl]-4′-carbonyl) Benzoate (**6g**)

Purified by silica gel column chromatography eluting with hexane/toluene 100:0 to 0:100; 42% yield over two steps (32.7 mg); white solid; mp 104–105 °C; ^1^H NMR (400 MHz, CDCl_3_): δ 7.73 (d, *J* = 7.9 Hz, 1H), 7.62–7.58 (m, 5H), 7.38–7.30 (m, 5H), 7.25–7.16 (m, 3H), 7.00 (dt, *J* = 9.5, 2.6 Hz, 2H), 4.21 (q, *J* = 7.2 Hz, 2H), 3.86 (s, 3H), 1.26 (t, *J* = 7.1 Hz, 3H); ^13^C{^1^H} NMR (101 MHz, CDCl_3_): δ 197.1, 167.6, 159.9, 143.8, 143.2, 140.7, 140.2, 135.4, 132.1, 132.0, 131.8, 130.5, 130.4, 129.6, 129.2, 129.0, 128.3, 127.9, 127.2, 125.0, 114.4, 61.5, 55.3, 13.9; HRMS (ESI) *m*/*z*: [M + Na]^+^ calcd for C_29_H_24_O_4_Na: 459.1567; found, 459.1563.

### Ethyl 2-(4-Methyl-[1,1′:3′,1″-terphenyl]-4′-carbonyl) Benzoate (**6h**)

Purified by silica gel column chromatography eluting with hexane/toluene 100:0 to 0:100; 38% yield over two steps (30.9 mg); white solid; mp 81–82 °C; ^1^H NMR (400 MHz, CDCl_3_): δ 7.74 (d, *J* = 8.1 Hz, 1H), 7.64 (dd, *J* = 7.9, 1.8 Hz, 1H), 7.62–7.56 (m, 4H), 7.38–7.34 (m, 3H), 7.33–7.27 (m, 4H), 7.25–7.16 (m, 3H), 4.22 (q, *J* = 7.1 Hz, 2H), 2.42 (s, 3H), 1.26 (t, *J* = 7.1 Hz, 3H); ^13^C{^1^H} NMR (101 MHz, CDCl_3_): δ 197.1, 167.7, 144.1, 143.2, 140.6, 140.1, 138.1, 136.8, 135.7, 132.0, 131.7, 130.5, 130.4, 129.7, 129.4, 129.2, 129.0, 127.9, 127.2, 127.1, 125.3, 61.5, 21.1, 13.9; HRMS (ESI) *m*/*z*: [M + Na]^+^ calcd for C_29_H_24_O_3_Na: 443.1618; found, 443.1614.

### Ethyl 2-[5-(Naphthalen-2-yl)-[1,1′-biphenyl]-2-carbonyl] Benzoate (**6i**)

Purified by silica gel column chromatography eluting with hexane/toluene 100:0 to 0:100; 46% yield over two steps (25.0 mg); colorless oil; ^1^H NMR (400 MHz, CDCl_3_): δ 8.13 (s, 1H), 7.95–7.86 (m, 3H), 7.80 (dd, *J* = 6.5, 1.8 Hz, 3H), 7.76 (d, *J* = 0.8 Hz, 1H), 7.61 (d, *J* = 7.3 Hz, 1H), 7.54–7.49 (m, 2H), 7.41–7.29 (m, 5H), 7.26–7.17 (m, 3H), 4.23 (q, *J* = 7.1 Hz, 2H), 1.27 (t, *J* = 7.1 Hz, 3H); ^13^C{^1^H} NMR (101 MHz, CDCl_3_): δ 197.1, 167.7, 144.0, 143.3, 140.5, 140.1, 137.0, 136.1, 133.5, 133.0, 132.0, 131.8, 130.5, 129.9, 129.7, 129.2, 129.0, 128.7, 128.3, 128.0, 127.7, 127.3, 126.5, 126.4, 126.3, 125.8, 125.2, 61.5, 13.9; HRMS (ESI) *m*/*z*: [M + Na]^+^ calcd for C_32_H_24_O_3_Na: 479.1618; found, 479.1625.

### Ethyl 2-[5-(Thiophen-2-yl)-[1,1′-biphenyl]-2-carbonyl] Benzoate (**6j**)

Purified by silica gel column chromatography eluting with hexane/toluene 100:0 to 0:100; 48% yield over two steps (20.6 mg); colorless oil; ^1^H NMR (400 MHz, CDCl_3_): δ 7.70–7.60 (m, 4H), 7.44 (dd, *J* = 3.6, 1.1 Hz, 1H), 7.38–7.33 (m, 4H), 7.31–7.17 (m, 5H), 7.12 (dd, *J* = 5.0, 3.7 Hz, 1H), 4.21 (q, *J* = 7.1 Hz, 2H), 1.26 (t, *J* = 7.2 Hz, 3H); ^13^C{^1^H} NMR (101 MHz, CDCl_3_): δ 196.8, 143.4, 142.9, 140.3, 140.2, 137.3, 136.0, 131.9, 131.9, 130.6, 130.4, 129.6, 129.1, 129.0, 128.3, 128.1, 128.0, 127.3, 126.3, 124.5, 124.1, 61.5, 13.9; HRMS (ESI) *m*/*z*: [M + Na]^+^ calcd for C_26_H_20_O_3_NaS: 435.1025; found, 435.1030.

### Ethyl 2-(5-Ethyl-[1,1′-biphenyl]-2-carbonyl)benzoate (**6k**)

Purified by silica gel column chromatography eluting with hexane/toluene 100:0 to 0:100; 45% yield over two steps (18.3 mg); colorless oil; ^1^H NMR (400 MHz, CDCl_3_): δ 7.59–7.56 (m, 2H), 7.35–7.23 (m, 6H), 7.21–7.12 (m, 4H), 4.18 (q, *J* = 7.1 Hz, 2H), 2.73 (q, *J* = 7.6 Hz, 2H), 1.28 (t, *J* = 7.6 Hz, 3H), 1.23 (t, *J* = 7.1 Hz, 3H); ^13^C{^1^H} NMR (101 MHz, CDCl_3_): δ 197.2, 167.7, 148.2, 142.8, 140.7, 140.2, 134.9, 132.0, 131.3, 130.5, 130.4, 130.3, 129.7, 129.1, 128.9, 127.9, 127.0, 126.6, 61.4, 28.8, 15.2, 13.9; HRMS (ESI) *m*/*z*: [M + Na]^+^ calcd for C_24_H_22_O_3_Na: 381.1461; found, 381.1460.

### Ethyl 2-(4″-Fluoro-[1,1′:3′,1″-terphenyl]-4′-carbonyl) Benzoate (**6L**)

Purified by silica gel column chromatography eluting with hexane/toluene 100:0 to 0:100; 43% yield over two steps (27.7 mg); yellow oil; ^1^H NMR (400 MHz, CDCl_3_): δ 7.73 (d, *J* = 8.1 Hz, 1H), 7.67–7.64 (m, 4H), 7.59 (d, *J* = 1.6 Hz, 1H), 7.48 (d, *J* = 7.2 Hz, 2H), 7.43–7.31 (m, 6H), 6.93 (t, *J* = 8.6 Hz, 2H), 4.21 (q, *J* = 7.1 Hz, 2H), 1.26 (t, *J* = 7.1 Hz, 3H); ^13^C{^1^H} NMR (101 MHz, CDCl_3_): δ 197.1, 167.5, 162.2 (d, *J* = 252.5 Hz), 144.3, 142.1, 140.1, 139.6, 136.6, 135.9, 131.9, 131.8, 130.84, 130.75, 130.7, 130.6, 129.7, 129.5, 129.1, 129.0, 128.2, 127.2, 125.7, 114.9 (d, *J* = 20 Hz), 61.5, 13.9; ^19^F NMR (376 MHz, CDCl_3_): δ −115.1; HRMS (ESI) *m*/*z*: [M + Na]^+^ calcd for C_28_H_21_FO_3_Na: 447.1367; found, 447.1377.

### Ethyl 2-(4″-Chloro-[1,1′:3′,1″-terphenyl]-4′-carbonyl) Benzoate (**6m**)

Purified by silica gel column chromatography eluting with hexane/toluene 100:0 to 0:100; 41% yield over two steps (29.8 mg); colorless oil; ^1^H NMR (400 MHz, CDCl_3_): δ 7.71–7.63 (m, 5H), 7.58 (d, *J* = 1.5 Hz, 1H), 7.50–7.36 (m, 5H), 7.35–7.32 (m, 3H), 7.22 (dt, *J* = 8.8, 2.2 Hz, 2H), 4.20 (q, *J* = 7.2 Hz, 2H), 1.25 (t, *J* = 7.2 Hz, 3H); ^13^C{^1^H} NMR (101 MHz, CDCl_3_): δ 196.9, 167.3, 144.4, 142.0, 140.3, 139.6, 139.1, 135.8, 133.3, 131.8, 130.8, 130.6, 130.4, 129.7, 129.5, 129.2, 129.0, 128.3, 128.1, 127.2, 125.9, 61.5, 13.9; HRMS (ESI) *m*/*z*: [M + Na]^+^ calcd for C_28_H_21_O_3_NaCl: 463.1071; found, 463.1075.

### Ethyl 2-(4″-Bromo-[1,1′:3′,1″-terphenyl]-4′-carbonyl) Benzoate

Purified by silica gel column chromatography eluting with hexane/toluene 100:0 to 0:100; 45% yield over two steps (38.0 mg); colorless oil; ^1^H NMR (400 MHz, CDCl_3_): δ 7.69–7.67 (m, 2H), 7.63 (dd, *J* = 7.4, 2.7 Hz, 3H), 7.56 (d, *J* = 1.5 Hz, 1H), 7.48–7.35 (m, 7H), 7.32 (d, *J* = 7.3 Hz, 1H), 7.28 (s, 1H), 7.25 (d, *J* = 2.8 Hz, 1H), 4.18 (q, *J* = 7.1 Hz, 2H), 1.23 (t, *J* = 7.1 Hz, 3H); ^13^C{^1^H} NMR (101 MHz, CDCl_3_): δ 196.9, 167.3, 144.4, 142.0, 140.3, 139.6, 139.5, 135.8, 131.8, 131.1, 130.9, 130.7, 130.6, 129.6, 129.5, 129.2, 129.0, 128.3, 127.2, 125.9, 121.6, 61.5, 13.9; HRMS (ESI) *m*/*z*: [M + Na]^+^ calcd for C_28_H_21_O_3_NaBr: 507.0566; found, 507.0576.

### Ethyl 2-(4″-Methoxy-[1,1′:3′,1″-terphenyl]-4′-carbonyl) Benzoate (**6o**)

Purified by silica gel column chromatography eluting with hexane/toluene 100:0 to 0:100; 39% yield over two steps (25.5 mg); colorless oil; ^1^H NMR (400 MHz, CDCl_3_): δ 7.73 (dd, *J* = 8.7, 1.6 Hz, 1H), 7.68–7.60 (m, 5H), 7.49–7.45 (m, 2H), 7.42–7.29 (m, 6H), 6.77 (dt, *J* = 8.7, 2.0 Hz, 2H), 4.21 (q, *J* = 7.2 Hz, 2H), 3.76 (s, 3H), 1.26 (t, *J* = 7.1 Hz, 3H); ^13^C{^1^H} NMR (101 MHz, CDCl_3_): δ 197.3, 167.8, 158.9, 144.1, 142.8, 140.0, 139.8, 136.0, 132.9, 132.1, 131.7, 130.51, 130.46, 130.4, 129.7, 129.6, 129.0, 128.9, 128.1, 127.2, 125.3, 113.5, 61.5, 55.2, 13.9; HRMS (ESI) *m*/*z*: [M + Na]^+^ calcd for C_29_H_24_O_4_Na: 459.1567; found, 459.1566.

### Ethyl 2-[3-(Naphthalen-2-yl)-[1,1′-biphenyl]-4-carbonyl] Benzoate (**6p**)

Purified by silica gel column chromatography eluting with hexane/toluene 100:0 to 0:100; 44% yield over two steps (28.3 mg); colorless oil; ^1^H NMR (400 MHz, CDCl_3_): δ 7.82 (d, *J* = 7.9 Hz, 2H), 7.80–7.73 (m, 3H), 7.69 (dd, *J* = 8.0, 1.9 Hz, 4H), 7.55–7.39 (m, 7H), 7.34–7.32 (m, 1H), 7.22–7.20 (m, 2H), 4.23 (q, *J* = 7.1 Hz, 2H), 1.28 (t, *J* = 7.1 Hz, 3H); ^13^C{^1^H} NMR (101 MHz, CDCl_3_): δ 197.1, 167.4, 144.3, 143.2, 140.3, 139.8, 138.1, 136.1, 133.0, 132.3, 131.9, 131.7, 130.5, 130.2, 130.0, 129.4, 129.0, 128.3, 128.2, 128.1, 127.5, 127.3, 126.0, 125.9, 125.7, 77.3, 61.5, 14.0; HRMS (ESI) *m*/*z*: [M + Na]^+^ calcd for C_32_H_24_O_3_Na: 479.1618; found, 479.1613.

### Ethyl 2-[3-(Thiophen-2-yl)-[1,1′-biphenyl]-4-carbonyl] Benzoate (**6q**)

Purified by silica gel column chromatography eluting with hexane/toluene 100:0 to 0:100; 37% yield over two steps (19.9 mg); colorless oil; ^1^H NMR (400 MHz, CDCl_3_): δ 7.74 (d, *J* = 1.5 Hz, 1H), 7.71–7.62 (m, 5H), 7.48 (t, *J* = 7.4 Hz, 2H), 7.45–7.39 (m, 2H), 7.35 (d, *J* = 4.0 Hz, 2H), 7.19 (dd, *J* = 5.0, 1.1 Hz, 1H), 7.12 (dd, *J* = 3.6, 1.1 Hz, 1H), 6.86 (dd, *J* = 5.1, 3.6 Hz, 1H), 4.27 (q, *J* = 7.1 Hz, 2H), 1.29 (t, *J* = 7.2 Hz, 3H); ^13^C{^1^H} NMR (101 MHz, CDCl_3_): δ 196.9, 168.0, 144.1, 141.2, 139.5, 139.2, 136.6, 135.0, 132.5, 131.4, 130.9, 130.4, 129.8, 129.7, 129.0, 128.7, 128.2, 127.4, 127.2, 126.3, 126.2, 61.6, 14.0; HRMS (ESI) *m*/*z*: [M + Na]^+^ calcd for C_26_H_20_O_3_NaS: 435.1025; found, 435.1026.

### Methyl 2-([1,1′:3′,1″-Terphenyl]-4′-carbonyl)benzoate (**6r**)

Purified by silica gel column chromatography eluting with hexane/toluene 100:0 to 0:100; 43% yield over two steps (20 mg); colorless oil; ^1^H NMR (400 MHz, CDCl_3_): δ 7.74 (d, *J* = 8.1 Hz, 1H), 7.68–7.65 (m, 3H), 7.64–7.63 (m, 1H), 7.61 (dt, *J* = 7.2, 0.8 Hz, 1H), 7.48 (t, *J* = 7.4 Hz, 2H), 7.42–7.40 (m, 1H), 7.39–7.33 (m, 5H), 7.25–7.18 (m, 3H), 3.72 (s, 3H); ^13^C{^1^H} NMR (101 MHz, CDCl_3_): δ 197.2, 168.1, 144.2, 143.1, 140.5, 140.1, 139.7, 136.0, 131.6, 130.7, 130.5, 129.8, 129.7, 129.14, 129.09, 128.9, 128.2, 128.0, 127.3, 125.6, 52.4; HRMS (ESI) *m*/*z*: [M + Na]^+^ calcd for C_27_H_20_O_3_Na: 415.1305; found, 415.1304.

### Butyl 2-([1,1′:3′,1″-Terphenyl]-4′-carbonyl)benzoate (**6s**)

Purified by silica gel column chromatography eluting with hexane/toluene 100:0 to 0:100; 23% yield over two steps (11.6 mg); colorless oil; ^1^H NMR (400 MHz, CDCl_3_): δ 7.75 (d, *J* = 8.1 Hz, 1H), 7.68–7.62 (m, 5H), 7.48 (t, *J* = 7.6 Hz, 2H), 7.42–7.32 (m, 6H), 7.26–7.17 (m, 3H), 4.16 (t, *J* = 6.8 Hz, 2H), 1.66–1.59 (m, 2H), 1.40–1.31 (m, 2H), 0.90 (t, *J* = 7.4 Hz, 3H); ^13^C{^1^H} NMR (101 MHz, CDCl_3_): δ 197.1, 167.6, 144.2, 143.2, 140.6, 140.3, 139.7, 135.9, 132.0, 131.8, 130.5, 130.4, 129.7, 129.5, 129.1, 129.0, 128.9, 128.2, 128.0, 127.2, 125.5, 77.3, 65.4, 30.4, 19.1, 13.7; HRMS (ESI) *m*/*z*: [M + Na]^+^ calcd for C_30_H_26_O_3_Na: 457.1774; found, 457.1771.

### Isopropyl 2-([1,1′:3′,1″-Terphenyl]-4′-carbonyl)benzoate (**6t**)

Purified by silica gel column chromatography eluting with hexane/toluene 100:0 to 0:100; 12% yield over two steps (5.9 mg); colorless oil; ^1^H NMR (400 MHz, CDCl_3_): δ 7.74 (d, *J* = 8.1 Hz, 1H), 7.66–7.61 (m, 4H), 7.58 (d, *J* = 7.5 Hz, 1H), 7.46 (t, *J* = 7.4 Hz, 2H), 7.41–7.33 (m, 4H), 7.28 (d, *J* = 4.0 Hz, 2H), 7.25–7.15 (m, 3H), 5.15–5.06 (m, 1H), 1.25 (d, *J* = 6.3 Hz, 6H); ^13^C{^1^H} NMR (101 MHz, CDCl_3_): δ 197.0, 167.2, 144.2, 143.2, 140.6, 140.1, 139.8, 136.0, 132.5, 131.8, 130.4, 130.3, 129.7, 129.6, 129.2, 128.9, 128.1, 128.0, 127.3, 127.2, 125.6, 69.1, 21.6; HRMS (ESI) *m*/*z*: [M + Na]^+^ calcd for C_29_H_24_O_3_Na: 443.1618; found, 443.1624.

### 10-Butoxy-3,10-diphenylanthracen-9(10*H*)-one (**7**)

In a 7 mL glass vial, tritylone **5a** (36 mg, 0.1 mmol), *n*-BuOH (9 μL, 0.1 mmol), and *p*-TSA (10 mol %, 0.01 mmol) were added and dissolved in 0.5 mL of DCM. The reaction was stirred for 5 h at ambient temperature. After the reaction was completed (confirmed by TLC), the solvent was removed, and the crude mixture was purified by column chromatography (hexane/EA 20:1 to 10:1) to obtain the pure products **7** (38.5 mg, 92% yield).

Colorless oil; ^1^H NMR (400 MHz, CDCl_3_): δ 8.42 (d, *J* = 8.2 Hz, 1H), 8.36 (d, *J* = 7.9 Hz, 1H), 7.71–7.68 (m, 2H), 7.58–7.50 (m, 4H), 7.45 (q, *J* = 7.3 Hz, 3H), 7.38 (d, *J* = 7.6 Hz, 3H), 7.20 (t, *J* = 7.5 Hz, 2H), 7.11 (t, *J* = 7.2 Hz, 1H), 3.16–3.06 (m, 2H), 1.63–1.57 (m, 2H), 1.50–1.41 (m, 2H), 0.88 (t, *J* = 7.3 Hz, 3H); ^13^C{^1^H} NMR (101 MHz, CDCl_3_): δ 183.3, 146.5, 146.4, 146.1, 145.7, 139.8, 133.9, 131.7, 130.6, 128.9, 128.6, 128.3, 128.2, 128.0, 127.7, 127.3, 126.9, 126.9, 126.6, 125.7, 78.0, 63.3, 32.2, 19.5, 13.9.; HRMS (FAB) *m*/*z*: [M + H]^+^ calcd for C_30_H_27_O_2_: 419.2011; found, 419.2002.

### 2-([1,1′:3′,1″-Terphenyl]-4′-carbonyl)benzoic Acid (**8**)

In a 7 mL glass vial, **6a** (65 mg, 0.16 mmol) was dissolved in 1.4 mL of DCM, then 2N NaOH (dissolved in MeOH) was added; the final concentration of the alkali would be about 0.1–0.2 N, and the mixture was stirred for 24 h at room temperature. After the reaction was completed (confirmed by TLC), the solvent was removed in vacuo, the residue was diluted with water, and the aqueous solution was extracted with DCM. The aqueous phase was then cooled, acidified to pH 2–3 with 2 N HCl, and extracted with DCM. The combined organic layers were dried over Na_2_SO_4_, and the solvent was removed to afford the product **8**. 73% yield (44.8 mg); white solid; mp 111–112 °C; ^1^H NMR (400 MHz, CDCl_3_): δ 7.74–7.70 (m, 2H), 7.64–7.59 (m, 4H), 7.43 (t, *J* = 7.3 Hz, 2H), 7.39–7.30 (m, 6H), 7.23–7.16 (m, 3H); ^13^C{^1^H} NMR (101 MHz, CDCl_3_): δ 197.4, 172.0, 144.3, 143.4, 141.3, 140.6, 139.6, 135.7, 131.9, 131.6, 130.3, 130.1, 129.9, 129.8, 129.5, 129.0, 128.9, 128.1, 128.0, 127.3, 127.2, 125.6; HRMS (ESI) *m*/*z*: [M + H]^+^ calcd for C_26_H_19_O_3_: 379.1329; found, 379.1318.

### 3-([1,1′:3′,1″-Terphenyl]-4′-yl)isobenzofuran-1(3*H*)-one (**9**)

In a 7 mL glass vial, **6a** (85 mg, 0.21 mmol) was dissolved in THF/MeOH in a 3:1 ratio (2 mL). After the mixture was stirred at 0 °C for 10 min, NaBH_4_ (16 mg, 0.42 mmol) was added, and the mixture was stirred at room temperature for 12 h. After the reaction was completed (confirmed by TLC), saturated aqueous NH_4_Cl was added, and the mixture was extracted with EA. The combined organic layers were washed with brine, and the organic phase was dried over Na_2_SO_4_. The solvent was removed on a rotary evaporator. The residue was purified by column chromatography (hexane/EA 20:1 to 5:1) to obtain pure product **9**.

80% yield (60.5 mg); white solid; mp 82–83 °C; ^1^H NMR (400 MHz, CDCl_3_): δ 7.94 (d, *J* = 7.6 Hz, 1H), 7.65–7.57 (m, 6H), 7.54–7.49 (m, 3H), 7.47–7.41 (m, 4H), 7.38–7.35 (m, 1H), 7.25 (dd, *J* = 8.0, 0.8 Hz, 1H), 6.98 (d, *J* = 8.1 Hz, 1H), 6.57 (s, 1H); ^13^C{^1^H} NMR (101 MHz, CDCl_3_): δ 170.6, 150.0, 143.7, 142.0, 139.9, 139.6, 134.2, 132.4, 129.7, 129.2, 128.8, 128.5, 128.4, 127.8, 127.7, 127.1, 126.6, 126.2, 125.5, 123.0, 80.0; HRMS (ESI) *m*/*z*: [M + Na]^+^ calcd for C_26_H_18_O_2_Na: 385.1199; found, 385.1207.

## Data Availability

The data underlying this study are available in the published article and its Supporting Information.

## Supporting Information Available

General information, X-ray analysis data, and NMR spectra of all compounds (PDF)
