# Supplementary material for: Switchable Synthesis of Tritylone Alcohols and 2-Benzoylbenzoate Esters from Spiroindane-1,3-diones
Source: J Org Chem. 2024 Aug 12;89(17):12360–9. doi: 10.1021/acs.joc.4c01296 (PMC11382160; doi:10.1021/acs.joc.4c01296)

**Supporting Information**

# Switchable Synthesis of Tritylone Alcohols and 2-Benzoylbenzoate Esters from Spiroindane-1,3-diones

Jen-Yu Kuan, Jing-Huei Chen, and Jeng-Liang Han*

*Department of Chemistry, National Chung Hsing University, 145 Xingda Rd., South Dist.,* Taichung City 402, Taiwan

E-mail: jlhan@nchu.edu.tw

**Table of Contents:**

1. **General Experimental Details……………………………………………………...S2**
2. **Starting Materials……………………………………………………………….....S3**
3. **References……………………………………………………………………………..S7**
4. **X-Ray Analysis Data…………………………………………………………………...S8**
5. **Copies of NMR Spectra of Products……………………………………………S32**

## 1. General Experimental Details

All commercially available reagents were used without further purification unless otherwise stated. All reaction solvents were purified before use. Proton nuclear magnetic resonance ($^{1}$H NMR) spectra were recorded on a commercial instrument at 400 MHz. Carbon-13 nuclear magnetic resonance ($^{13}$C{$^{1}$H } NMR) spectra were recorded at 100 MHz. The proton signal for residual non-deuterated solvent (δ 7.26 for $CHCl_3$) was used as an internal reference for $^{1}$H NMR spectra. For $^{13}$C{$^{1}$H} NMR spectra, chemical shifts are reported relative to the δ 77.0 resonance of $CHCl_3$. Coupling constants are reported in Hz. Melting points were determined on a BUCHI B-545 melting point apparatus and are uncorrected. High resolution mass spectra were recorded on a Thermo Fisher Scientific LTQ Orbitrap XL mass spectrometer. The single crystal was measured by Bruker D8 VENTURE X-ray Single Crystal Diffractometer. Analytical thin-layer chromatography (TLC) was performed on silica gel 60 F254 pre-coated plates with visualization under UV light. Column chromatography was generally performed using 40-63 μm (230-400 mesh) silica gel, typically using a 50-100:1 weight ratio of silica gel to crude product.

## 2. Starting Materials

### 2.1 Preparation of spiroindane-1,3-diones 3

Spiroindane-1,3-diones **3** were prepared according to known procedures. [1]

**Scheme S1**. Spiroindane-1,3-diones **3** were used in the manuscript.

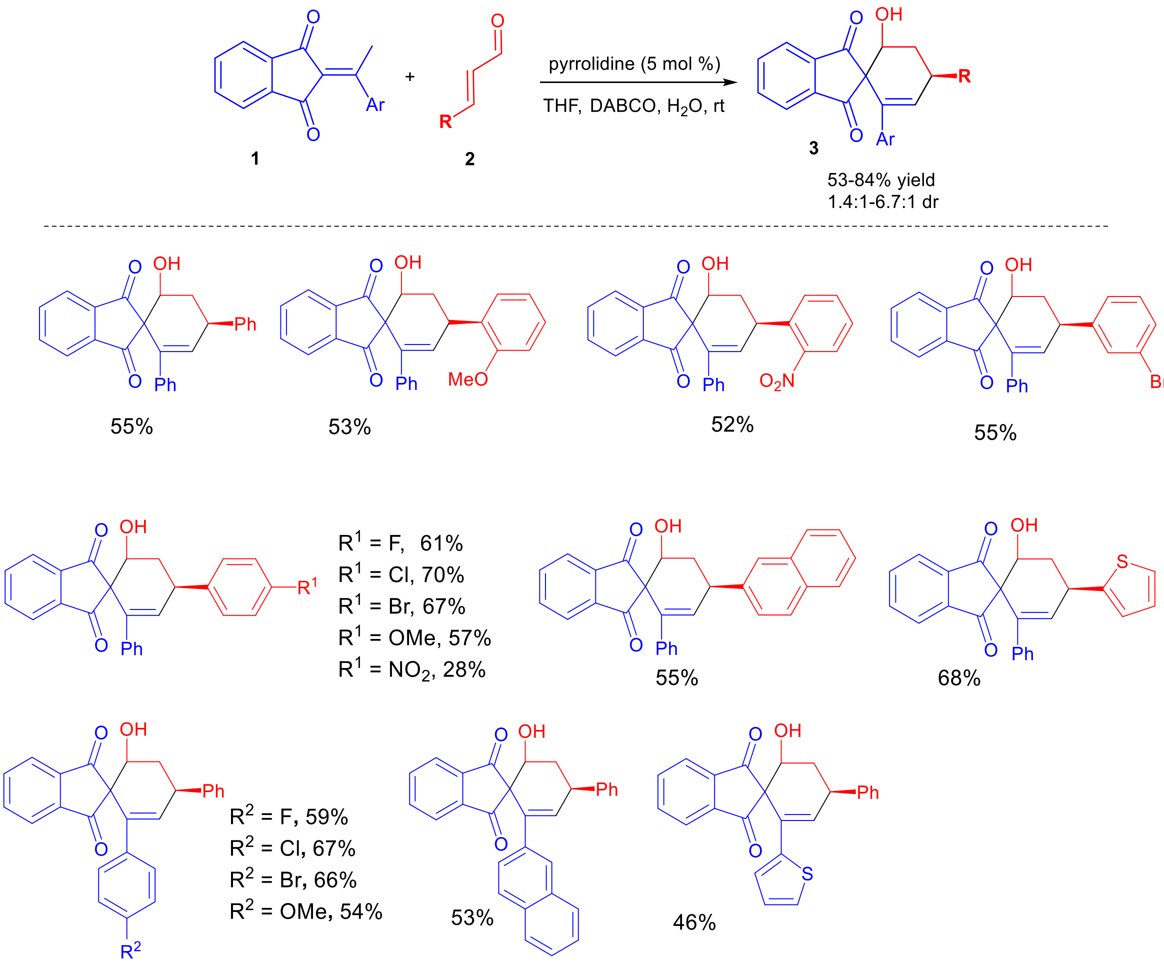


**2-Hydroxy-4-(4-nitrophenyl)-6-phenylspiro[cyclohexene-1,2'-indene]-1',3'-dione**

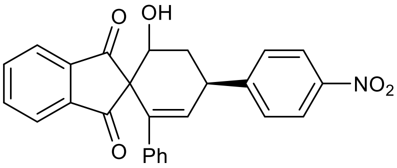


Purified by silica gel column chromatography eluting with Hexane/EA 5:1 to 2:1; 28% yield (24 mg); Light yellow solid; m.p.: 220-221 °C; 2.56:1 of diastereomer ratio; $^1$H NMR (400 MHz, $CDCl_3$): Major: δ 8.24-8.19 (m, 2H), 8.01-7.99 (m, 1H), 7.84-7.79 (m, 2H), 7.76-7.72 (m, 1H), 7.63-7.60 (m, 2H), 7.10-7.02 (m, 3H), 6.91-6.88 (m, 2H), 6.00 (d, *J* = 2.0 Hz, 1H), 4.63 (dd, *J* = 12.0, 3.8 Hz, 1H), 3.91 (ddd, *J* = 10.8, 6.2, 2.4 Hz, 1H), 2.69-2.56 (m, 1H), 2.23 (ddd, *J* = 12.4, 6.0, 4.0 Hz, 1H).; Minor: δ 8.24-8.19 (m, 2H), 7.94-7.90 (m, 2H), 7.84-7.79 (m, 2H), 7.63-7.60 (m, 2H), 7.10-7.02 (m, 3H), 6.96-6.93 (m, 2H), 6.18 (d, *J* = 3.6 Hz, 1H), 4.31 (dd, *J* = 8.4, 2.8 Hz, 1H), 4.11-4.07 (m, 1H), 2.69-2.56

(m, 1H), 2.11 (ddd, $J$ = 13.2, 6.0, 2.8 Hz, 1H). $^{13}$C NMR (101 MHz, $CDCl_3$) Mixture: δ 202.6, 201.7, 200.9, 200.7, 151.9, 151.8, 146.9, 146.8, 143.6, 143.2, 142.5, 142.4, 139.3, 138.9, 136.1, 136.1, 136.0, 135.8, 135.5, 134.2, 133.5, 129.1, 128.8, 128.3, 128.2, 127.6, 127.5, 127.4, 124.0, 123.9, 123.5, 123.5, 123.1, 122.9, 73.0, 68.7, 64.4, 63.9, 42.4, 39.6, 35.8, 34.0.; HRMS (FAB) $m/z$: $[M+H]^+$ calcd. for $C_{26}H_{20}NO_5$: 426.1341, Found: 426.1343.

## 2.2 Preparation of spiroindane-1,3-diones 3a-d1 and 3a-d2

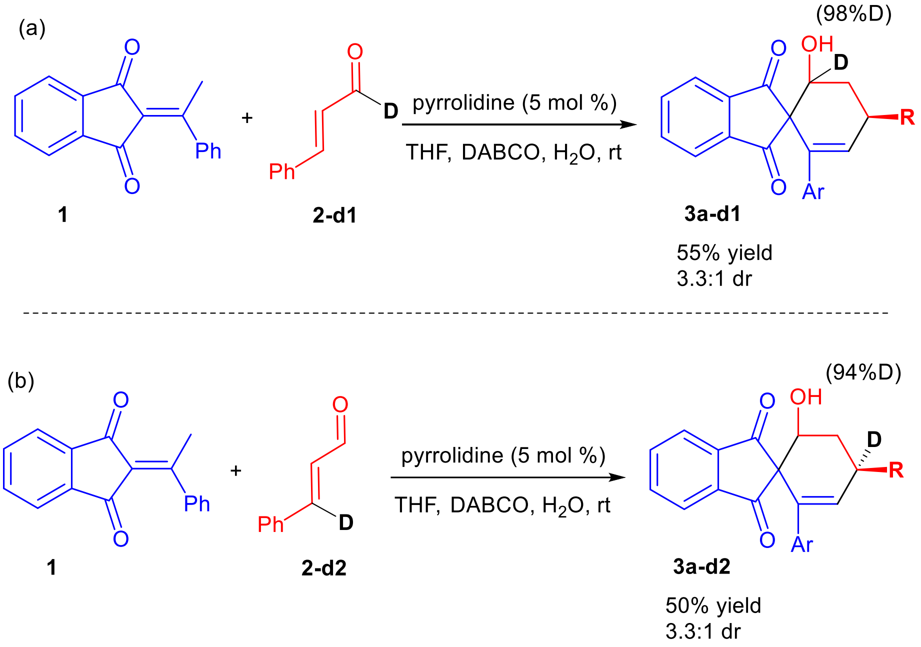


Aldehydes **2-d1** [2] and **2-d2** [3] were prepared according to known procedures.

## 2.3 Determination of the percentage of deuterium-labeling

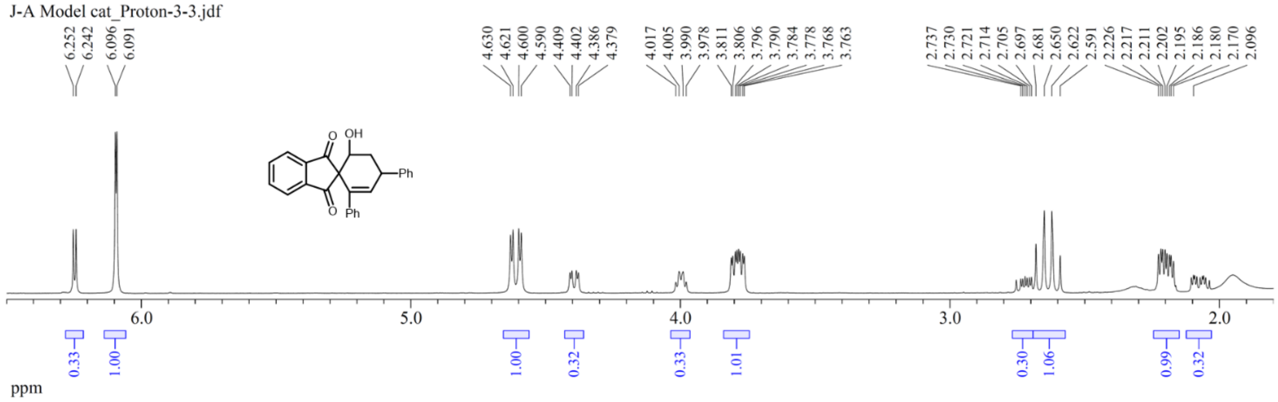


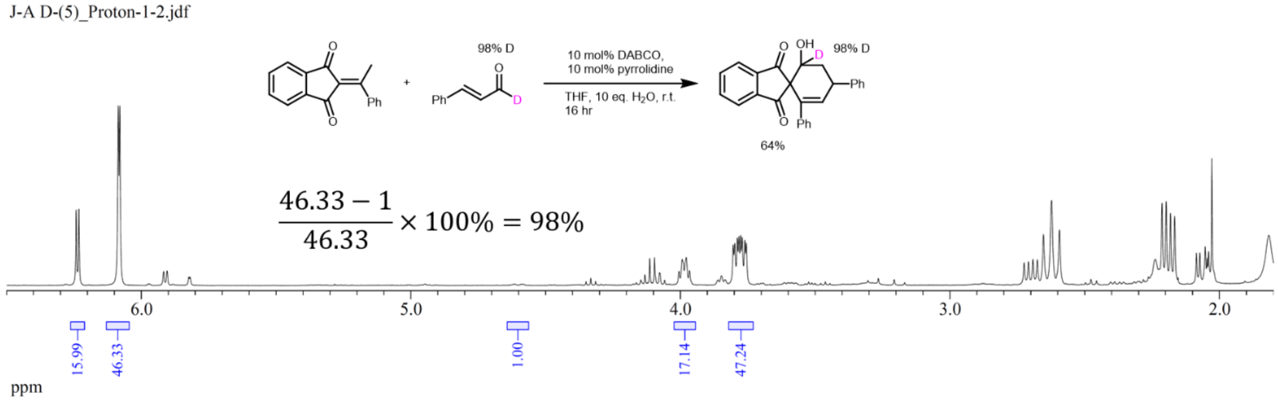


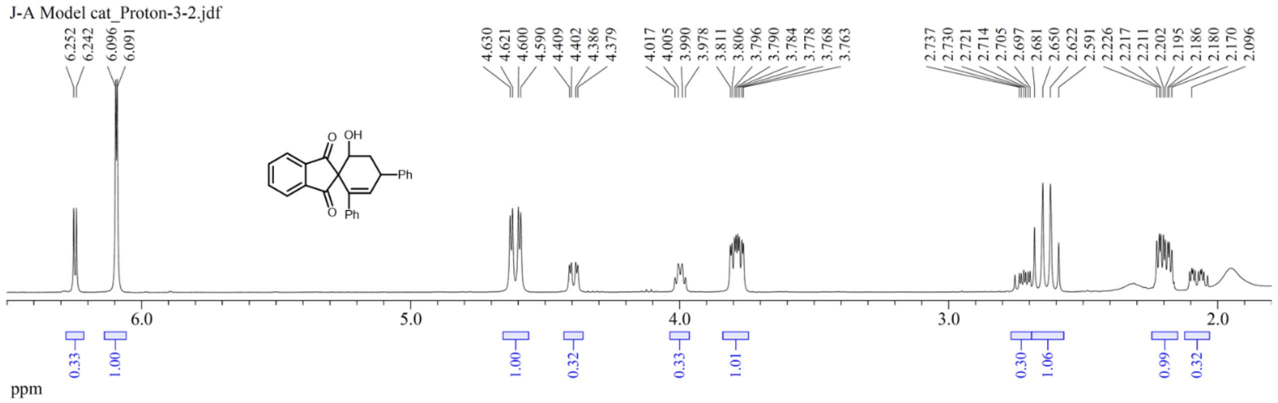


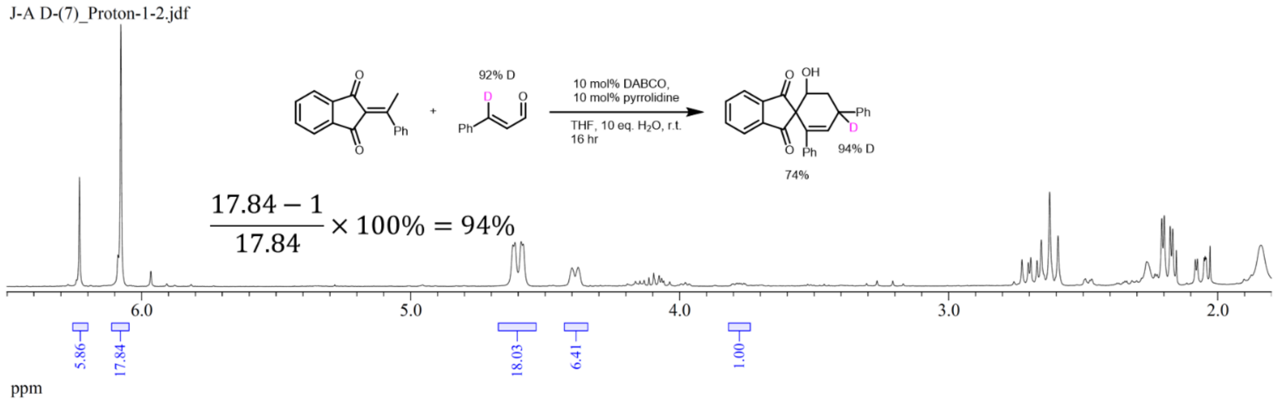

J-A try OMs DBU_Proton-1-2.jdf

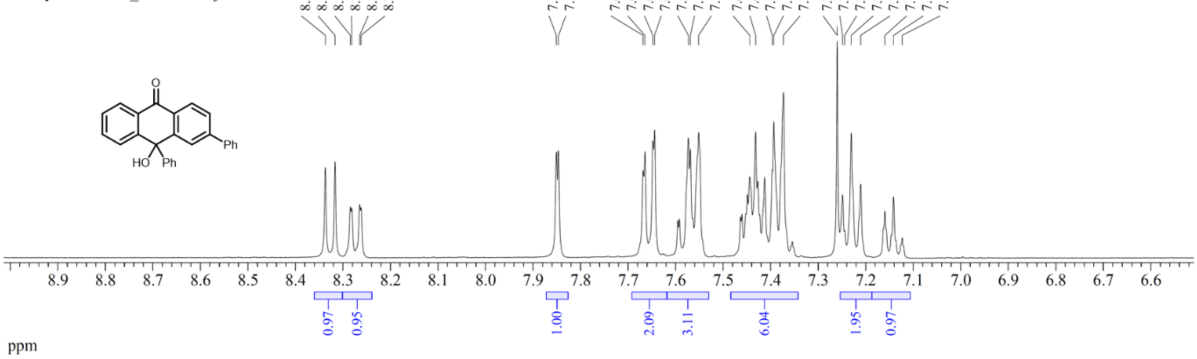


J-F D-(5)_Proton-1-2.jdf

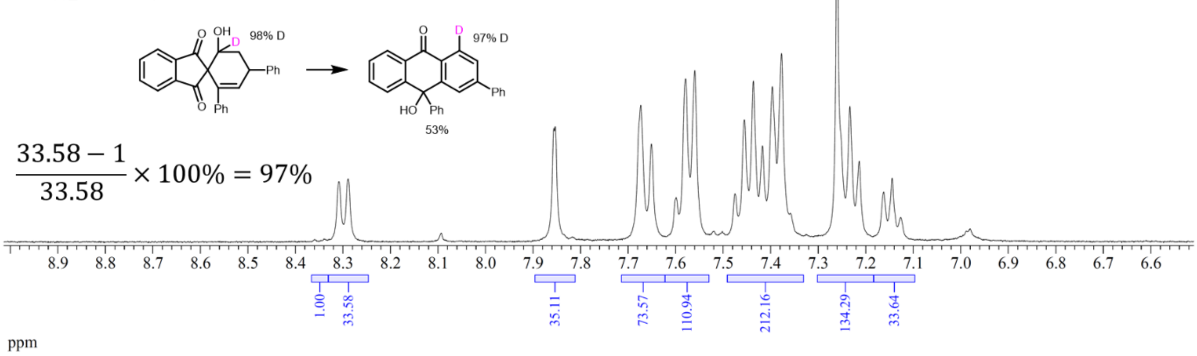


$$\frac{33.58-1}{33.58}\times 100\% = 97\%$$

J-A try OMs DBU_Proton-1-2.jdf

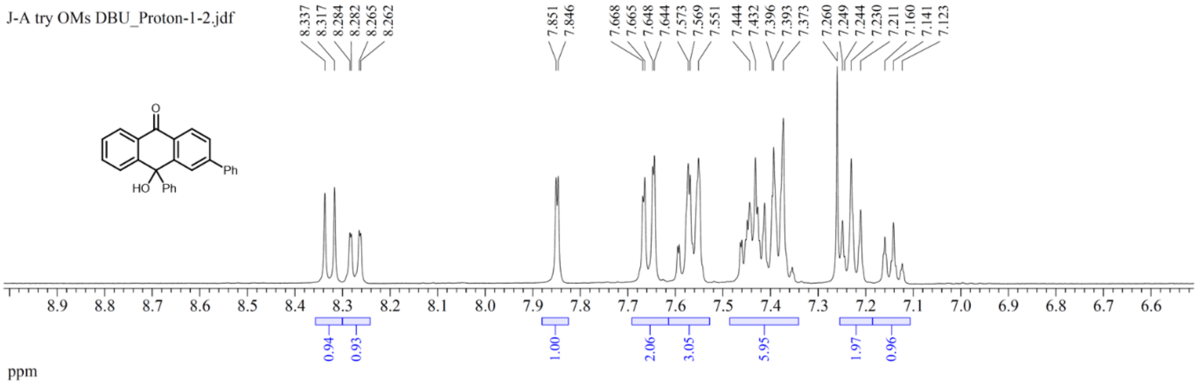


J-F D-(7)_Proton-1-2.jdf

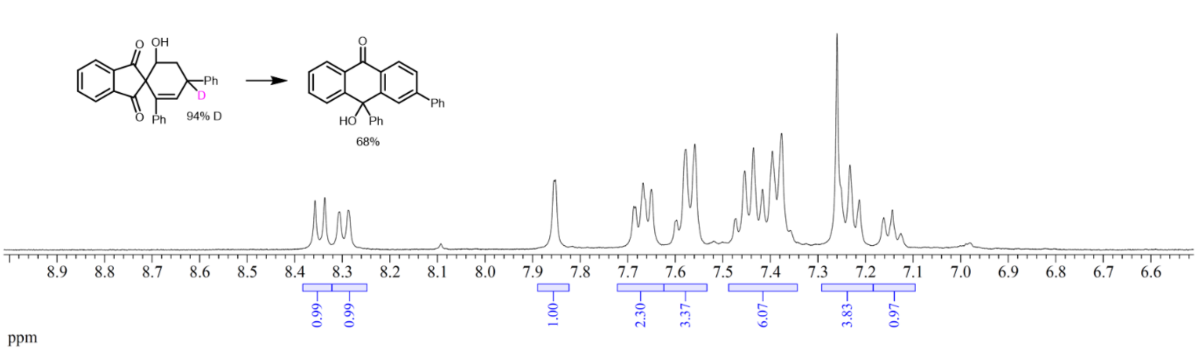

## 3. References

1. Kuan, J.-Y.; Chen, I.-T.; Lin, H.; Han, J.-L. Organocatalytic Vinylogous Michael Addition/Cyclization Cascade of 2-Alkylidene Indane-1,3-diones with Enals: A Regioand Stereocontrolled Diversity-Oriented Route to Indane-1,3-dione Derivatives. *Adv. Synth. Catal.* **2023**, *365*, 3493.
2. Niu, G.-H.; Huang, P.-R.; Chuang, G. J. Triphenylphosphine/triethylamine-mediated decarboxylation of α-oxocarboxylic acids and application in a one-pot synthesis of deuterated aldehydes. *Asian J. Org. Chem.* **2016**, *5*, 57.
3. Wu, Q.; Dong, Z.; Xu, J.; Yang, Z. Sulfur-controlled and Rhodium-catalyzed Formal (3 + 3) Transannulation of Thioacyl Carbenes with Alk-2-enals and Mechanistic Insights. *Org. Biomol. Chem.* **2021**, *19*, 3173

## 4. Absolute Configuration and X-Ray Analysis Data

**CCDC 2348937 (5g)**

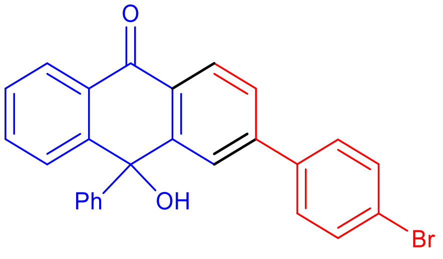


**5g**

ORTEP drawing of **5g** showing thermal ellipsoids at the 50% probability level

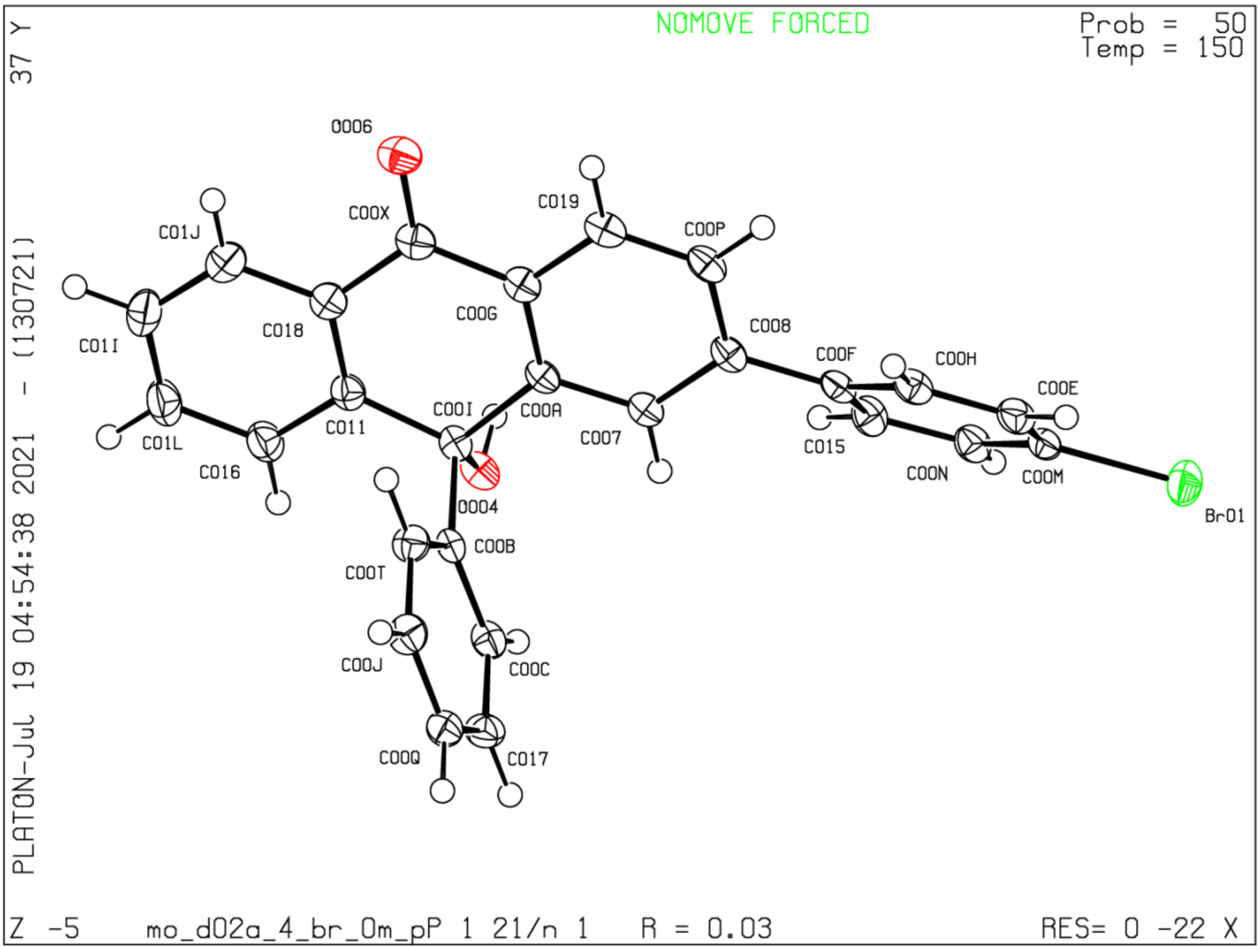


The crystal was obtained by slow evaporation of **5g** in Hexane : DCM = 3:1 at room temperature.

**Table S1**. Crystal data and structure refinement for **5g**.

| | | |
|---|---|---|
| Identification code | mo_d02a_4_br_0m_pl | |
| Empirical formula | C26 H17 Br O2 | |
| Formula weight | 441.30 | |
| Temperature | 150.15 K | |
| Wavelength | 0.71076 Å | |
| Crystal system | Monoclinic | |
| Space group | P 1 21/n 1 | |
| Unit cell dimensions | a = 10.739(3) Å | a= 90°. |
| | b = 9.857(2) Å | b= 94.546(13)°. |
| | c = 17.944(5) Å | g = 90°. |
| Volume | 1893.5(8) Å3 | |
| Z | 4 | |
| Density (calculated) | 1.548 Mg/m3 | |
| Absorption coefficient | 2.192 mm-1 | |
| F(000) | 896 | |
| Crystal size | | |
| Theta range for data collection | 2.359 to 26.449°. | |
| Index ranges | -13<=h<=13, -12<=k<=12, -22<=l<=22 | |
| Reflections collected | 54597 | |
| Independent reflections | 3883 [R(int) = 0.0597] | |
| Completeness to theta = 25.243° | 99.8 % | |
| Absorption correction | None | |
| Max. and min. transmission | 0.7454 and 0.6670 | |
| Refinement method | Full-matrix least-squares on F2 | |
| Data / restraints / parameters | 3883 / 0 / 263 | |
| Goodness-of-fit on F2 | 1.212 | |
| Final R indices [I>2sigma(I)] | R1 = 0.0269, wR2 = 0.0812 | |
| R indices (all data) | R1 = 0.0374, wR2 = 0.1000 | |
| Extinction coefficient | n/a | |
| Largest diff. peak and hole | 0.924 and -0.899 e.Å-3 | |

**Table S2**. Atomic coordinates ( x $10^4$) and equivalent isotropic displacement parameters (Å$^2$x $10^3$) for **5g**. U(eq) is defined as one third of the trace of the orthogonalized $U^{ij}$ tensor.

| | x | y | z | U(eq) |
|---|---|---|---|---|
| Br(01) | 11265(1) | -872(1) | -1818(1) | 32(1) |
| O(004) | 4210(1) | 1681(2) | 293(1) | 22(1) |
| O(006) | 5666(2) | 6682(2) | 1024(1) | 27(1) |
| C(007) | 6822(2) | 2279(2) | 226(1) | 19(1) |
| C(008) | 7940(2) | 2772(2) | -17(1) | 20(1) |
| C(00A) | 6015(2) | 3121(2) | 583(1) | 18(1) |
| C(00B) | 5249(2) | 1554(2) | 1520(1) | 17(1) |
| C(00C) | 5230(2) | 151(2) | 1444(1) | 21(1) |
| C(00E) | 10813(2) | 1073(2) | -692(1) | 24(1) |
| C(00F) | 8753(2) | 1886(2) | -433(1) | 20(1) |
| C(00G) | 6314(2) | 4493(2) | 685(1) | 19(1) |
| C(00H) | 10058(2) | 1897(2) | -290(1) | 23(1) |
| C(00I) | 4838(2) | 2499(2) | 864(1) | 18(1) |
| C(00J) | 6099(2) | 1298(2) | 2800(1) | 25(1) |
| C(00M) | 10262(2) | 251(2) | -1248(1) | 22(1) |
| C(00N) | 8975(2) | 227(2) | -1412(1) | 23(1) |

| | | | | |
|---|---|---|---|---|
| C(00P) | 8250(2) | 4140(2) | 114(1) | 22(1) |
| C(00Q) | 6064(2) | -112(2) | 2724(1) | 23(1) |
| C(00T) | 5699(2) | 2124(2) | 2203(1) | 23(1) |
| C(00X) | 5443(2) | 5452(2) | 1000(1) | 21(1) |
| C(011) | 3941(2) | 3544(2) | 1144(1) | 21(1) |
| C(015) | 8231(2) | 1036(2) | -994(1) | 23(1) |
| C(016) | 2772(2) | 3093(2) | 1331(1) | 27(1) |
| C(017) | 5630(2) | -678(2) | 2047(1) | 25(1) |
| C(018) | 4256(2) | 4909(2) | 1251(1) | 21(1) |
| C(019) | 7444(2) | 4988(2) | 449(1) | 22(1) |
| C(01I) | 2271(2) | 5337(3) | 1758(1) | 30(1) |
| C(01J) | 3414(2) | 5798(2) | 1561(1) | 26(1) |
| C(01L) | 1947(2) | 3978(3) | 1635(1) | 31(1) |

**Table S3**. Bond lengths [Å] and angles [°] for **5g**.

| | |
|---|---|
| Br(01)-C(00M) | 1.897(2) |
| O(004)-C(00I) | 1.430(2) |
| O(006)-C(00X) | 1.236(3) |
| C(007)-C(008) | 1.397(3) |
| C(007)-C(00A) | 1.392(3) |
| C(008)-C(00F) | 1.479(3) |
| C(008)-C(00P) | 1.404(3) |
| C(00A)-C(00G) | 1.399(3) |
| C(00A)-C(00I) | 1.526(3) |
| C(00B)-C(00C) | 1.389(3) |
| C(00B)-C(00I) | 1.538(3) |
| C(00B)-C(00T) | 1.399(3) |
| C(00C)-C(017) | 1.395(3) |
| C(00E)-C(00H) | 1.389(3) |
| C(00E)-C(00M) | 1.382(3) |
| C(00F)-C(00H) | 1.404(3) |
| C(00F)-C(015) | 1.393(3) |
| C(00G)-C(00X) | 1.474(3) |
| C(00G)-C(019) | 1.405(3) |
| C(00I)-C(011) | 1.522(3) |
| C(00J)-C(00Q) | 1.396(3) |
| C(00J)-C(00T) | 1.388(3) |
| C(00M)-C(00N) | 1.391(3) |
| C(00N)-C(015) | 1.389(3) |
| C(00P)-C(019) | 1.376(3) |
| C(00Q)-C(017) | 1.384(3) |
| C(00X)-C(018) | 1.485(3) |
| C(011)-C(016) | 1.397(3) |
| C(011)-C(018) | 1.398(3) |
| C(016)-C(01L) | 1.386(3) |
| C(018)-C(01J) | 1.405(3) |
| C(01I)-C(01J) | 1.381(4) |
| C(01I)-C(01L) | 1.397(3) |
| C(00A)-C(007)-C(008) | 121.20(19) |
| C(007)-C(008)-C(00F) | 120.61(19) |
| C(007)-C(008)-C(00P) | 118.7(2) |

| | |
|---|---|
| C(00P)-C(008)-C(00F) | 120.63(19) |
| C(007)-C(00A)-C(00G) | 119.48(18) |
| C(007)-C(00A)-C(00I) | 118.35(18) |
| C(00G)-C(00A)-C(00I) | 122.16(18) |
| C(00C)-C(00B)-C(00I) | 121.84(17) |
| C(00C)-C(00B)-C(00T) | 119.12(18) |
| C(00T)-C(00B)-C(00I) | 119.00(18) |
| C(00B)-C(00C)-C(017) | 120.43(19) |
| C(00M)-C(00E)-C(00H) | 118.9(2) |
| C(00H)-C(00F)-C(008) | 121.62(19) |
| C(015)-C(00F)-C(008) | 120.01(18) |
| C(015)-C(00F)-C(00H) | 118.4(2) |
| C(00A)-C(00G)-C(00X) | 121.65(19) |
| C(00A)-C(00G)-C(019) | 119.43(19) |
| C(019)-C(00G)-C(00X) | 118.86(19) |
| C(00E)-C(00H)-C(00F) | 121.1(2) |
| O(004)-C(00I)-C(00A) | 110.20(15) |
| O(004)-C(00I)-C(00B) | 107.29(15) |
| O(004)-C(00I)-C(011) | 110.05(16) |
| C(00A)-C(00I)-C(00B) | 107.52(16) |
| C(011)-C(00I)-C(00A) | 113.58(17) |
| C(011)-C(00I)-C(00B) | 107.96(16) |
| C(00T)-C(00J)-C(00Q) | 120.28(19) |
| C(00E)-C(00M)-Br(01) | 120.16(16) |
| C(00E)-C(00M)-C(00N) | 121.6(2) |
| C(00N)-C(00M)-Br(01) | 118.27(17) |
| C(015)-C(00N)-C(00M) | 118.8(2) |
| C(019)-C(00P)-C(008) | 120.5(2) |
| C(017)-C(00Q)-C(00J) | 119.49(19) |
| C(00J)-C(00T)-C(00B) | 120.33(19) |
| O(006)-C(00X)-C(00G) | 121.0(2) |
| O(006)-C(00X)-C(018) | 120.8(2) |
| C(00G)-C(00X)-C(018) | 118.11(18) |
| C(016)-C(011)-C(00I) | 117.94(19) |
| C(016)-C(011)-C(018) | 119.0(2) |
| C(018)-C(011)-C(00I) | 122.97(18) |
| C(00N)-C(015)-C(00F) | 121.3(2) |
| C(01L)-C(016)-C(011) | 120.6(2) |

| | |
|---|---|
| C(00Q)-C(017)-C(00C) | 120.35(19) |
| C(011)-C(018)-C(00X) | 120.65(19) |
| C(011)-C(018)-C(01J) | 119.9(2) |
| C(01J)-C(018)-C(00X) | 119.31(19) |
| C(00P)-C(019)-C(00G) | 120.60(19) |
| C(01J)-C(01I)-C(01L) | 119.4(2) |
| C(01I)-C(01J)-C(018) | 120.6(2) |
| C(016)-C(01L)-C(01I) | 120.4(2) |

Symmetry transformations used to generate equivalent atoms:

**Table S4**. Anisotropic displacement parameters ($Å^2$x $10^3$) for **5g**. The anisotropic displacement factor exponent takes the form: $-2p^2[ h^2 a^{*2}U^{11} + ... + 2 h k a^* b^* U^{12} ]$

| | U11<br>U33<br>U12 | U22<br>U23 | U13 |
|---|---|---|---|
| Br(01) | 27(1) | 36(1) | 35(1) |
| | 2(1) | 9(1) | 9(1) |
| O(004) | 22(1) | 27(1) | 15(1) |
| | -1(1) | -1(1) | -8(1) |
| O(006) | 39(1) | 22(1) | 21(1) |
| | -4(1) | 2(1) | -6(1) |
| C(007) | 20(1) | 22(1) | 17(1) |
| | 1(1) | 1(1) | -4(1) |
| C(008) | 18(1) | 27(1) | 14(1) |
| | 3(1) | -1(1) | -3(1) |
| C(00A) | 19(1) | 22(1) | 13(1) |
| | 1(1) | 0(1) | -4(1) |
| C(00B) | 16(1) | 20(1) | 16(1) |
| | -1(1) | 4(1) | -2(1) |
| C(00C) | 22(1) | 23(1) | 20(1) |
| | -4(1) | 4(1) | -6(1) |
| C(00E) | 15(1) | 28(1) | 30(1) |
| | 8(1) | 2(1) | -1(1) |
| C(00F) | 18(1) | 24(1) | 17(1) |
| | 6(1) | 2(1) | -3(1) |
| C(00G) | 22(1) | 23(1) | 11(1) |
| | -1(1) | -3(1) | -3(1) |
| C(00H) | 19(1) | 27(1) | 22(1) |
| | 5(1) | -1(1) | -4(1) |
| C(00I) | 18(1) | 21(1) | 16(1) |
| | -3(1) | 1(1) | -4(1) |
| C(00J) | 33(1) | 28(1) | 15(1) |
| | -3(1) | 0(1) | 2(1) |
| C(00M) | 21(1) | 23(1) | 23(1) |
| | 8(1) | 7(1) | 3(1) |
| C(00N) | 22(1) | 28(1) | 20(1) |

| | | | |
|---|---|---|---|
| | 2(1) | 2(1) | -1(1) |
| C(00P) | 18(1) | 28(1) | 18(1) |
| | 2(1) | -1(1) | -7(1) |
| C(00Q) | 26(1) | 25(1) | 20(1) |
| | 2(1) | 3(1) | 1(1) |
| C(00T) | 28(1) | 20(1) | 20(1) |
| | -4(1) | 2(1) | -1(1) |
| C(00X) | 27(1) | 24(1) | 12(1) |
| | -1(1) | -3(1) | -3(1) |
| C(011) | 21(1) | 26(1) | 15(1) |
| | 2(1) | 1(1) | 2(1) |
| C(015) | 17(1) | 30(1) | 23(1) |
| | 1(1) | 1(1) | -2(1) |
| C(016) | 23(1) | 31(1) | 28(1) |
| | 3(1) | 5(1) | -1(1) |
| C(017) | 28(1) | 19(1) | 26(1) |
| | 0(1) | 4(1) | -4(1) |
| C(018) | 24(1) | 25(1) | 14(1) |
| | 0(1) | -2(1) | 2(1) |
| C(019) | 25(1) | 24(1) | 16(1) |
| | 0(1) | -2(1) | -7(1) |
| C(01I) | 29(1) | 39(1) | 22(1) |
| | -1(1) | 3(1) | 12(1) |
| C(01J) | 32(1) | 28(1) | 18(1) |
| | -1(1) | -3(1) | 7(1) |
| C(01L) | 22(1) | 42(1) | 30(1) |
| | 4(1) | 8(1) | 3(1) |

**Table S5**. Atomic coordinates ( x $10^4$) and equivalent isotropic displacement parameters ($Å^2$x $10^3$) for **5g**.

| | x<br>U(eq) | y | z |
|---|---|---|---|
| H(004) | 4097 | 2135 | - |
| 103 | 32 | | |
| H(007) | 6609 | 1352 | 147 |
| | 23 | | |
| H(00C) | 4942 | - | |
| 246 | 980 | 26 | |
| H(00E) | 11694 | 1074 | - |
| 585 | 29 | | |
| H(00H) | 10430 | 2477 | 87 |
| | 27 | | |
| H(00J) | 6398 | 1692 | 3263 |
| | 30 | | |
| H(00N) | 8610 | -332 | - |
| 1801 | 28 | | |
| H(00P) | 9023 | 4482 | - |
| 29 | 26 | | |
| H(00Q) | 6337 | - | |
| 678 | 3133 | 28 | |
| H(00T) | 5731 | 3083 | 2258 |
| | 27 | | |
| H(015) | 7349 | 1009 | - |
| 1093 | 28 | | |
| H(016) | 2541 | 2172 | 1249 |
| | 33 | | |
| H(017) | 5603 | - | |
| 1637 | 1992 | 30 | |
| H(019) | 7655 | 5918 | 522 |
| | 26 | | |
| H(01I) | 1711 | 5938 | 1975 |
| | 36 | | |
| H(01J) | 3634 | 6725 | 1636 |

| | | | |
|---|---|---|---|
| H(01L) | 31<br>1155<br>37 | 3658 | 1761 |

**Table S6**. Torsion angles [°] for **5g**.

| | |
|---|---|
| Br(01)-C(00M)-C(00N)-C(015) | 179.44(15) |
| O(004)-C(00I)-C(011)-C(016) | 48.6(2) |
| O(004)-C(00I)-C(011)-C(018) | -134.32(19) |
| O(006)-C(00X)-C(018)-C(011) | 172.53(18) |
| O(006)-C(00X)-C(018)-C(01J) | -3.9(3) |
| C(007)-C(008)-C(00F)-C(00H) | -138.4(2) |
| C(007)-C(008)-C(00F)-C(015) | 43.0(3) |
| C(007)-C(008)-C(00P)-C(019) | -2.6(3) |
| C(007)-C(00A)-C(00G)-C(00X) | 174.70(18) |
| C(007)-C(00A)-C(00G)-C(019) | -2.2(3) |
| C(007)-C(00A)-C(00I)-O(004) | -47.9(2) |
| C(007)-C(00A)-C(00I)-C(00B) | 68.7(2) |
| C(007)-C(00A)-C(00I)-C(011) | -171.88(17) |
| C(008)-C(007)-C(00A)-C(00G) | 1.4(3) |
| C(008)-C(007)-C(00A)-C(00I) | -177.36(17) |
| C(008)-C(00F)-C(00H)-C(00E) | -179.17(19) |
| C(008)-C(00F)-C(015)-C(00N) | 177.79(19) |
| C(008)-C(00P)-C(019)-C(00G) | 1.8(3) |
| C(00A)-C(007)-C(008)-C(00F) | -176.48(18) |
| C(00A)-C(007)-C(008)-C(00P) | 1.0(3) |
| C(00A)-C(00G)-C(00X)-O(006) | -173.24(18) |
| C(00A)-C(00G)-C(00X)-C(018) | 3.5(3) |
| C(00A)-C(00G)-C(019)-C(00P) | 0.6(3) |
| C(00A)-C(00I)-C(011)-C(016) | 172.63(17) |
| C(00A)-C(00I)-C(011)-C(018) | -10.2(3) |
| C(00B)-C(00C)-C(017)-C(00Q) | -0.8(3) |
| C(00B)-C(00I)-C(011)-C(016) | -68.2(2) |
| C(00B)-C(00I)-C(011)-C(018) | 108.9(2) |
| C(00C)-C(00B)-C(00I)-O(004) | 13.1(2) |
| C(00C)-C(00B)-C(00I)-C(00A) | -105.4(2) |
| C(00C)-C(00B)-C(00I)-C(011) | 131.68(19) |
| C(00C)-C(00B)-C(00T)-C(00J) | -1.1(3) |
| C(00E)-C(00M)-C(00N)-C(015) | -1.0(3) |
| C(00F)-C(008)-C(00P)-C(019) | 174.85(18) |
| C(00G)-C(00A)-C(00I)-O(004) | 133.37(19) |
| C(00G)-C(00A)-C(00I)-C(00B) | -110.0(2) |

| | |
|---|---|
| C(00G)-C(00A)-C(00I)-C(011) | 9.4(3) |
| C(00G)-C(00X)-C(018)-C(011) | -4.2(3) |
| C(00G)-C(00X)-C(018)-C(01J) | 179.37(18) |
| C(00H)-C(00E)-C(00M)-Br(01) | 179.20(15) |
| C(00H)-C(00E)-C(00M)-C(00N) | -0.4(3) |
| C(00H)-C(00F)-C(015)-C(00N) | -0.8(3) |
| C(00I)-C(00A)-C(00G)-C(00X) | -6.6(3) |
| C(00I)-C(00A)-C(00G)-C(019) | 176.54(17) |
| C(00I)-C(00B)-C(00C)-C(017) | 178.89(19) |
| C(00I)-C(00B)-C(00T)-C(00J) | -178.80(19) |
| C(00I)-C(011)-C(016)-C(01L) | 175.7(2) |
| C(00I)-C(011)-C(018)-C(00X) | 8.1(3) |
| C(00I)-C(011)-C(018)-C(01J) | -175.50(18) |
| C(00J)-C(00Q)-C(017)-C(00C) | 0.1(3) |
| C(00M)-C(00E)-C(00H)-C(00F) | 1.2(3) |
| C(00M)-C(00N)-C(015)-C(00F) | 1.6(3) |
| C(00P)-C(008)-C(00F)-C(00H) | 44.2(3) |
| C(00P)-C(008)-C(00F)-C(015) | -134.4(2) |
| C(00Q)-C(00J)-C(00T)-C(00B) | 0.4(3) |
| C(00T)-C(00B)-C(00C)-C(017) | 1.3(3) |
| C(00T)-C(00B)-C(00I)-O(004) | -169.29(17) |
| C(00T)-C(00B)-C(00I)-C(00A) | 72.2(2) |
| C(00T)-C(00B)-C(00I)-C(011) | -50.7(2) |
| C(00T)-C(00J)-C(00Q)-C(017) | 0.1(3) |
| C(00X)-C(00G)-C(019)-C(00P) | -176.40(18) |
| C(00X)-C(018)-C(01J)-C(01I) | 176.10(19) |
| C(011)-C(016)-C(01L)-C(01I) | 0.1(4) |
| C(011)-C(018)-C(01J)-C(01I) | -0.3(3) |
| C(015)-C(00F)-C(00H)-C(00E) | -0.6(3) |
| C(016)-C(011)-C(018)-C(00X) | -174.79(19) |
| C(016)-C(011)-C(018)-C(01J) | 1.6(3) |
| C(018)-C(011)-C(016)-C(01L) | -1.5(3) |
| C(019)-C(00G)-C(00X)-O(006) | 3.7(3) |
| C(019)-C(00G)-C(00X)-C(018) | -179.58(17) |
| C(01J)-C(01I)-C(01L)-C(016) | 1.1(3) |
| C(01L)-C(01I)-C(01J)-C(018) | -1.0(3) |

Symmetry transformations used to generate equivalent atoms:

**CCDC 2348953 (8)**

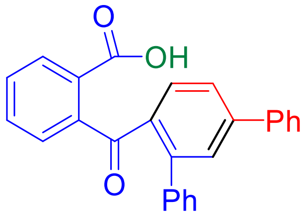


**8**

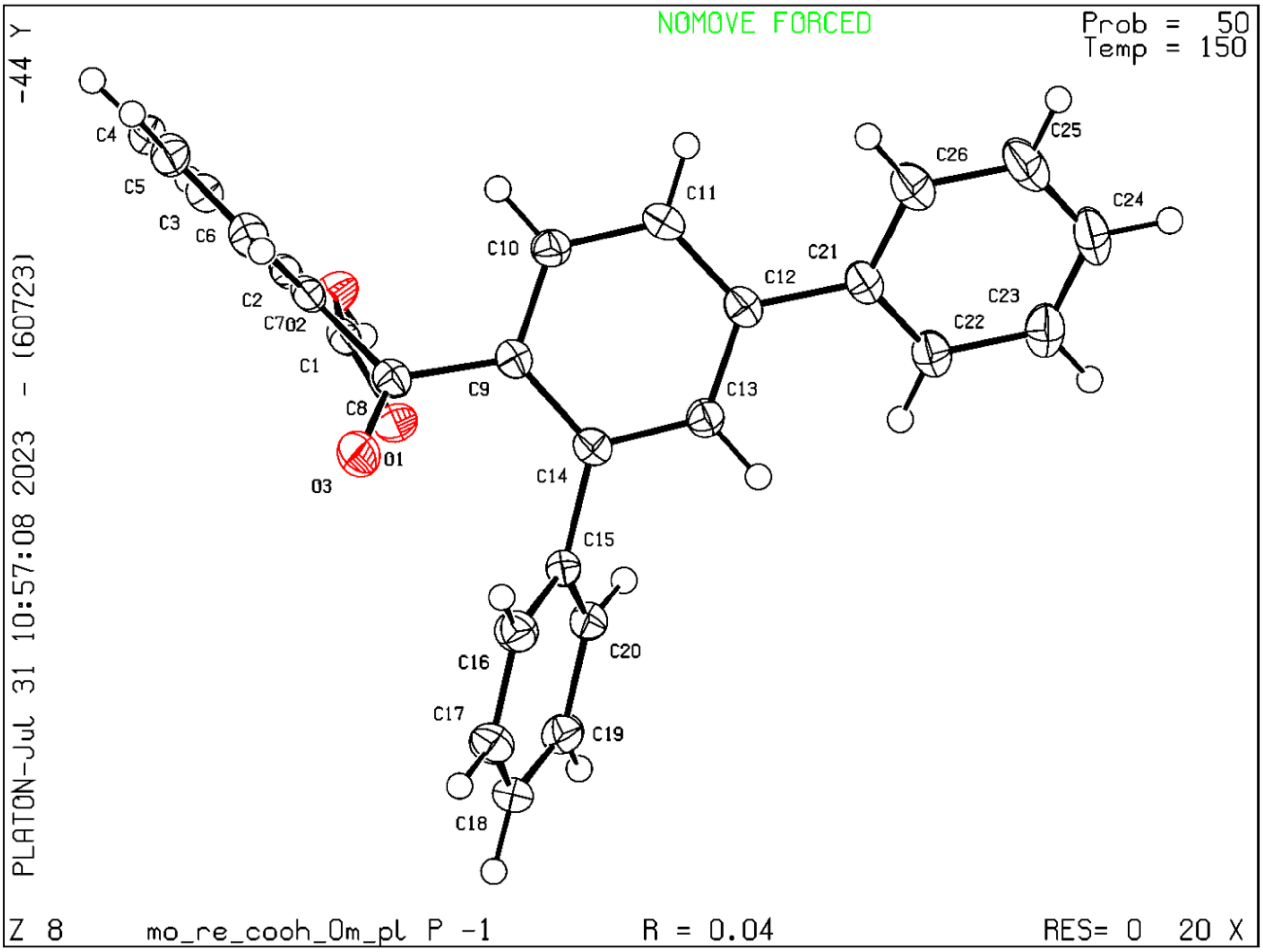


ORTEP drawing of **8** showing thermal ellipsoids at the 50% probability level

The crystal was obtained by slow evaporation of **8** in Hexane : DCM = 3:1 at room temperature.

**Table S7**. Crystal data and structure refinement for **8**.

| | | |
|---|---|---|
| Identification code | mo_re_cooh_0m_pl | |
| Empirical formula | C26 H18 O3 | |
| Formula weight | 378.40 | |
| Temperature | 150.15 K | |
| Wavelength | 0.71076 Å | |
| Crystal system | Triclinic | |
| Space group | P-1 | |
| Unit cell dimensions | a = 6.9362(4) Å | a= 108.430(2)°. |
| | b = 9.9775(6) Å | b= 99.787(2)°. |
| | c = 14.7045(9) Å | g = 91.730(2)°. |
| Volume | 947.55(10) $Å^3$ | |
| Z | 2 | |
| Density (calculated) | 1.326 $Mg/m^3$ | |
| Absorption coefficient | 0.086 $mm^{-1}$ | |
| F(000) | 396 | |
| Crystal size | | |
| Theta range for data collection | 2.974 to 26.355°. | |
| Index ranges | -8<=h<=8, -12<=k<=12, -18<=l<=18 | |
| Reflections collected | 25421 | |
| Independent reflections | 3857 [R(int) = 0.0294] | |
| Completeness to theta = 25.243° | 99.9 % | |
| Absorption correction | None | |
| Refinement method | Full-matrix least-squares on $F^2$ | |
| Data / restraints / parameters | 3857 / 0 / 263 | |
| Goodness-of-fit on $F^2$ | 1.055 | |
| Final R indices [I>2sigma(I)] | R1 = 0.0354, wR2 = 0.0834 | |
| R indices (all data) | R1 = 0.0433, wR2 = 0.0919 | |
| Extinction coefficient | n/a | |
| Largest diff. peak and hole | 0.210 and -0.233 e.$Å^{-3}$ | |

**Table S8**. Atomic coordinates (x $10^4$) and equivalent isotropic displacement parameters (Å$^2$x $10^3$) for **8**. U(eq) is defined as one third of the trace of the orthogonalized $U^{ij}$ tensor.

| | x | y | z | U(eq) |
|---|---|---|---|---|
| O(1) | 3447(1) | 4974(1) | 722(1) | 23(1) |
| O(2) | 3674(1) | 6511(1) | -93(1) | 27(1) |
| O(3) | 171(1) | 4496(1) | 2114(1) | 31(1) |
| C(1) | 2856(2) | 5972(1) | 472(1) | 19(1) |
| C(2) | 1122(2) | 6686(1) | 790(1) | 20(1) |
| C(3) | 189(2) | 7542(1) | 295(1) | 24(1) |
| C(4) | -1469(2) | 8173(1) | 567(1) | 29(1) |
| C(5) | -2209(2) | 7942(1) | 1325(1) | 29(1) |
| C(6) | -1330(2) | 7059(1) | 1802(1) | 26(1) |
| C(7) | 359(2) | 6433(1) | 1548(1) | 20(1) |
| C(8) | 1210(2) | 5503(1) | 2118(1) | 21(1) |
| C(9) | 3187(2) | 5950(1) | 2763(1) | 20(1) |
| C(10) | 3749(2) | 7403(1) | 3182(1) | 23(1) |
| C(11) | 5509(2) | 7916(1) | 3833(1) | 23(1) |
| C(12) | 6795(2) | 6978(1) | 4070(1) | 22(1) |
| C(13) | 6255(2) | 5523(1) | 3624(1) | 22(1) |
| C(14) | 4475(2) | 4981(1) | 2983(1) | 20(1) |
| C(15) | 4186(2) | 3408(1) | 2493(1) | 21(1) |
| C(16) | 2685(2) | 2530(1) | 2594(1) | 28(1) |
| C(17) | 2527(2) | 1082(1) | 2107(1) | 32(1) |
| C(18) | 3846(2) | 484(1) | 1519(1) | 31(1) |
| C(19) | 5376(2) | 1341(1) | 1440(1) | 28(1) |
| C(20) | 5554(2) | 2792(1) | 1931(1) | 23(1) |
| C(21) | 8711(2) | 7486(1) | 4750(1) | 23(1) |
| C(22) | 10337(2) | 6707(1) | 4629(1) | 27(1) |
| C(23) | 12134(2) | 7187(2) | 5261(1) | 32(1) |
| C(24) | 12324(2) | 8443(2) | 6029(1) | 36(1) |
| C(25) | 10726(2) | 9226(2) | 6156(1) | 39(1) |
| C(26) | 8930(2) | 8761(2) | 5523(1) | 32(1) |

**Table S9**. Bond lengths [Å] and angles [°] for **8**.

| | |
|---|---|
| O(1)-C(1) | 1.2246(14) |
| O(2)-H(2) | 0.8400 |
| O(2)-C(1) | 1.3124(14) |
| O(3)-C(8) | 1.2154(15) |
| C(1)-C(2) | 1.4854(15) |
| C(2)-C(3) | 1.3944(17) |
| C(2)-C(7) | 1.3993(17) |
| C(3)-H(3) | 0.9500 |
| C(3)-C(4) | 1.3863(17) |
| C(4)-H(4) | 0.9500 |
| C(4)-C(5) | 1.382(2) |
| C(5)-H(5) | 0.9500 |
| C(5)-C(6) | 1.3851(19) |
| C(6)-H(6) | 0.9500 |
| C(6)-C(7) | 1.3951(16) |
| C(7)-C(8) | 1.5067(17) |
| C(8)-C(9) | 1.4939(16) |
| C(9)-C(10) | 1.3970(16) |
| C(9)-C(14) | 1.4109(16) |
| C(10)-H(10) | 0.9500 |
| C(10)-C(11) | 1.3865(17) |
| C(11)-H(11) | 0.9500 |
| C(11)-C(12) | 1.3947(17) |
| C(12)-C(13) | 1.3998(16) |
| C(12)-C(21) | 1.4870(16) |
| C(13)-H(13) | 0.9500 |
| C(13)-C(14) | 1.3951(16) |
| C(14)-C(15) | 1.4967(16) |
| C(15)-C(16) | 1.3974(17) |
| C(15)-C(20) | 1.3933(17) |
| C(16)-H(16) | 0.9500 |
| C(16)-C(17) | 1.3860(18) |
| C(17)-H(17) | 0.9500 |
| C(17)-C(18) | 1.384(2) |
| C(18)-H(18) | 0.9500 |
| C(18)-C(19) | 1.3862(19) |

| | |
|---|---|
| C(19)-H(19) | 0.9500 |
| C(19)-C(20) | 1.3892(17) |
| C(20)-H(20) | 0.9500 |
| C(21)-C(22) | 1.3940(18) |
| C(21)-C(26) | 1.3959(18) |
| C(22)-H(22) | 0.9500 |
| C(22)-C(23) | 1.3908(18) |
| C(23)-H(23) | 0.9500 |
| C(23)-C(24) | 1.381(2) |
| C(24)-H(24) | 0.9500 |
| C(24)-C(25) | 1.380(2) |
| C(25)-H(25) | 0.9500 |
| C(25)-C(26) | 1.3898(19) |
| C(26)-H(26) | 0.9500 |
| C(1)-O(2)-H(2) | 109.5 |
| O(1)-C(1)-O(2) | 124.04(10) |
| O(1)-C(1)-C(2) | 122.12(10) |
| O(2)-C(1)-C(2) | 113.84(10) |
| C(3)-C(2)-C(1) | 119.84(11) |
| C(3)-C(2)-C(7) | 120.11(11) |
| C(7)-C(2)-C(1) | 119.95(10) |
| C(2)-C(3)-H(3) | 119.9 |
| C(4)-C(3)-C(2) | 120.17(12) |
| C(4)-C(3)-H(3) | 119.9 |
| C(3)-C(4)-H(4) | 120.1 |
| C(5)-C(4)-C(3) | 119.81(12) |
| C(5)-C(4)-H(4) | 120.1 |
| C(4)-C(5)-H(5) | 119.8 |
| C(4)-C(5)-C(6) | 120.49(11) |
| C(6)-C(5)-H(5) | 119.8 |
| C(5)-C(6)-H(6) | 119.8 |
| C(5)-C(6)-C(7) | 120.42(12) |
| C(7)-C(6)-H(6) | 119.8 |
| C(2)-C(7)-C(8) | 124.83(10) |
| C(6)-C(7)-C(2) | 118.95(11) |
| C(6)-C(7)-C(8) | 116.22(11) |
| O(3)-C(8)-C(7) | 118.21(10) |
| O(3)-C(8)-C(9) | 122.38(11) |

| | |
|---|---|
| C(9)-C(8)-C(7) | 118.95(10) |
| C(10)-C(9)-C(8) | 117.80(10) |
| C(10)-C(9)-C(14) | 119.02(11) |
| C(14)-C(9)-C(8) | 123.16(10) |
| C(9)-C(10)-H(10) | 119.1 |
| C(11)-C(10)-C(9) | 121.76(11) |
| C(11)-C(10)-H(10) | 119.1 |
| C(10)-C(11)-H(11) | 119.9 |
| C(10)-C(11)-C(12) | 120.18(11) |
| C(12)-C(11)-H(11) | 119.9 |
| C(11)-C(12)-C(13) | 117.88(11) |
| C(11)-C(12)-C(21) | 121.80(11) |
| C(13)-C(12)-C(21) | 120.29(11) |
| C(12)-C(13)-H(13) | 118.5 |
| C(14)-C(13)-C(12) | 122.97(11) |
| C(14)-C(13)-H(13) | 118.5 |
| C(9)-C(14)-C(15) | 124.72(10) |
| C(13)-C(14)-C(9) | 118.13(10) |
| C(13)-C(14)-C(15) | 116.75(10) |
| C(16)-C(15)-C(14) | 124.11(11) |
| C(20)-C(15)-C(14) | 117.10(10) |
| C(20)-C(15)-C(16) | 118.75(11) |
| C(15)-C(16)-H(16) | 120.0 |
| C(17)-C(16)-C(15) | 120.05(12) |
| C(17)-C(16)-H(16) | 120.0 |
| C(16)-C(17)-H(17) | 119.6 |
| C(18)-C(17)-C(16) | 120.87(12) |
| C(18)-C(17)-H(17) | 119.6 |
| C(17)-C(18)-H(18) | 120.3 |
| C(17)-C(18)-C(19) | 119.43(12) |
| C(19)-C(18)-H(18) | 120.3 |
| C(18)-C(19)-H(19) | 120.0 |
| C(18)-C(19)-C(20) | 120.05(12) |
| C(20)-C(19)-H(19) | 120.0 |
| C(15)-C(20)-H(20) | 119.6 |
| C(19)-C(20)-C(15) | 120.76(11) |
| C(19)-C(20)-H(20) | 119.6 |
| C(22)-C(21)-C(12) | 120.86(11) |

| | |
|---|---|
| C(22)-C(21)-C(26) | 118.30(12) |
| C(26)-C(21)-C(12) | 120.84(12) |
| C(21)-C(22)-H(22) | 119.5 |
| C(23)-C(22)-C(21) | 120.92(12) |
| C(23)-C(22)-H(22) | 119.5 |
| C(22)-C(23)-H(23) | 119.9 |
| C(24)-C(23)-C(22) | 120.17(13) |
| C(24)-C(23)-H(23) | 119.9 |
| C(23)-C(24)-H(24) | 120.3 |
| C(25)-C(24)-C(23) | 119.50(12) |
| C(25)-C(24)-H(24) | 120.3 |
| C(24)-C(25)-H(25) | 119.6 |
| C(24)-C(25)-C(26) | 120.73(13) |
| C(26)-C(25)-H(25) | 119.6 |
| C(21)-C(26)-H(26) | 119.8 |
| C(25)-C(26)-C(21) | 120.38(13) |
| C(25)-C(26)-H(26) | 119.8 |

Symmetry transformations used to generate equivalent atoms:

**Table S10.** Anisotropic displacement parameters ($Å^2$x $10^3$) for **8**. The anisotropic displacement factor exponent takes the form: $-2p^2[ h^2 a^{*2}U^{11} + ... + 2 h k a^* b^* U^{12} ]$

| | $U^{11}$ | $U^{22}$ | $U^{33}$ | $U^{23}$ | $U^{13}$ | $U^{12}$ |
|---|---|---|---|---|---|---|
| O(1) | 24(1) | 23(1) | 25(1) | 8(1) | 9(1) | 6(1) |
| O(2) | 25(1) | 30(1) | 33(1) | 16(1) | 13(1) | 8(1) |
| O(3) | 23(1) | 30(1) | 39(1) | 13(1) | 2(1) | -4(1) |
| C(1) | 18(1) | 19(1) | 18(1) | 2(1) | 1(1) | -1(1) |
| C(2) | 17(1) | 17(1) | 20(1) | 1(1) | 0(1) | -1(1) |
| C(3) | 23(1) | 21(1) | 24(1) | 5(1) | 0(1) | 1(1) |
| C(4) | 26(1) | 23(1) | 30(1) | 4(1) | -4(1) | 6(1) |
| C(5) | 21(1) | 27(1) | 31(1) | -1(1) | 2(1) | 9(1) |
| C(6) | 21(1) | 29(1) | 24(1) | 1(1) | 5(1) | 3(1) |
| C(7) | 17(1) | 19(1) | 20(1) | 0(1) | 1(1) | 0(1) |
| C(8) | 20(1) | 21(1) | 20(1) | 3(1) | 6(1) | 2(1) |
| C(9) | 19(1) | 22(1) | 18(1) | 5(1) | 5(1) | 1(1) |
| C(10) | 23(1) | 21(1) | 24(1) | 6(1) | 5(1) | 3(1) |
| C(11) | 26(1) | 18(1) | 23(1) | 3(1) | 5(1) | -2(1) |
| C(12) | 22(1) | 24(1) | 18(1) | 6(1) | 4(1) | -2(1) |
| C(13) | 22(1) | 22(1) | 21(1) | 8(1) | 3(1) | 1(1) |
| C(14) | 22(1) | 20(1) | 19(1) | 6(1) | 5(1) | -1(1) |
| C(15) | 21(1) | 20(1) | 21(1) | 7(1) | 0(1) | 0(1) |
| C(16) | 24(1) | 25(1) | 34(1) | 10(1) | 5(1) | 0(1) |
| C(17) | 27(1) | 24(1) | 44(1) | 13(1) | 0(1) | -5(1) |
| C(18) | 33(1) | 18(1) | 35(1) | 5(1) | -5(1) | 1(1) |
| C(19) | 31(1) | 25(1) | 27(1) | 6(1) | 2(1) | 7(1) |
| C(20) | 22(1) | 22(1) | 24(1) | 9(1) | 1(1) | 1(1) |
| C(21) | 25(1) | 26(1) | 19(1) | 8(1) | 2(1) | -5(1) |
| C(22) | 26(1) | 31(1) | 24(1) | 10(1) | 2(1) | -3(1) |
| C(23) | 25(1) | 43(1) | 33(1) | 21(1) | -1(1) | -4(1) |
| C(24) | 31(1) | 47(1) | 29(1) | 19(1) | -9(1) | -14(1) |
| C(25) | 44(1) | 37(1) | 25(1) | 3(1) | -4(1) | -13(1) |
| C(26) | 34(1) | 31(1) | 26(1) | 4(1) | 2(1) | -4(1) |

**Table S11**. Hydrogen coordinates ( x $10^4$) and isotropic displacement parameters (Å$^2$x $10^3$) for **8**.

| | x | y | z | U(eq) |
|---|---|---|---|---|
| H(2) | 4582 | 6024 | -286 | 40 |
| H(3) | 692 | 7692 | -231 | 29 |
| H(4) | -2096 | 8764 | 233 | 34 |
| H(5) | -3329 | 8392 | 1520 | 35 |
| H(6) | -1880 | 6878 | 2305 | 32 |
| H(10) | 2904 | 8056 | 3017 | 27 |
| H(11) | 5839 | 8910 | 4118 | 28 |
| H(13) | 7140 | 4875 | 3765 | 26 |
| H(16) | 1772 | 2926 | 2997 | 33 |
| H(17) | 1500 | 493 | 2178 | 39 |
| H(18) | 3704 | -505 | 1174 | 37 |
| H(19) | 6304 | 936 | 1049 | 34 |
| H(20) | 6620 | 3370 | 1883 | 28 |
| H(22) | 10217 | 5836 | 4108 | 32 |
| H(23) | 13233 | 6649 | 5164 | 39 |
| H(24) | 13546 | 8765 | 6467 | 44 |
| H(25) | 10856 | 10093 | 6682 | 47 |
| H(26) | 7844 | 9314 | 5617 | 38 |

**Table S12**. Torsion angles [°] for **8**.

| | |
|---|---|
| O(1)-C(1)-C(2)-C(3) | -162.89(11) |
| O(1)-C(1)-C(2)-C(7) | 13.38(16) |
| O(2)-C(1)-C(2)-C(3) | 17.21(15) |
| O(2)-C(1)-C(2)-C(7) | -166.52(10) |
| O(3)-C(8)-C(9)-C(10) | -141.89(12) |
| O(3)-C(8)-C(9)-C(14) | 36.50(17) |
| C(1)-C(2)-C(3)-C(4) | 177.84(10) |
| C(1)-C(2)-C(7)-C(6) | -176.78(10) |
| C(1)-C(2)-C(7)-C(8) | 2.26(17) |
| C(2)-C(3)-C(4)-C(5) | -0.63(18) |
| C(2)-C(7)-C(8)-O(3) | -121.11(13) |
| C(2)-C(7)-C(8)-C(9) | 66.53(15) |
| C(3)-C(2)-C(7)-C(6) | -0.52(16) |
| C(3)-C(2)-C(7)-C(8) | 178.52(11) |
| C(3)-C(4)-C(5)-C(6) | -1.37(19) |
| C(4)-C(5)-C(6)-C(7) | 2.43(19) |
| C(5)-C(6)-C(7)-C(2) | -1.46(17) |
| C(5)-C(6)-C(7)-C(8) | 179.41(11) |
| C(6)-C(7)-C(8)-O(3) | 57.96(15) |
| C(6)-C(7)-C(8)-C(9) | -114.40(12) |
| C(7)-C(2)-C(3)-C(4) | 1.57(17) |
| C(7)-C(8)-C(9)-C(10) | 30.14(15) |
| C(7)-C(8)-C(9)-C(14) | -151.47(11) |
| C(8)-C(9)-C(10)-C(11) | 176.32(11) |
| C(8)-C(9)-C(14)-C(13) | -177.61(10) |
| C(8)-C(9)-C(14)-C(15) | 9.87(18) |
| C(9)-C(10)-C(11)-C(12) | 1.45(18) |
| C(9)-C(14)-C(15)-C(16) | -68.04(17) |
| C(9)-C(14)-C(15)-C(20) | 114.30(13) |
| C(10)-C(9)-C(14)-C(13) | 0.76(17) |
| C(10)-C(9)-C(14)-C(15) | -171.76(11) |
| C(10)-C(11)-C(12)-C(13) | 0.60(17) |
| C(10)-C(11)-C(12)-C(21) | 178.91(11) |
| C(11)-C(12)-C(13)-C(14) | -1.99(18) |
| C(11)-C(12)-C(21)-C(22) | -147.27(12) |
| C(11)-C(12)-C(21)-C(26) | 32.29(18) |

| | |
|---|---|
| C(12)-C(13)-C(14)-C(9) | 1.31(17) |
| C(12)-C(13)-C(14)-C(15) | 174.43(11) |
| C(12)-C(21)-C(22)-C(23) | 179.52(11) |
| C(12)-C(21)-C(26)-C(25) | 179.94(12) |
| C(13)-C(12)-C(21)-C(22) | 31.00(17) |
| C(13)-C(12)-C(21)-C(26) | -149.43(12) |
| C(13)-C(14)-C(15)-C(16) | 119.35(13) |
| C(13)-C(14)-C(15)-C(20) | -58.32(15) |
| C(14)-C(9)-C(10)-C(11) | -2.13(18) |
| C(14)-C(15)-C(16)-C(17) | 179.70(12) |
| C(14)-C(15)-C(20)-C(19) | -179.03(11) |
| C(15)-C(16)-C(17)-C(18) | 0.2(2) |
| C(16)-C(15)-C(20)-C(19) | 3.18(18) |
| C(16)-C(17)-C(18)-C(19) | 1.8(2) |
| C(17)-C(18)-C(19)-C(20) | -1.32(19) |
| C(18)-C(19)-C(20)-C(15) | -1.20(19) |
| C(20)-C(15)-C(16)-C(17) | -2.67(19) |
| C(21)-C(12)-C(13)-C(14) | 179.67(11) |
| C(21)-C(22)-C(23)-C(24) | 0.7(2) |
| C(22)-C(21)-C(26)-C(25) | -0.5(2) |
| C(22)-C(23)-C(24)-C(25) | -0.9(2) |
| C(23)-C(24)-C(25)-C(26) | 0.3(2) |
| C(24)-C(25)-C(26)-C(21) | 0.3(2) |
| C(26)-C(21)-C(22)-C(23) | -0.05(19) |

Symmetry transformations used to generate equivalent atoms:

## 5. Copies of NMR Spectra of Products

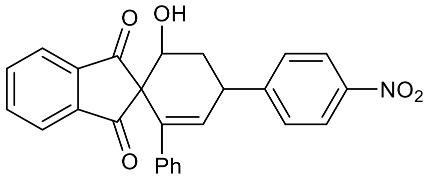


($^1$H NMR, 400MHz, $CDCl_3$)

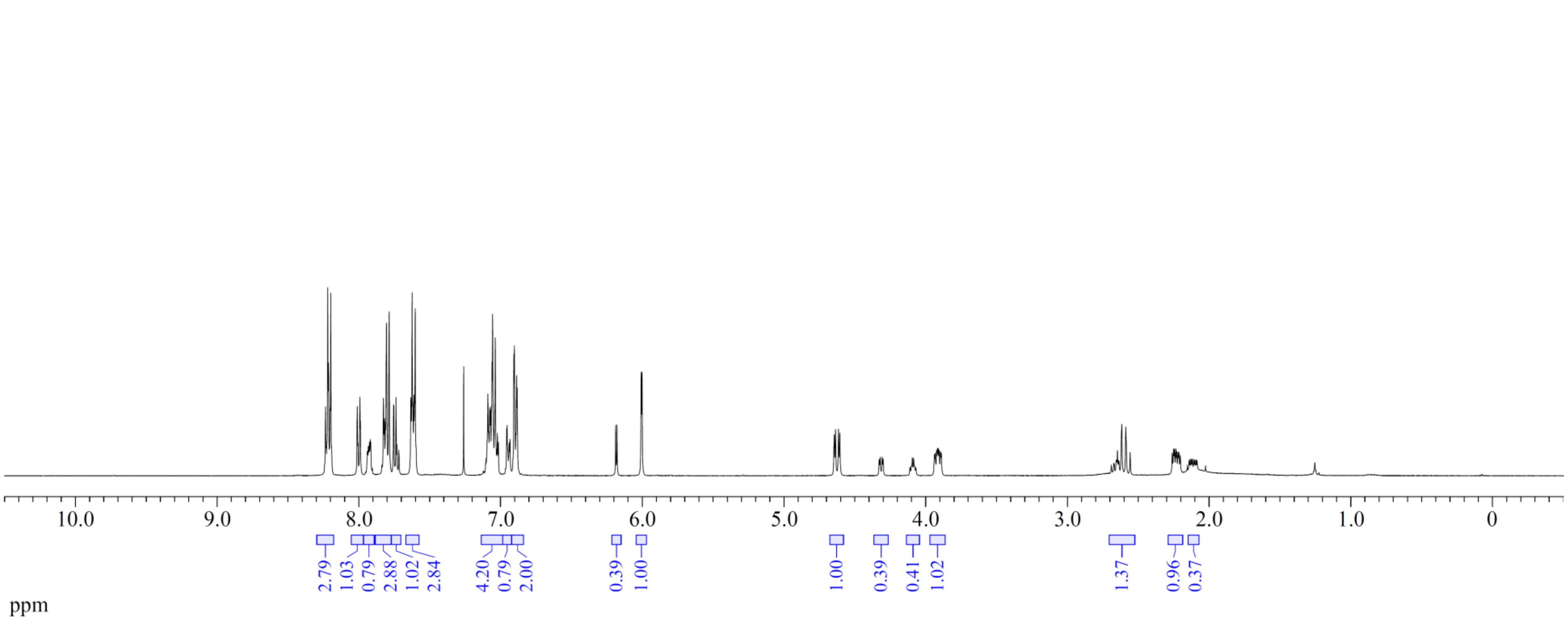

J-A E 4-NO2_Carbon_copy2-1-2.jdf

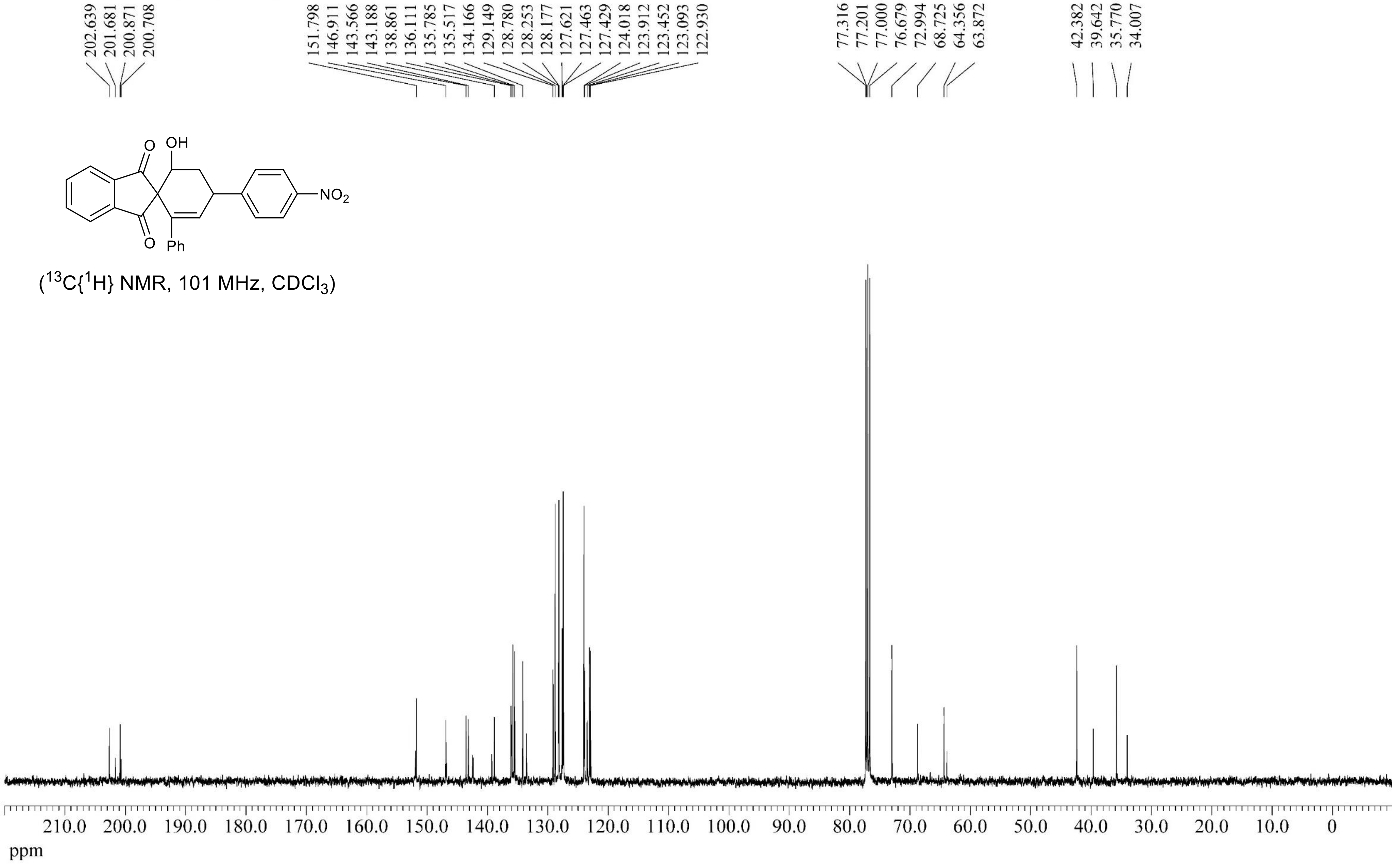


($^{13}C\{^1H\}$ NMR, 101 MHz, $CDCl_3$)

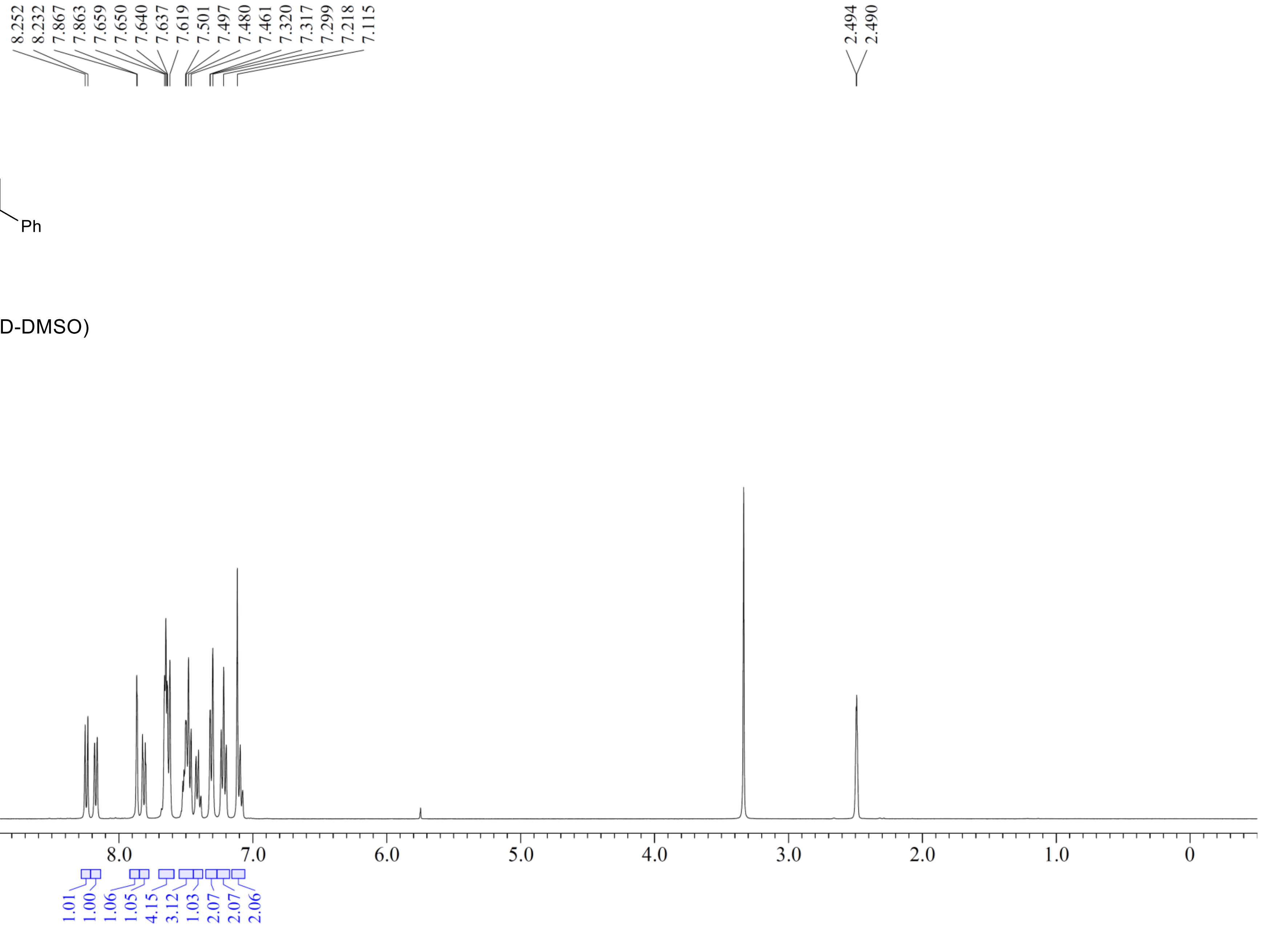


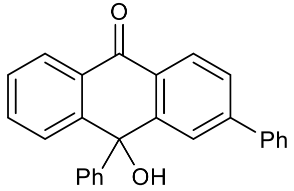


**5a**

($^1$H NMR, 400MHz, D-DMSO)

J-F model_carbon_copy1-1-2.jdf

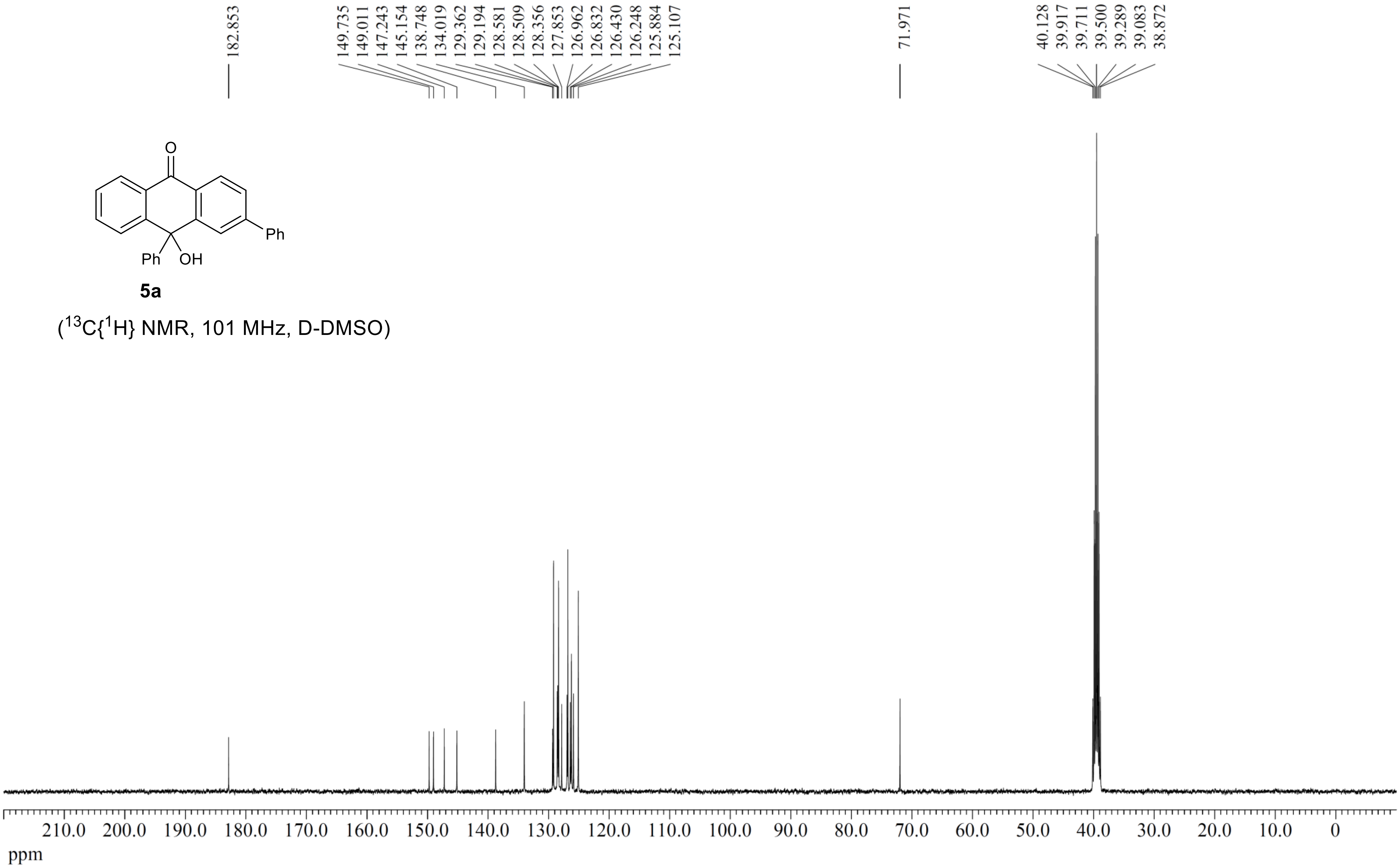


**5a**

($^{13}C\{^{1}H\}$ NMR, 101 MHz, D-DMSO)

J-F E 2-NO2_Proton-3-2.jdf

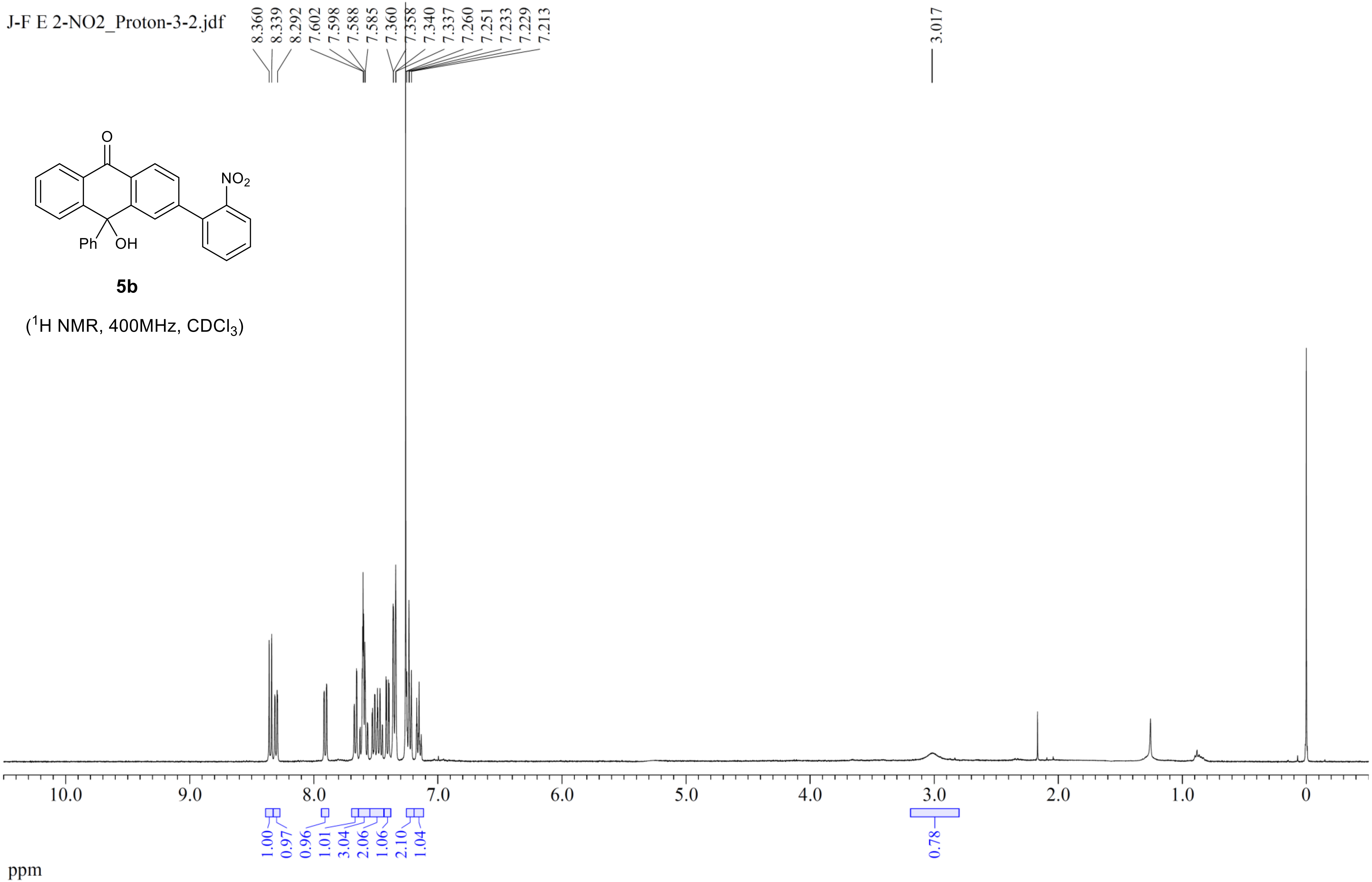


**5b**

($^1$H NMR, 400MHz, $CDCl_3$)

J-F E 2-NO2_Carbon_copy4-1-2.jdf

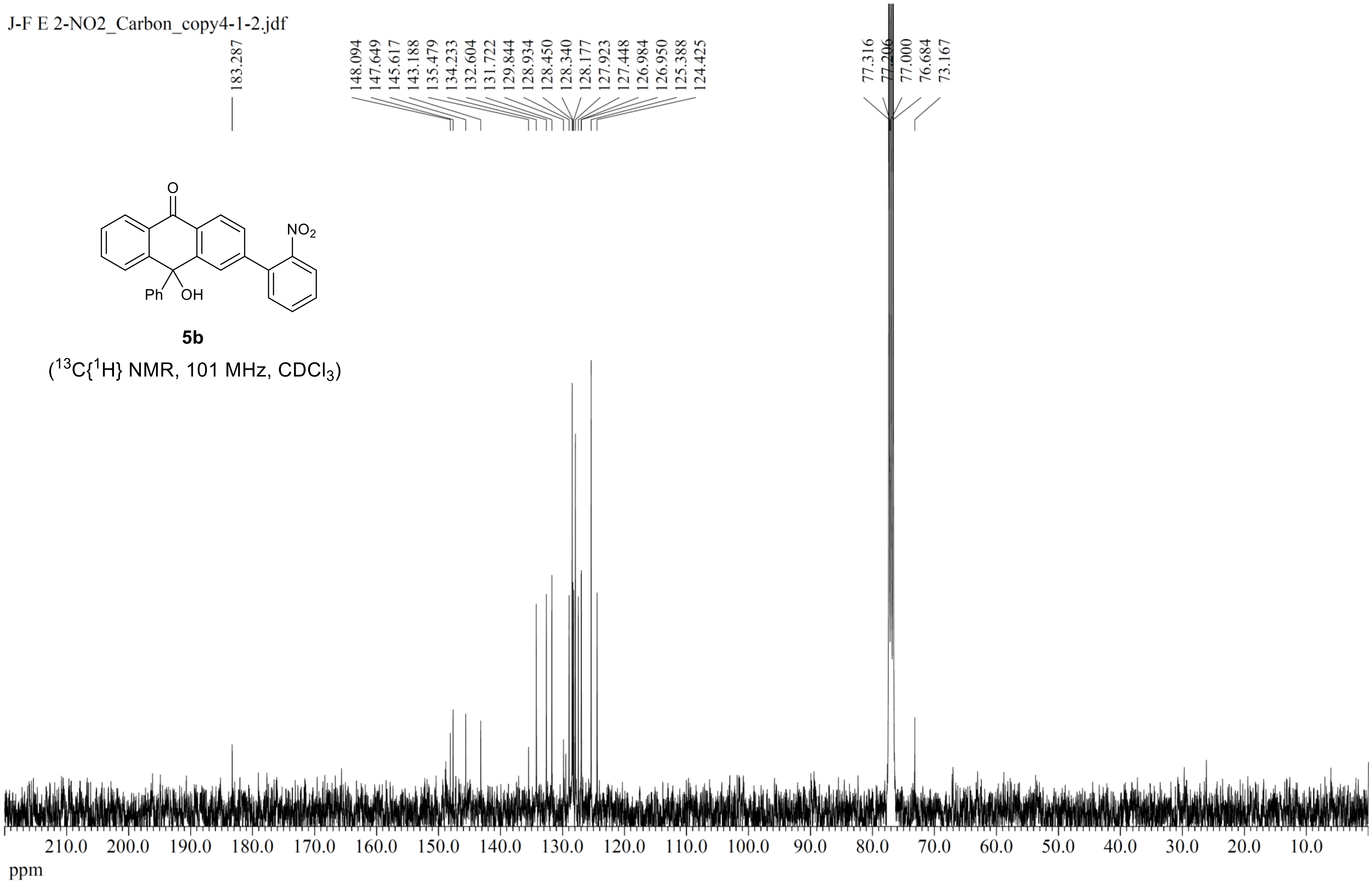


**5b**

($^{13}C\{^1H\}$ NMR, 101 MHz, $CDCl_3$)

J-F E 2-OMe_Proton-4-2.jdf

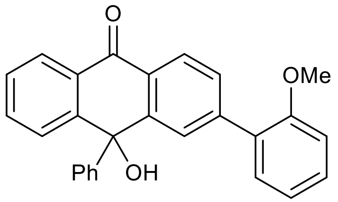


**5c**

($^{1}$H NMR, 400MHz, $CDCl_3$)

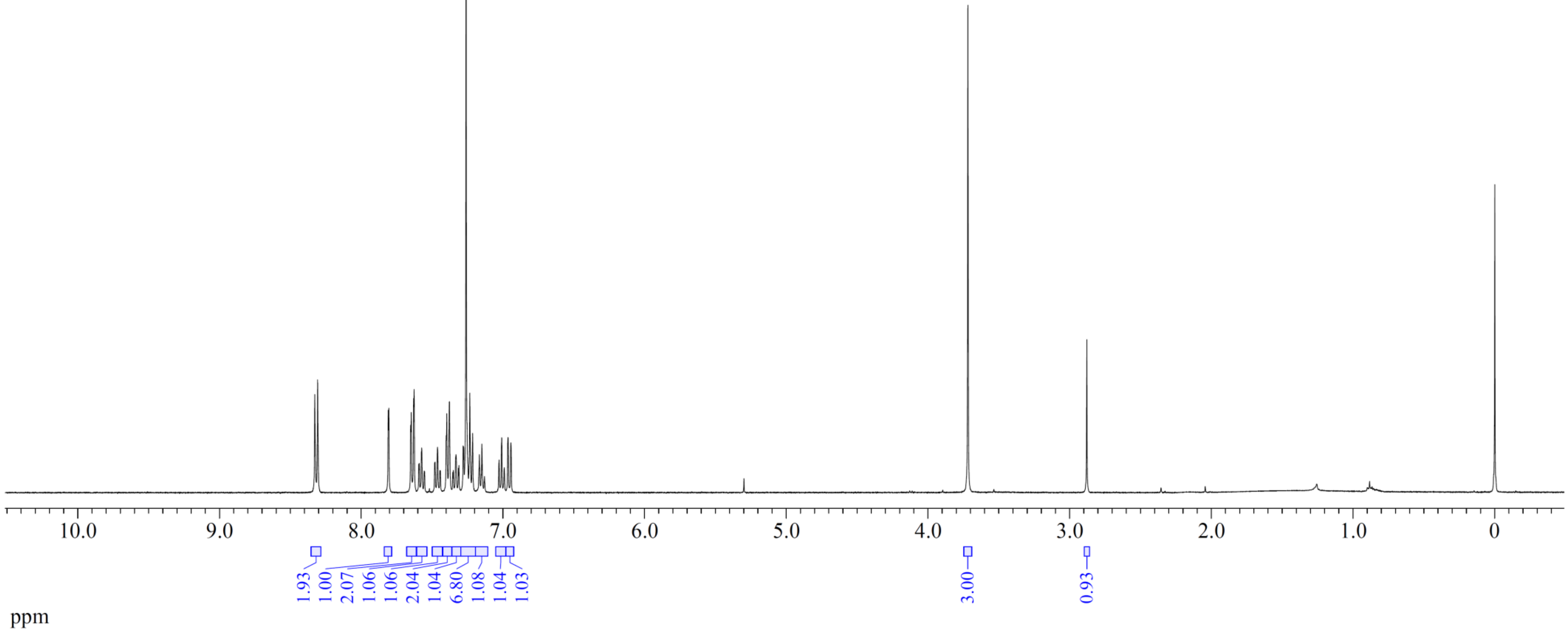

J-F E 2-OMe_Carbon_copy2-1-2.jdf

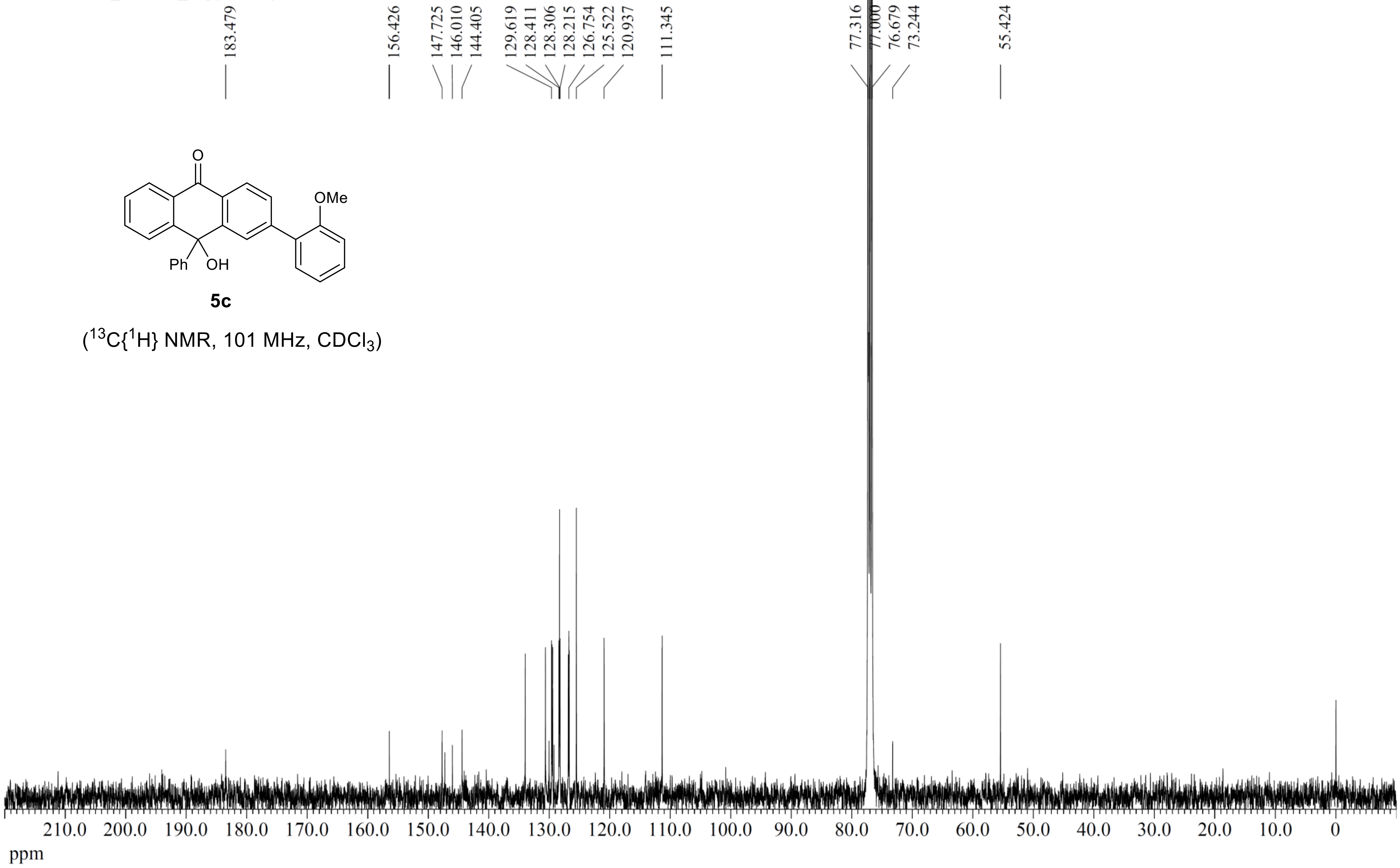


**5c**

($^{13}C\{^{1}H\}$ NMR, 101 MHz, $CDCl_3$)

J-F E 3-Br_Proton-1-2.jdf

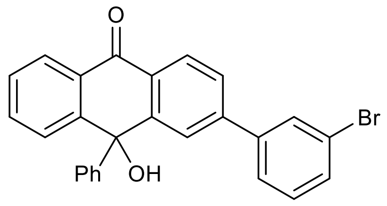


**5d**

($^1$H NMR, 400MHz, CDCl$_3$)

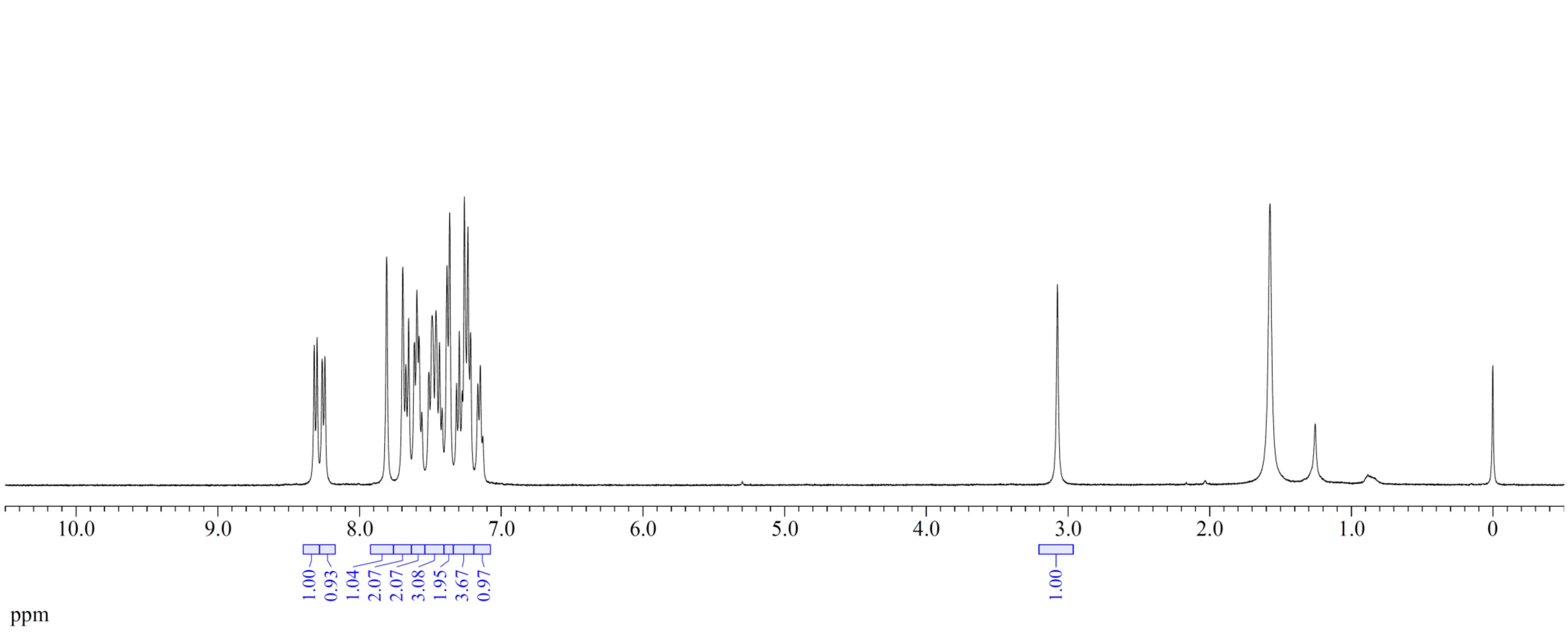

J-F E 3-Br_Carbon_copy1-1-2.jdf

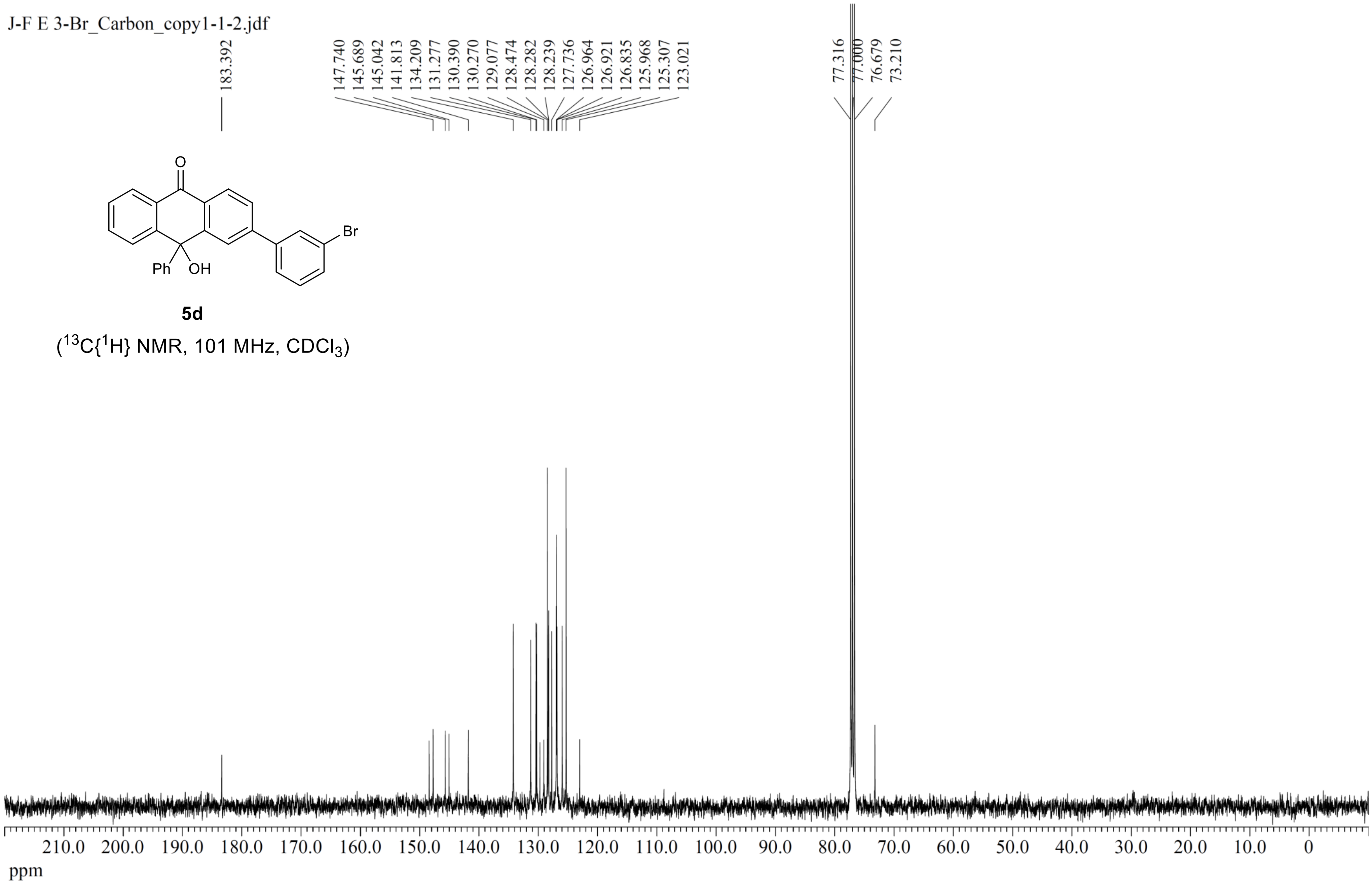


**5d**

($^{13}C\{^1H\}$ NMR, 101 MHz, $CDCl_3$)

J-F E 4-F_Proton-2-2.jdf

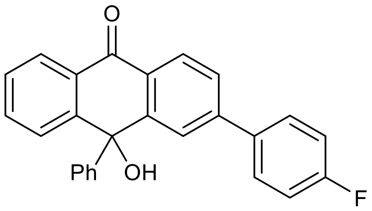

**5e**

($^1$H NMR, 400MHz, $CDCl_3$)

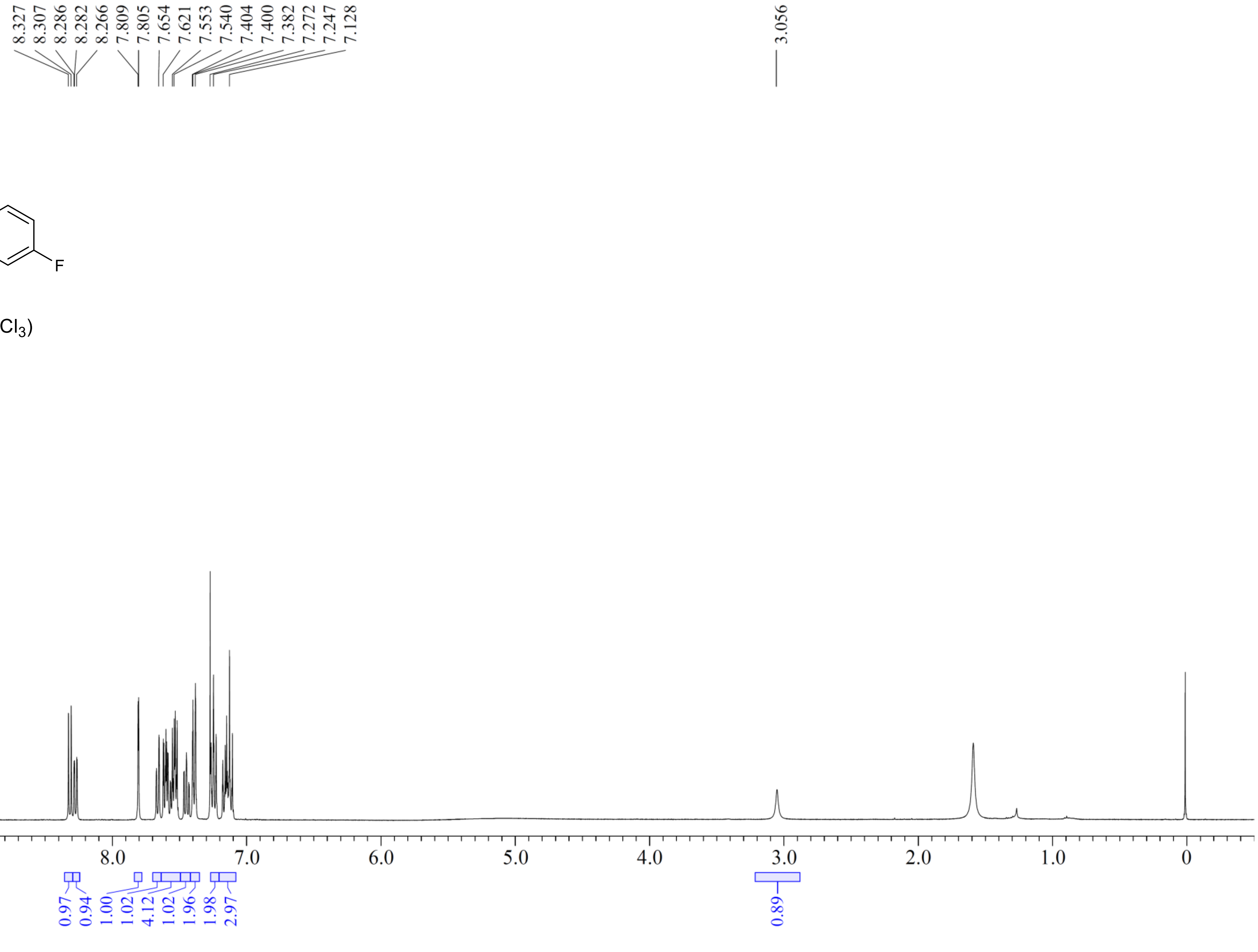

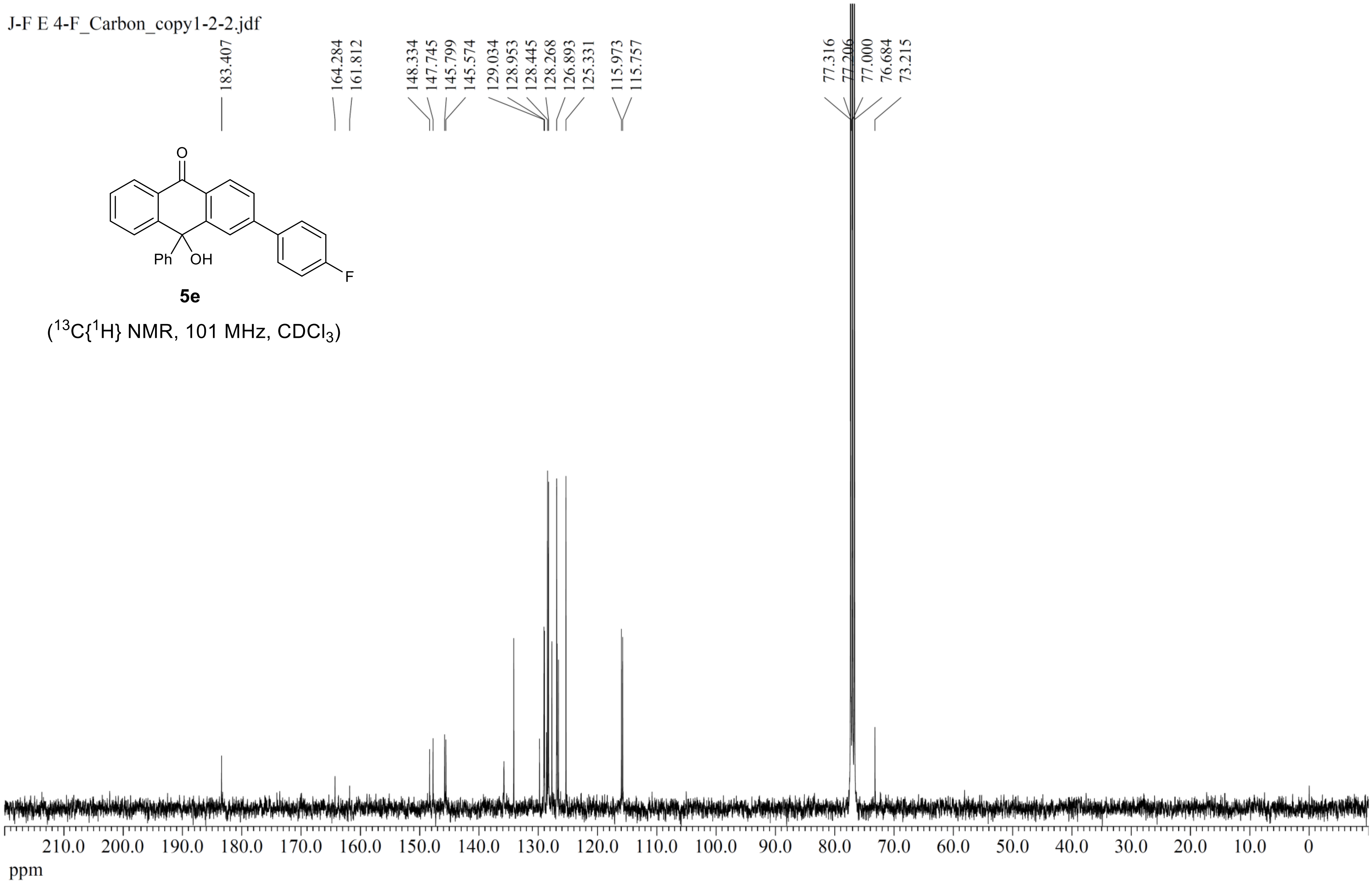

J-F E 4-F_Carbon_copy1-2-2.jdf
183.407
164.284
161.812
148.334
147.745
145.799
145.574
129.034
128.953
128.445
128.268
126.893
125.331
115.973
115.757
77.316
77.206
77.000
76.684
73.215
O
Ph
OH
F
5e
($^{13}$C{$^{1}$H} NMR, 101 MHz, $CDCl_3$)
210.0
200.0
190.0
180.0
170.0
160.0
150.0
140.0
130.0
120.0
110.0
100.0
90.0
80.0
70.0
60.0
50.0
40.0
30.0
20.0
10.0
0
ppm

J-F E 4-F_fluorine_1h_dec_copy1-1-2.jdf

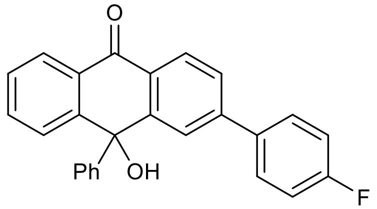


**5e**

($^{19}$F NMR, 376MHz, $CDCl_3$)

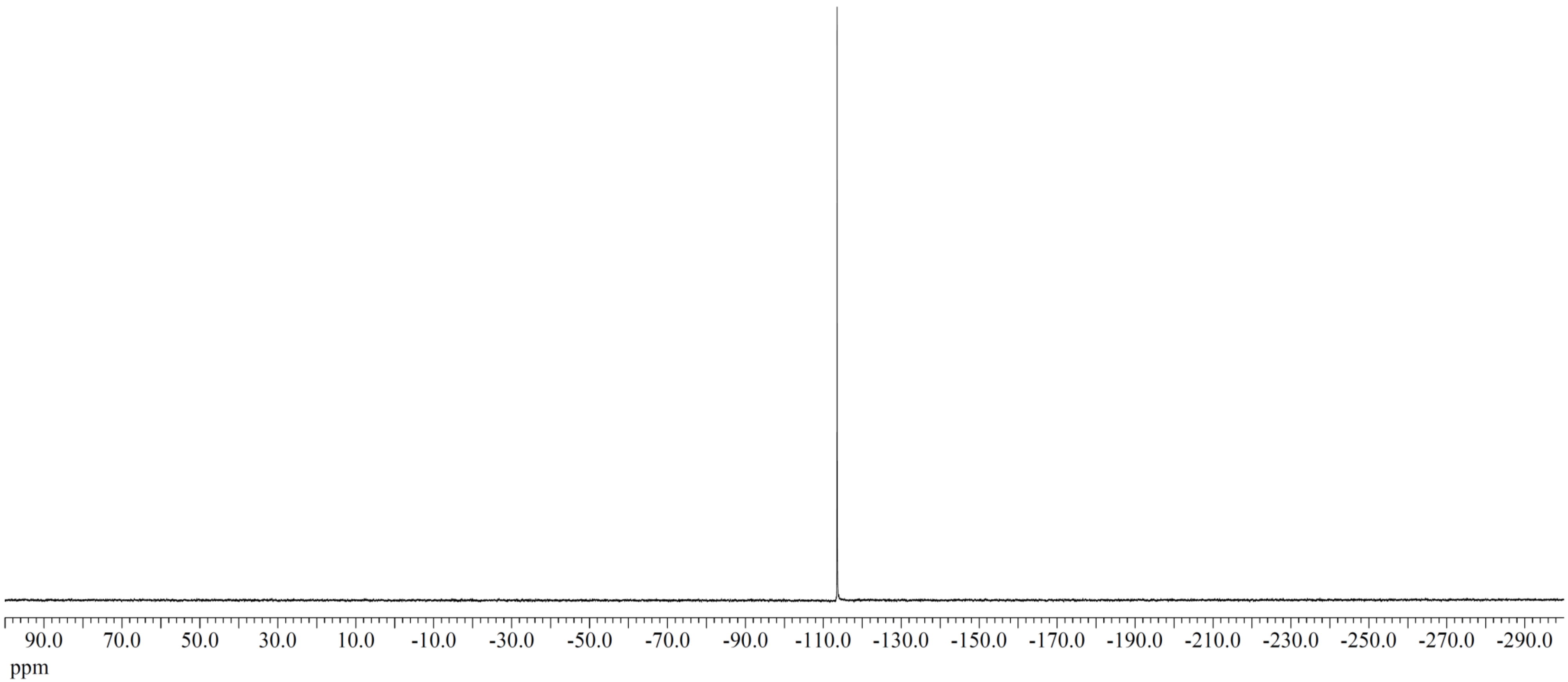

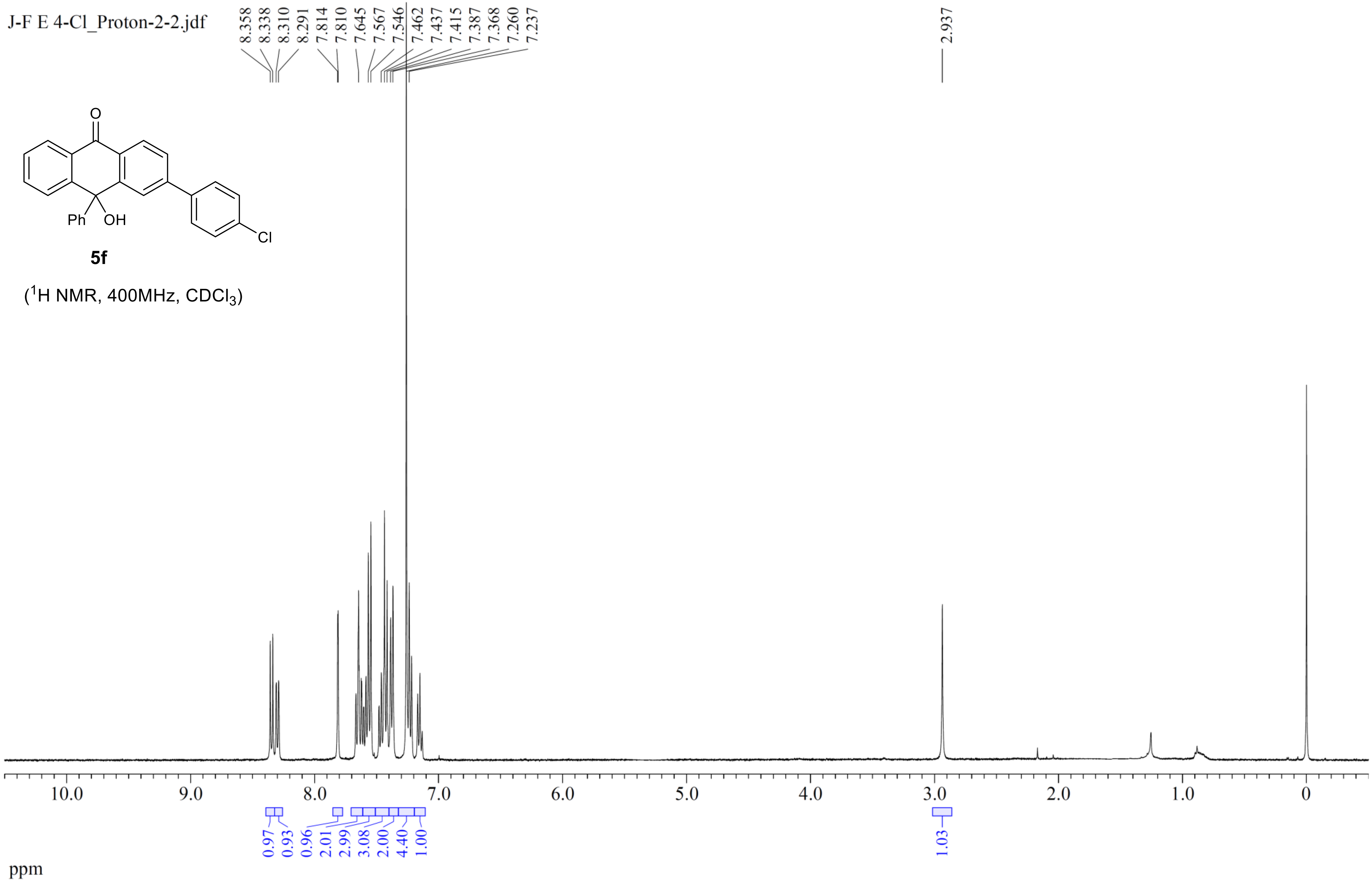

J-F E 4-Cl_Proton-2-2.jdf
8.358
8.338
8.310
8.291
7.814
7.810
7.645
7.567
7.546
7.462
7.437
7.415
7.387
7.368
7.260
7.237
2.937
O
Ph
OH
Cl
5f
(1H NMR, 400MHz, CDCl3)
10.0
9.0
8.0
7.0
6.0
5.0
4.0
3.0
2.0
1.0
0
0.97
0.93
0.96
2.01
2.99
3.08
2.00
4.40
1.00
1.03
ppm

J-F E 4-Cl_Carbon_copy3-1-2.jdf

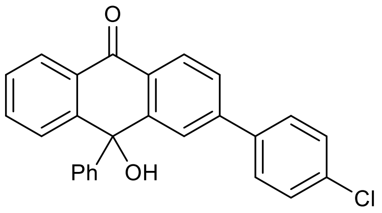

**5f**

($^{13}C\{^{1}H\}$ NMR, 101 MHz, $CDCl_3$)

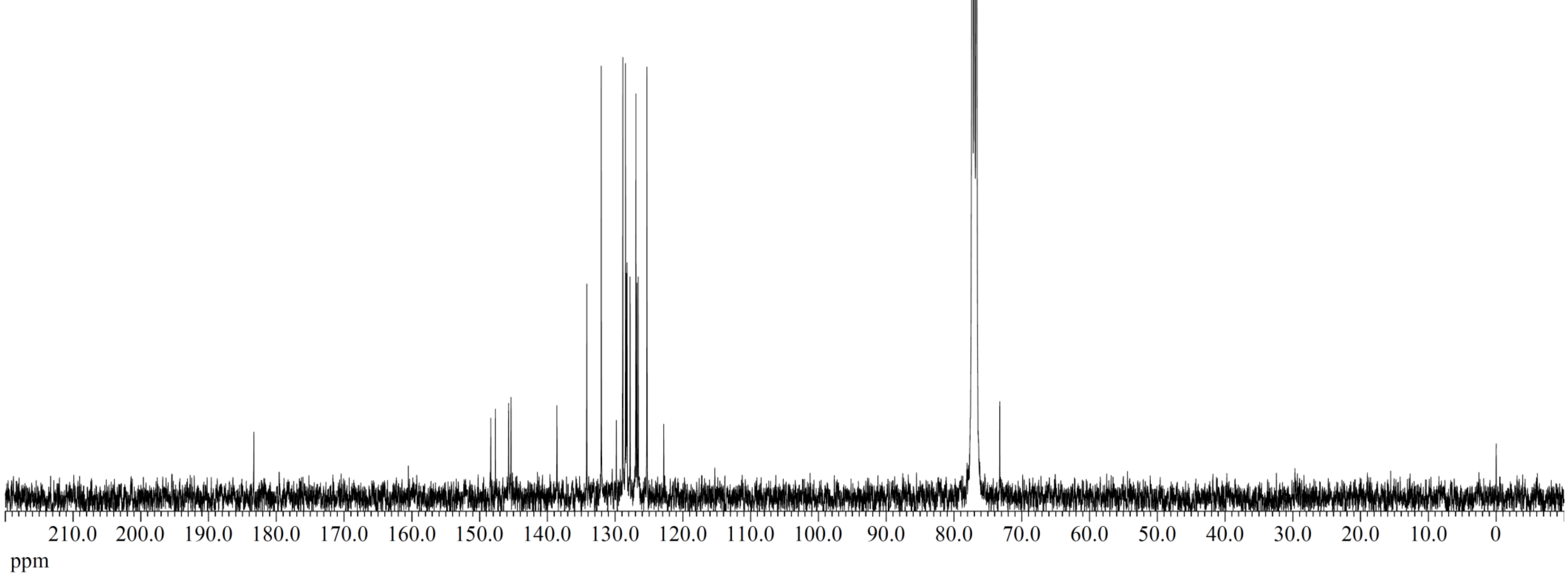

J-F E 4-Br_Proton-2-2.jdf

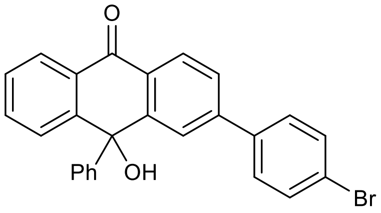


**5g**

($^{1}$H NMR, 400MHz, $CDCl_3$)

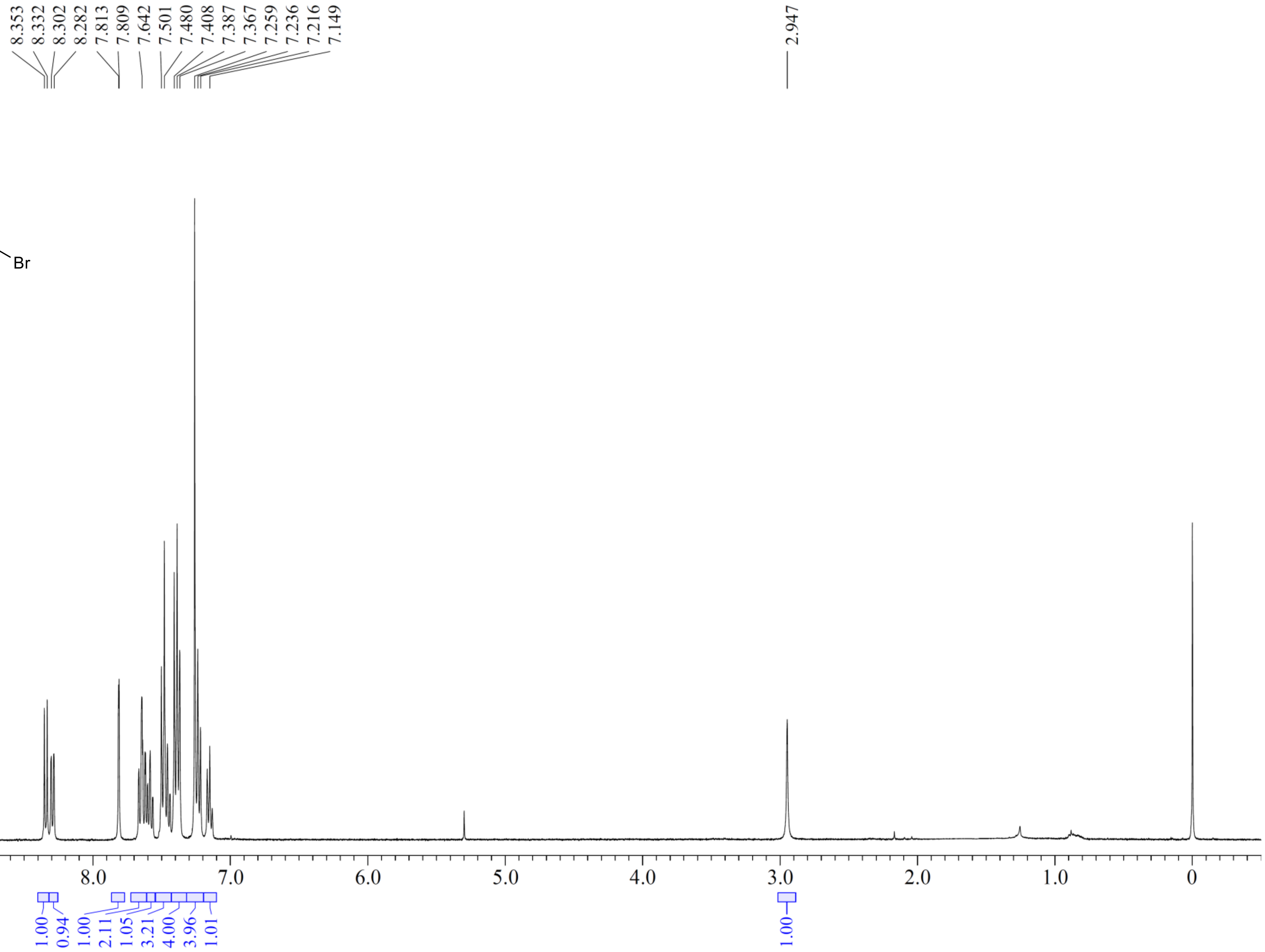


ppm

J-F E 4-Br_Carbon_copy3-1-2.jdf

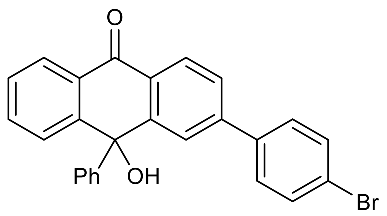

**5g**

($^{13}C\{^{1}H\}$ NMR, 101 MHz, $CDCl_3$)

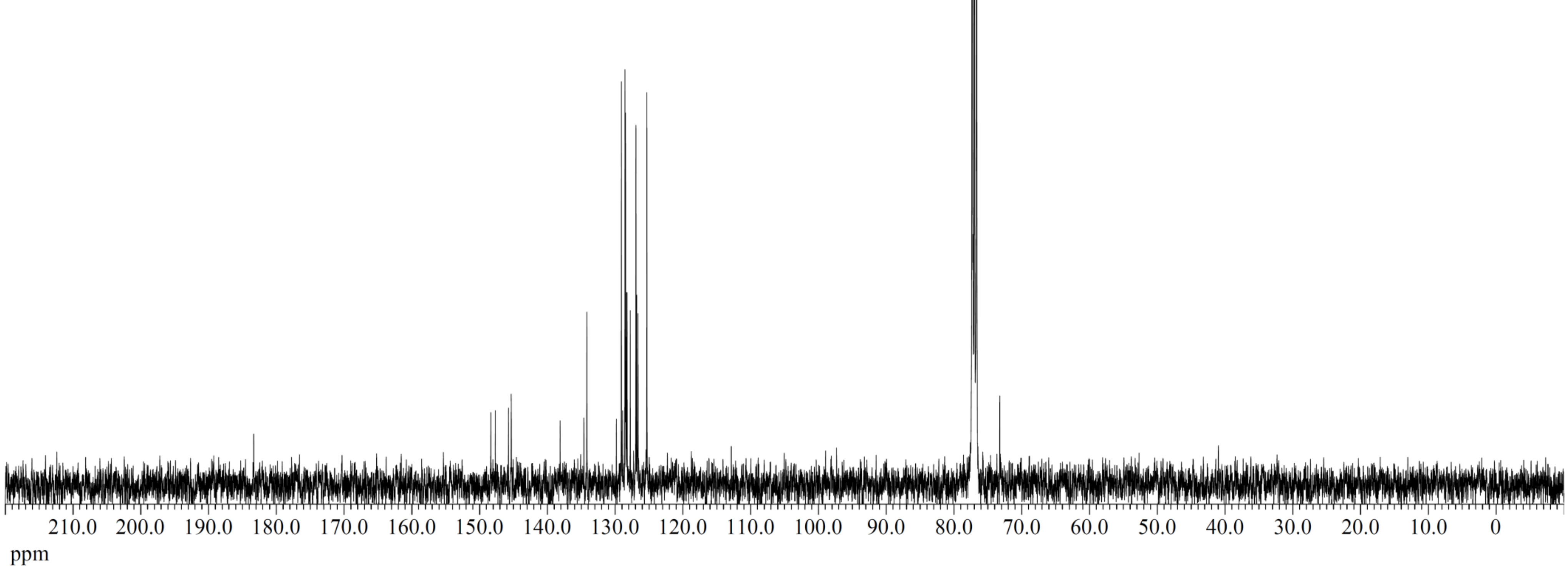

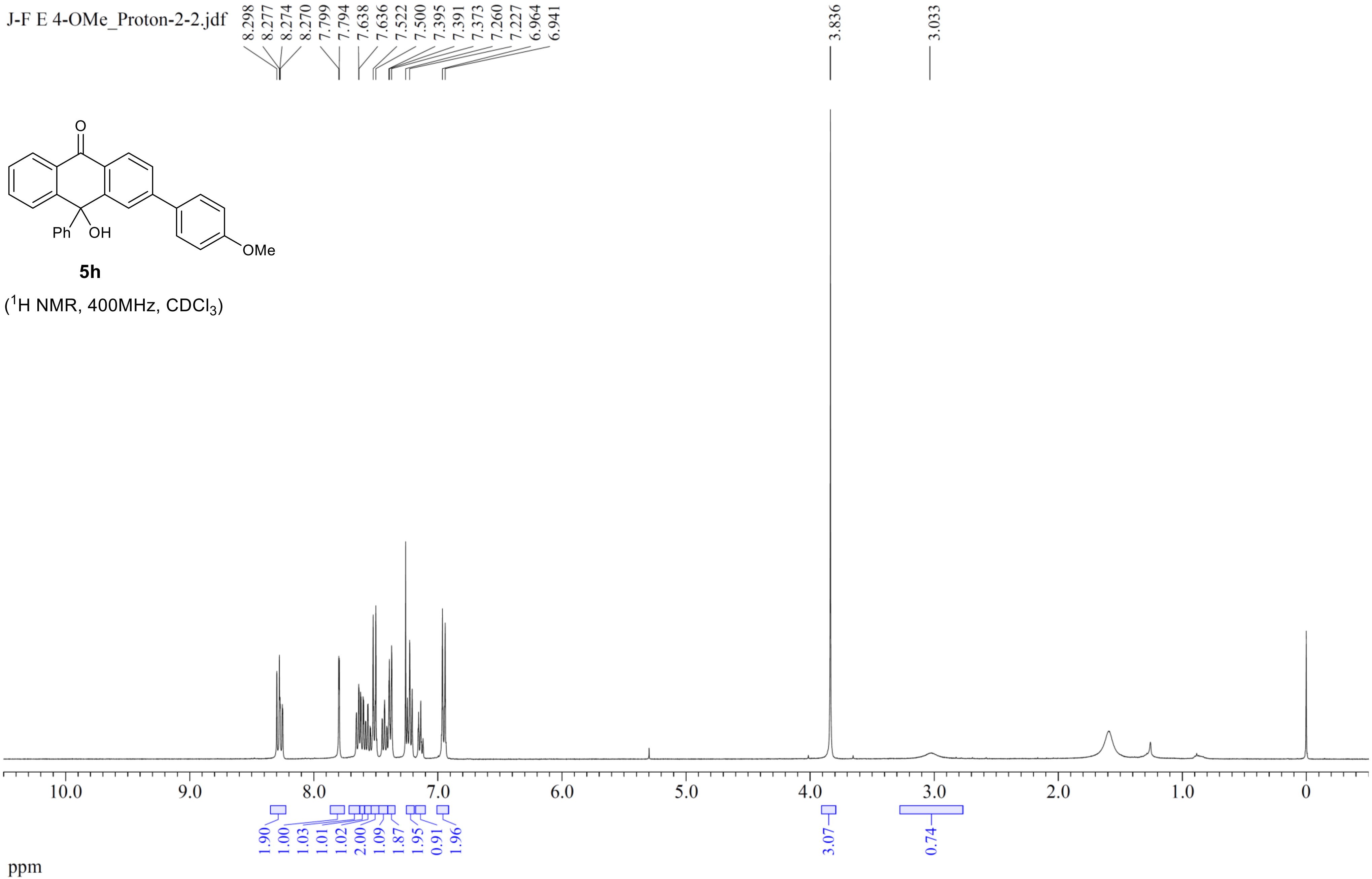
J-F E 4-OMe_Proton-2-2.jdf
8.298
8.277
8.274
8.270
7.799
7.794
7.638
7.636
7.522
7.500
7.395
7.391
7.373
7.260
7.227
6.964
6.941
3.836
3.033
O
Ph
OH
OMe
5h
($^1$H NMR, 400MHz, $CDCl_3$)
10.0
9.0
8.0
7.0
6.0
5.0
4.0
3.0
2.0
1.0
0
1.90
1.00
1.03
1.01
1.02
2.00
1.09
1.87
1.95
0.91
1.96
3.07
0.74
ppm

J-F E 4-OMe_Carbon_copy6-1-2.jdf

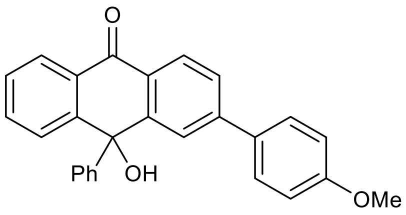

**5h**

($^{13}C\{^{1}H\}$ NMR, 101 MHz, $CDCl_3$)

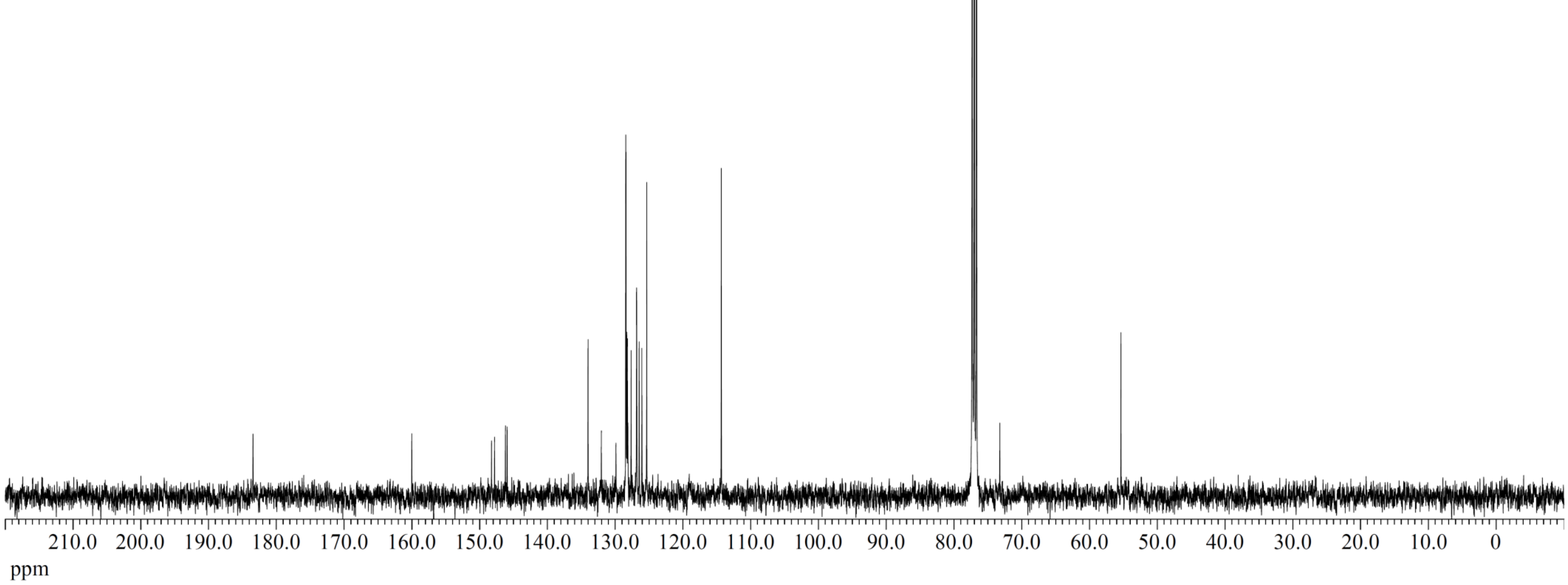

J-F E 4-NO2_Proton-2-2.jdf

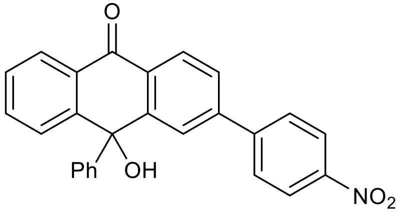


**5i**

($^1$H NMR, 400MHz, $CDCl_3$)

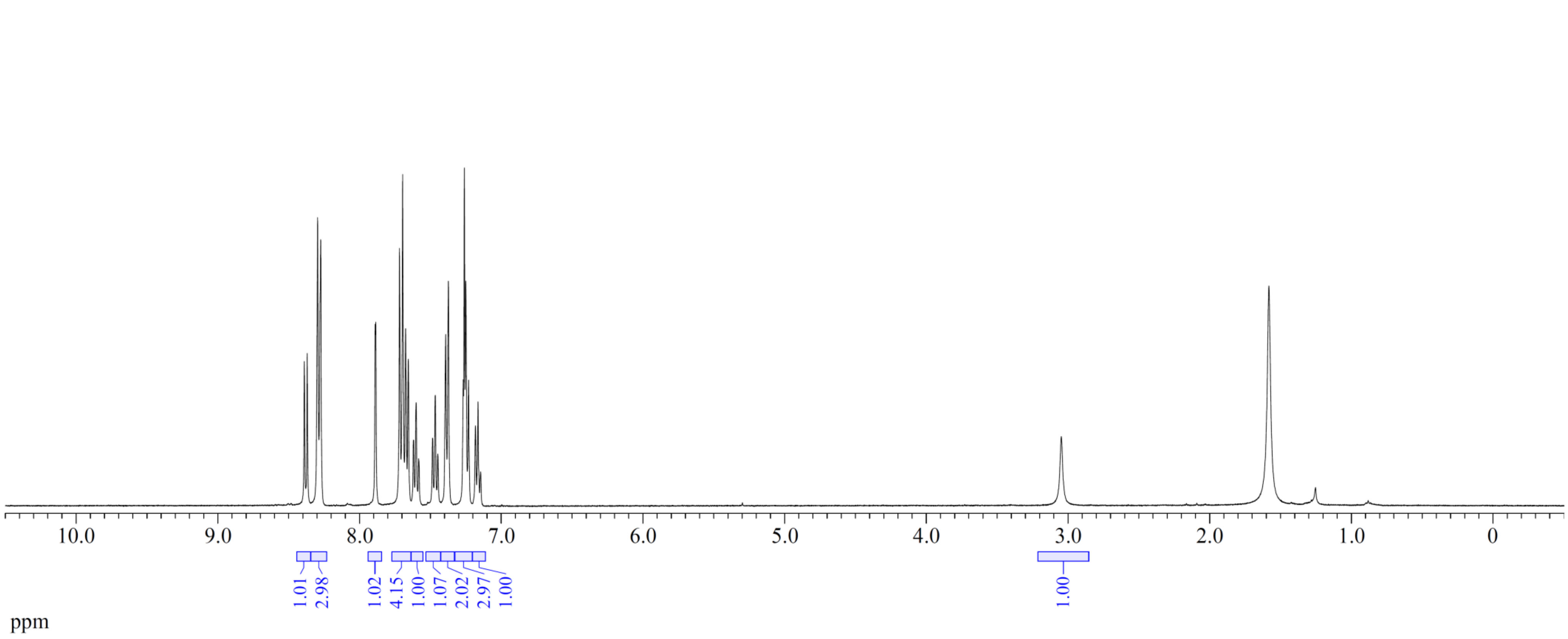

J-F E 4-NO2_Carbon_copy2-2-2.jdf

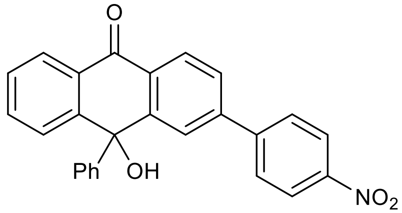

**5i**

($^{13}C\{^{1}H\}$ NMR, 101 MHz, $CDCl_3$)

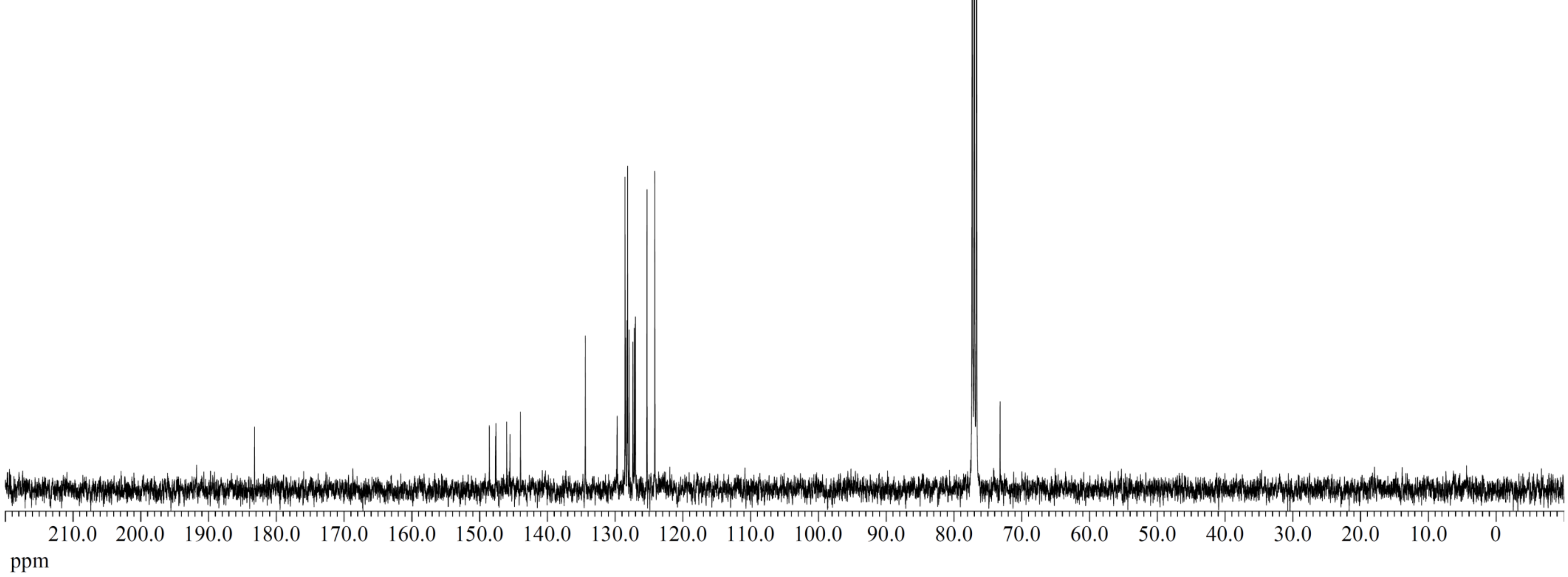

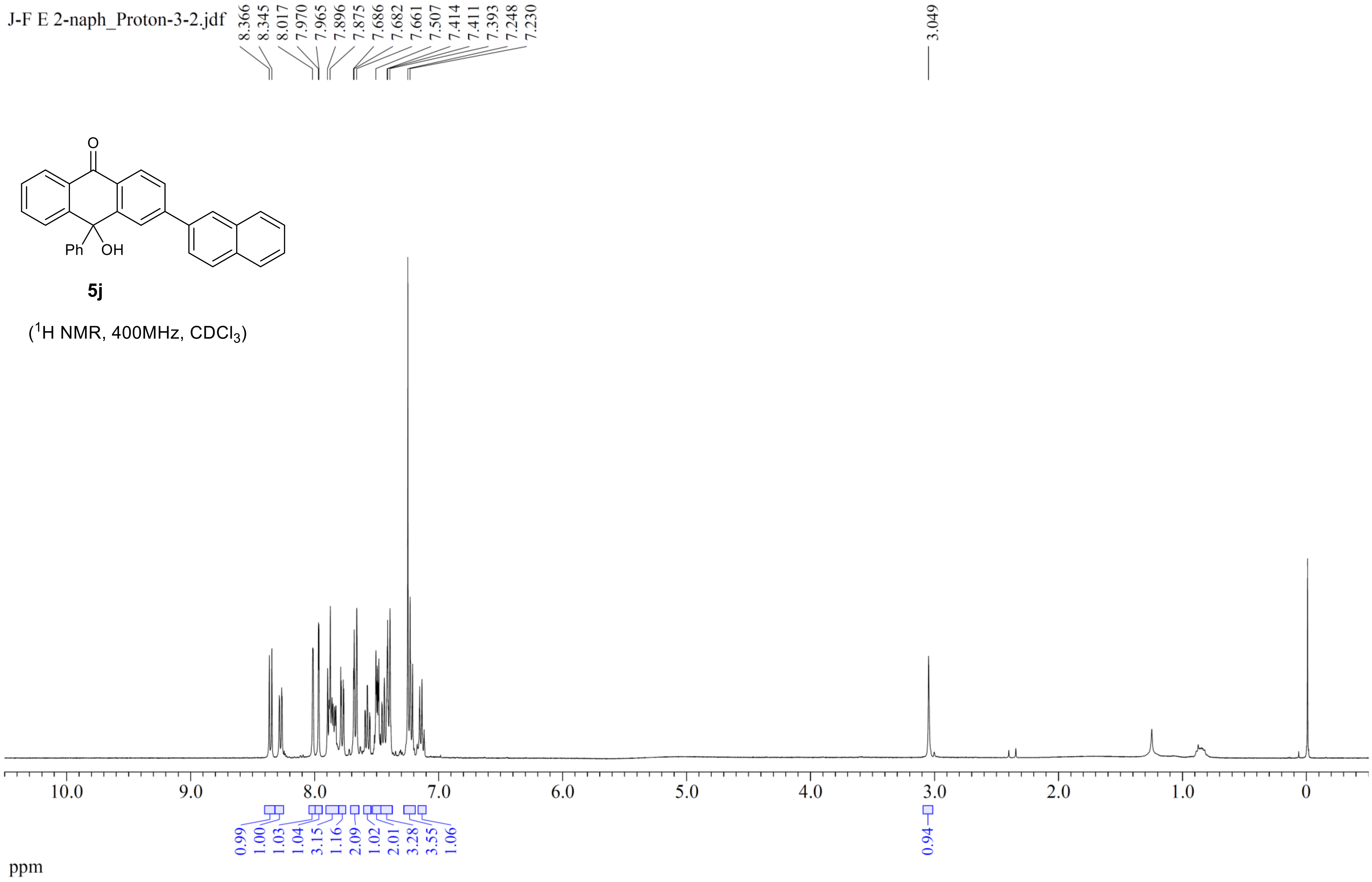


**5j**

($^{1}H$ NMR, 400MHz, $CDCl_3$)

J-F E 2-naph_Carbon_copy6-1-2.jdf

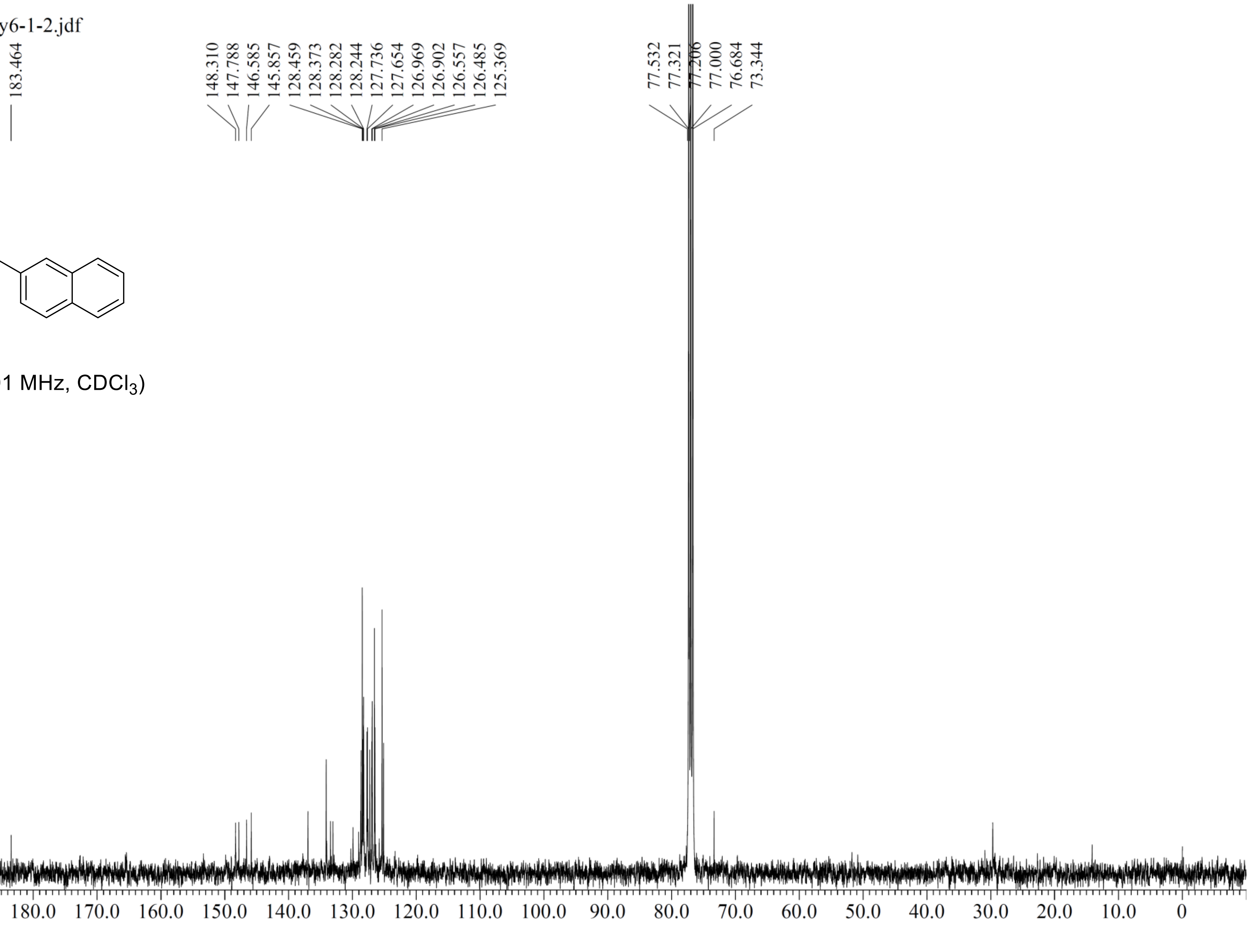


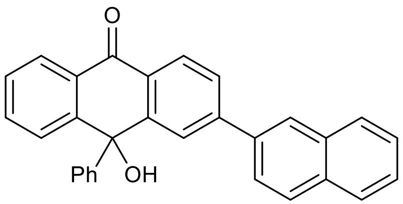


**5j**

($^{13}C\{^{1}H\}$ NMR, 101 MHz, $CDCl_3$)

J-F E 2-thienyl D-DMSO_Proton-1-2.jdf

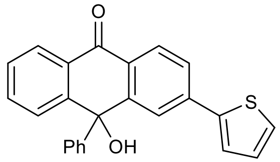


**5k**

($^1$H NMR, 400MHz, D-DMSO)

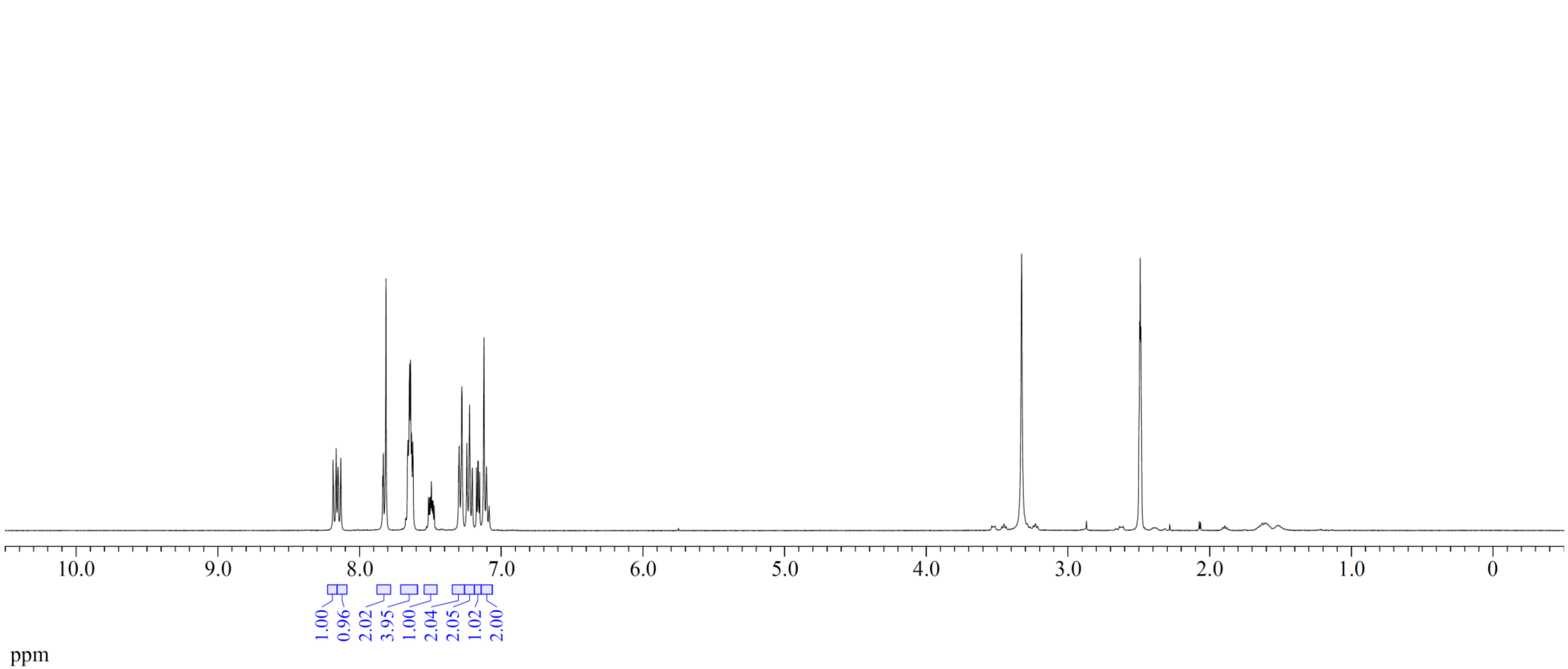

J-F E 2-thienyl D-DMSO_Carbon_copy2-2-2.jdf

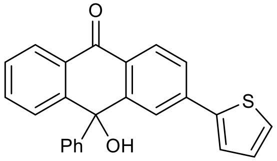

**5k**

($^{13}C\{^{1}H\}$ NMR, 101 MHz, D-DMSO)

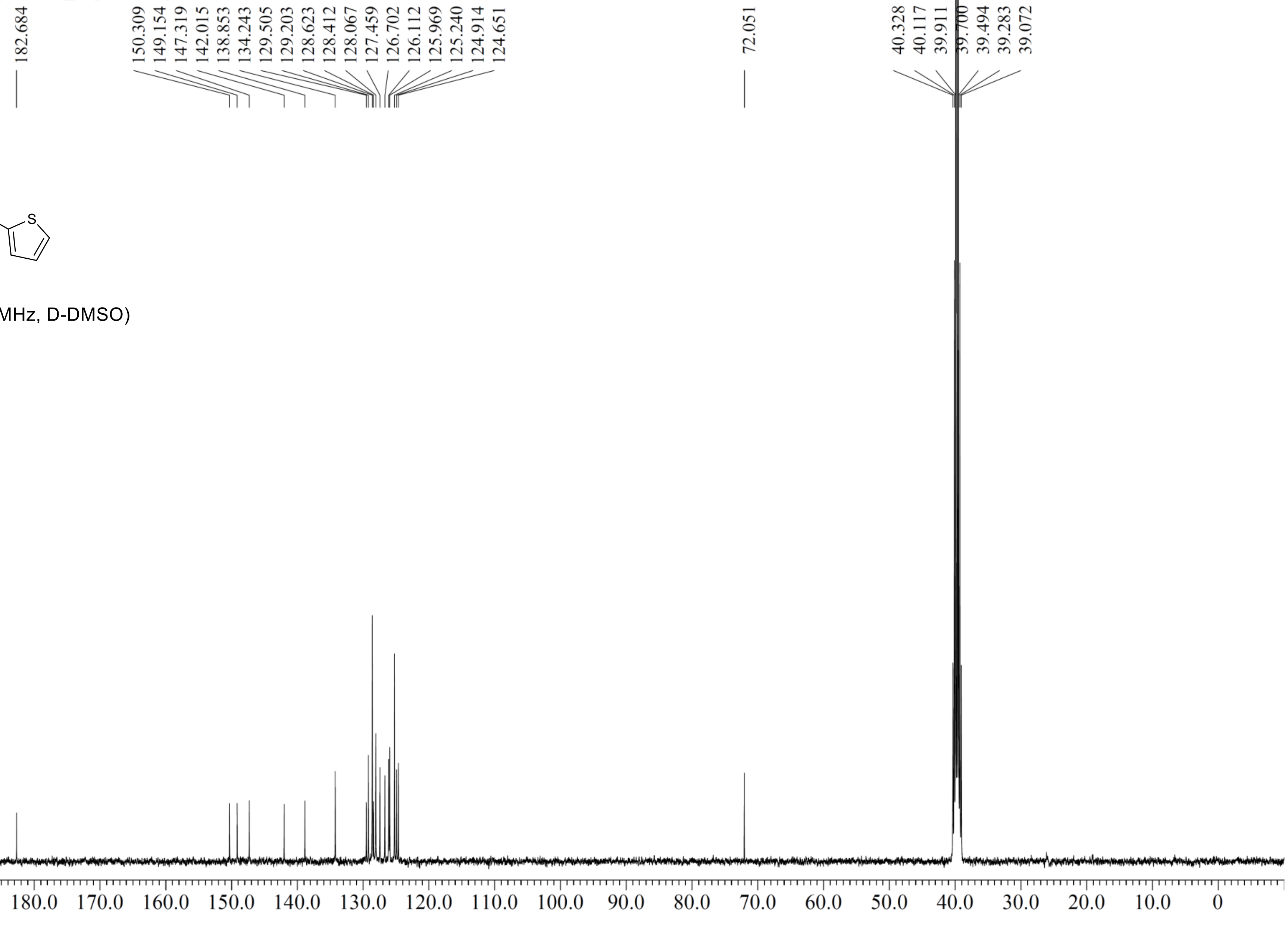

J-F Nu 4-F_Proton-1-2.jdf

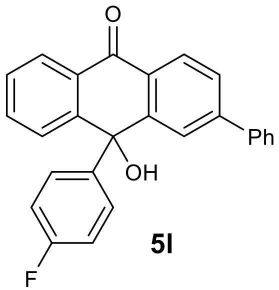

5l

($^1$H NMR, 400MHz, $CDCl_3$)

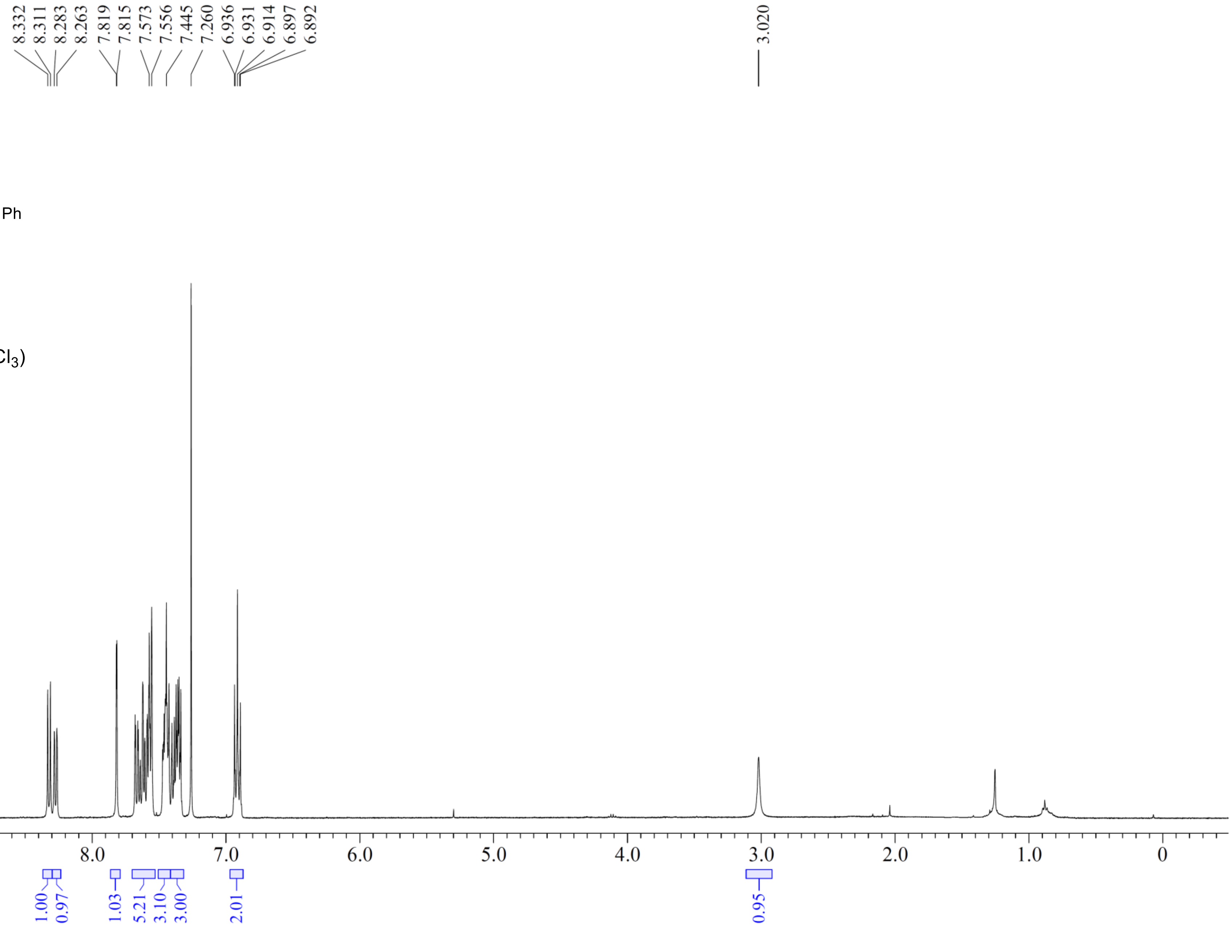

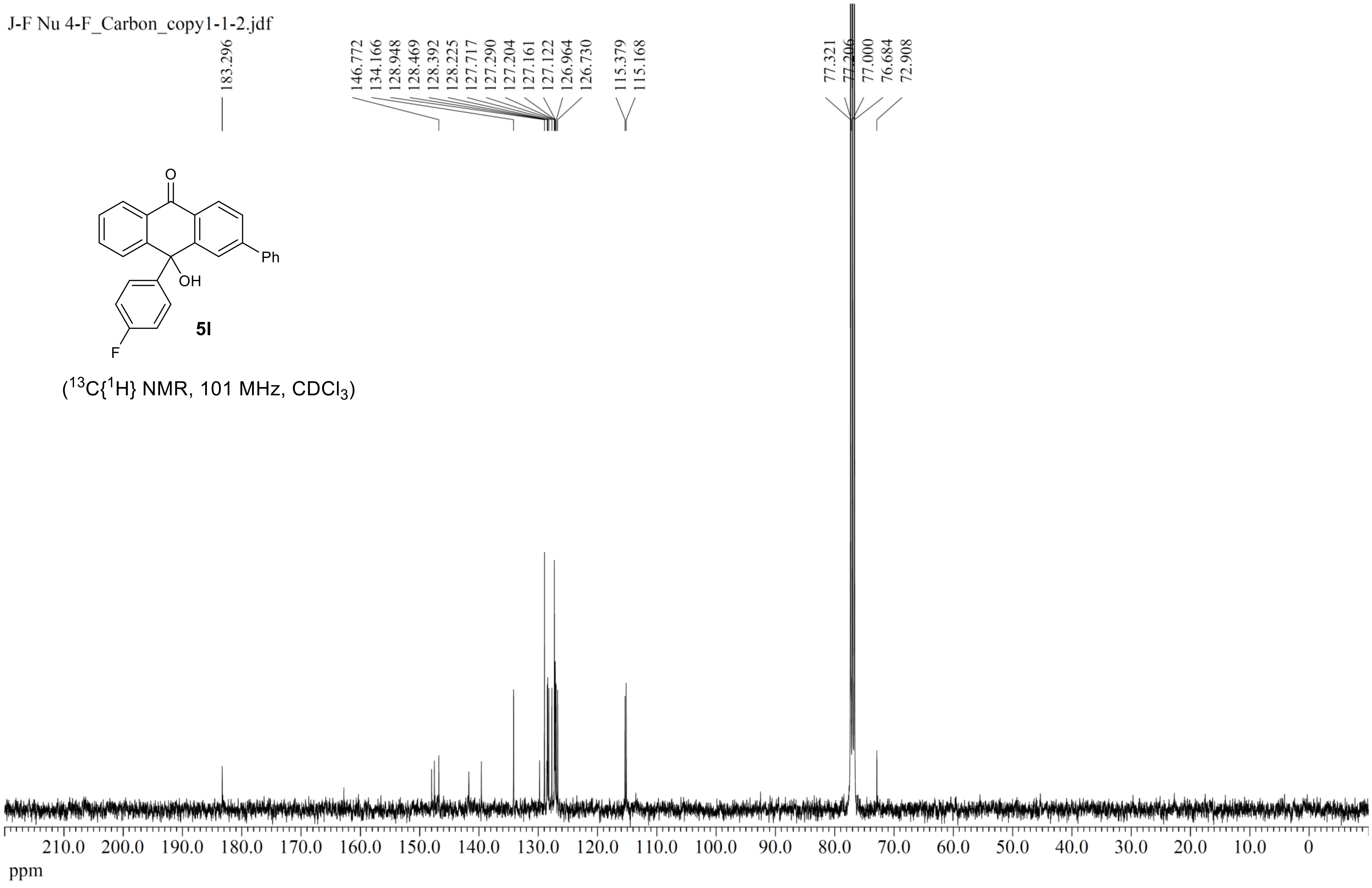

J-F Nu 4-F_Carbon_copy1-1-2.jdf
183.296
146.772
134.166
128.948
128.469
128.392
128.225
127.717
127.290
127.204
127.161
127.122
126.964
126.730
115.379
115.168
77.321
77.206
77.000
76.684
72.908
O
OH
Ph
F
5l
(13C{1H} NMR, 101 MHz, CDCl3)
210.0 200.0 190.0 180.0 170.0 160.0 150.0 140.0 130.0 120.0 110.0 100.0 90.0 80.0 70.0 60.0 50.0 40.0 30.0 20.0 10.0 0
ppm

J-F Nu 4-F_fluorine_1h_dec-1-2.jdf

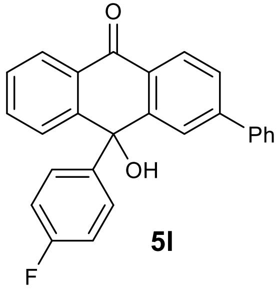


**5l**

($^{19}$F NMR, 376MHz, $CDCl_3$)

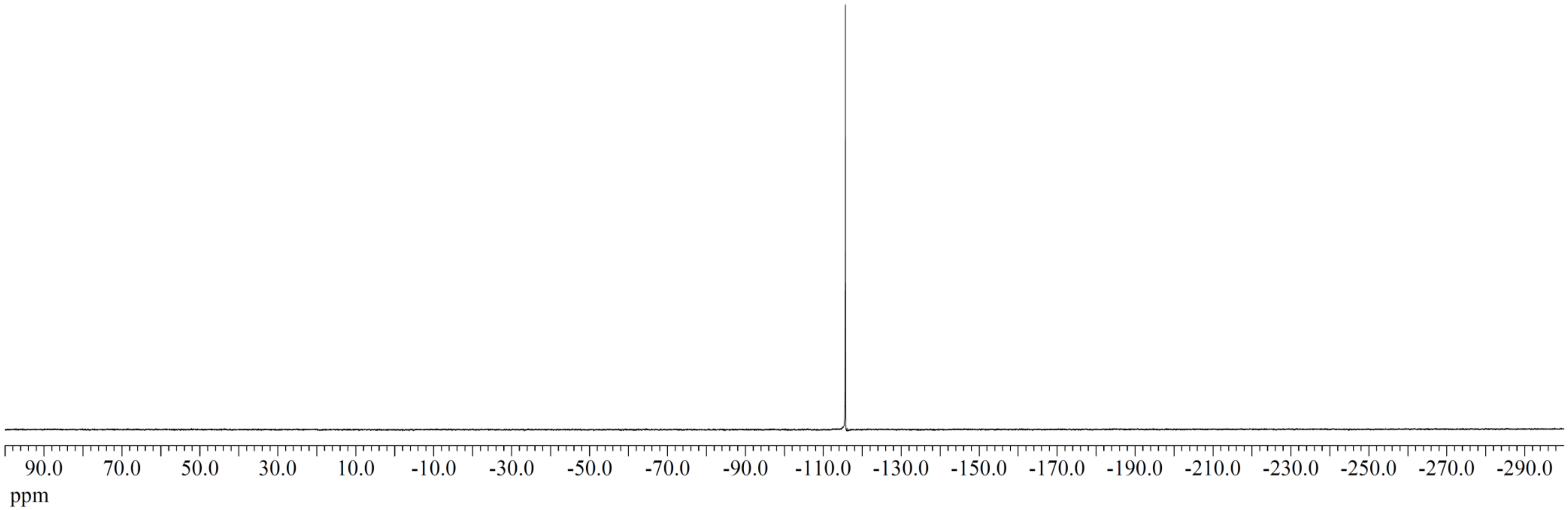

J-F Nu 4-Cl_Proton-3-2.jdf

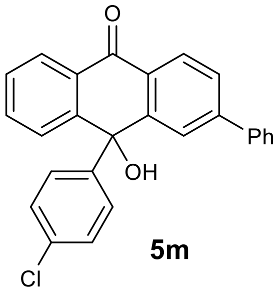

**5m**

($^{1}$H NMR, 400MHz, $CDCl_3$)

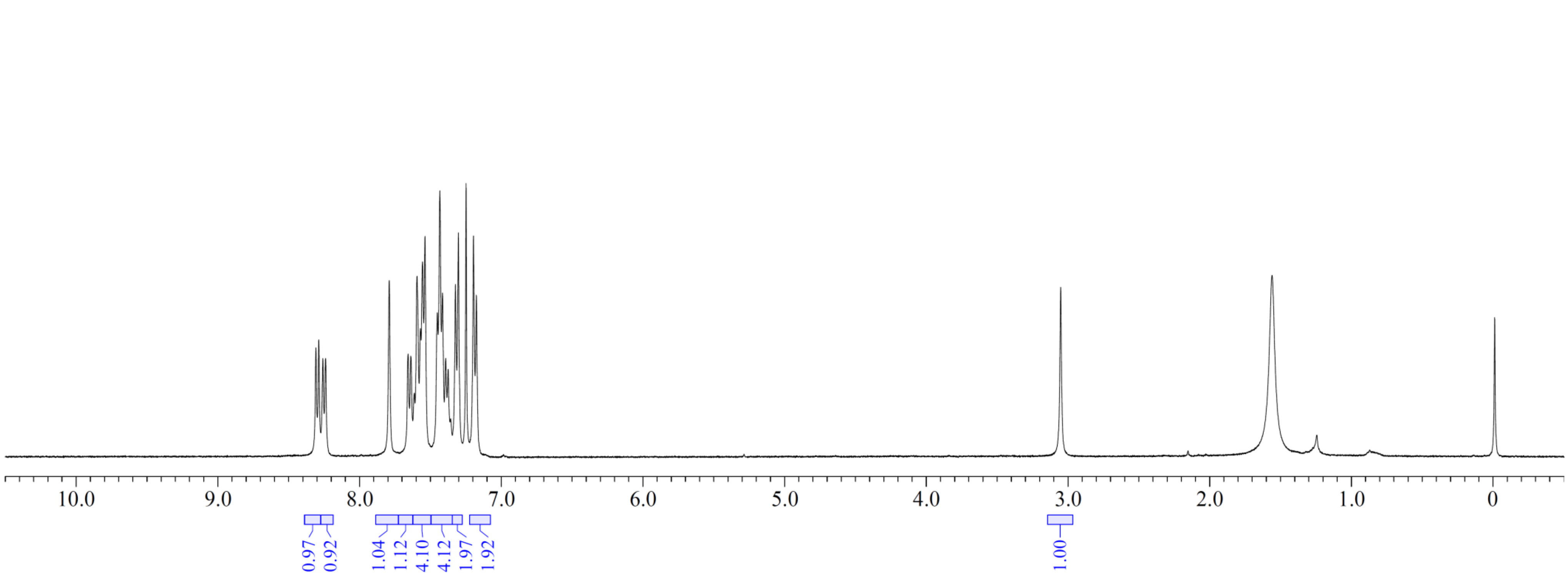

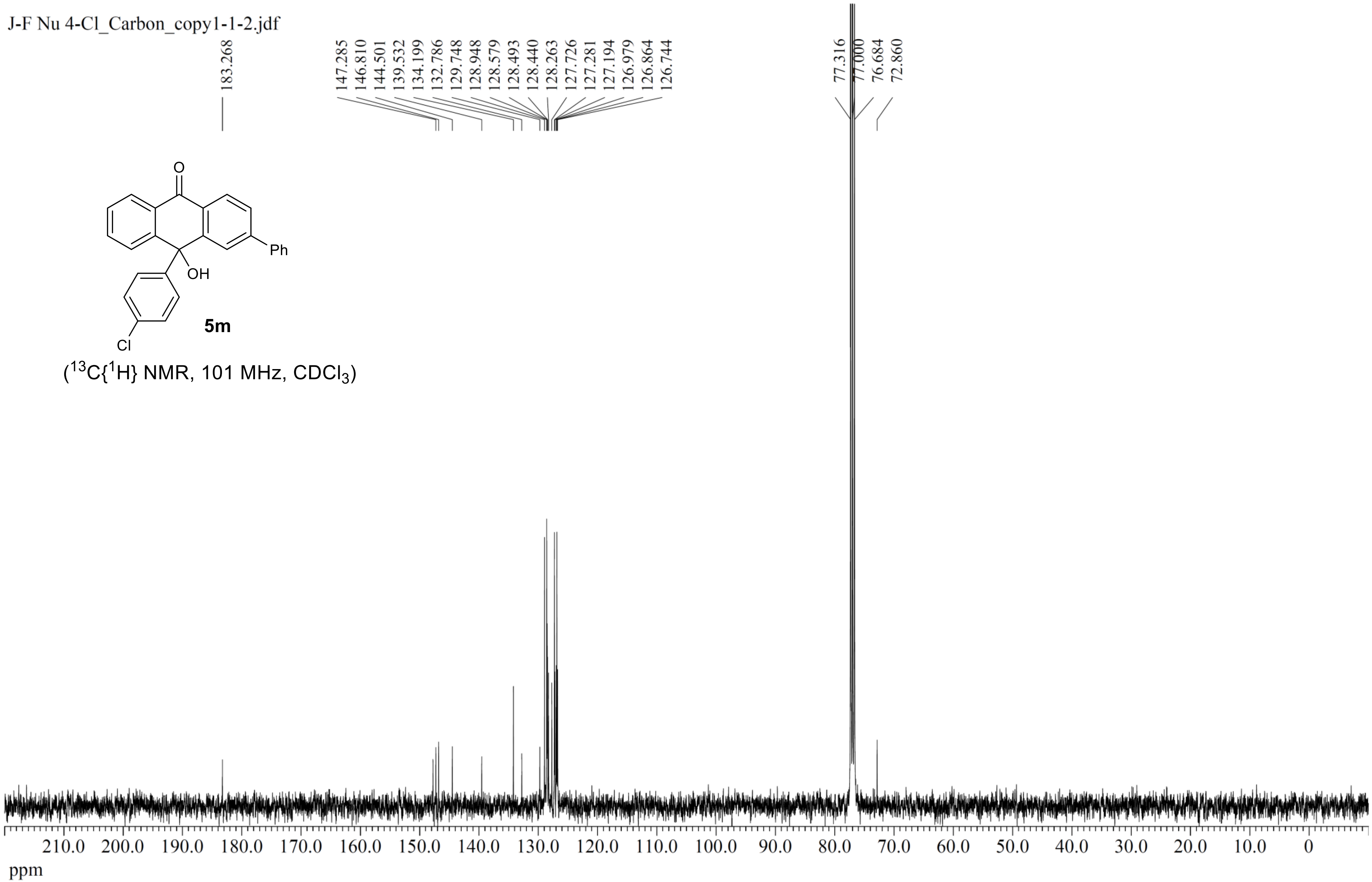


**5m**

($^{13}C\{^{1}H\}$ NMR, 101 MHz, $CDCl_3$)

J-F Nu 4-Br_Proton-1-2.jdf

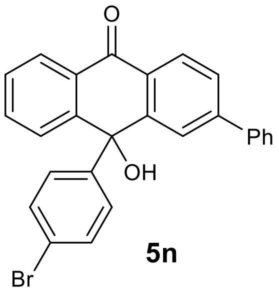

**5n**

($^1$H NMR, 400MHz, $CDCl_3$)

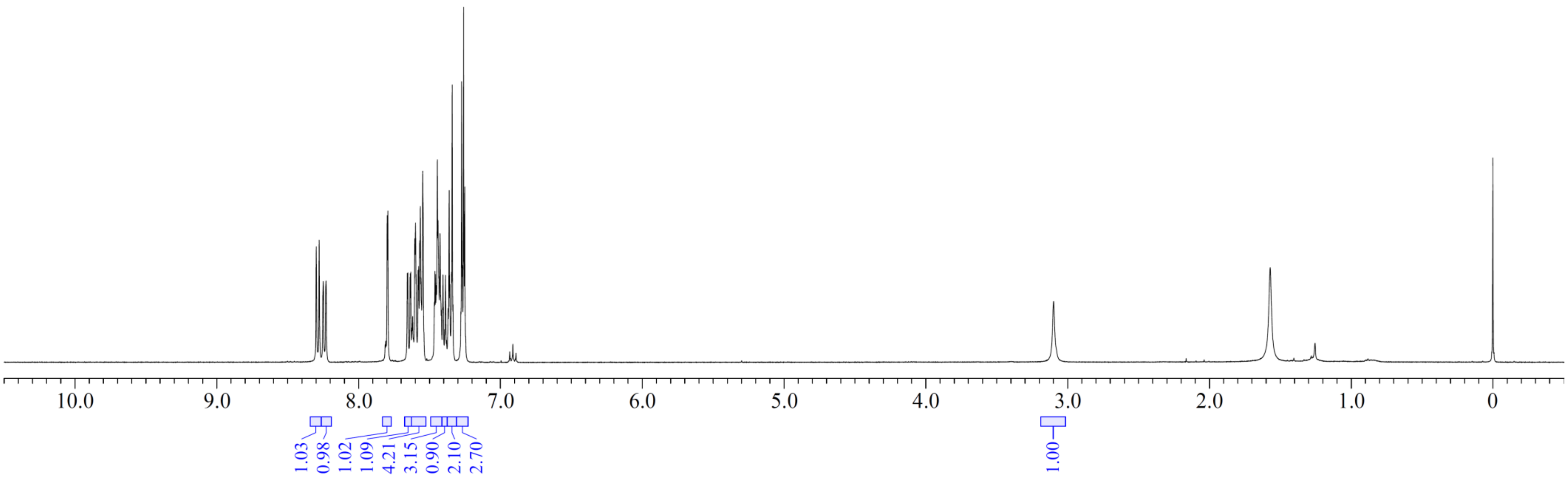

J-F Nu 4-Br_Carbon_copy1-1-2.jdf

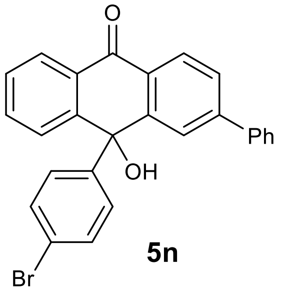

**5n**

($^{13}C\{^{1}H\}$ NMR, 101 MHz, $CDCl_3$)

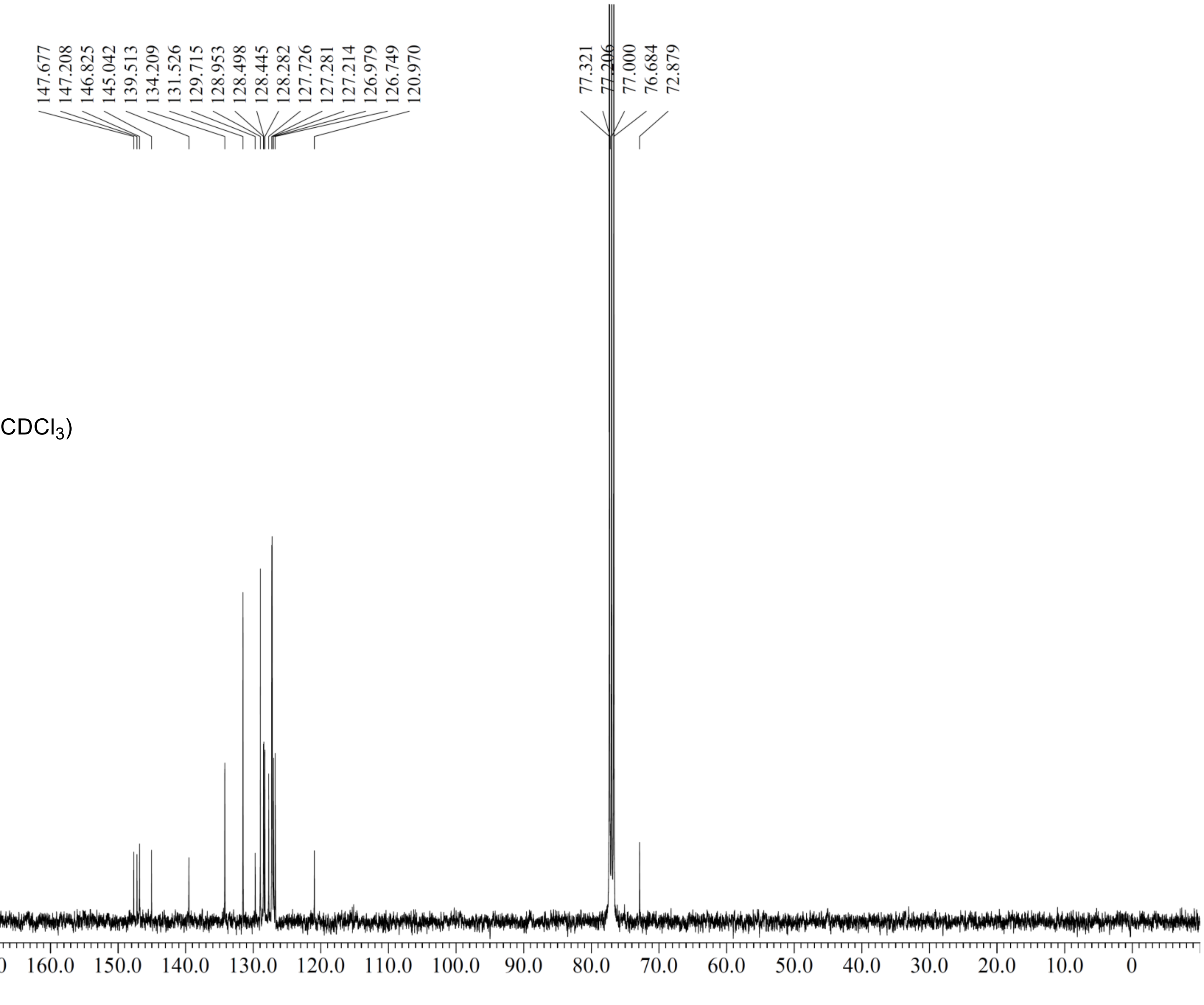

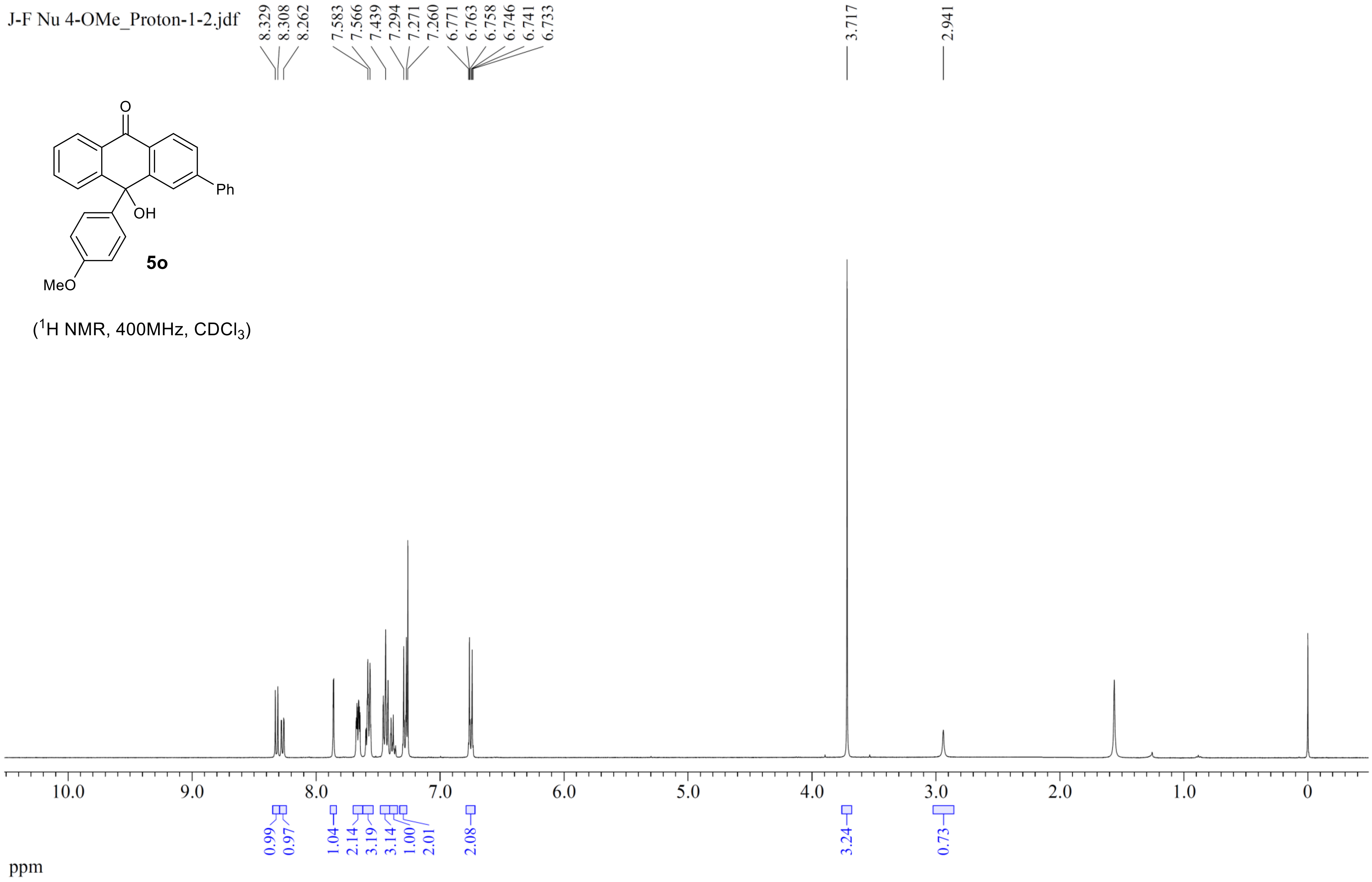
J-F Nu 4-OMe_Proton-1-2.jdf
8.329
8.308
8.262
7.583
7.566
7.439
7.294
7.271
7.260
6.771
6.763
6.758
6.746
6.741
6.733
3.717
2.941
O
OH
Ph
MeO
5o
(1H NMR, 400MHz, CDCl3)
10.0
9.0
8.0
7.0
6.0
5.0
4.0
3.0
2.0
1.0
0
0.99
0.97
1.04
2.14
3.19
3.14
1.00
2.01
2.08
3.24
0.73
ppm

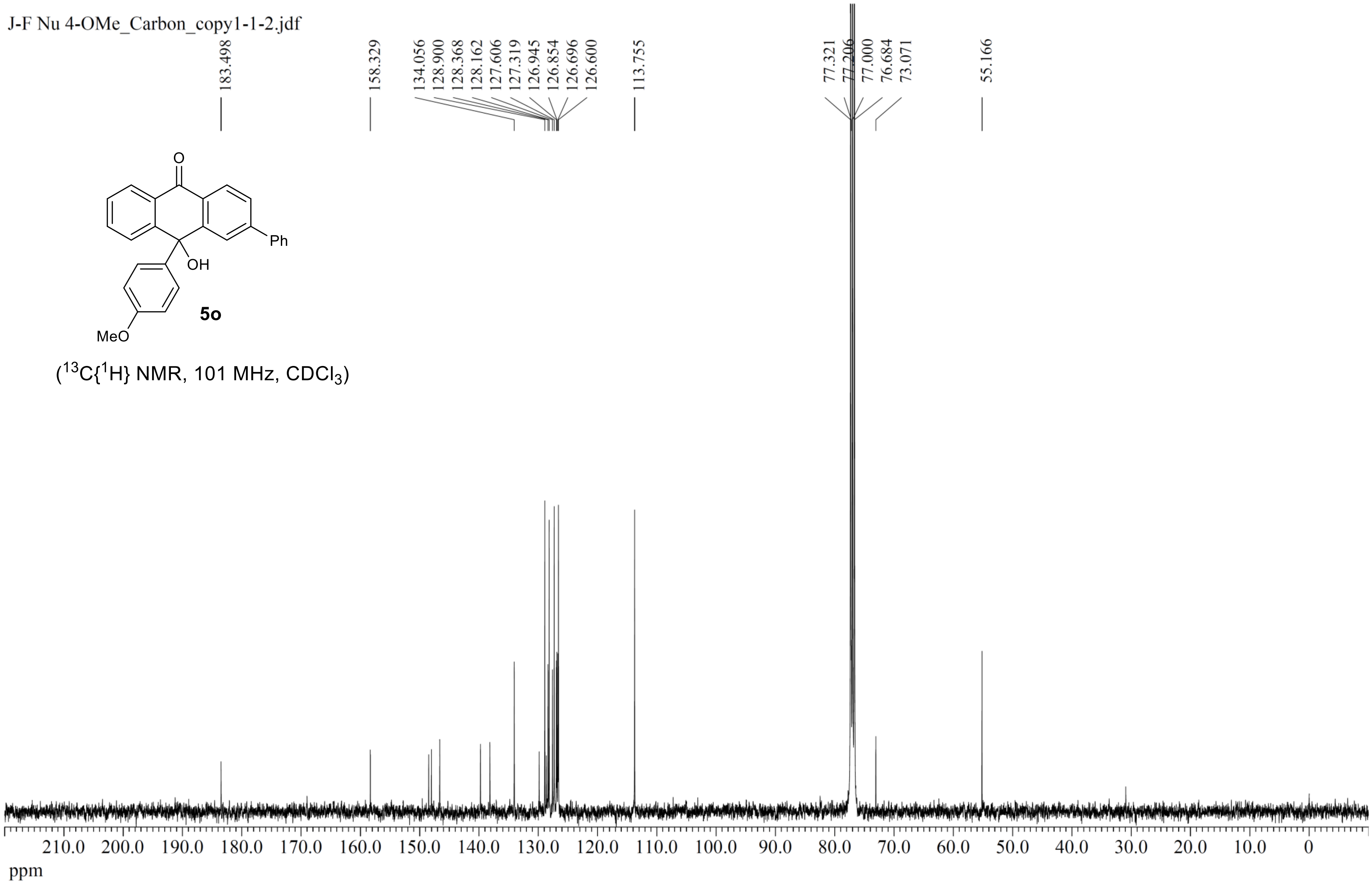
J-F Nu 4-OMe_Carbon_copy1-1-2.jdf
183.498
158.329
134.056
128.900
128.368
128.162
127.606
127.319
126.945
126.854
126.696
126.600
113.755
77.321
77.206
77.000
76.684
73.071
55.166
O
OH
Ph
MeO
5o
($^{13}$C{$^{1}$H} NMR, 101 MHz, $CDCl_3$)
210.0 200.0 190.0 180.0 170.0 160.0 150.0 140.0 130.0 120.0 110.0 100.0 90.0 80.0 70.0 60.0 50.0 40.0 30.0 20.0 10.0 0
ppm

J-F Nu 2-naph_Proton-2-2.jdf

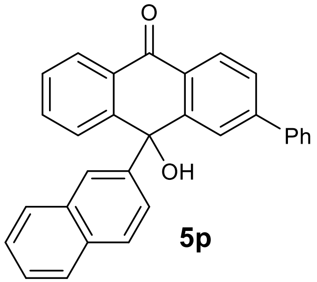

**5p**

($^1$H NMR, 400MHz, $CDCl_3$)

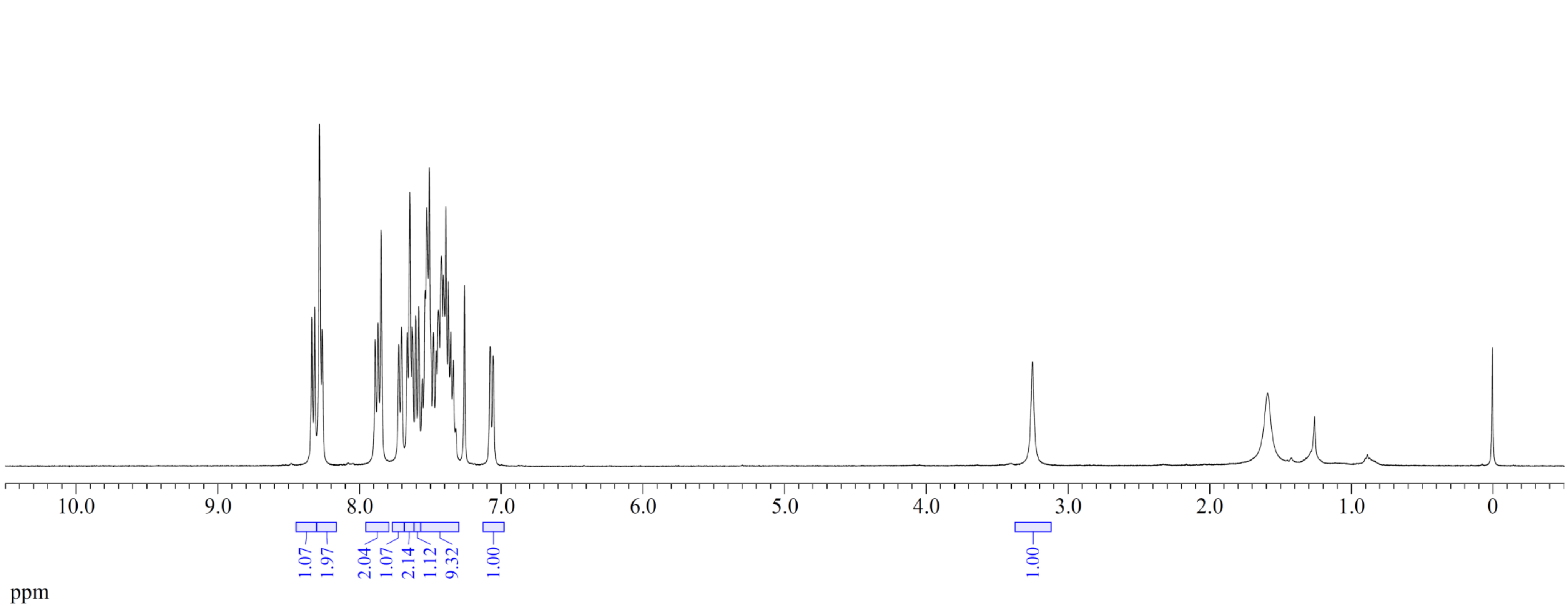

J-F Nu 2-naph_Carbon_copy4-1-2.jdf

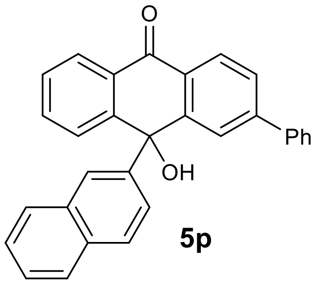


**5p**

($^{13}$C{$^{1}$H} NMR, 101 MHz, $CDCl_3$)

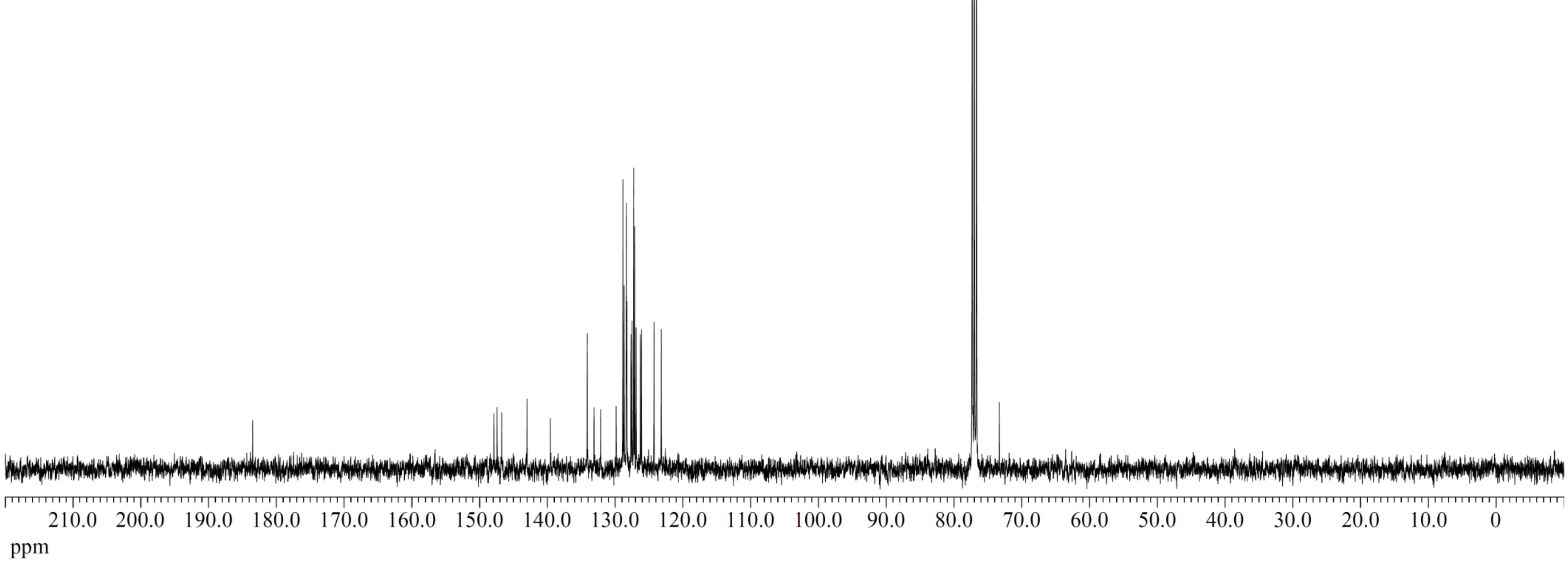

J-F Nu 2-thienyl_Proton-1-2.jdf

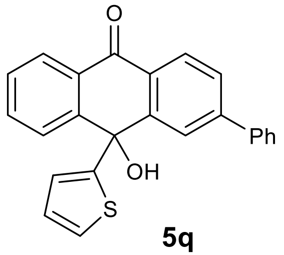

**5q**

($^1$H NMR, 400MHz, $CDCl_3$)

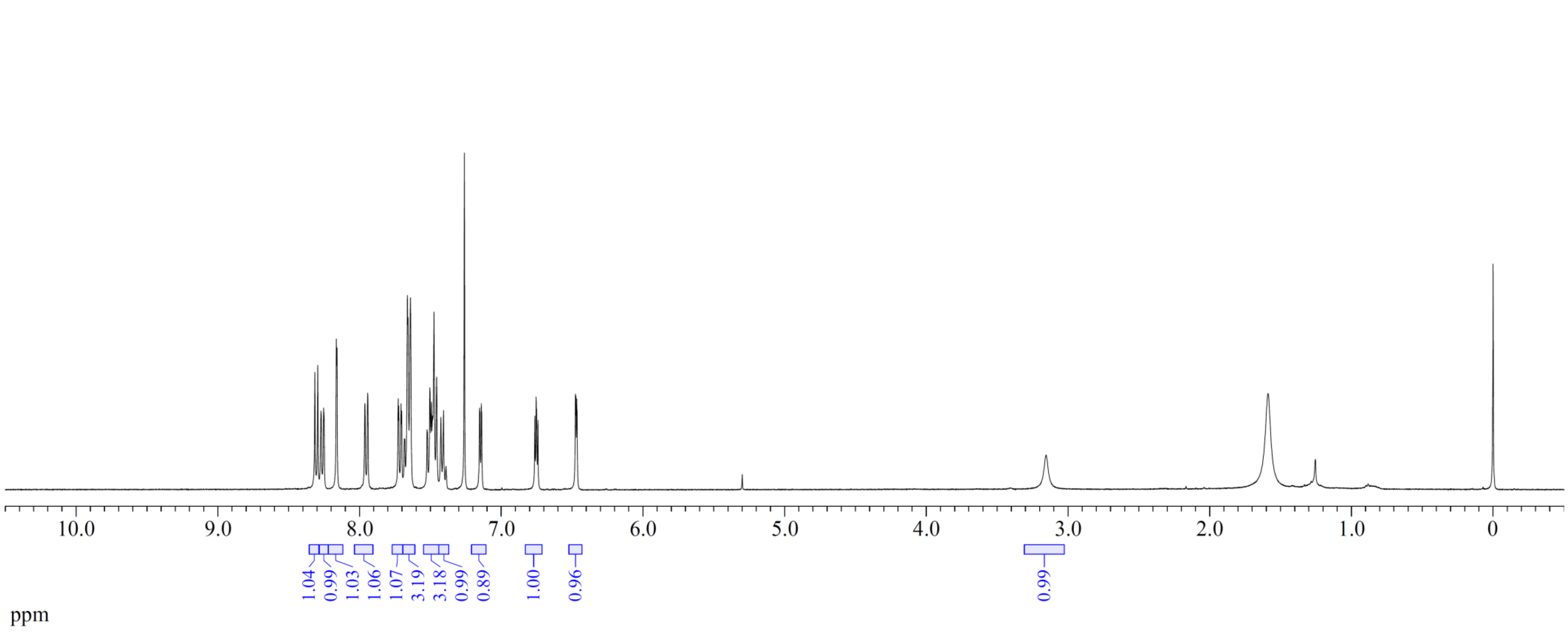

J-F Nu 2-thio_Carbon_copy1-1-2.jdf

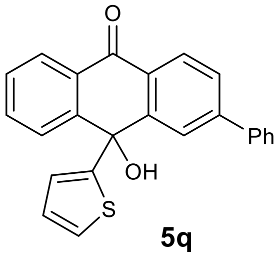

5q

($^{13}C\{^{1}H\}$ NMR, 101 MHz, $CDCl_3$)

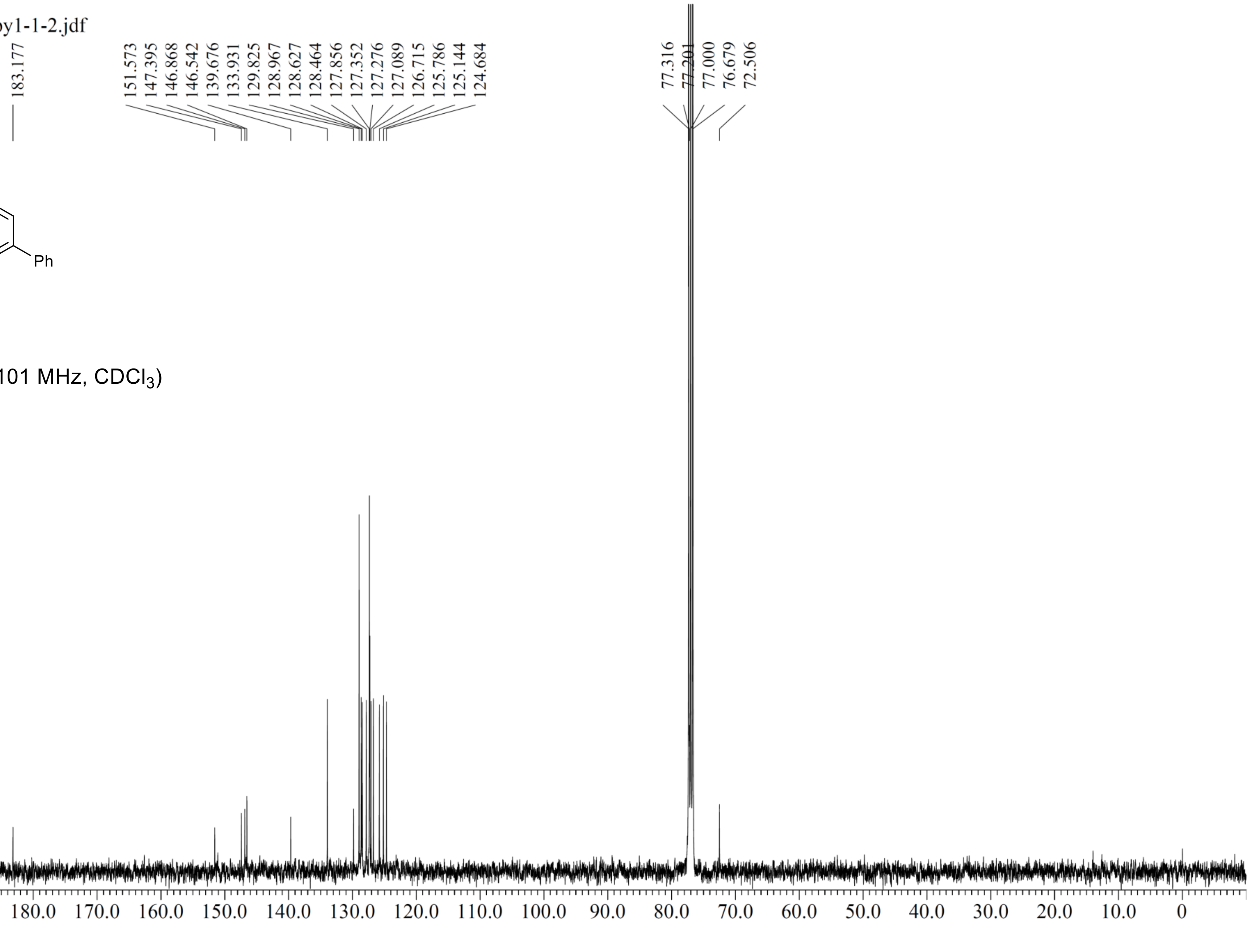

model-OEt-re_proton-1-2.jdf

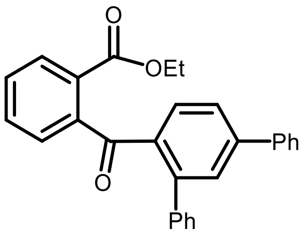

**6a**

($^{1}$H NMR, 400MHz, $CDCl_3$)

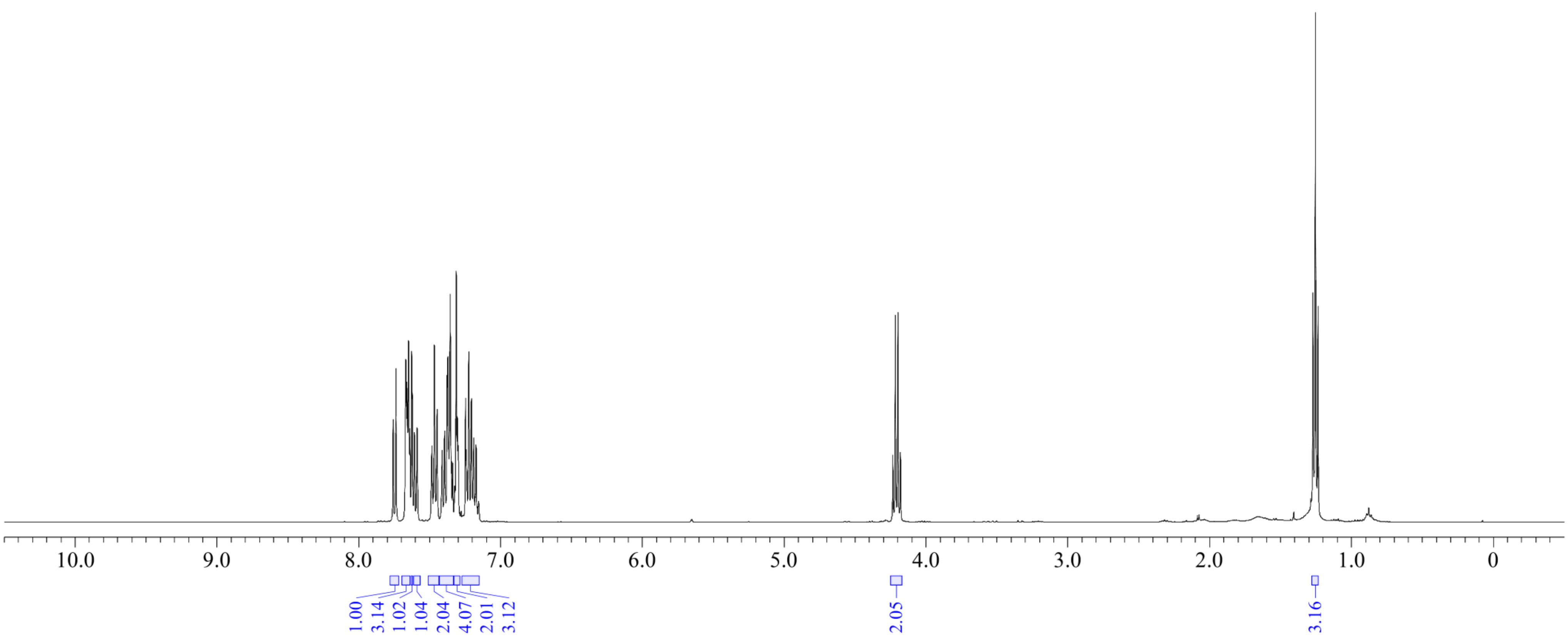

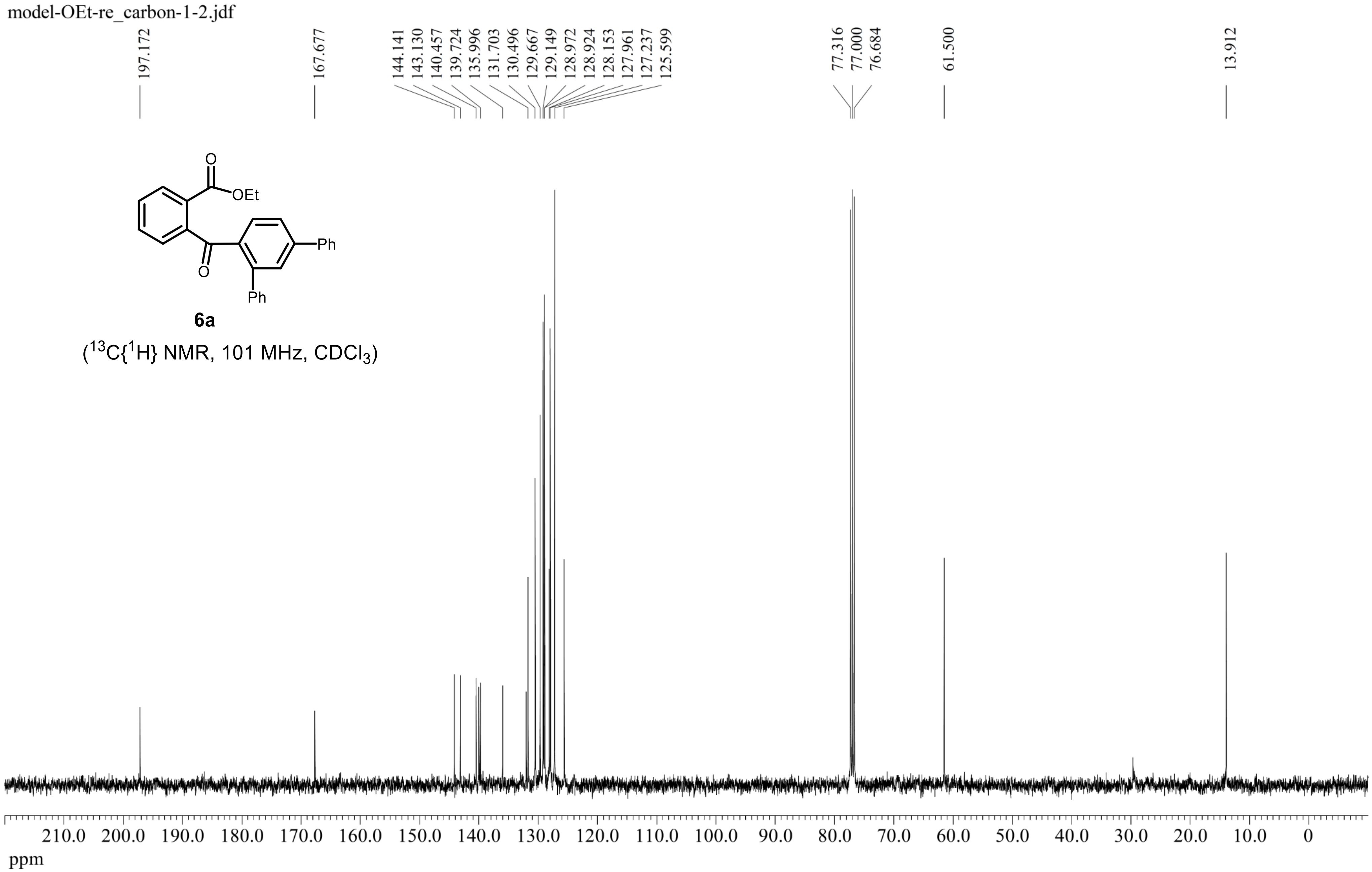
model-OEt-re_carbon-1-2.jdf
197.172
167.677
144.141
143.130
140.457
139.724
135.996
131.703
130.496
129.667
129.149
128.972
128.924
128.153
127.961
127.237
125.599
77.316
77.000
76.684
61.500
13.912
O
OEt
Ph
O
Ph
6a
($^{13}$C{$^1$H} NMR, 101 MHz, $CDCl_3$)
210.0 200.0 190.0 180.0 170.0 160.0 150.0 140.0 130.0 120.0 110.0 100.0 90.0 80.0 70.0 60.0 50.0 40.0 30.0 20.0 10.0 0
ppm

el-2OMere-plz_proton-1-2.jdf

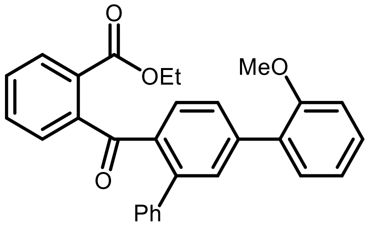


**6b**

($^1$H NMR, 400MHz, $CDCl_3$)

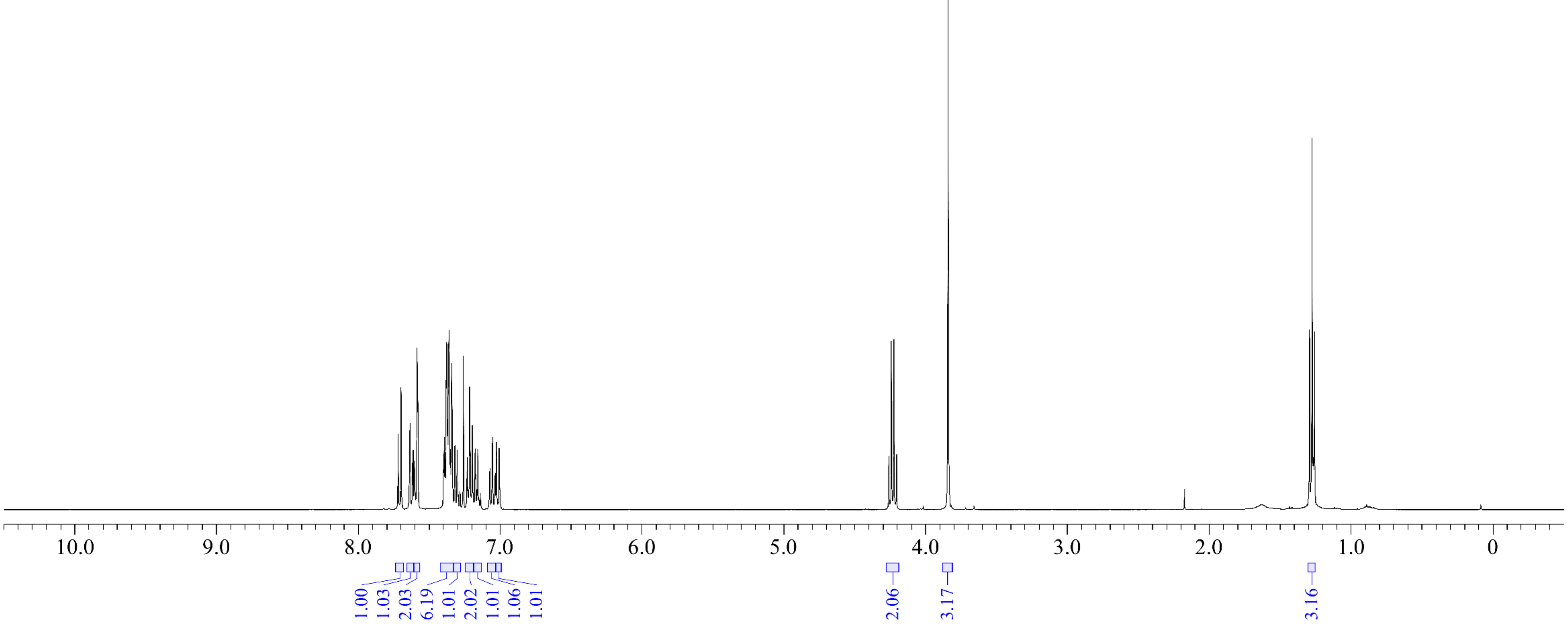


ppm

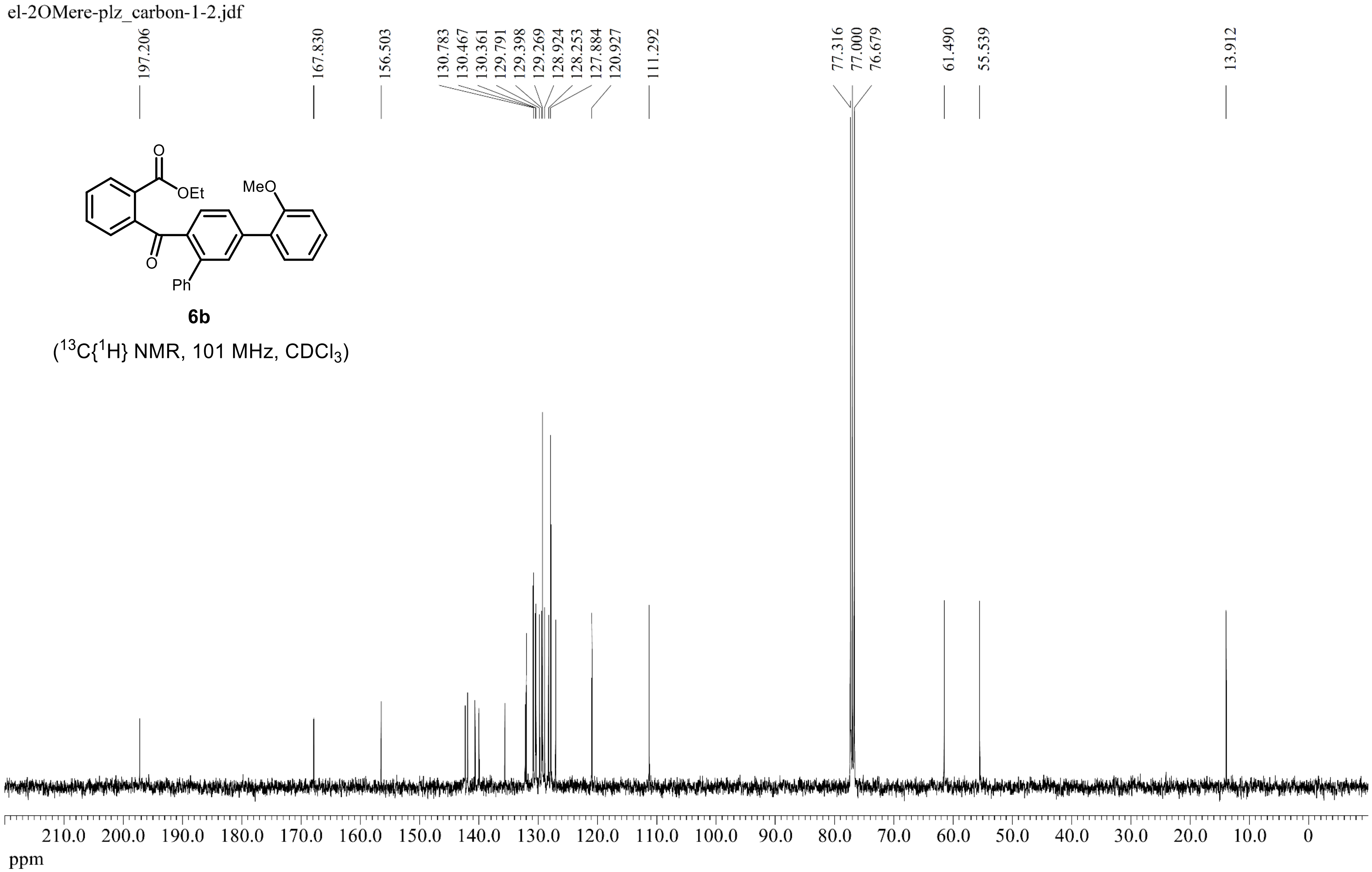
el-2OMere-plz_carbon-1-2.jdf
197.206
167.830
156.503
130.783
130.467
130.361
129.791
129.398
129.269
128.924
128.253
127.884
120.927
111.292
77.316
77.000
76.679
61.490
55.539
13.912
O
OEt
MeO
O
Ph
6b
($^{13}C\{^{1}H\}$ NMR, 101 MHz, $CDCl_3$)
210.0
200.0
190.0
180.0
170.0
160.0
150.0
140.0
130.0
120.0
110.0
100.0
90.0
80.0
70.0
60.0
50.0
40.0
30.0
20.0
10.0
0
ppm

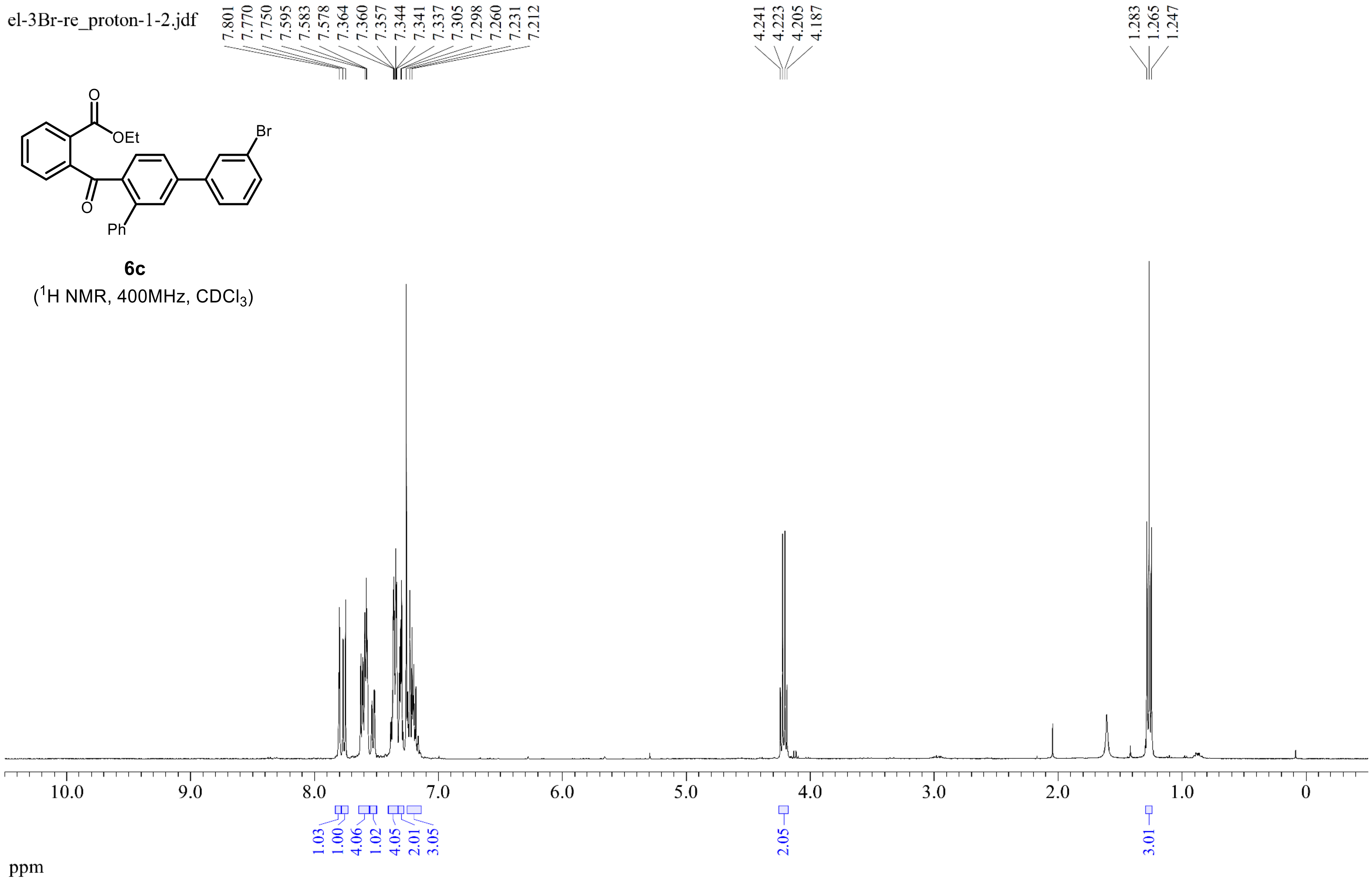


**6c**

($^1$H NMR, 400MHz, $CDCl_3$)

el-3Br-re_carbon-1-2.jdf

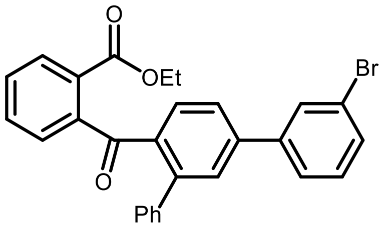

**6c**

($^{13}C\{^1H\}$ NMR, 101 MHz, $CDCl_3$)

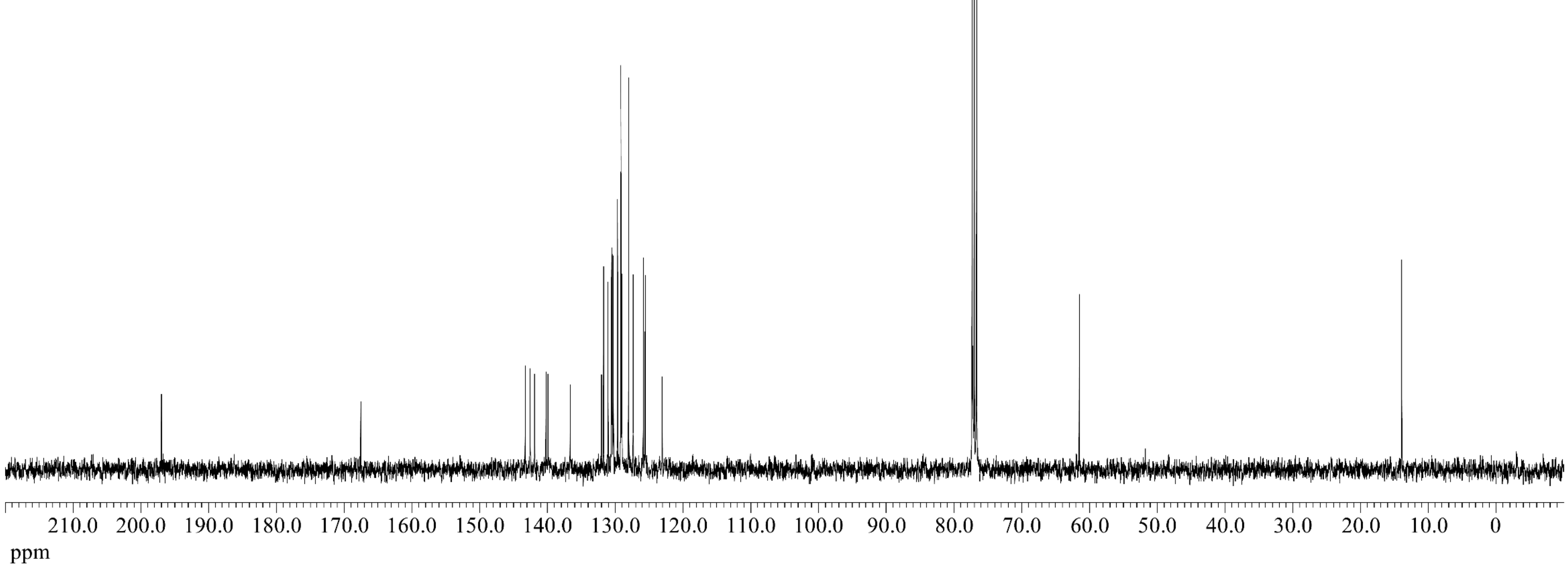

el4F-re_proton-1-2.jdf

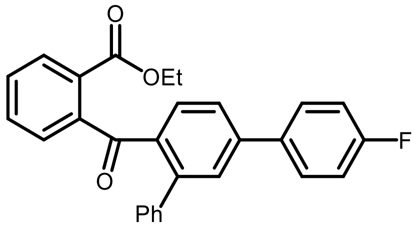


**6d**

($^1$H NMR, 400MHz, $CDCl_3$)

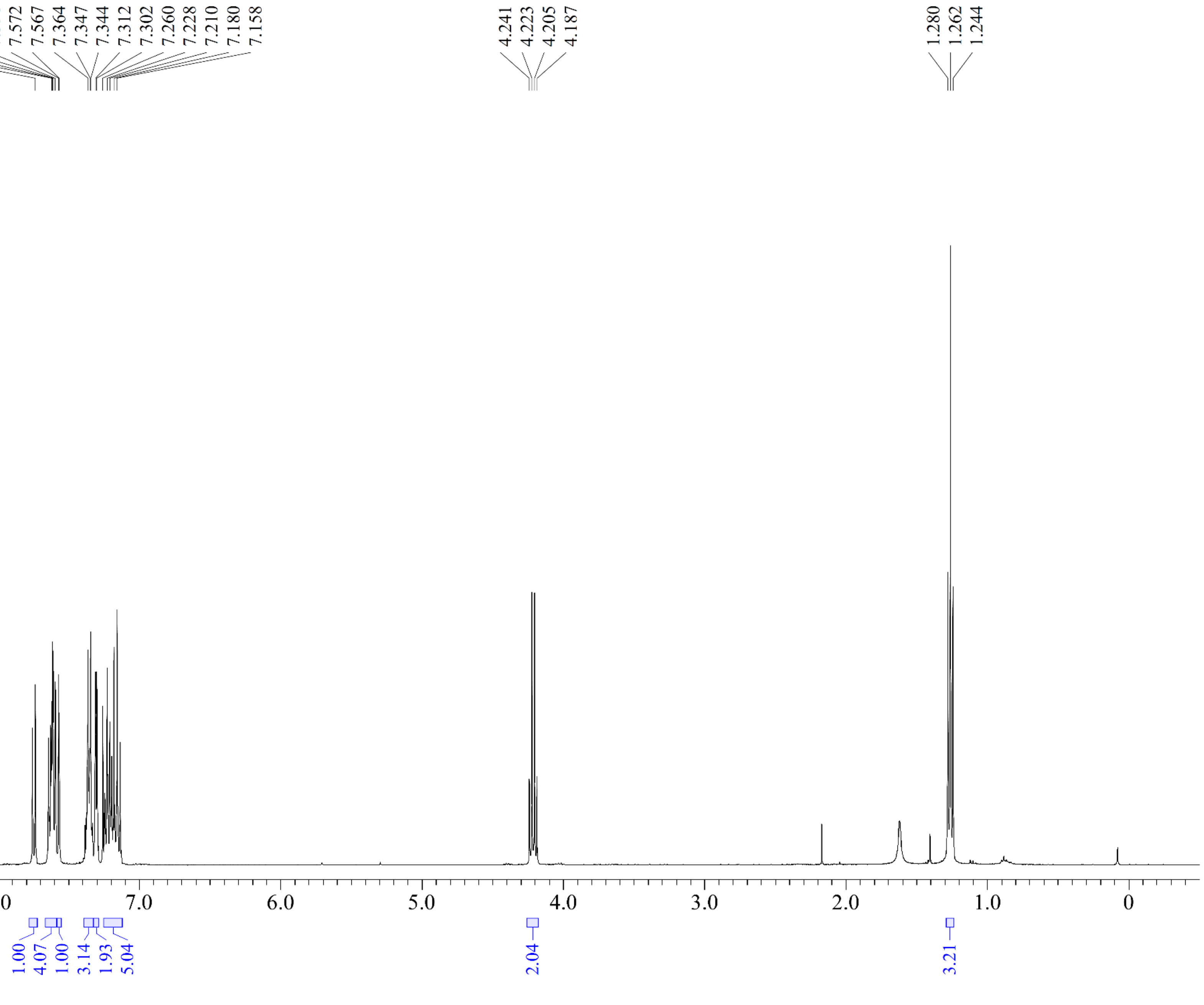

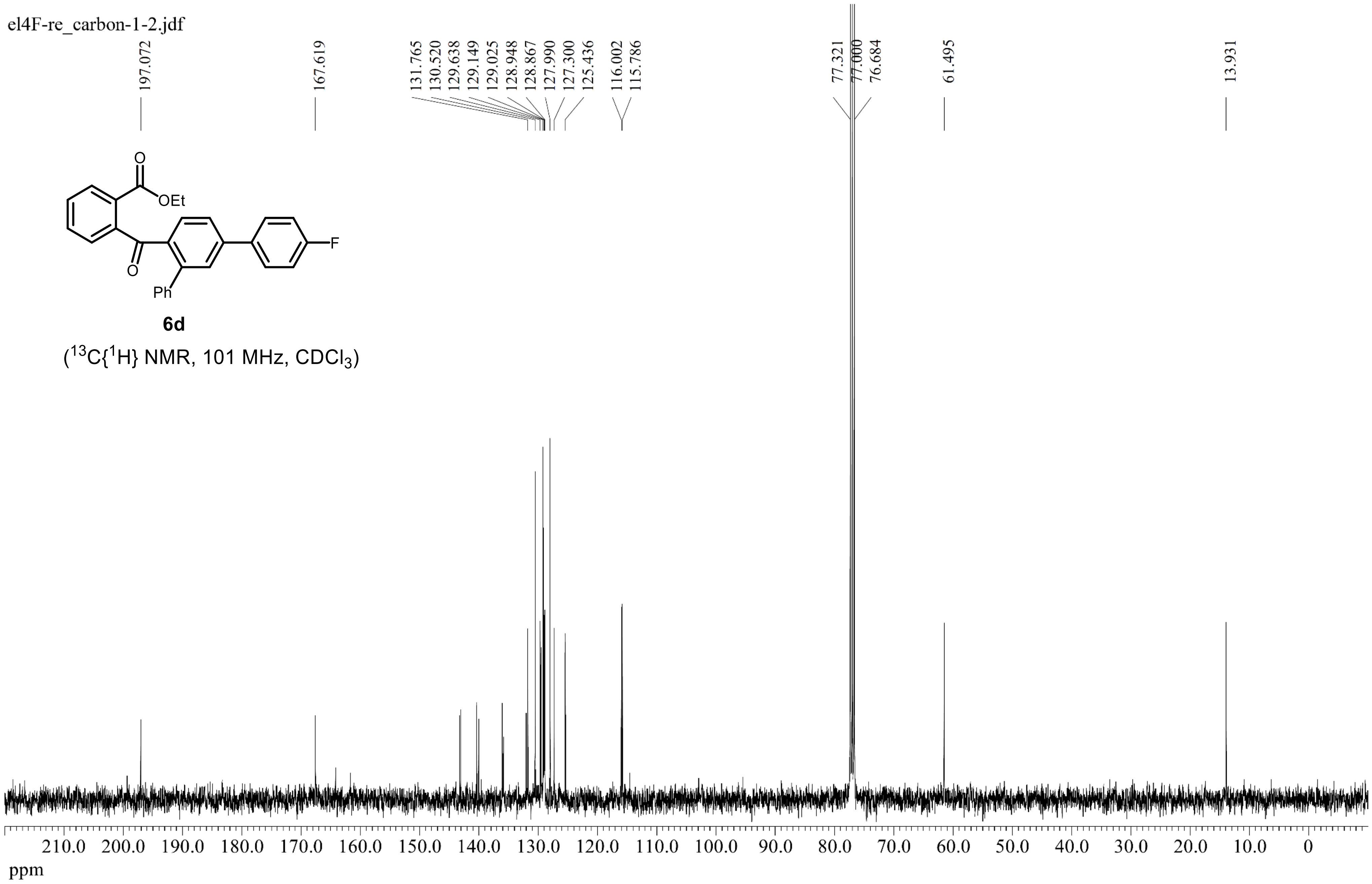

el4F-re_carbon-1-2.jdf
197.072
167.619
131.765
130.520
129.638
129.149
129.025
128.948
128.867
127.990
127.300
125.436
116.002
115.786
77.321
77.000
76.684
61.495
13.931
O
OEt
F
O
Ph
6d
($^{13}C\{^{1}H\}$ NMR, 101 MHz, $CDCl_3$)
210.0 200.0 190.0 180.0 170.0 160.0 150.0 140.0 130.0 120.0 110.0 100.0 90.0 80.0 70.0 60.0 50.0 40.0 30.0 20.0 10.0 0
ppm

el4F-re_19F-1-2.jdf

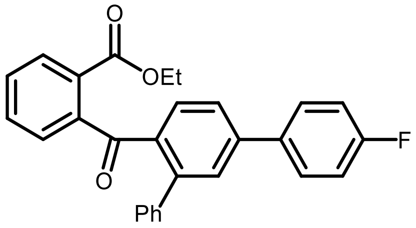


**6d**

($^{19}$F NMR, 376MHz, $CDCl_3$)

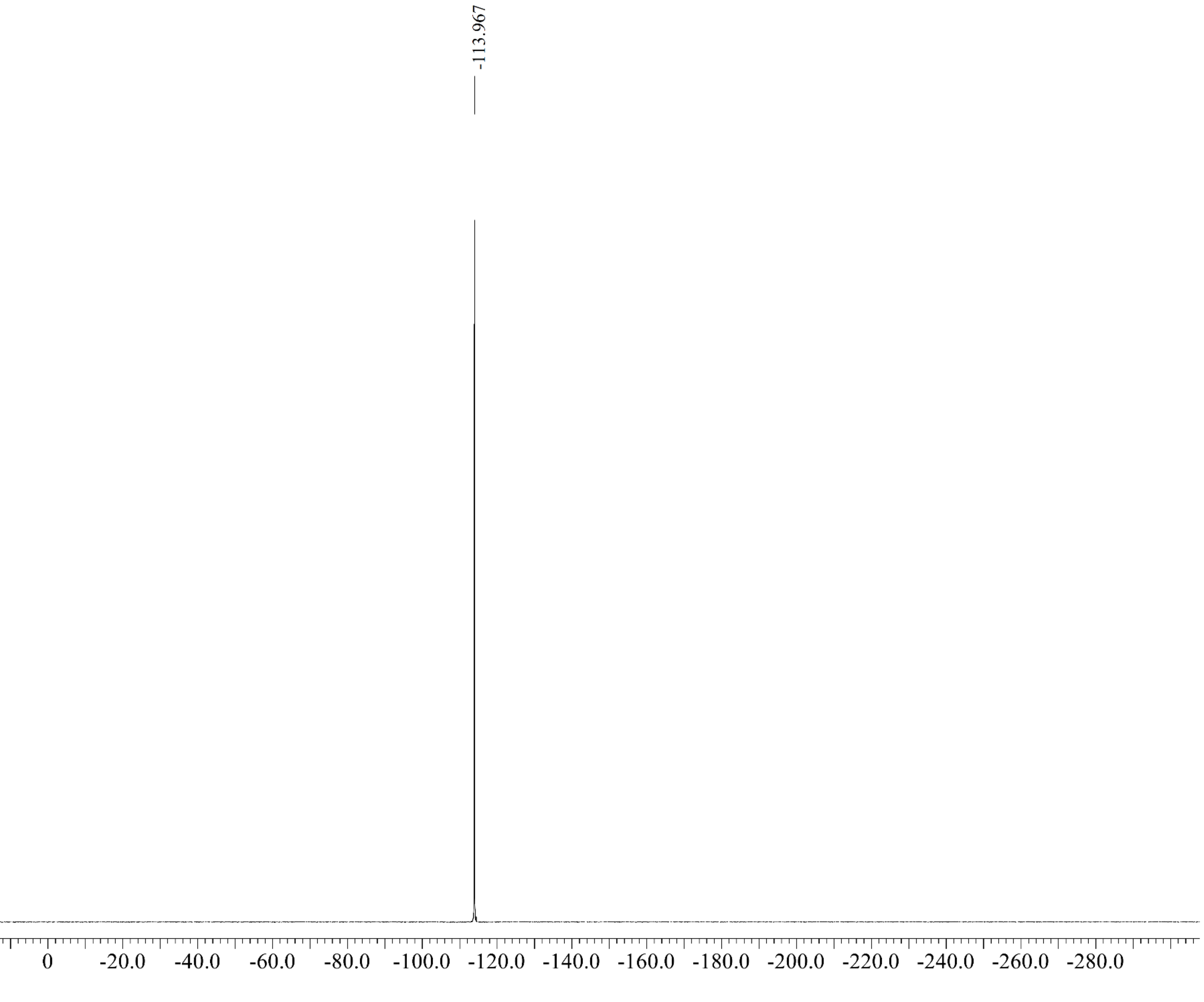

el-4Cl-re-plz_proton-1-2.jdf

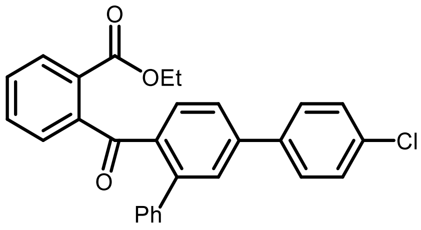


**6e**

($^{1}$H NMR, 400MHz, $CDCl_3$)

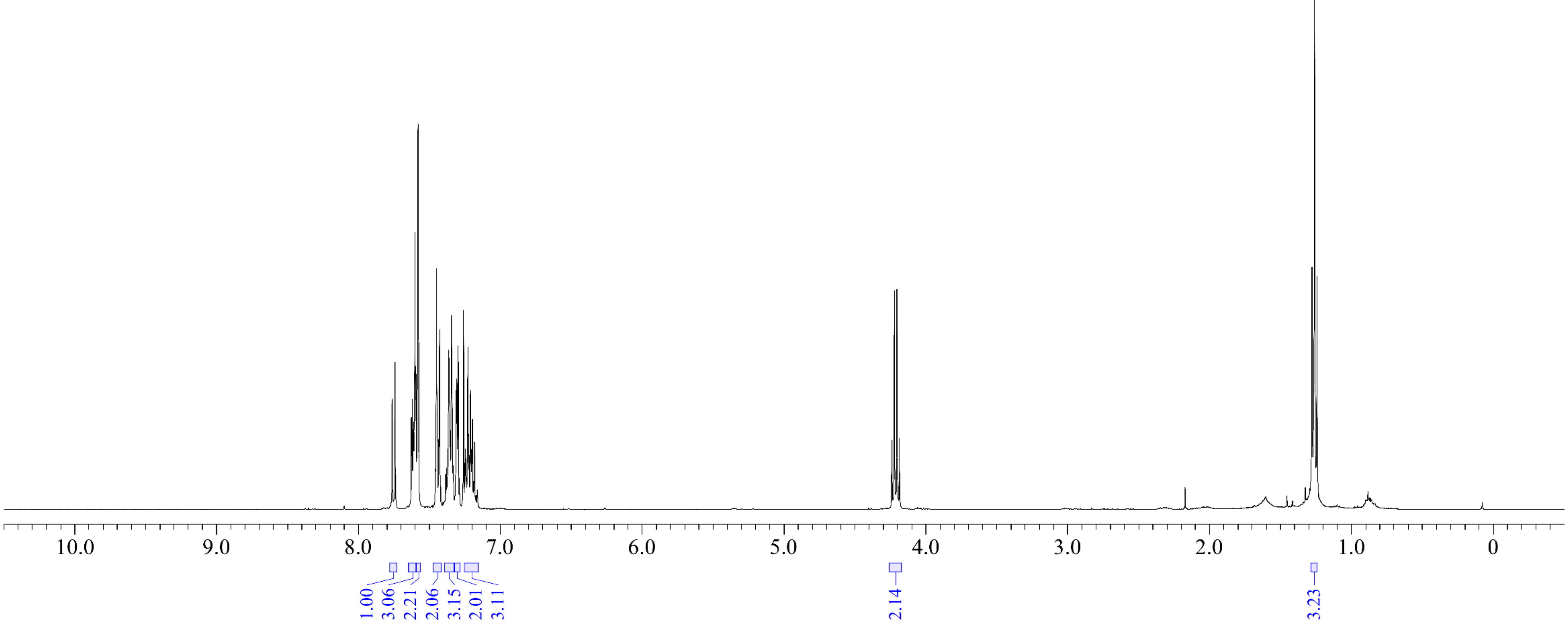


ppm

el-4Cl-re-plz_carbon-1-2.jdf

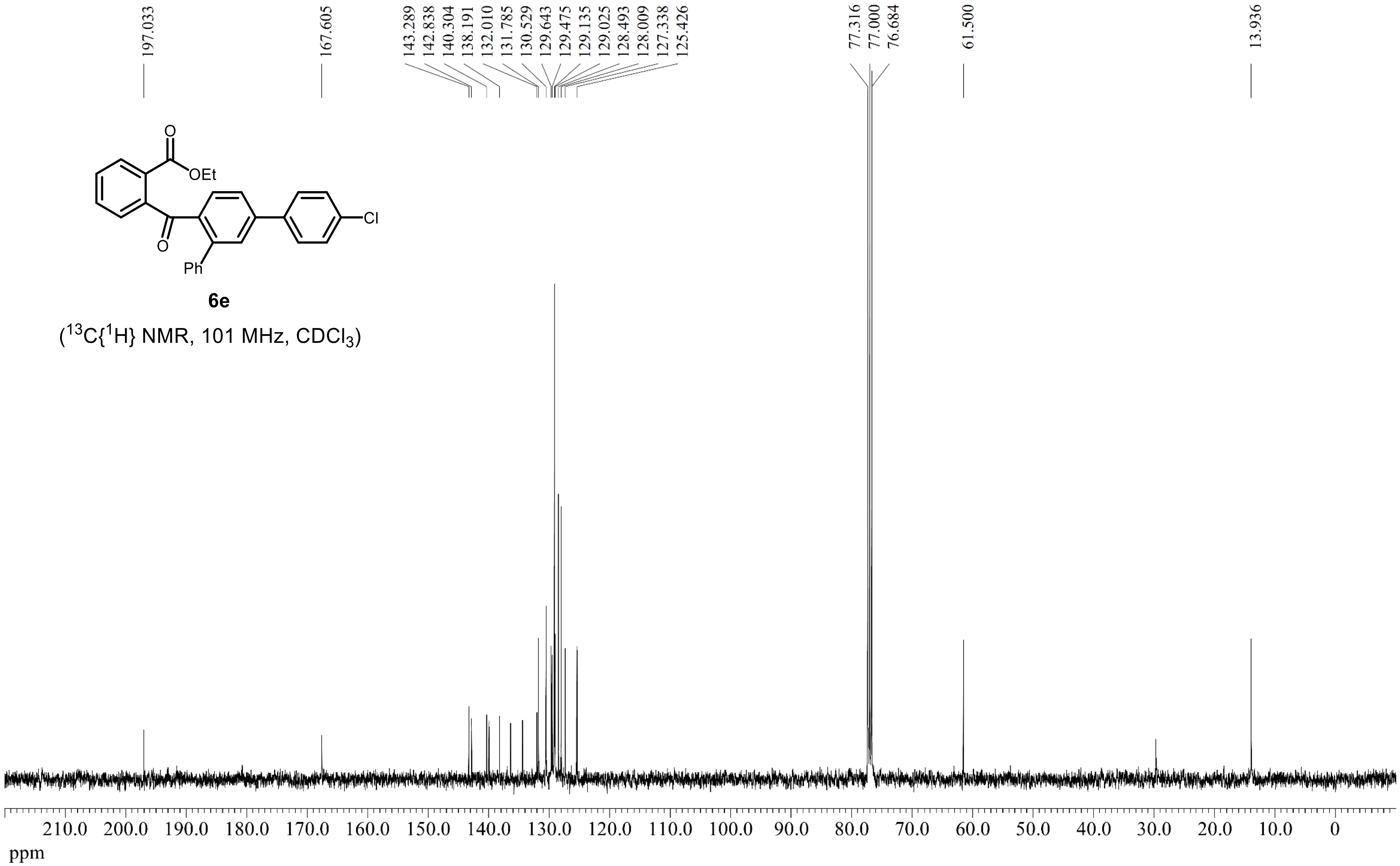


**6e**

($^{13}C\{^{1}H\}$ NMR, 101 MHz, $CDCl_3$)

el-4Br-re_proton-1-2.jdf

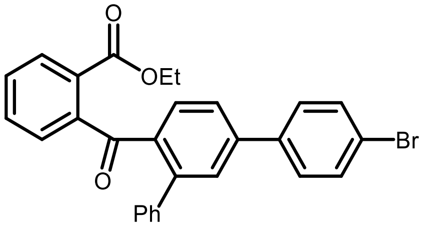


**6f**

($^{1}$H NMR, 400MHz, $CDCl_3$)

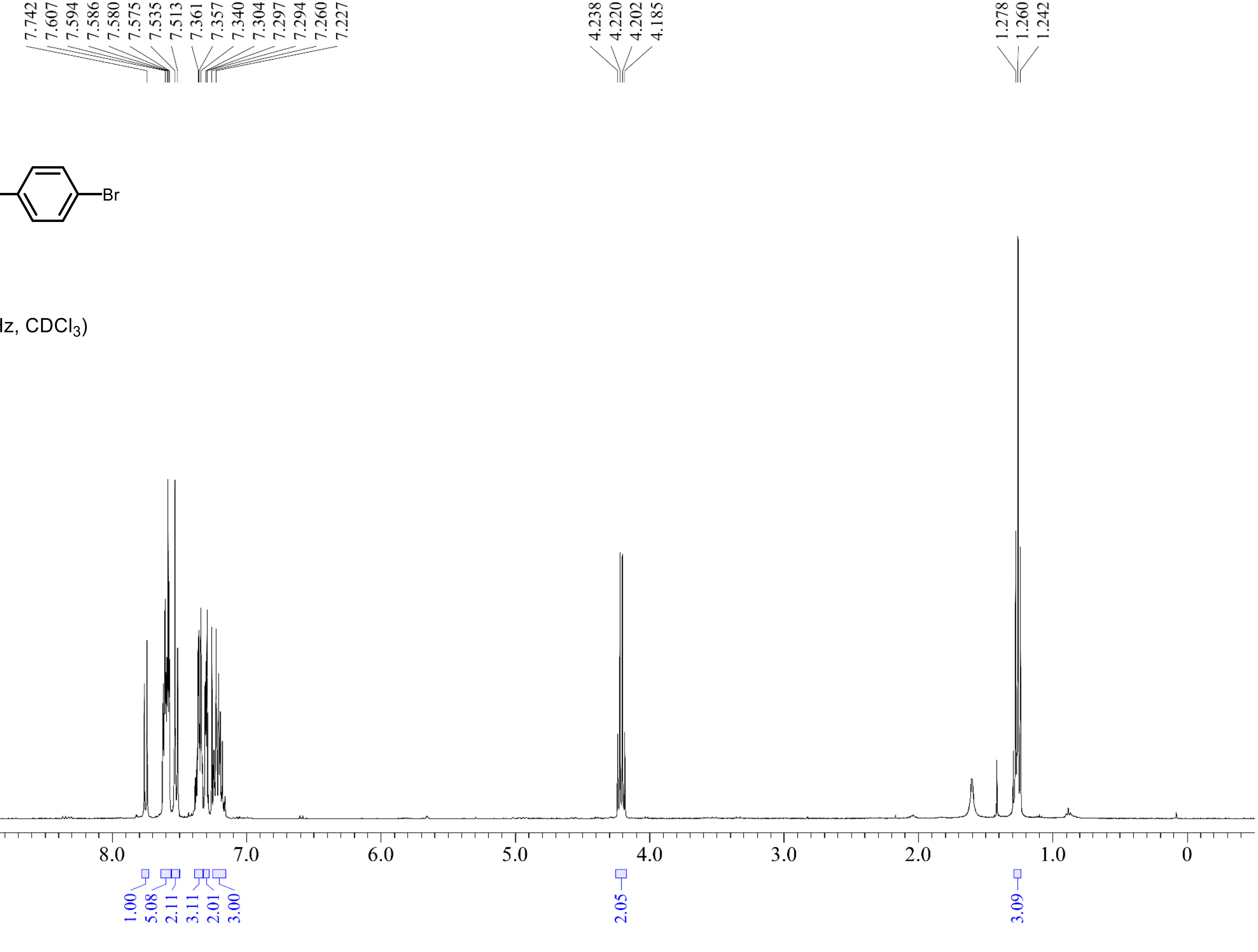

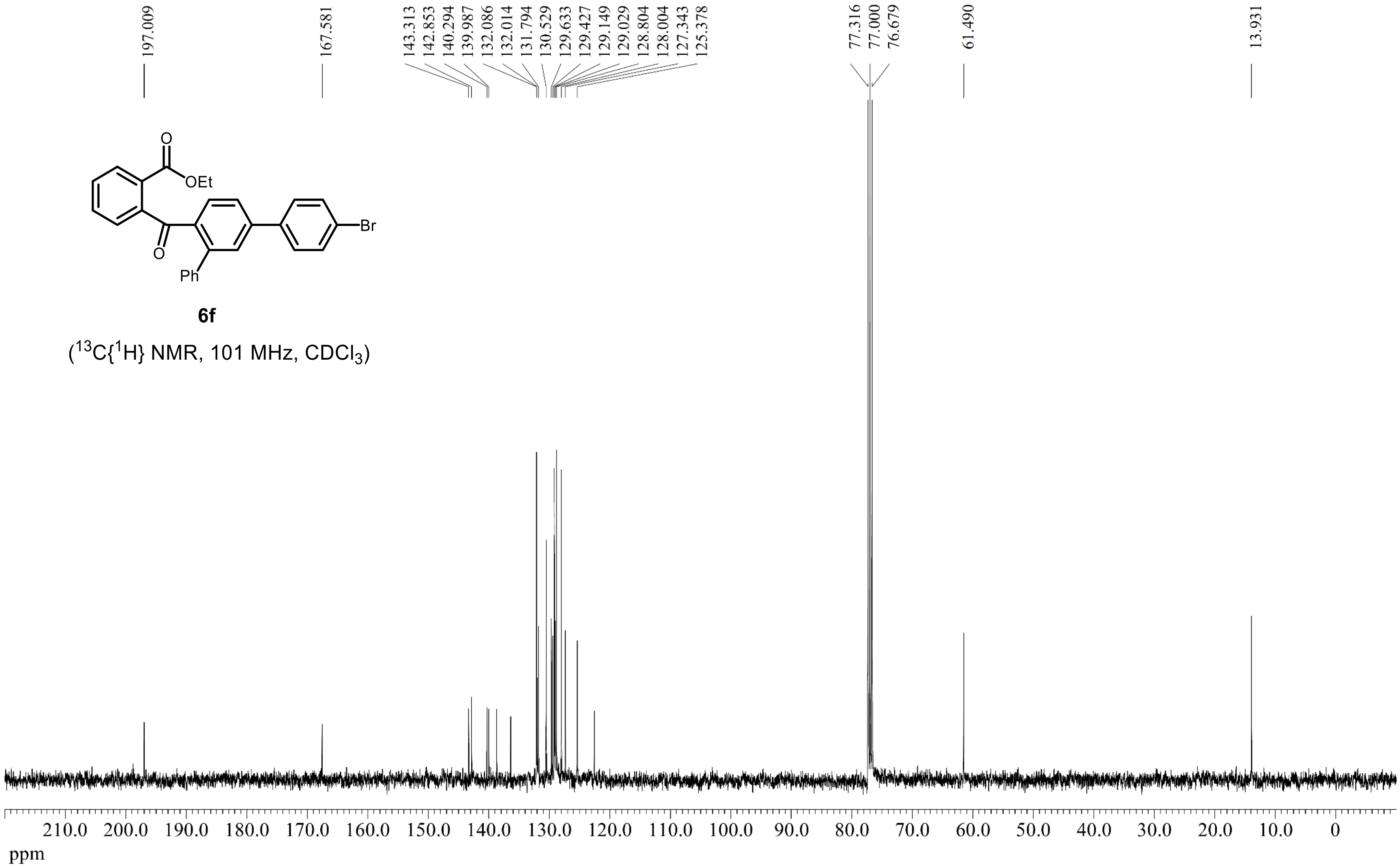


**6f**

($^{13}C\{^{1}H\}$ NMR, 101 MHz, $CDCl_3$)

el-4OMe-re_proton-1-2.jdf

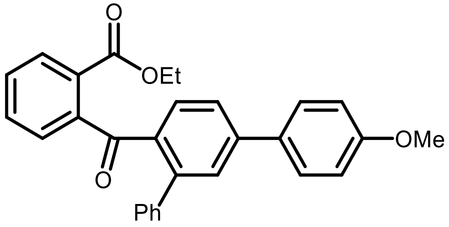

**6g**

($^{1}$H NMR, 400MHz, $CDCl_3$)

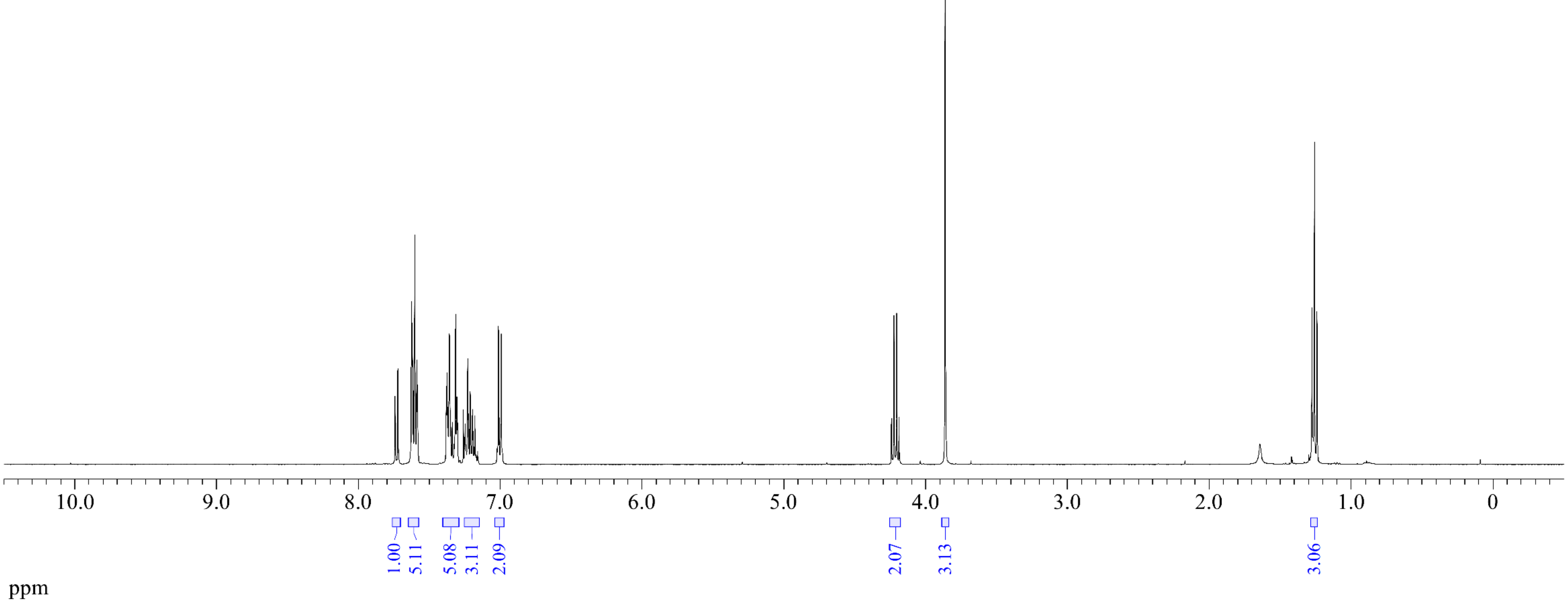

el-4OMe-re_carbon-1-2.jdf

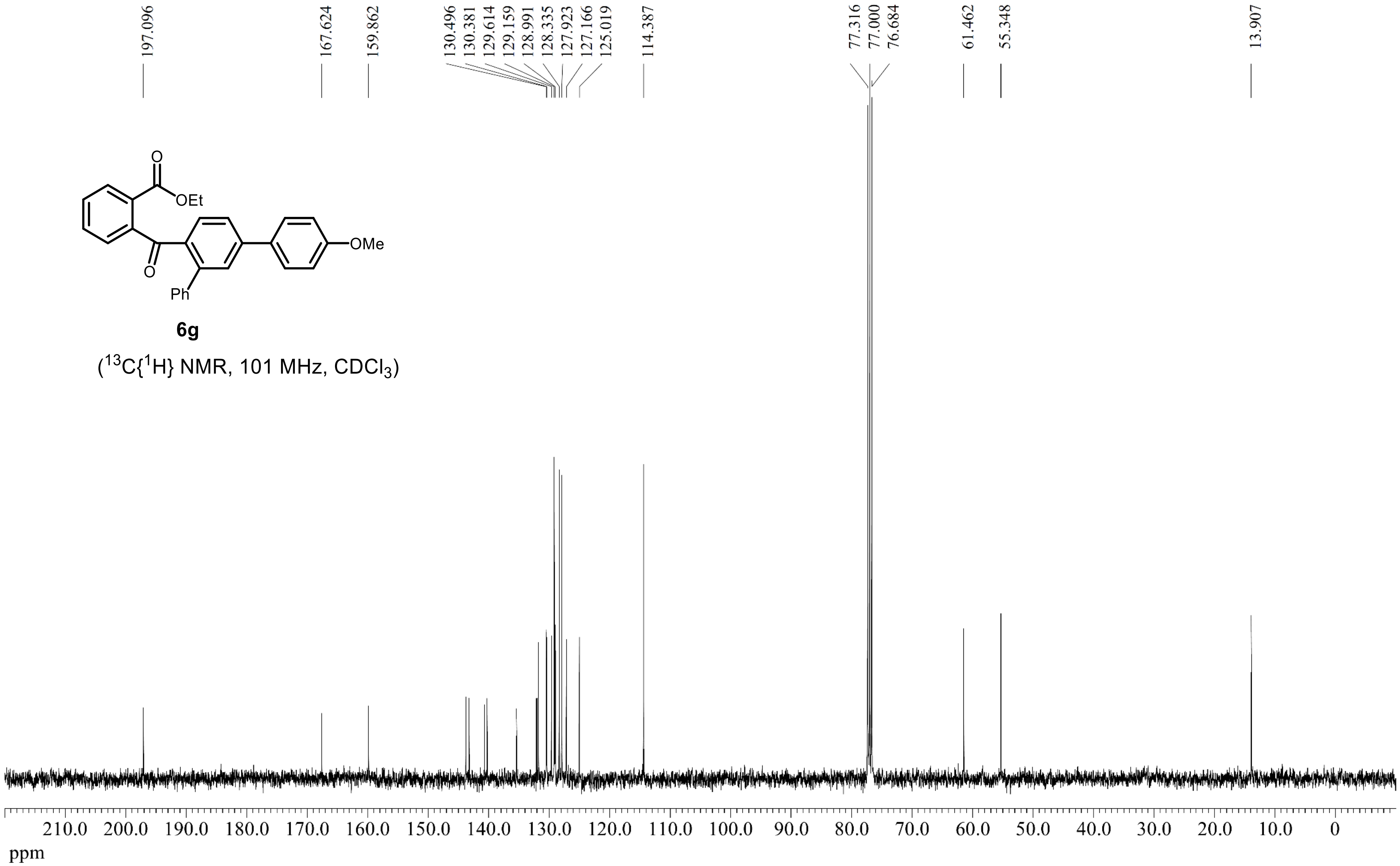


**6g**

($^{13}C\{^{1}H\}$ NMR, 101 MHz, $CDCl_3$)

el4Me-re_proton-1-2.jdf

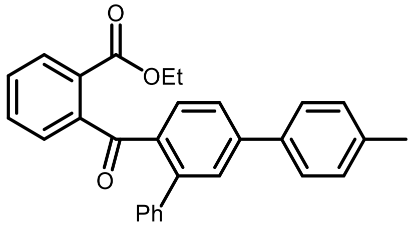


**6h**

($^{1}$H NMR, 400MHz, $CDCl_3$)

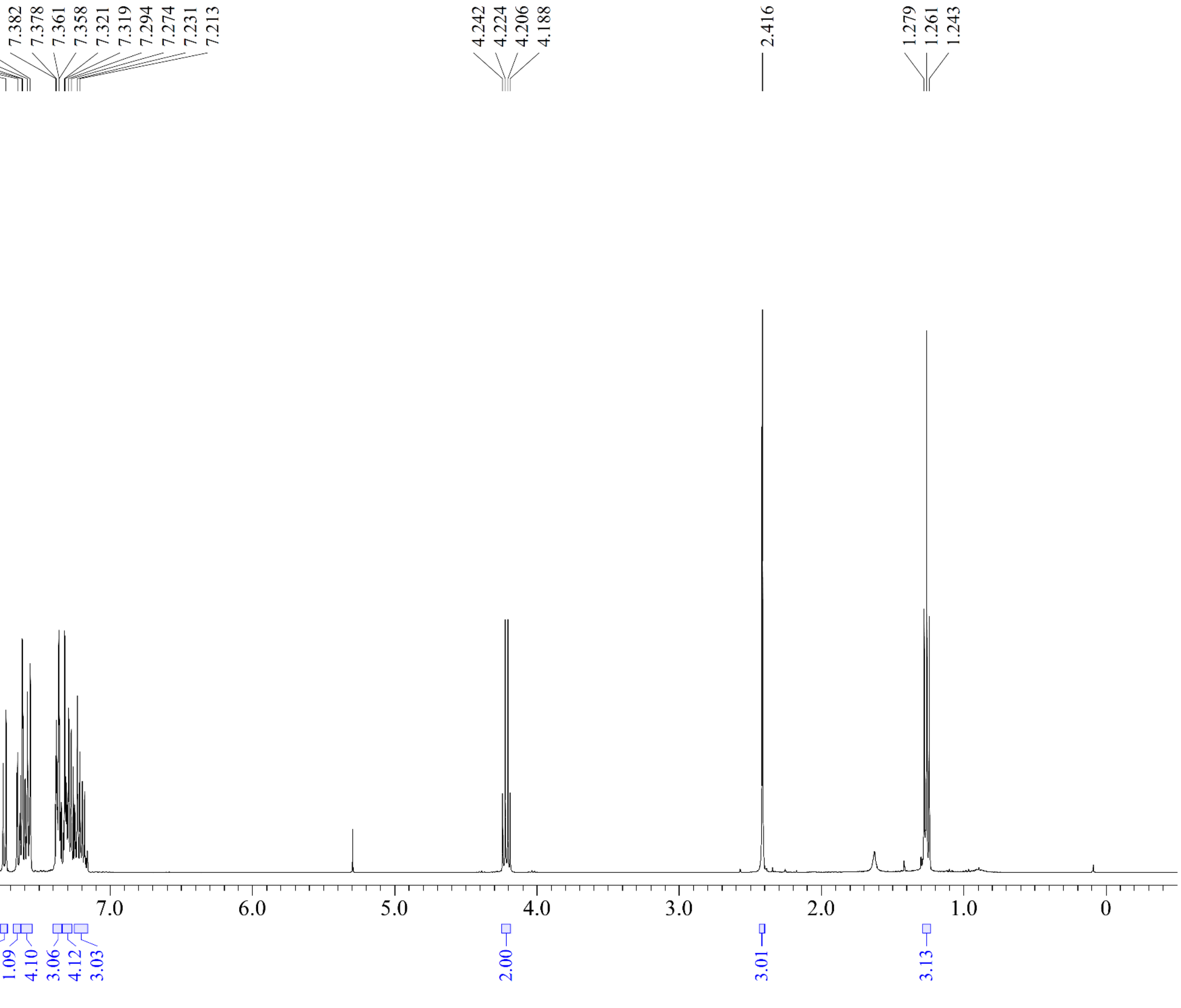

el4Me-re_carbon-1-2.jdf

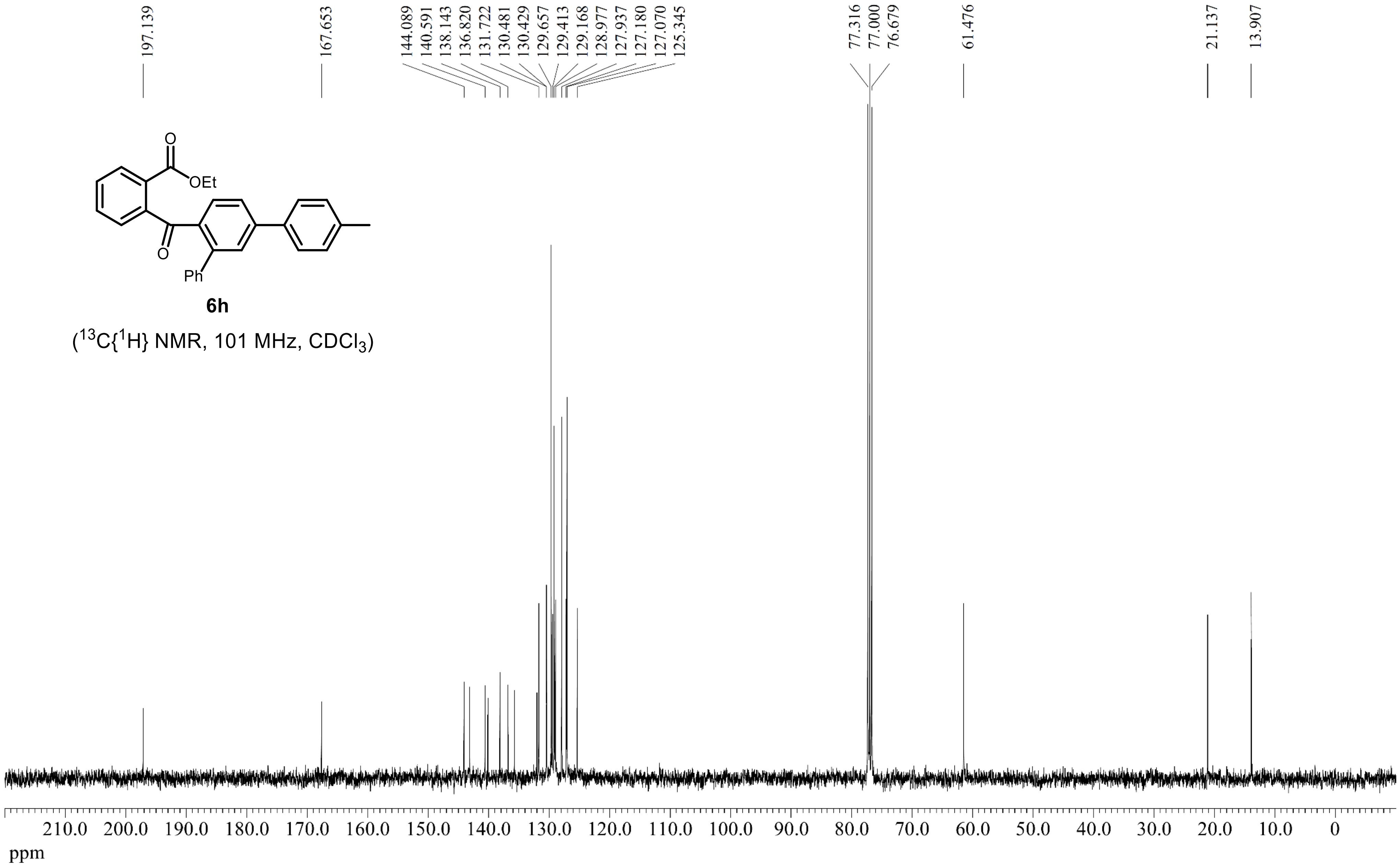

nu-2naph-re_proton-1-2.jdf 7.832 7.812 7.735 7.731 7.707 7.702 7.687 7.682 7.539 7.535 7.486 7.467 7.430 7.260 7.219 7.210 4.253 4.236 4.218 4.200 1.293 1.276 1.258

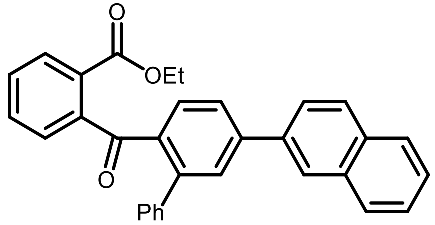


**6i**

($^1$H NMR, 400MHz, CDCl$_3$)

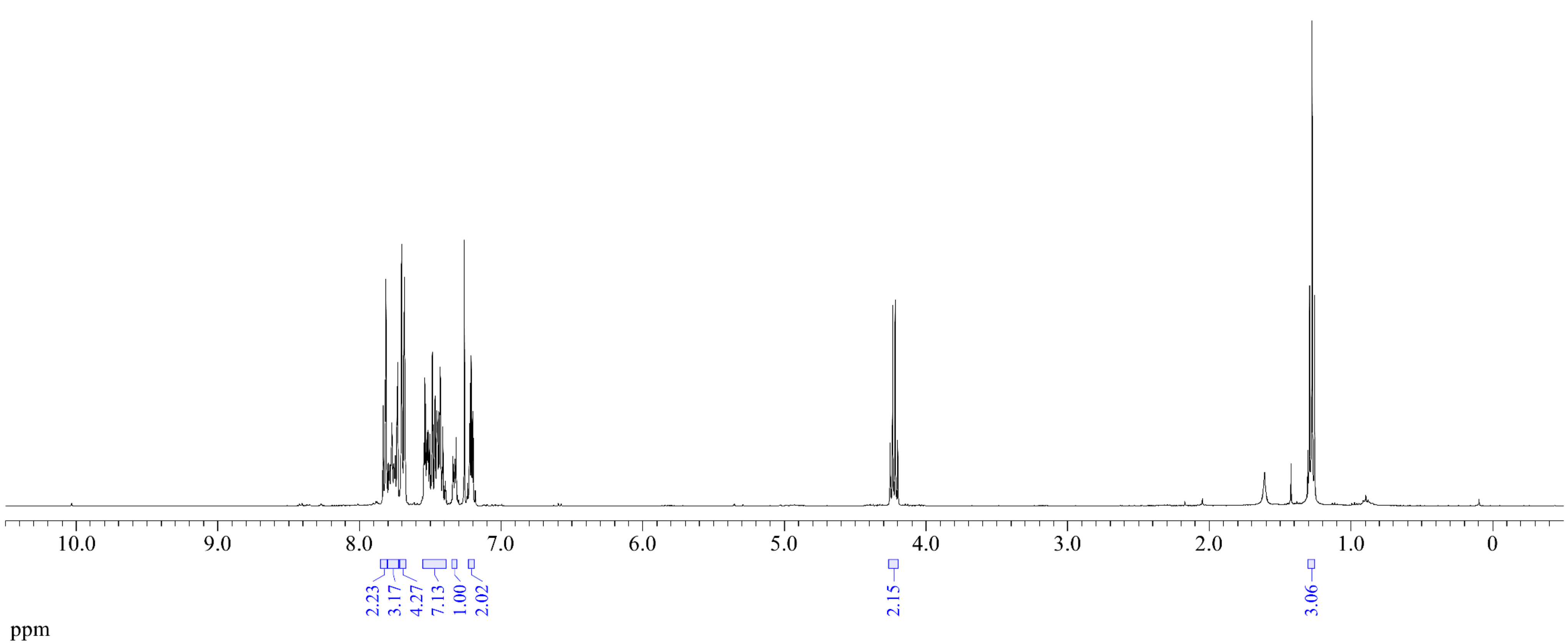

nu-2naph-re_carbon-1-2.jdf

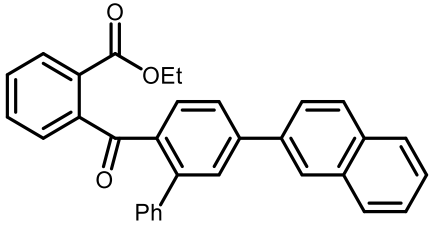


**6i**

($^{13}C\{^{1}H\}$ NMR, 101 MHz, $CDCl_3$)

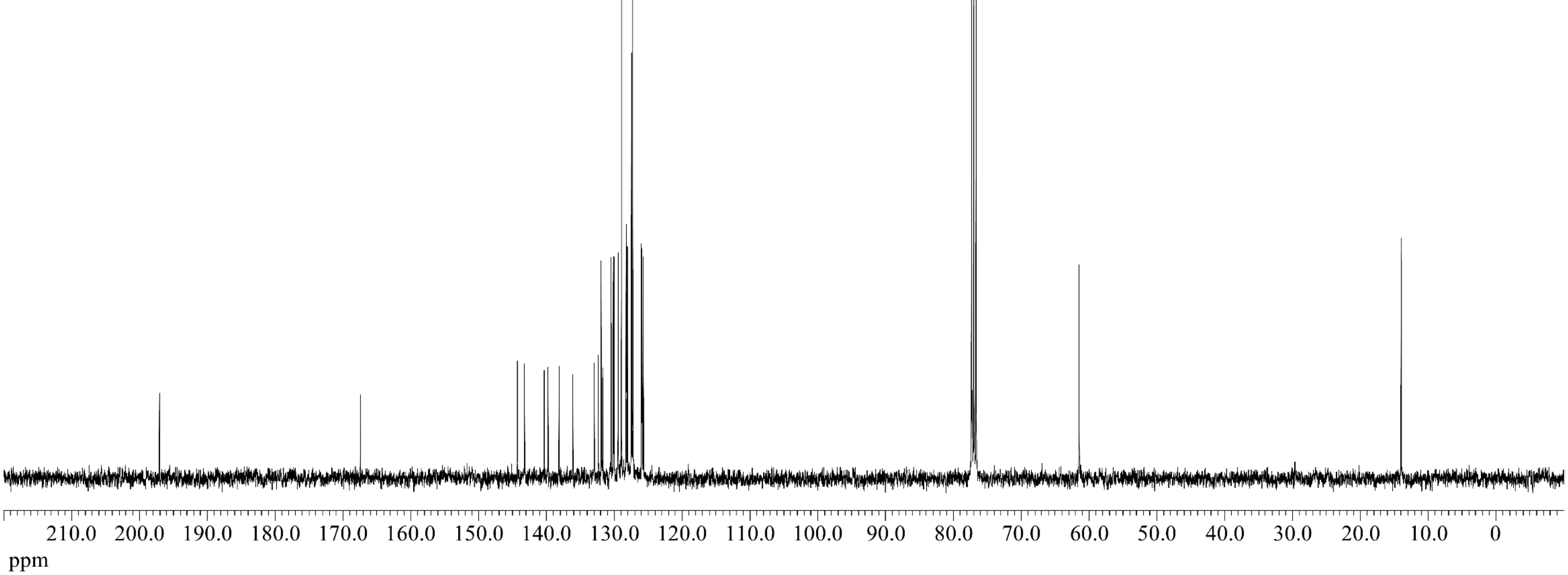

el-2thio-re_proton-1-2.jdf

7.686 7.669 7.665 7.624 7.619 7.372 7.364 7.359 7.356 7.343 7.340 7.297 7.293 7.260 7.233 7.215

4.233 4.215 4.197 4.180

1.615 1.418 1.297 1.277 1.259 1.241

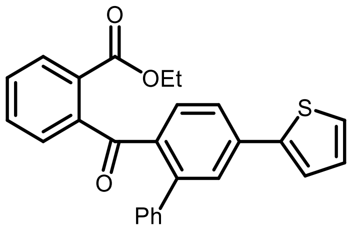


**6j**

($^{1}$H NMR, 400MHz, CDCl$_3$)

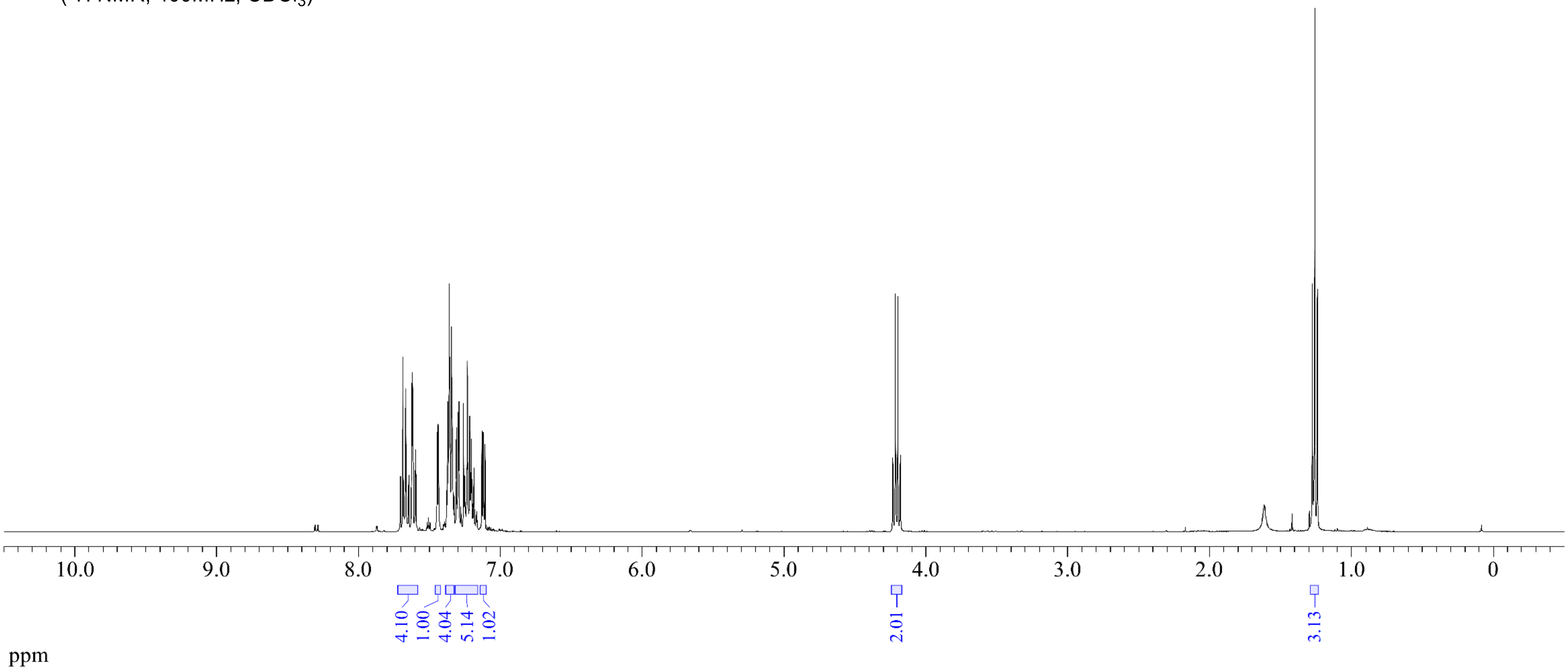

el-2thio-re_carbon-1-2.jdf

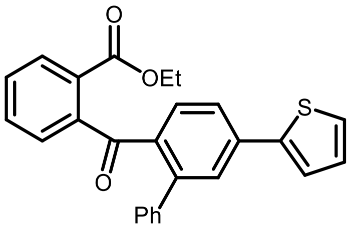

**6j**

($^{13}C\{^{1}H\}$ NMR, 101 MHz, $CDCl_3$)

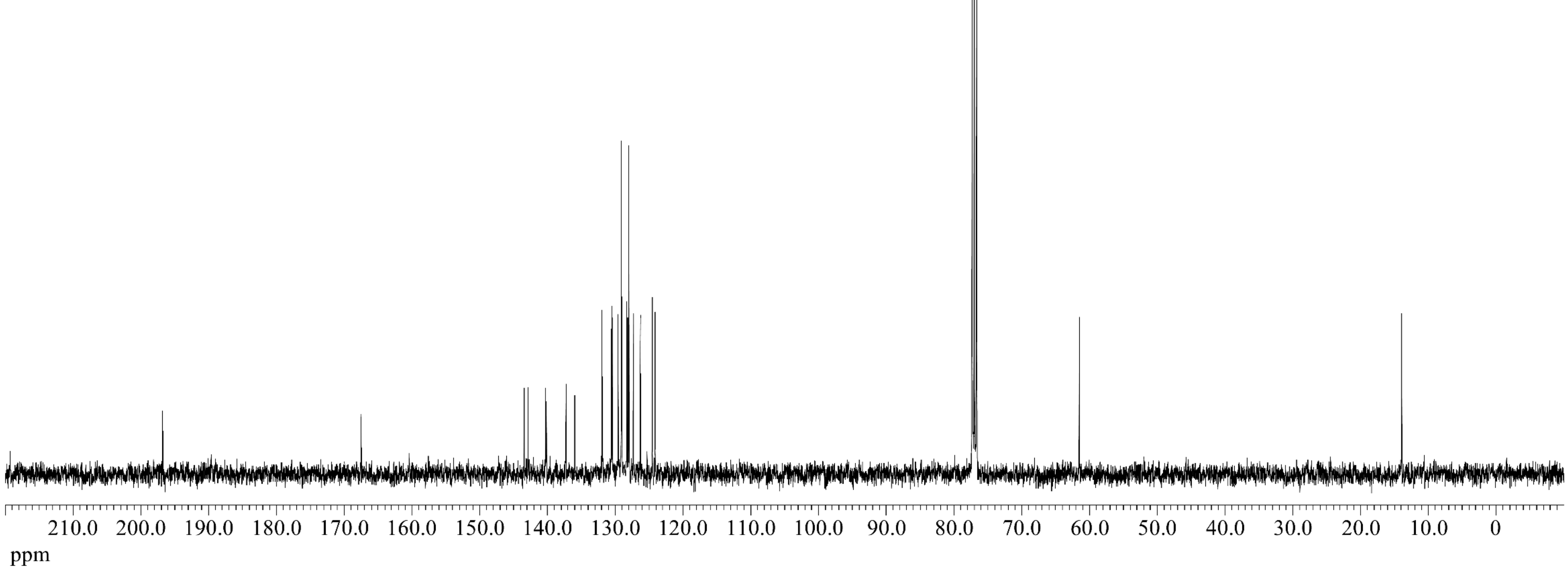

el-Et-re_proton-1-2.jdf

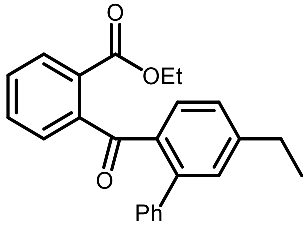

**6k**

($^1$H NMR, 400MHz, CDCl$_3$)

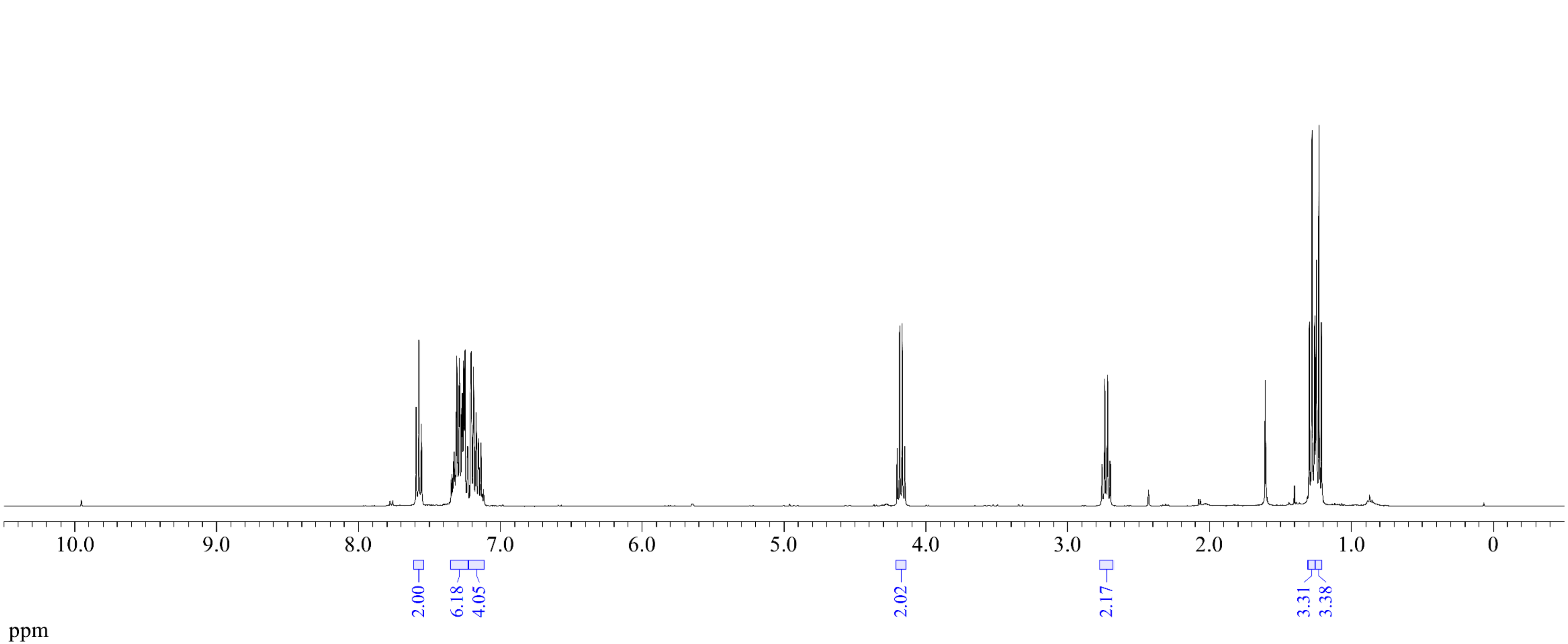

el-Et-re_carbon-1-2.jdf

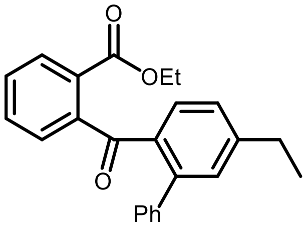

6k

($^{13}C\{^{1}H\}$ NMR, 101 MHz, $CDCl_3$)

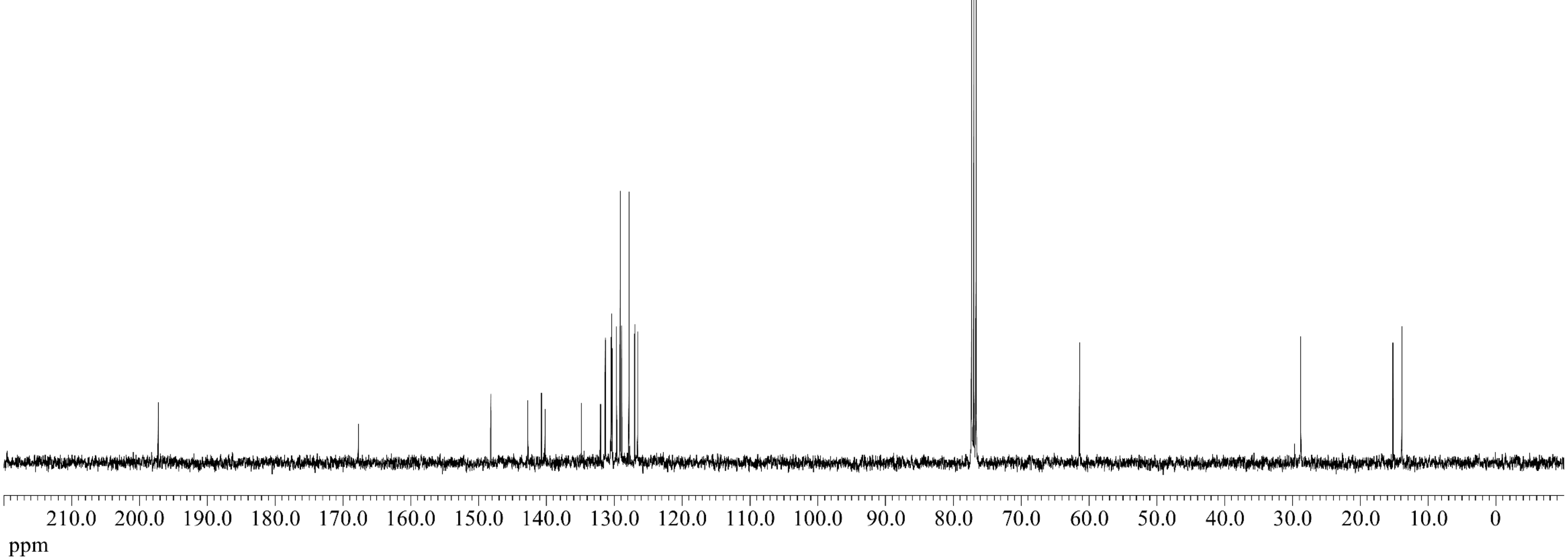

nu4F-re_proton-1-2.jdf

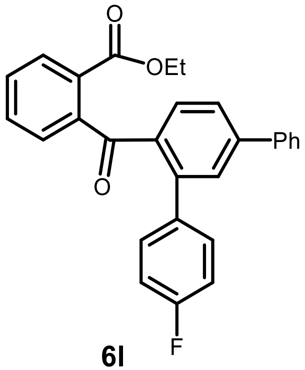

6l

($^1$H NMR, 400MHz, $CDCl_3$)

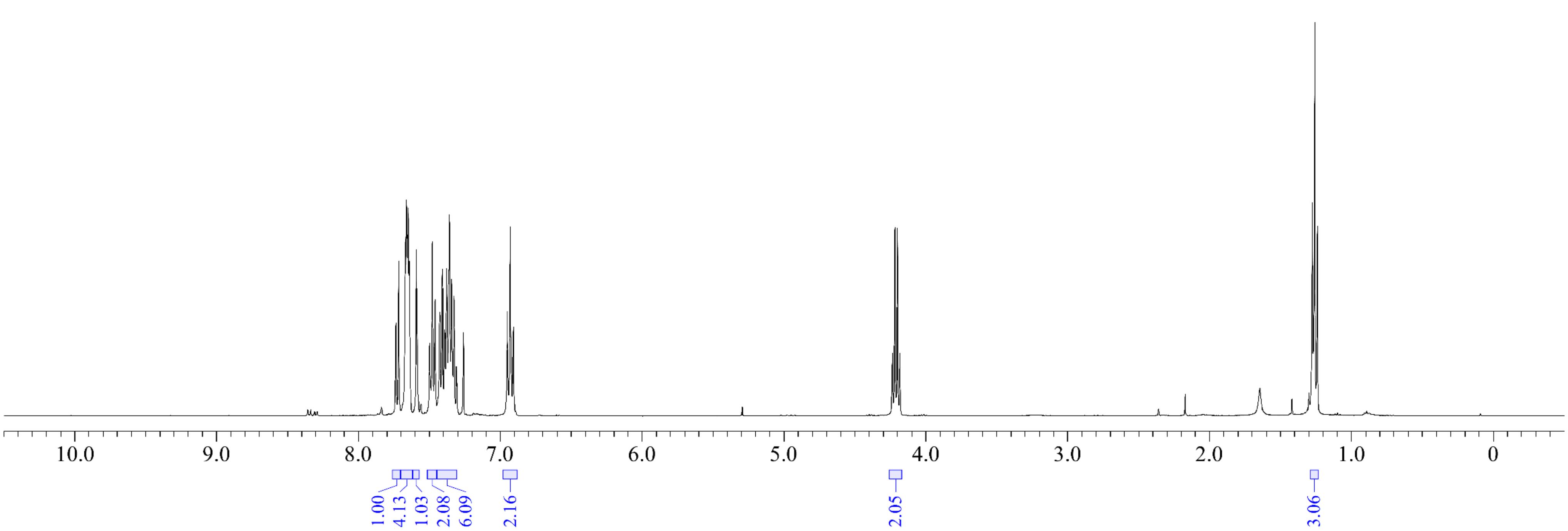

nu4F-re-OEt_carbon-1-2.jdf

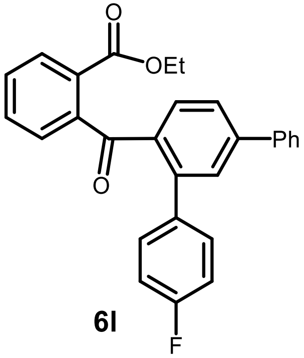

6l

($^{13}C\{^{1}H\}$ NMR, 101 MHz, $CDCl_3$)

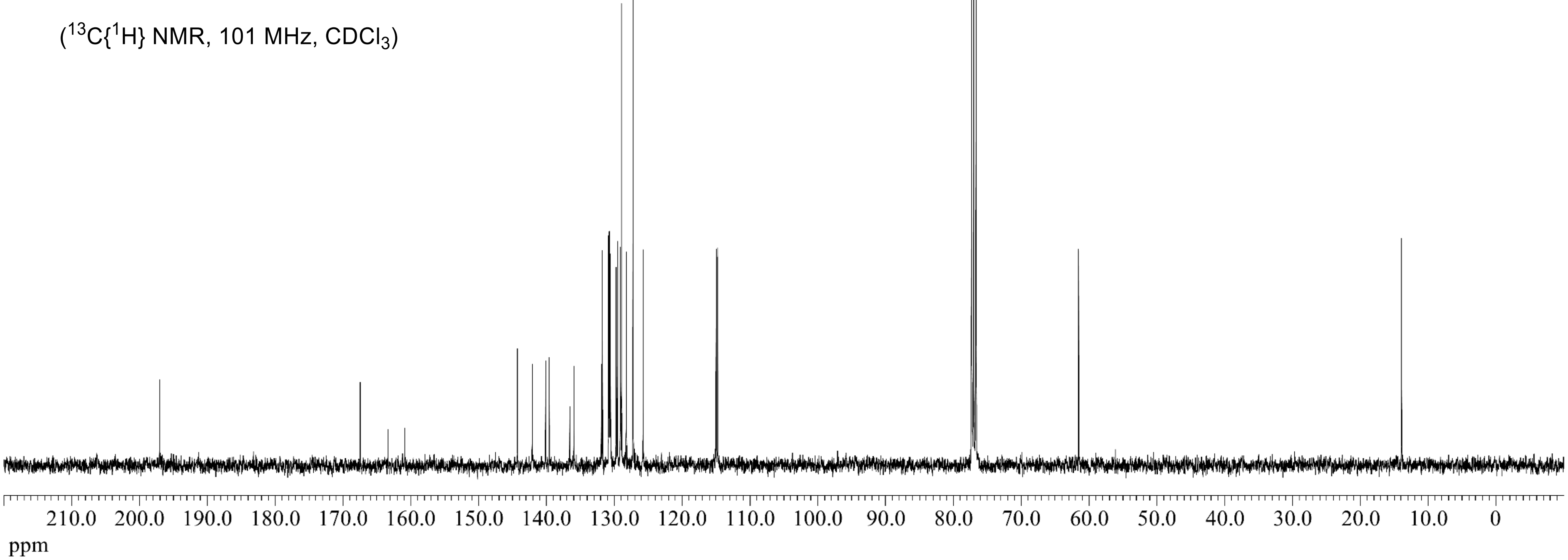

nu4F-re_19F_copy2-1-2.jdf

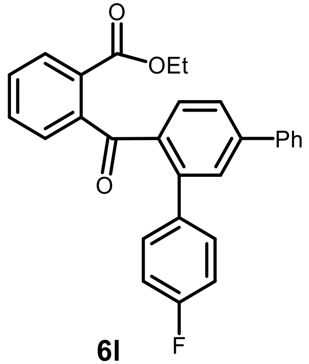

**6l**

($^{19}$F NMR, 376MHz, $CDCl_3$)

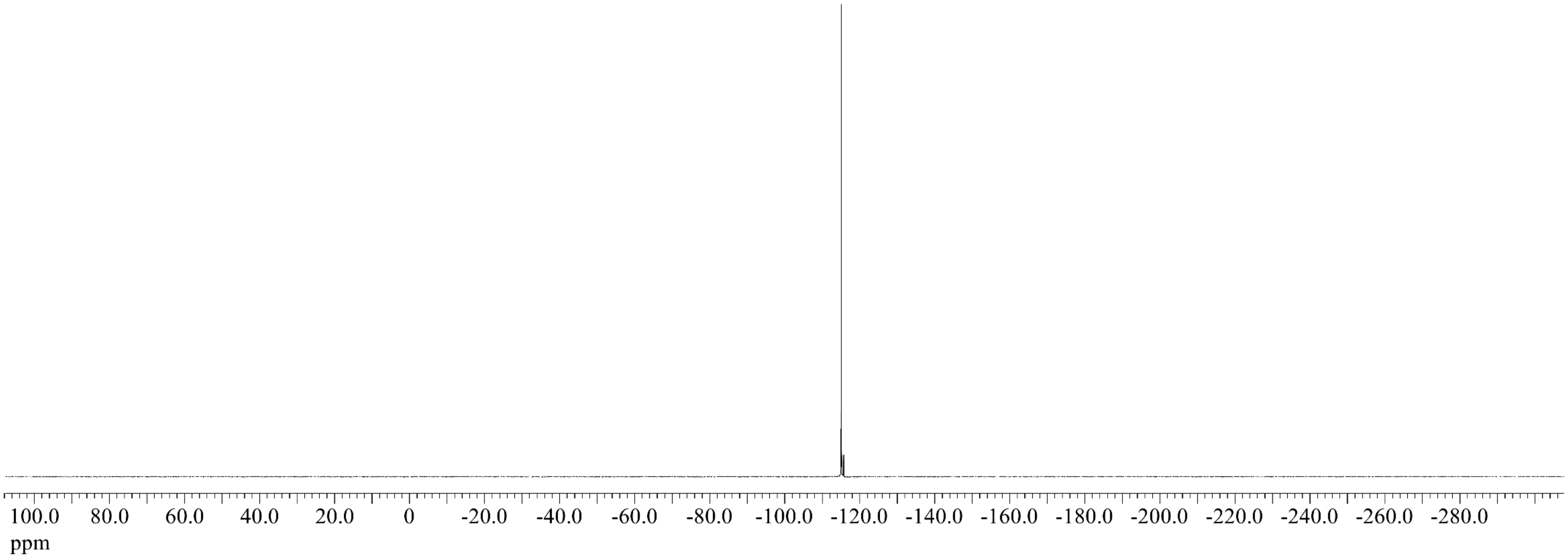

nuCl-re-check_proton-1-2.jdf

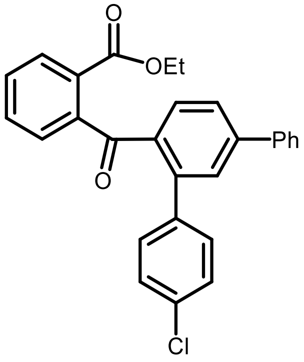


**6m**

($^{1}$H NMR, 400MHz, $CDCl_3$)

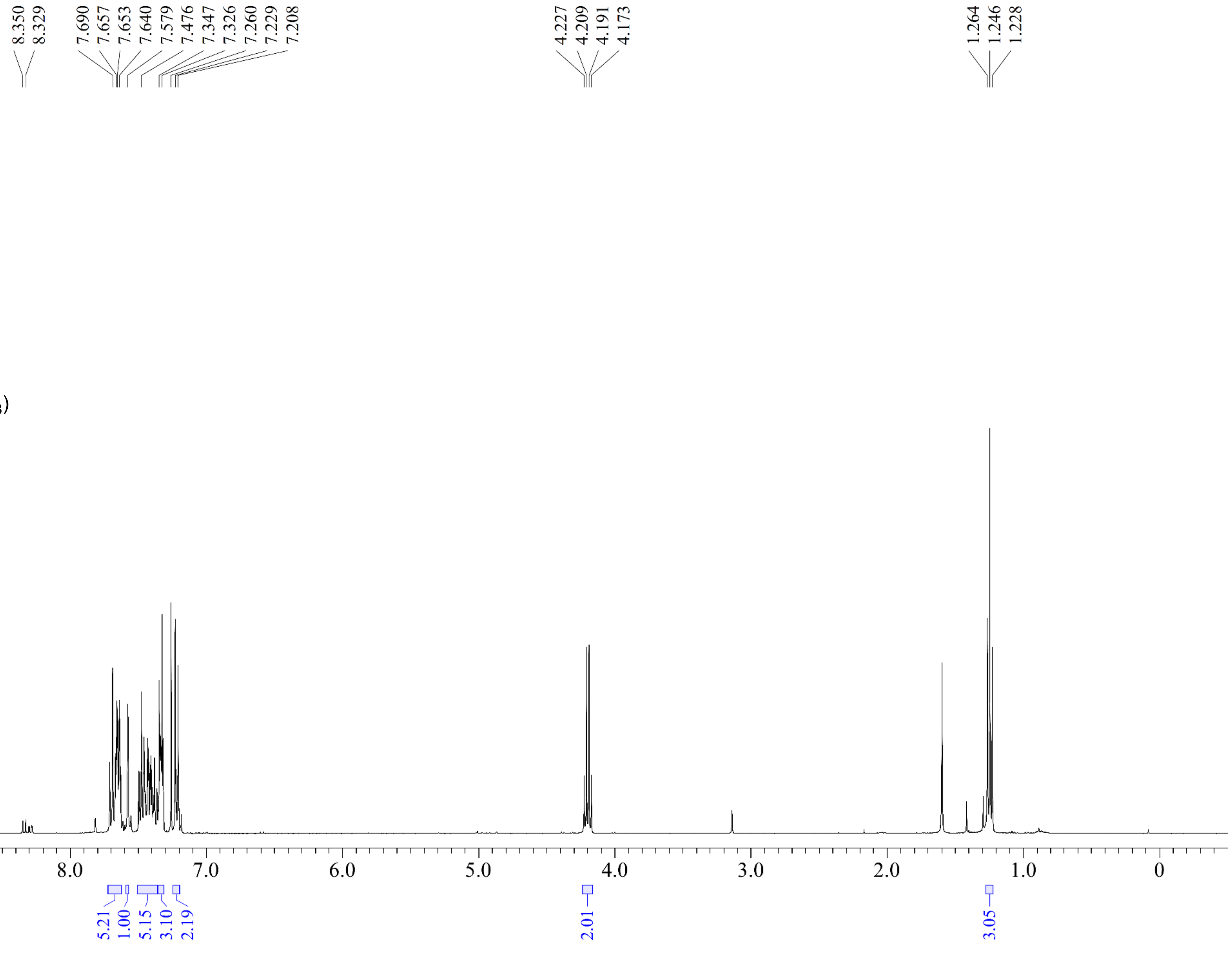

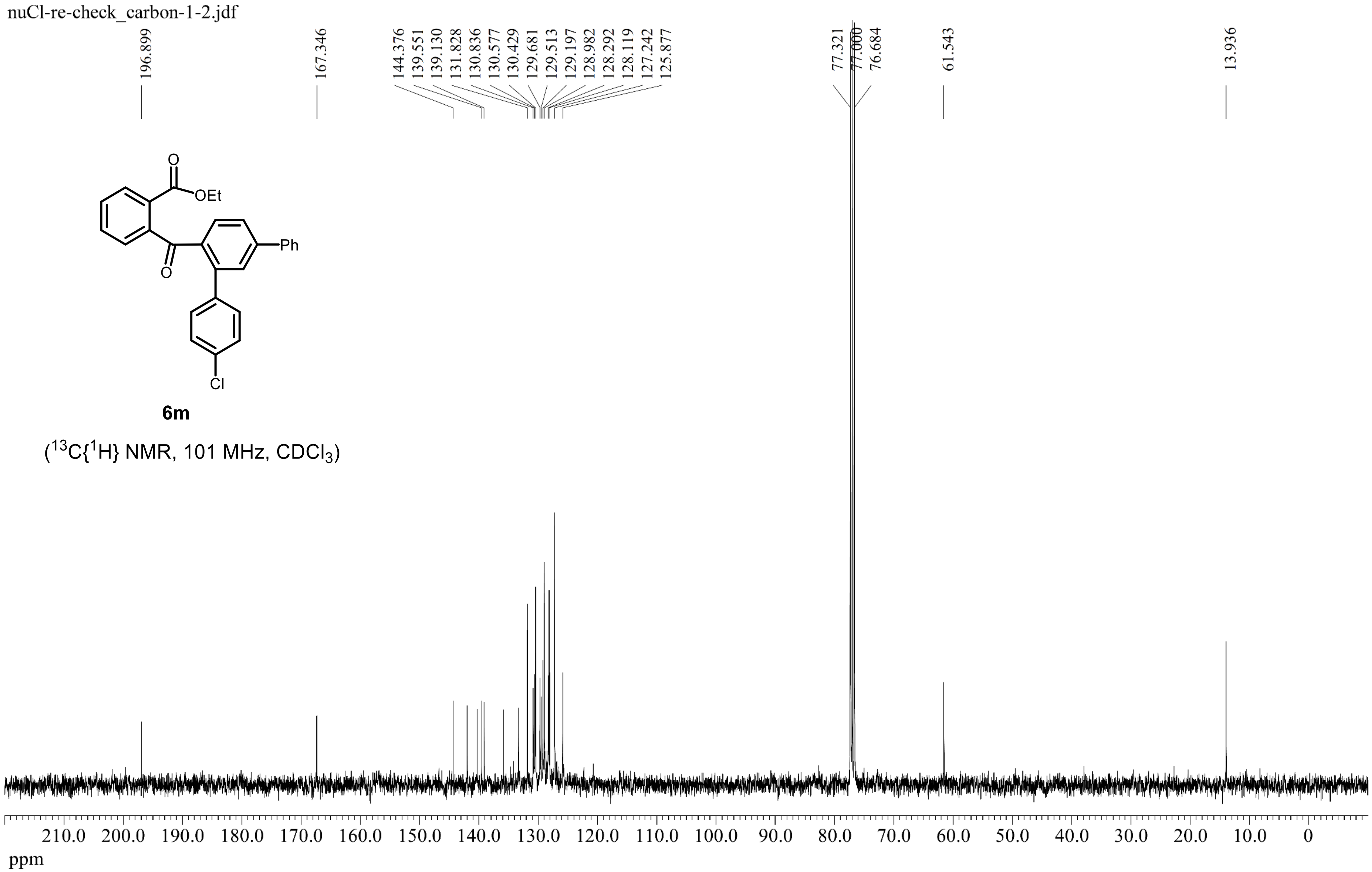
nuCl-re-check_carbon-1-2.jdf
196.899
167.346
144.376
139.551
139.130
131.828
130.836
130.577
130.429
129.681
129.513
129.197
128.982
128.292
128.119
127.242
125.877
77.321
77.000
76.684
61.543
13.936
O
OEt
Ph
O
Cl
6m
($^{13}$C{$^{1}$H} NMR, 101 MHz, $CDCl_3$)
210.0
200.0
190.0
180.0
170.0
160.0
150.0
140.0
130.0
120.0
110.0
100.0
90.0
80.0
70.0
60.0
50.0
40.0
30.0
20.0
10.0
0
ppm

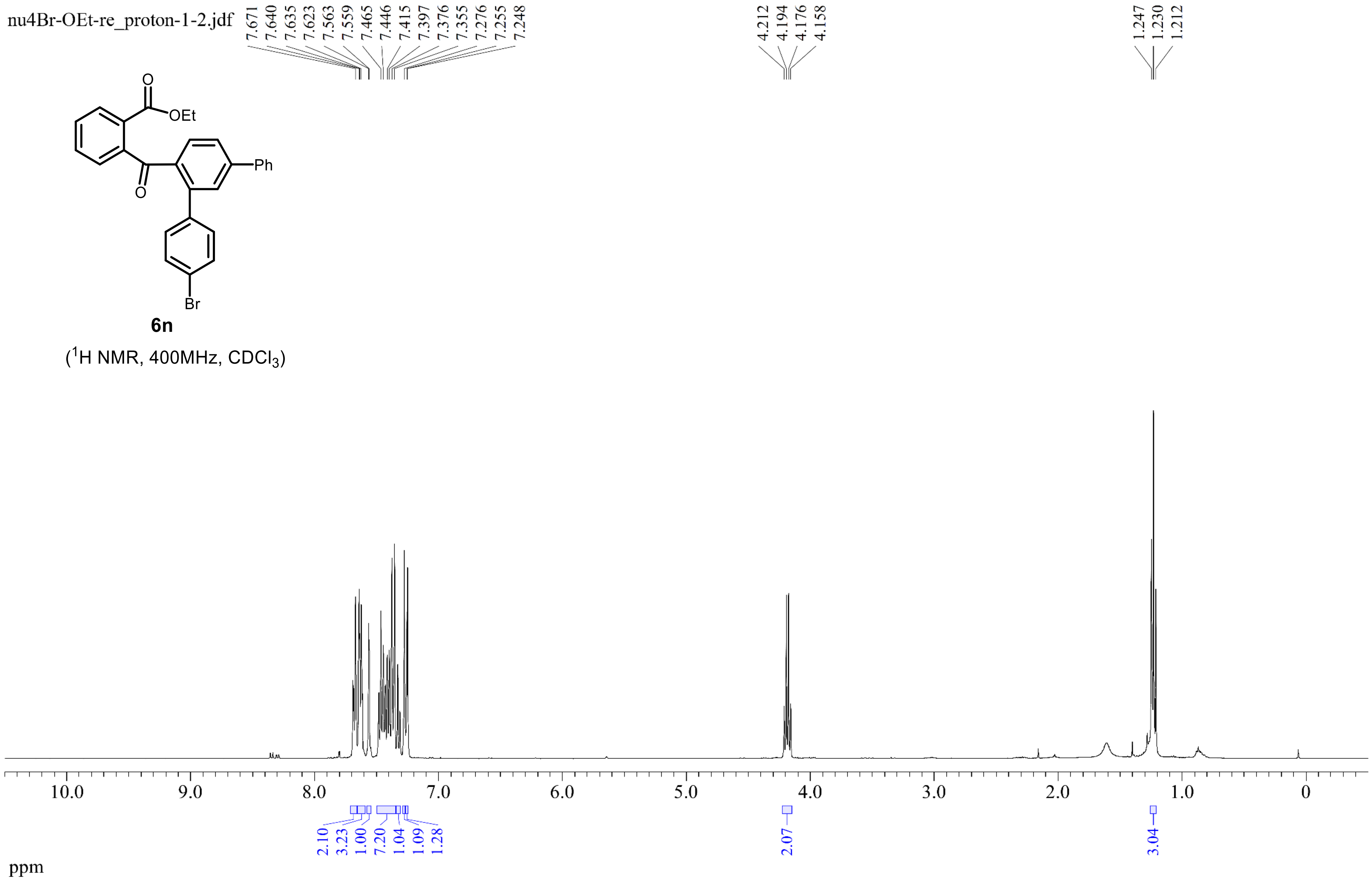


**6n**

($^{1}$H NMR, 400MHz, $CDCl_3$)

nu4Br-plz_carbon-1-2.jdf

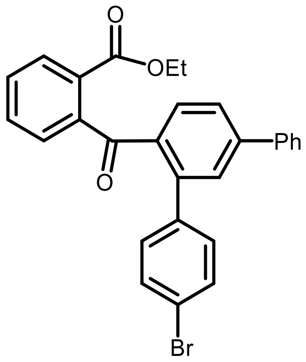


**6n**

($^{13}C\{^{1}H\}$ NMR, 101 MHz, $CDCl_3$)

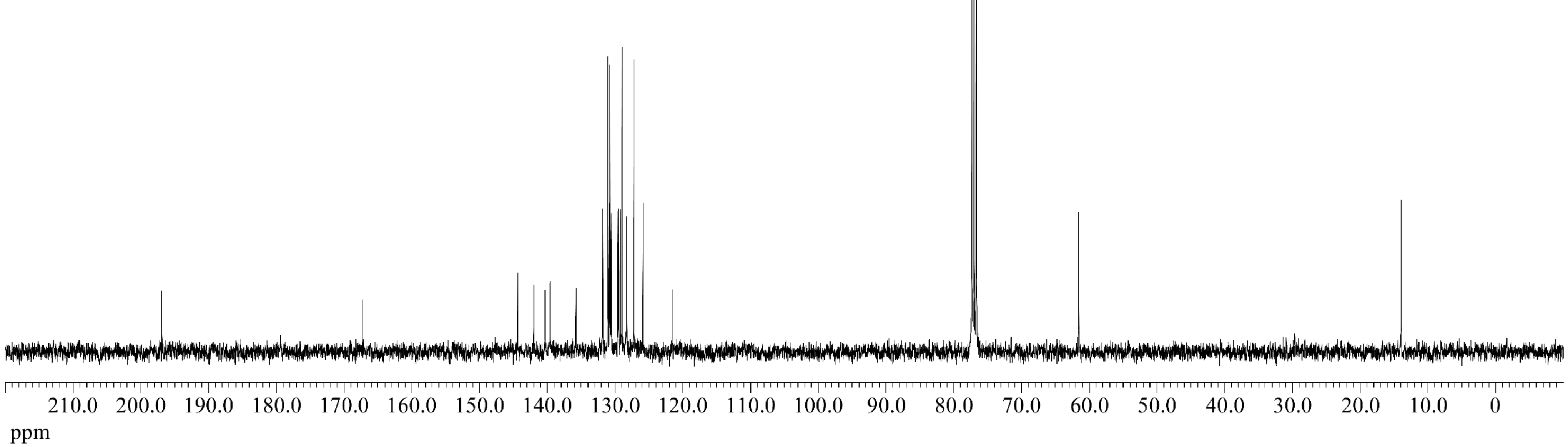

nuOMe-reoet_proton-1-2.jdf

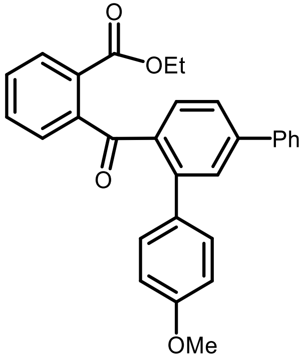


**6o**

($^1$H NMR, 400MHz, $CDCl_3$)

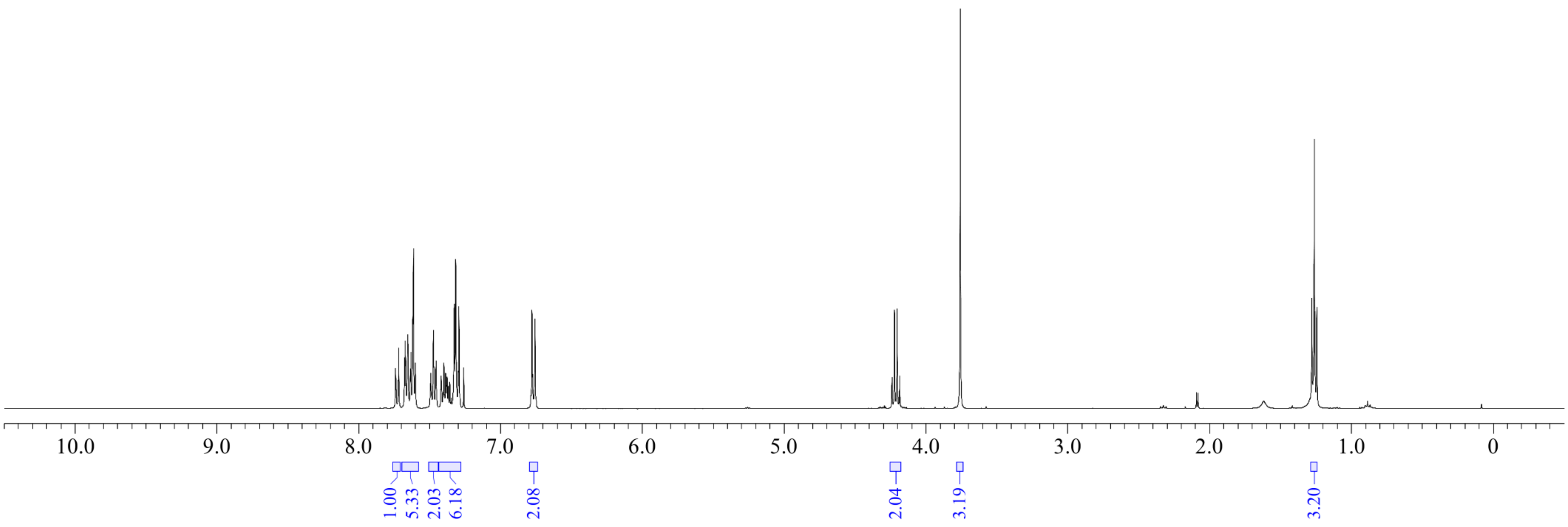

nuOMe-reoet_carbon-1-2.jdf

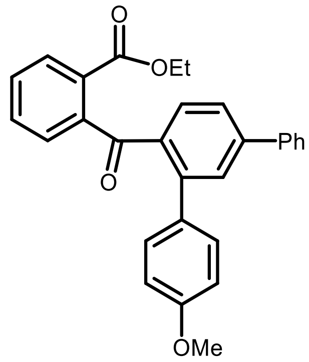


**6o**

($^{13}C\{^{1}H\}$ NMR, 101 MHz, $CDCl_3$)

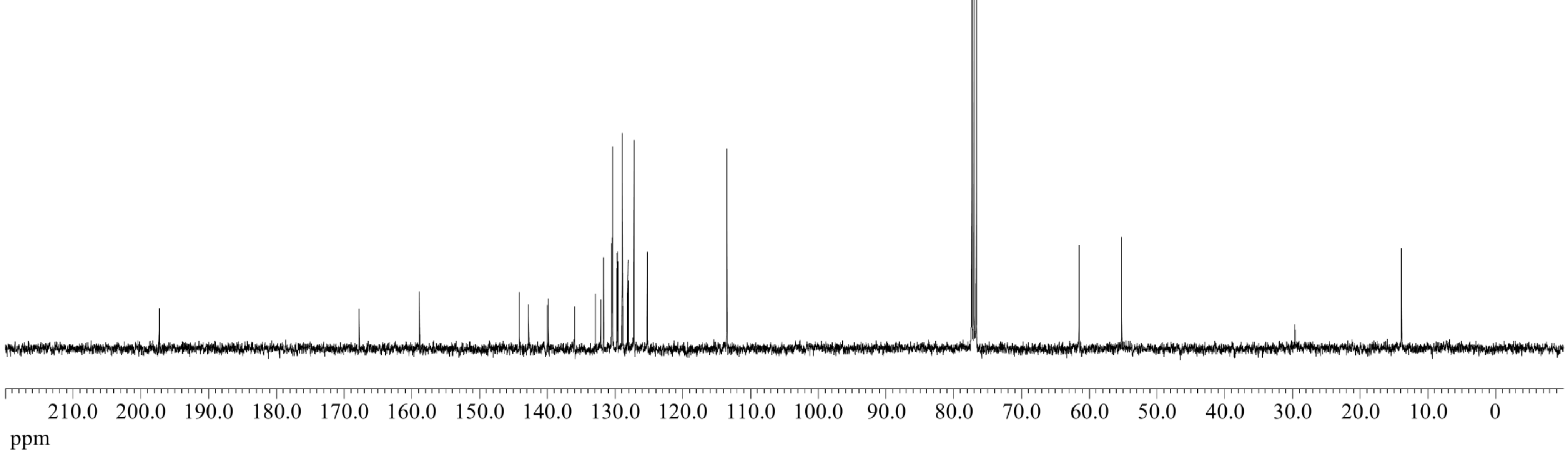

nu-2naph-re_proton-1-2.jdf

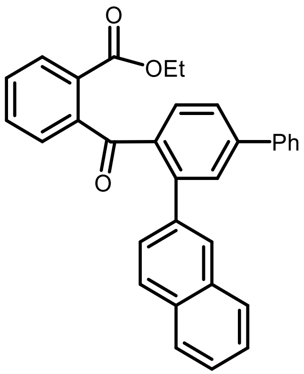


**6p**

($^{1}$H NMR, 400MHz, $CDCl_3$)

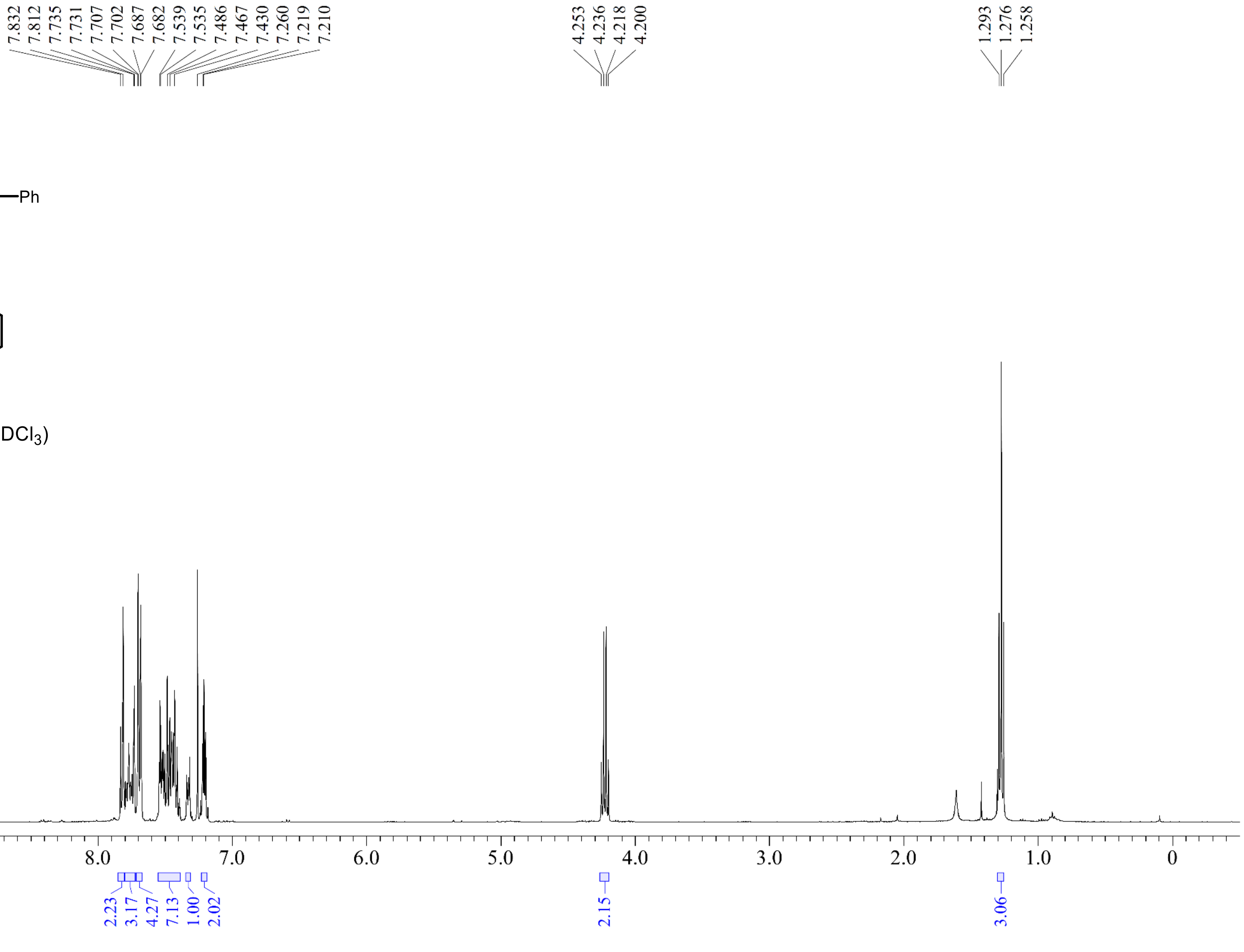

nu-2naph-re_carbon-1-2.jdf

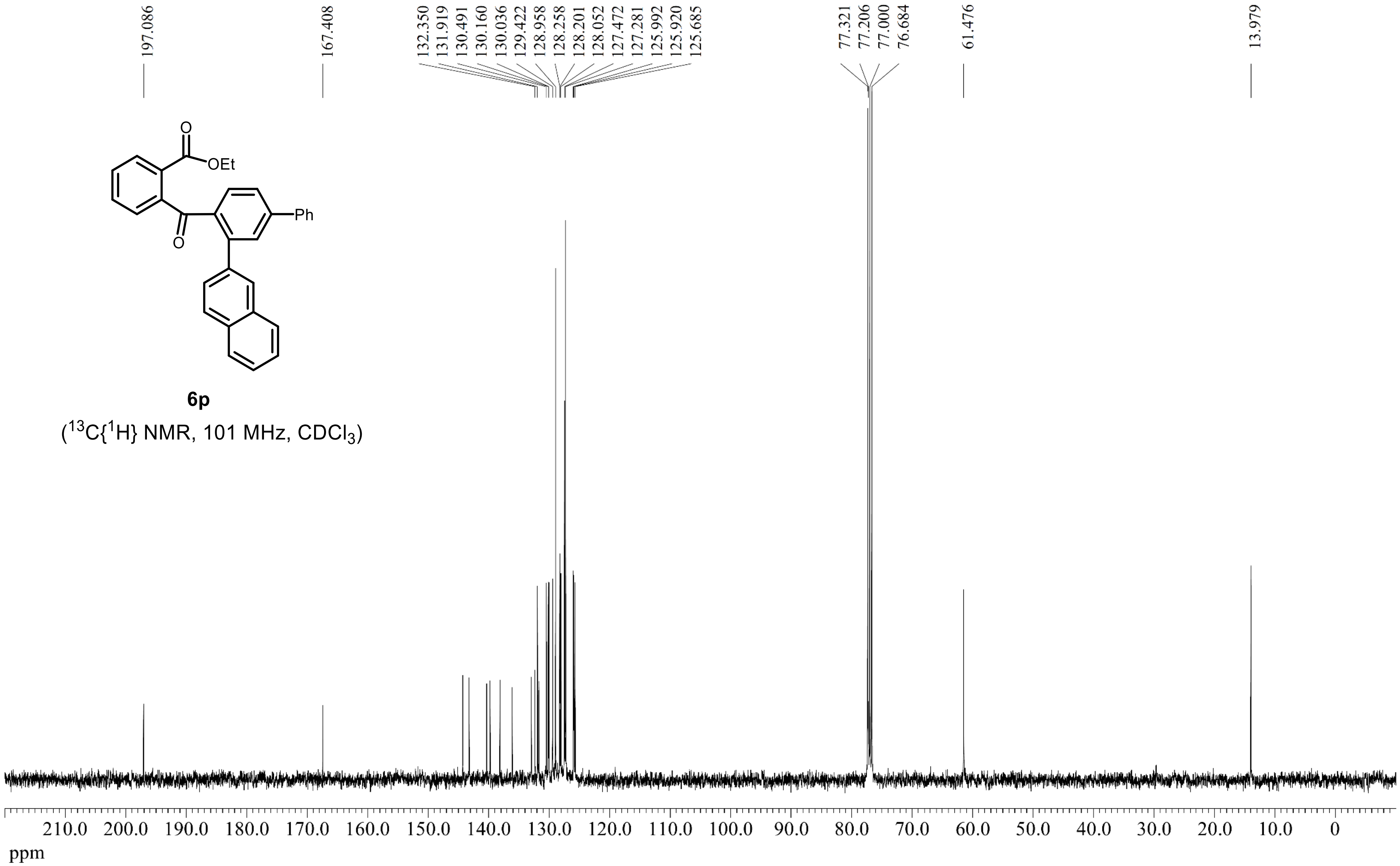


**6p**
($^{13}C\{^{1}H\}$ NMR, 101 MHz, $CDCl_3$)

nu2S-re_proton-1-2.jdf

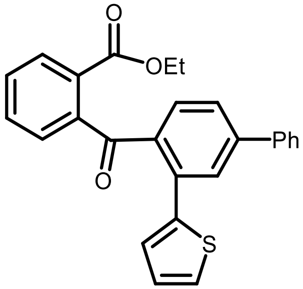


**6q**

($^{1}H$ NMR, 400MHz, $CDCl_3$)

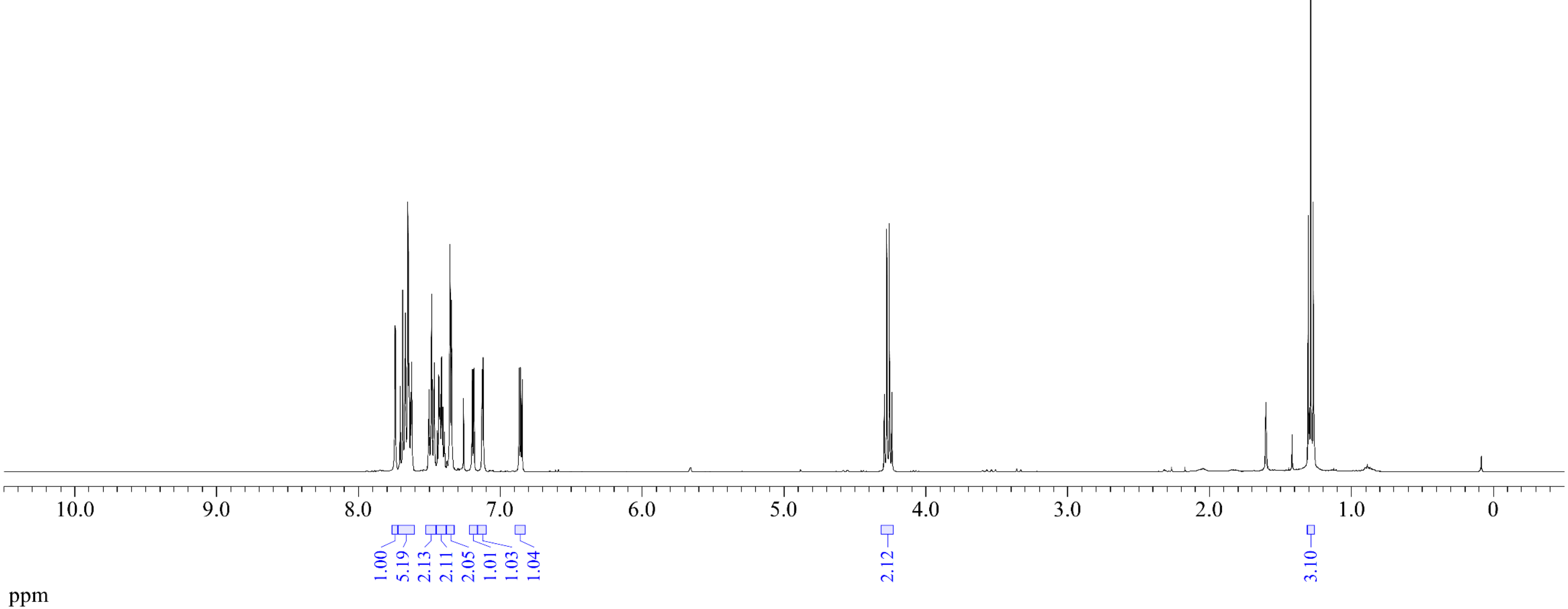

nu2S-re_carbon-1-2.jdf

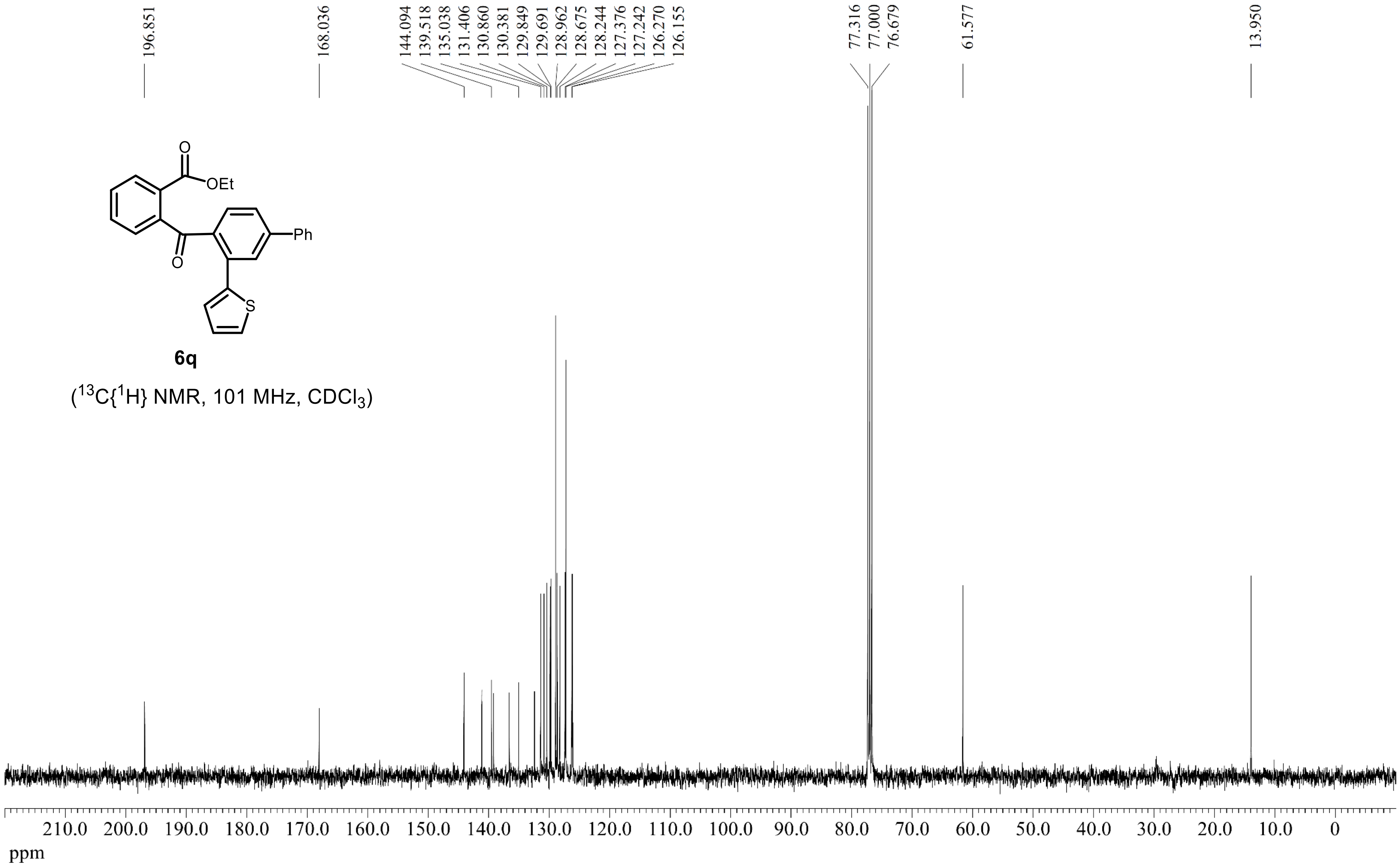


**6q**

($^{13}C\{^{1}H\}$ NMR, 101 MHz, $CDCl_3$)

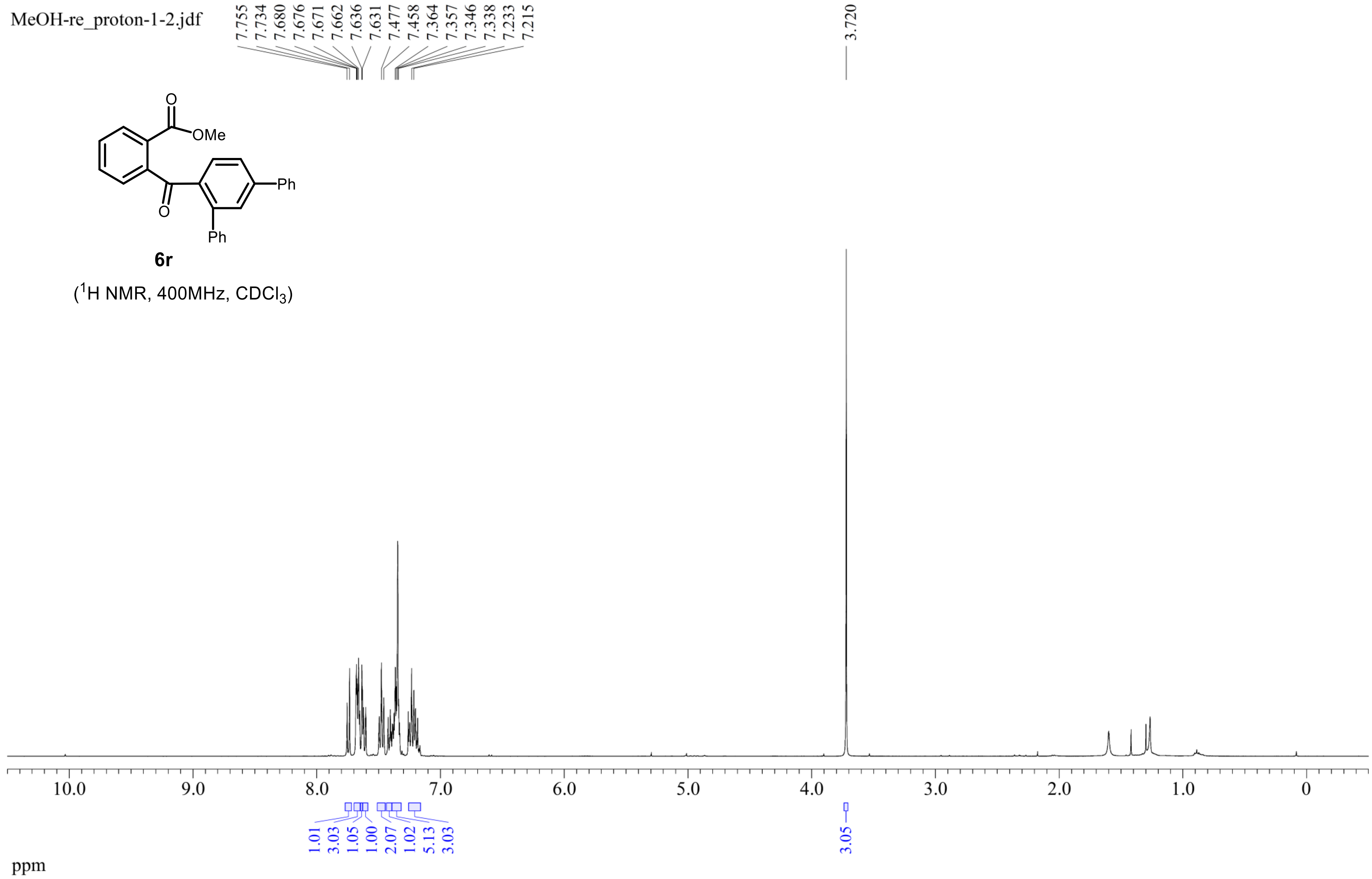
MeOH-re_proton-1-2.jdf
7.755
7.734
7.680
7.676
7.671
7.662
7.636
7.631
7.477
7.458
7.364
7.357
7.346
7.338
7.233
7.215
3.720
O
OMe
Ph
O
Ph
6r
($^1$H NMR, 400MHz, CDCl$_3$)
10.0
9.0
8.0
7.0
6.0
5.0
4.0
3.0
2.0
1.0
0
1.01
3.03
1.05
1.00
2.07
1.02
5.13
3.03
3.05
ppm

MeOH-re_carbon-1-2.jdf

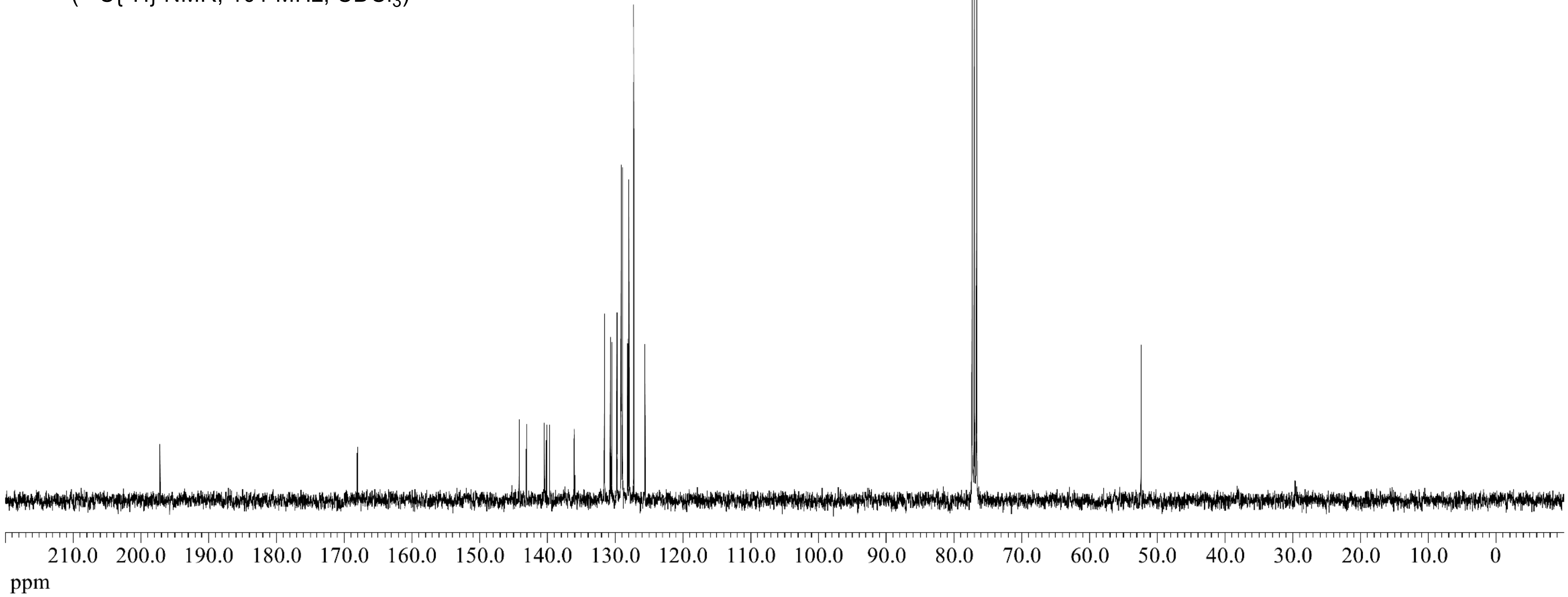


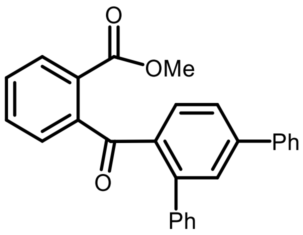


**6r**

($^{13}C\{^{1}H\}$ NMR, 101 MHz, $CDCl_3$)

nBu-re_proton-1-2.jdf

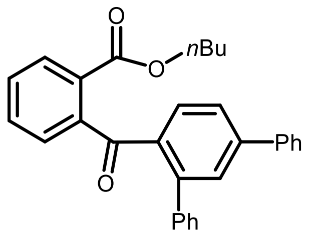

**6s**

($^{1}$H NMR, 400MHz, $CDCl_3$)

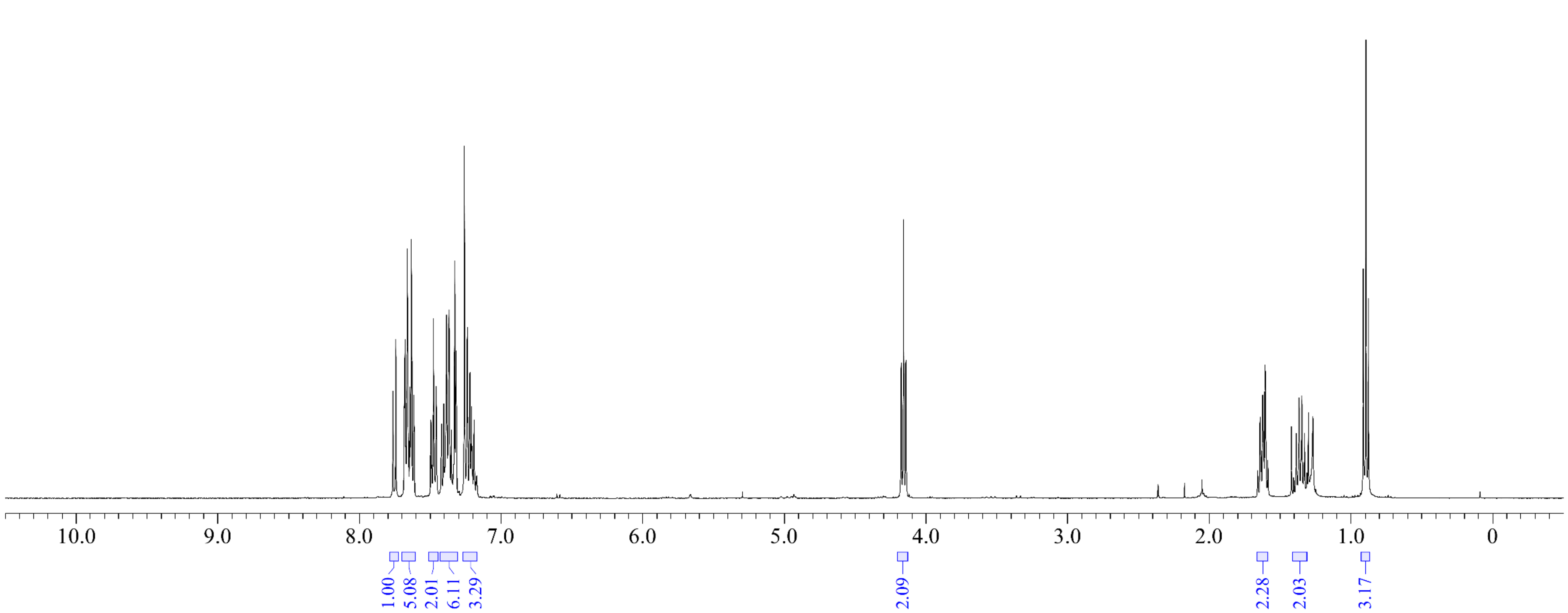

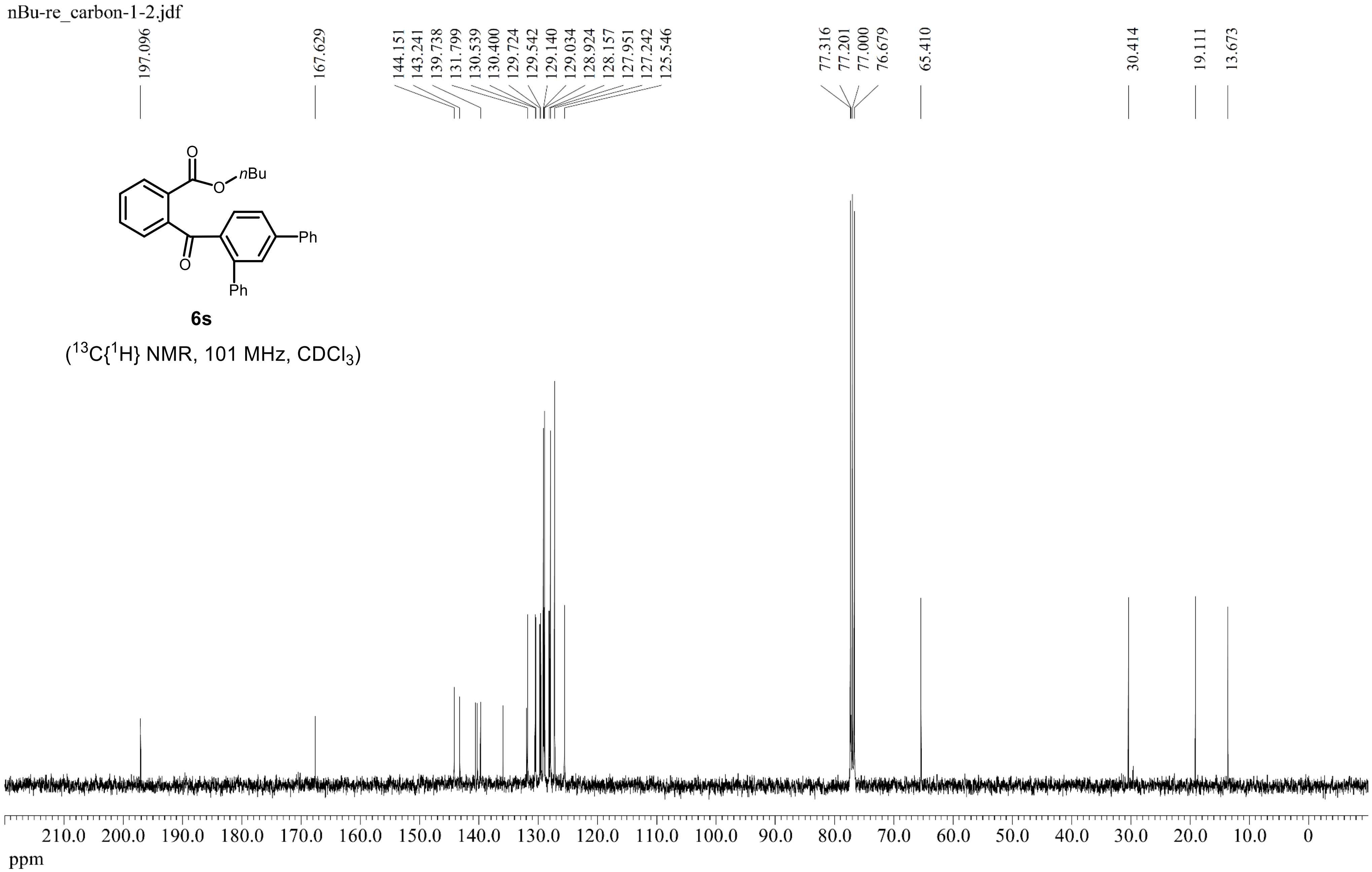
nBu-re_carbon-1-2.jdf
197.096
167.629
144.151
143.241
139.738
131.799
130.539
130.400
129.724
129.542
129.140
129.034
128.924
128.157
127.951
127.242
125.546
77.316
77.201
77.000
76.679
65.410
30.414
19.111
13.673
O
O
nBu
Ph
O
Ph
6s
($^{13}$C{$^{1}$H} NMR, 101 MHz, CDCl$_3$)
210.0
200.0
190.0
180.0
170.0
160.0
150.0
140.0
130.0
120.0
110.0
100.0
90.0
80.0
70.0
60.0
50.0
40.0
30.0
20.0
10.0
0
ppm

IPA-re-chec_proton-1-2.jdf

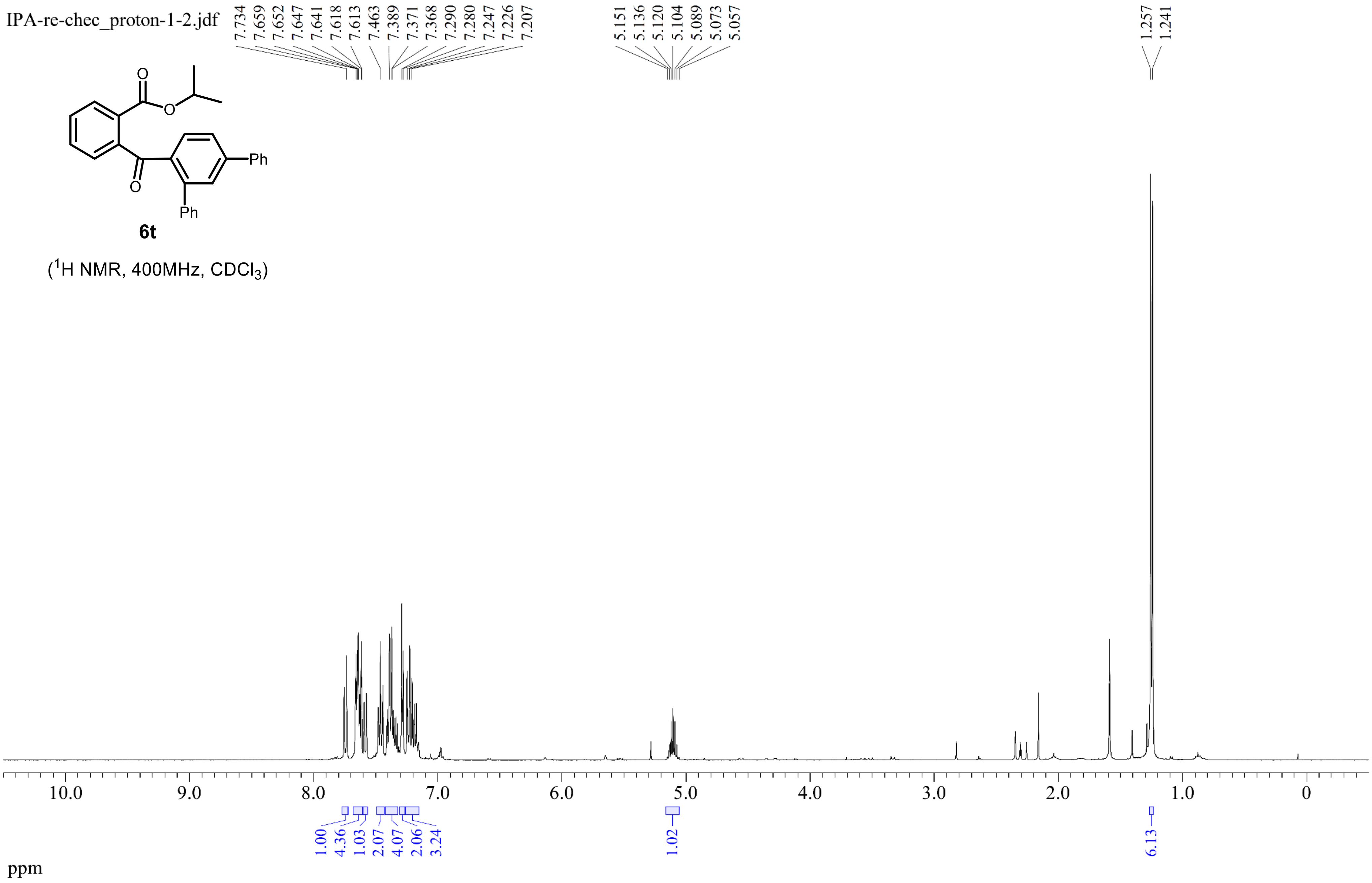


**6t**

($^1$H NMR, 400MHz, $CDCl_3$)

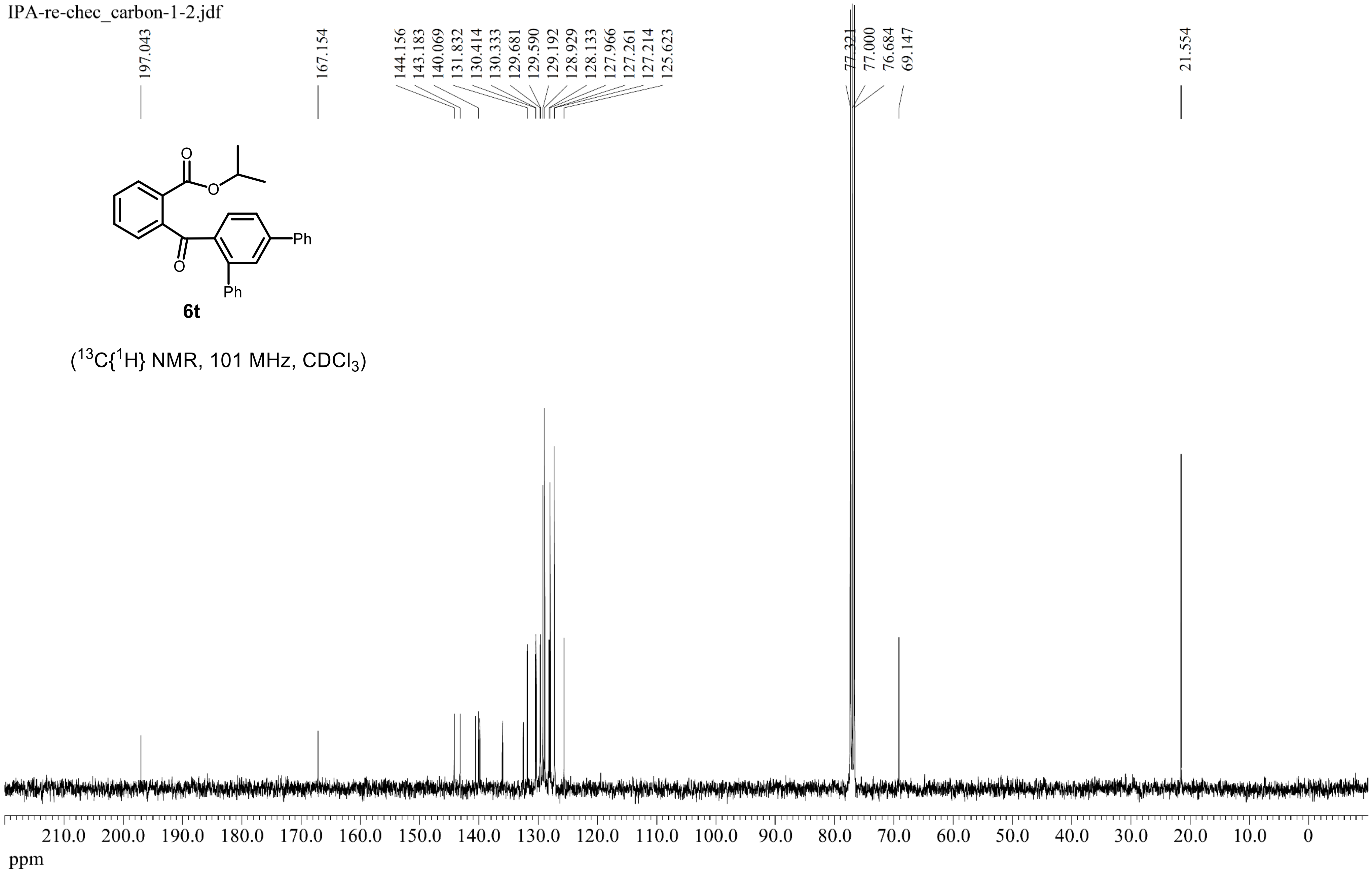
IPA-re-chec_carbon-1-2.jdf
197.043
167.154
144.156
143.183
140.069
131.832
130.414
130.333
129.681
129.590
129.192
128.929
128.133
127.966
127.261
127.214
125.623
77.321
77.000
76.684
69.147
21.554
O
O
O
Ph
Ph
6t
($^{13}$C{$^{1}$H} NMR, 101 MHz, $CDCl_3$)
210.0 200.0 190.0 180.0 170.0 160.0 150.0 140.0 130.0 120.0 110.0 100.0 90.0 80.0 70.0 60.0 50.0 40.0 30.0 20.0 10.0 0
ppm

J-F nBuOH_proton-3-2.jdf

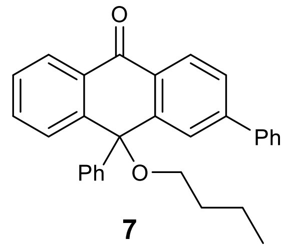


**7**

($^1$H NMR, 400MHz, $CDCl_3$)

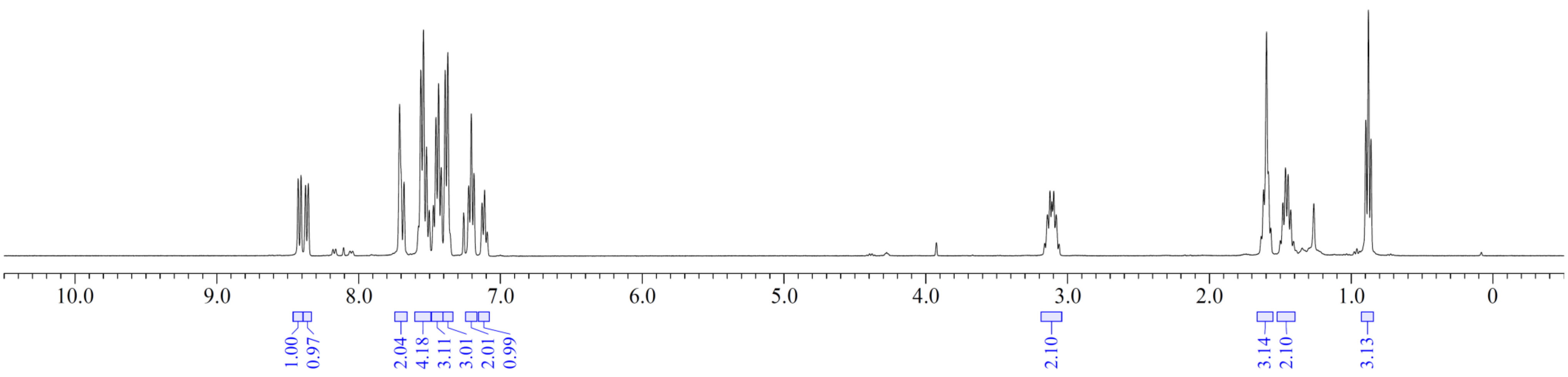

J-F nBuOH_carbon_copy1-1-2.jdf

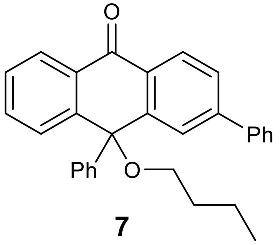

**7**

($^{13}C\{^{1}H\}$ NMR, 101 MHz, $CDCl_3$)

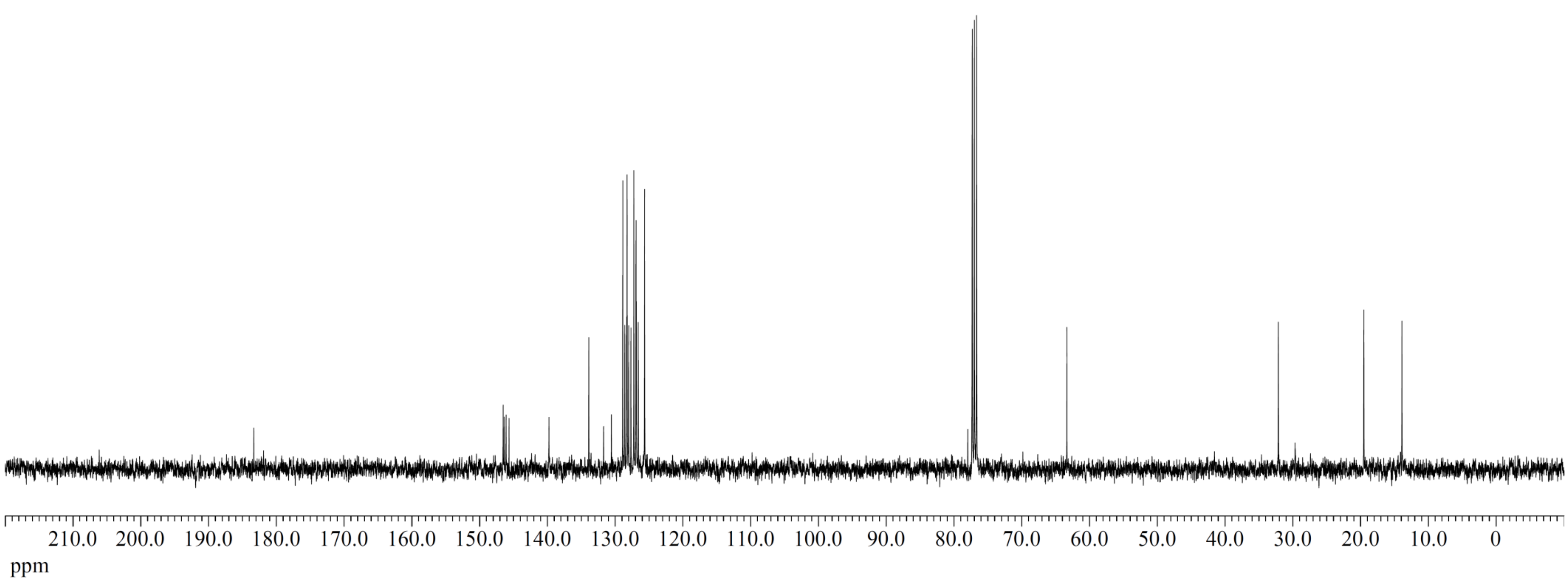

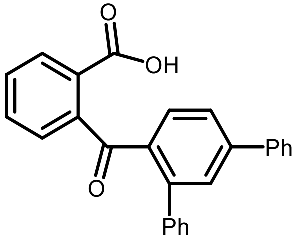


**8**

($^1$H NMR, 400MHz, $CDCl_3$)

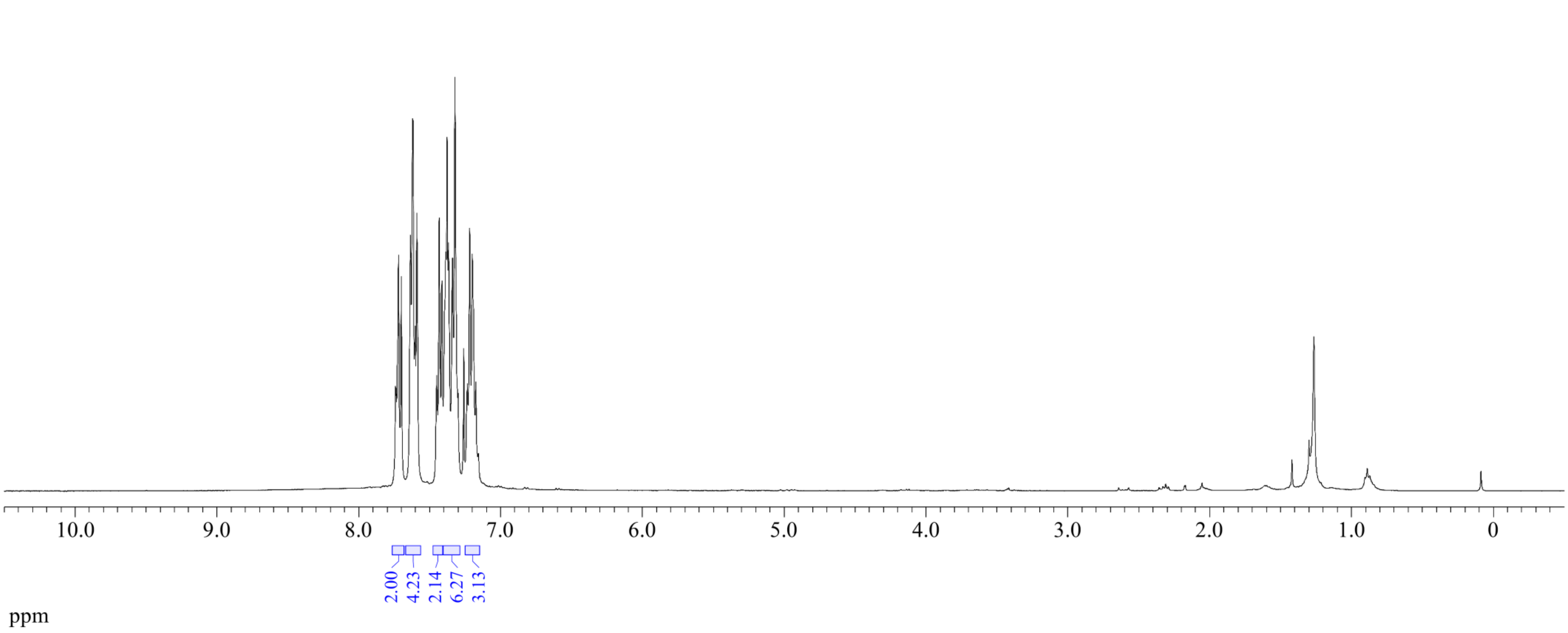

re-COOH-che_carbon-1-2.jdf

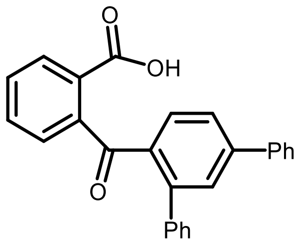

**8**

($^{13}C\{^{1}H\}$ NMR, 101 MHz, $CDCl_3$)

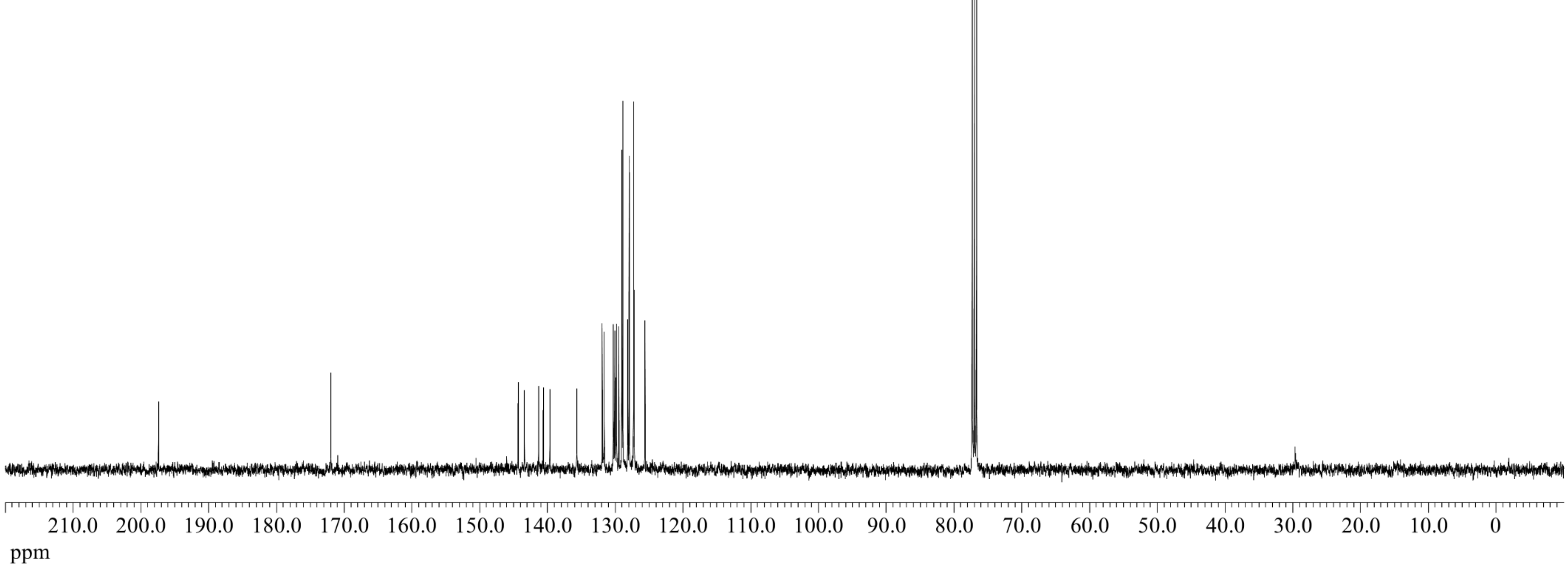

reOEt-nabh4_proton-1-2.jdf

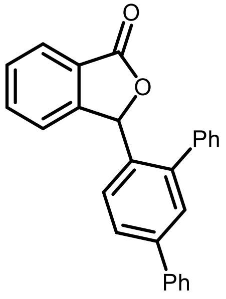


**9**

($^{1}$H NMR, 400MHz, $CDCl_3$)

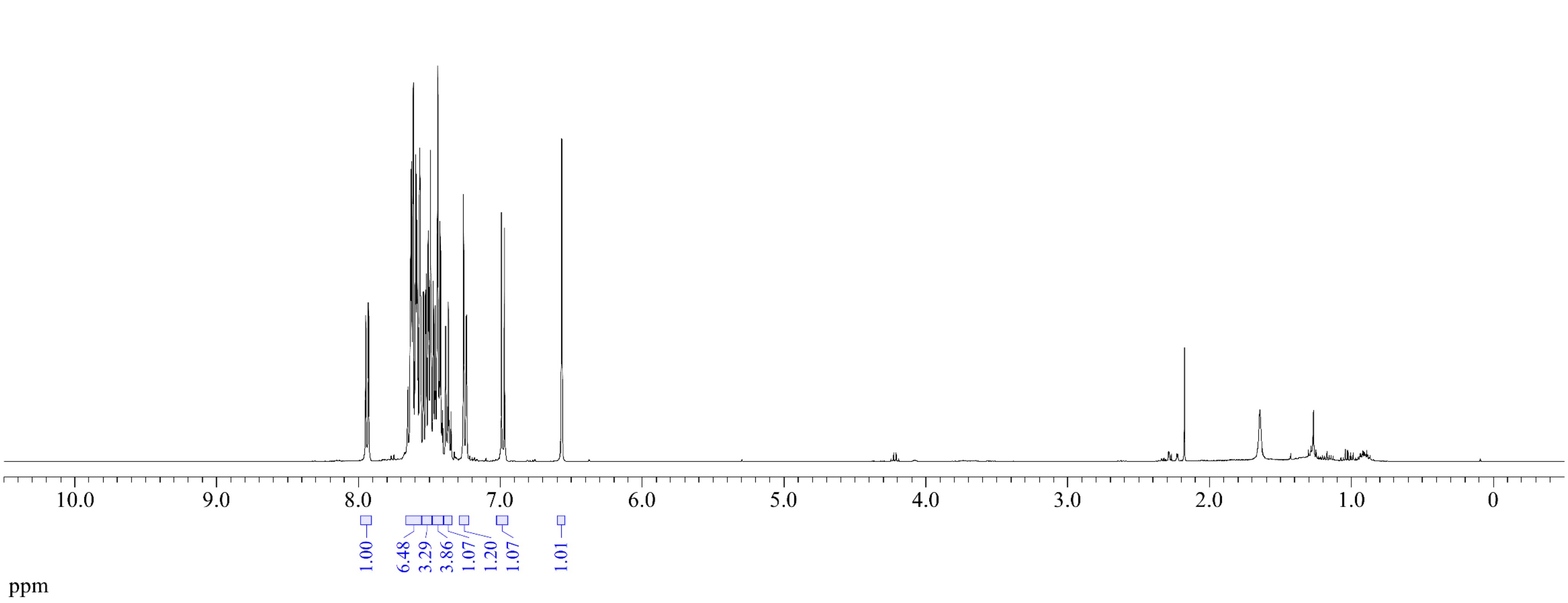

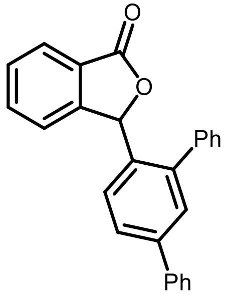

**9**

($^{13}C\{^{1}H\}$ NMR, 101 MHz, $CDCl_3$)

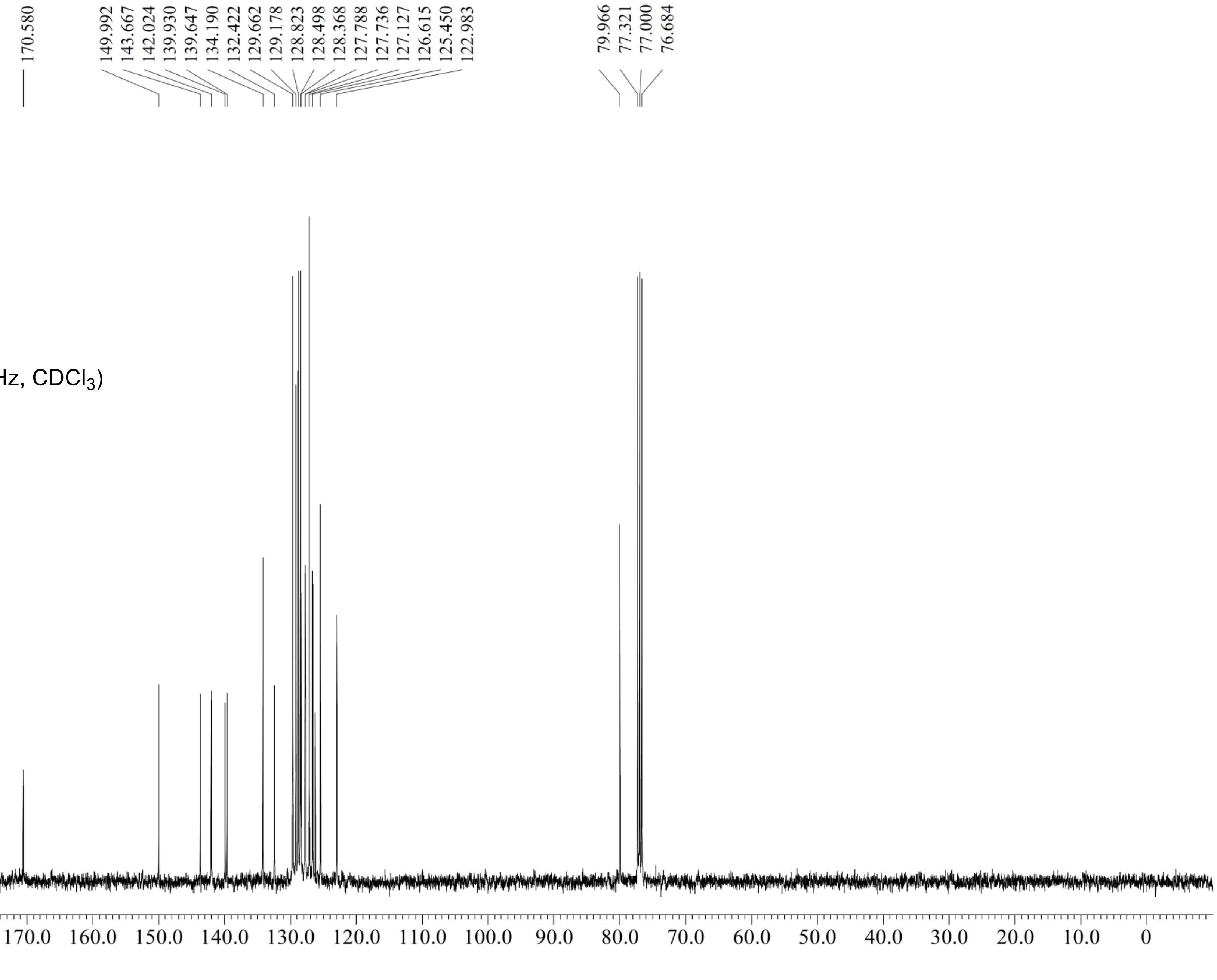

Supplement: Supplementary file 1 — jo4c01296_si_001.pdf [file jo4c01296_si_001.pdf]
